# Synthesis of azulenyl-substituted gold(i)-carbene complexes and investigation of their anticancer activity

**DOI:** 10.1039/d5ra07020a

**Published:** 2025-10-28

**Authors:** Martin C. Dietl, Christopher Hüßler, Matthias Scherr, Zoé M. Frederiksen, Jürgen Graf, Frank Rominger, Matthias Rudolph, Isabella Caligiuri, Laura Tripodi, Flavio Rizzolio, Thomas Scattolin, A. Stephen K. Hashmi

**Affiliations:** a Organisch-Chemisches Institut, Heidelberg University Im Neuenheimer Feld 270 69120 Heidelberg Germany hashmi@hashmi.de http://www.hashmi.de (+49)-6221-54-4205; b Pathology Unit, Department of Molecular Biology and Translational Research, Centro di Riferimento Oncologico di Aviano (CRO), IRCCS Via Franco Gallini 2 33081 Aviano Italy; c Dipartimento di Scienze Molecolari e Nanosistemi, Università Ca’ Foscari Campus Scientifico Via Torino 155 30174 Venezia-Mestre Italy; d Dipartimento di Scienze Chimiche, Università Degli Studi di Padova Via Marzolo 1 35131 Padova Italy thomas.scattolin@unipd.it; e Chemistry Department, Faculty of Science, King Abdulaziz University Jeddah 21589 Saudi Arabia

## Abstract

The direct and atom economic synthesis of azulenyl-substituted gold(i) carbene complexes, based on the modular template synthesis using gold(i) isonitrile complexes and amine nucleophiles, is presented. First, two azulenyl-substituted isonitriles as ligands were synthesized from a functionalizable azulene derivative, the latter stemming from a gold-catalyzed dimerization of internal alkynes. These azulene-bound gold(i) isonitrile complexes allow the smooth nucleophilic attack by both aliphatic and aromatic amines. The newly synthesized azulene-substituted gold(i) carbene complexes were evaluated for *in vitro* anticancer activity against multiple human cancer cell lines. Six lead compounds demonstrated potent and selective cytotoxicity, exceeding that of cisplatin by at least an order of magnitude in resistant and aggressive cancer models. Structure–activity relationship analysis revealed that specific ligand modifications, such as the position of the azulene moiety tethered to the carbene unit or nitrogen-bound ethyl or cyclic groups, are critical for enhancing the anticancer activity.

## Abstract 2

The direct and atom economic synthesis of azulenyl-substituted gold(i) carbene complexes, based on the modular template synthesis using gold(i) isonitrile complexes and amine nucleophiles, is presented.

## Graphical abstract

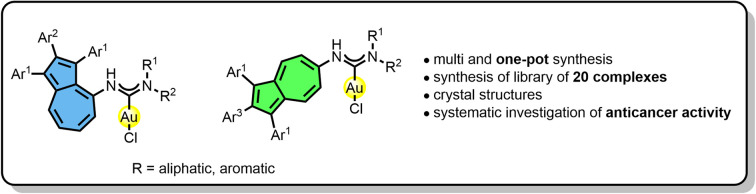

## Introduction

Azulene, the non-benzenoid isomer of naphthalene, consists of a fused five- and seven-membered ring system and represents a distinctive 10π-electron aromatic hydrocarbon.^1^ Its unique electronic structure, marked by a pronounced dipole moment and non-alternant π-system, makes azulene an exceptional chromophore among small hydrocarbons, exhibiting an intriguing blue colour.^2^ Azulene derivatives have found applications in organic materials,^2,3^ molecular sensors,^4–6^ photoswitches,^7^ and in medicinal chemistry.^8,9^ In coordination chemistry, azulene-containing isonitriles have been studied with various transition metals, including Cr(0),^10–14^ Ru(ii),^15^ Co(ii),^16^ Au(i),^17^ W(0),^14^ as well as on gold surfaces.^16,18–20^ Nevertheless, examples of azulene-tethered metal carbene complexes remain extremely scarce. To date, only a single report has described the conversion of azulene-tethered gold(i) isonitriles into carbene complexes *via* reaction with aliphatic amines,^21^ based on methodologies developed by Chugaev,^22–24^ Bonati and Minghetti.^25–27^ Gold complexes, especially those coordinated to carbene ligands, have emerged as promising candidates in the search for novel anticancer agents.^28–38^ These compounds often display potent activity against a broad range of tumour types, including those resistant to conventional treatments. Gold complexes act through different mechanisms, such as inhibition of thioredoxin reductase, disruption of redox homeostasis, and interference with mitochondrial function.^39,40^ Importantly, antiproliferative activity in gold complexes is highly sensitive to structural variations, rendering the rational design of new ligand frameworks a decisive factor for advancing the field.

In this context, the incorporation of azulene into gold(i) carbene complexes is of particular interest. The non-alternant aromatic system introduces an atypical electronic distribution and significant polarity, which can potentially enhance properties relevant to biological applications, such as lipophilicity, membrane permeability and cellular uptake. Thus, azulene represents a promising alternative to conventional aryl scaffolds in gold-based drug design.

**Fig. 1:**
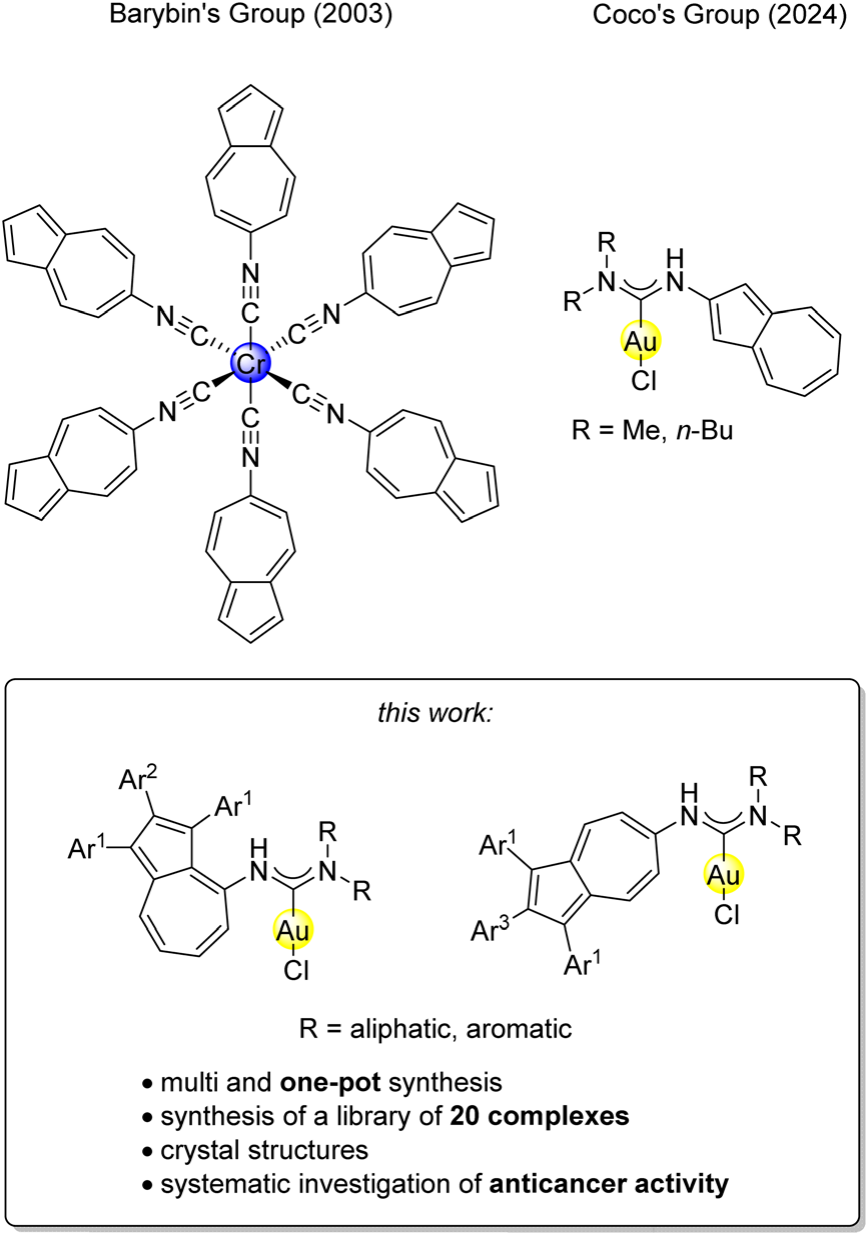
Previous works by the group of Barybin^7^ and Coco^12^ concerning azulene-tethered metal complexes and description of this work.

Herein, we report the first systematic study of azulene-tethered gold(i) carbene complexes. Building on our ongoing research on gold(i) carbene complexes,^41–54^ we establish a general synthetic strategy to access this novel compound class and investigate their cytotoxic activity across a panel of human cancer cell lines (Fig. 1).

## Results and discussion

**Scheme 1:**
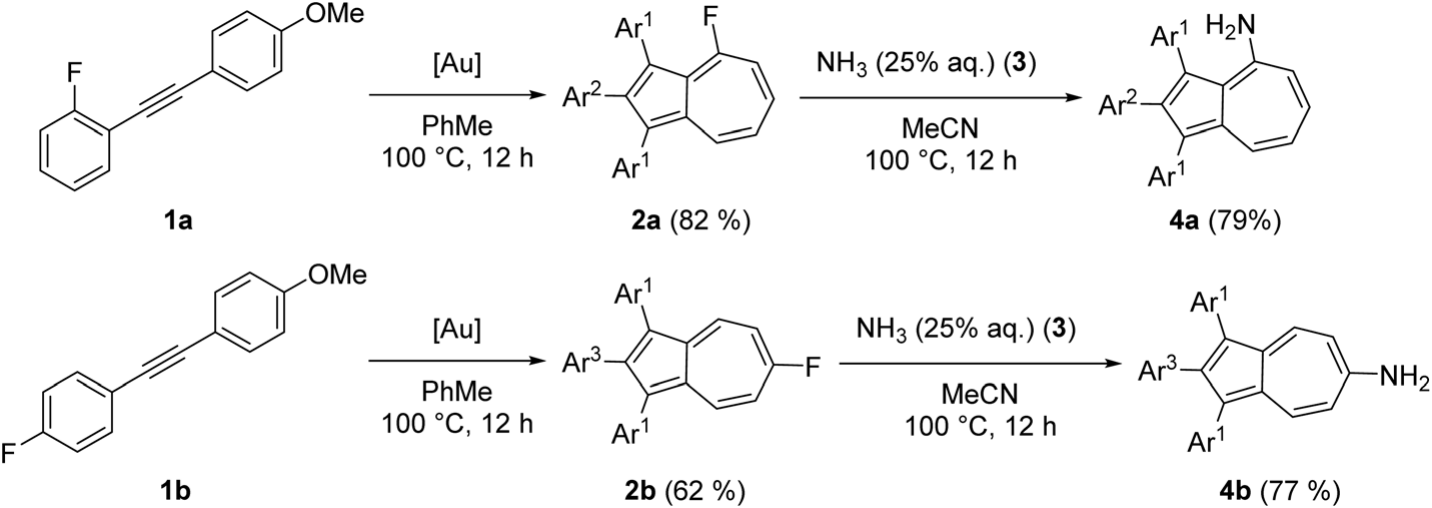
Synthesis of amino-substituted azulenes 4a-b from the azulenes 2a-b, the latter deriving from the dimerization push–pull diarylalkynes 1a or 1b. Conditions: 1a-b (1.00 eq.) [IPr*Au(NCMe)]SbF_6_ (5 mol%), PhMe, 100 °C, 12 h; 2a-b (1.00 eq.), 3 (50 eq., 25% aq.), MeCN, 100 °C, 12 h. Ar^1^ = *para*-methoxyphenyl-, Ar^2^ = *ortho*-fluorophenyl-, Ar^3^ = *para*-fluorophenyl-.

The azulene-containing gold(i) complexes were synthesized *via* the isonitrile-based methodology from our group.^41–50^ Starting from isonitrile gold(i) precursors, the use of different amines as nucleophiles allows for the systematic generation of a library of azulene-tethered gold(i) carbene complexes. This strategy offers a high degree of modularity, as structural variation can be achieved by variation of the nucleophile and the azulene-containing isonitrile. In contrast, alternative approaches, *e.g.*, *via* formamidinium salts require the preparation of a new precursor salt for each complex and therefore do not provide the same flexibility and efficiency.^55^ The synthesis of the azulene core was developed in a previous study by our group.^56^ Therein, a gold-catalyzed dimerization of push–pull diarylalkynes led to substituted azulenes. Therefore, we dimerized the internal alkynes 1a and 1b to form azulenes 2a and 2b used in this study in yields of 82% and 62%, respectively. As described,^56^ substitution of a fluorine substituent attached to the azulene core by suitable nucleophiles is feasible. Previously, amination was achieved by reacting 2a with ammonia (3) in glycol, affording the corresponding aminated azulene 4a. However, since glycol can also act as a nucleophile under these conditions, the reaction not only led to the desired aminated product but also to the formation of a glycol ether–substituted azulene as a side product.^56^ Our updated approach is presented here. Therein, 2a-b were converted to the corresponding aminated azulenes 4a-b, simply by reacting with 50 equivalents of a 25% aqueous solution of ammonia (3) in acetonitrile at 100 °C in a pressure vial. The reaction products 4a-b were obtained selectively in a yield of 79% and 77% (Scheme 1). The successful displacement of the fluorine atom at the seven-membered rings of 2a-b was proven both spectroscopically and by mass spectrometry. While 2a-b depict resonances in the ^19^F NMR spectra found at *δ* = −79.83 and −82.89 ppm corresponding to the fluorine atom attached to the seven-membered ring of an azulene, this signal was not observed in 4a-b, indicating a successful displacement by a nucleophile. In the ^1^H NMR spectra of both substitution products, the resonances of the protons of the formed primary amine are visible at *δ* = 4.35 ppm for 4a and 3.21 ppm for 4b.

**Scheme 2:**
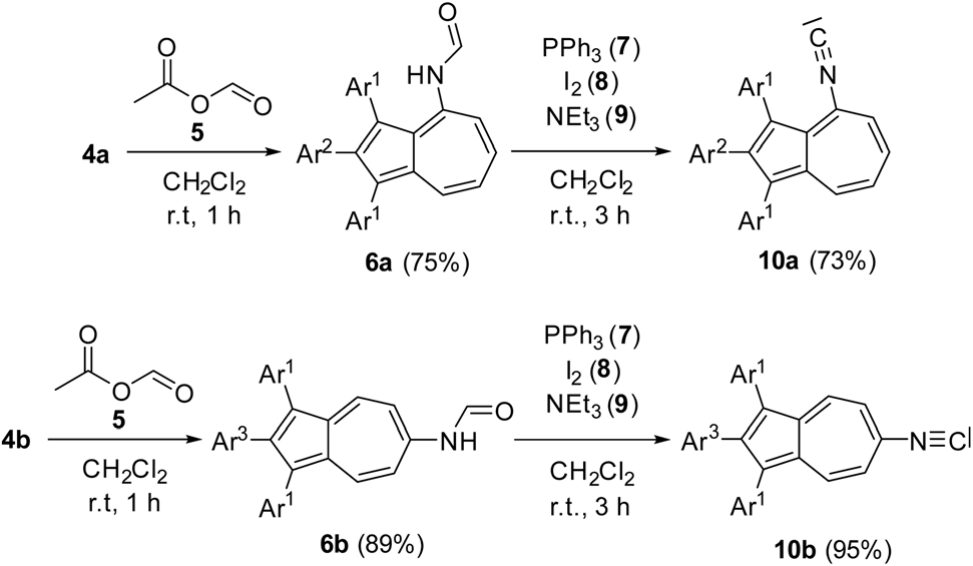
Synthesis of azulene *N*-formamides 6a-b from aminated azulenes 4a-b and acetic formic anhydride (5) and the subsequent dehydration with triphenylphosphine (7), I_2_ (8) and triethylamine (9) to azulene-tethered isonitriles 10a-b. Conditions: 4a-b (1.00 eq.), 5 (1.00 eq.), CH_2_Cl_2_, r.t., 1 h; 6a-b (1.00 eq.), 7 (3.00 eq.), 8 (3.00 eq.), 9 (6.00 eq.), CH_2_Cl_2_, 1–3 h. Ar^1^ = *para*-methoxyphenyl-, Ar^2^ = *ortho*-fluorophenyl-, Ar^3^ = *para*-fluorophenyl-.

**Fig. 2:**
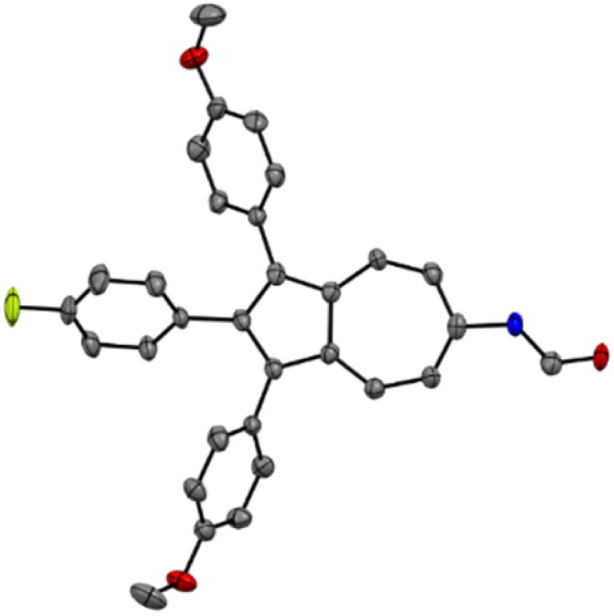
ORTEP molecular solid-state structure of azulene *N*-formamide 6b (CCDC 2449313). Thermal ellipsoids are shown with a 50% probability. Hydrogen atoms were omitted for clarity.

After having obtained the aminated azulenes 4a-b, a suitable reaction protocol for the conversion into the corresponding formamides was explored. The reaction of 4a in toluene and formic acid at 110 °C, did not show any conversion of the starting material after 12 h. This lack of reactivity was attributed to the low nucleophilicity of the amino group of 4a, since its free electron pair might show a stronger delocalization to the electron-deficient seven-membered ring of the azulene. In contrast, treatment of 4a with acetic formic anhydride (5) in dichloromethane at room temperature, afforded complete conversion to the formamide 6a within just 1 h. Under these conditions, the aminated azulenes 4a-b were successfully converted into their corresponding *N*-formamides 6a-b in a yield of 75% and 89% (Scheme 2). The introduction of the formamidyl-group was shown by the presence of the characteristic IR amide band at *ν̃*= 1691 cm^−1^ for 6a and *ν̃* = 1684 cm^−1^ for 6b. Furthermore, the resonances of the formamidyl-group are both visible in the ^1^H NMR and ^13^C NMR spectra of 6a and 6b. In the ^1^H NMR spectra, the resonance of the proton tethered to the nitrogen atom are found in the range between 7.40 and 8.08 ppm. Regarding the proton bound to the carbonyl moiety, its resonances are found in the ^1^H NMR spectrum between 8.42 and 8.88 ppm, while the resonances of the carbonyl carbon atom are visible in the ^13^C NMR spectra between 159.27 and 162.16 ppm. By slow diffusion of *n*-pentane into a concentrated solution of 6b in 1,2-dichloroethane, single crystals suitable for an X-ray single crystal structure analysis were obtained. The molecular structure in the solid state shows the desired connectivity of all atoms, thereby verifying the successful formamidation of 4b with acetic formic anhydride (5) to obtain the *N*-formamide 6b (Fig. 2). To dehydrate the synthesized *N*-formamides 6a-b to the corresponding isonitriles, suitable reaction conditions were subsequently investigated. Initial attempts to dehydrate 6b using phosphoryl chloride and *iso*-propylamine in dichloromethane at room temperature did not result in any conversion.^40^ Similarly, treatment of 6b with the Burgess reagent, a mild dehydrating agent,^57^ led to an unselective decomposition of the starting material after 12 h at room temperature. A microwave-assisted dehydration using cyanuric chloride and triethylamine in dichloromethane under microwave irradiation at 50 °C for 15 minutes showed only trace amounts of the desired isonitrile.^58^ Only upon applying the method developed by the group of Danishefsky,^59^ using triphosgene in dichloromethane at 0 °C, led to the formation of the desired isonitrile 10b in 47% yield after 2 h. A further improvement was achieved using a protocol reported by the group of Luo for the synthesis of aromatic isonitriles.^60^ With this method, 6b was converted to the target isonitrile 10b with triphenylphosphine (7), iodine (8), and triethylamine (9) in dichloromethane at room temperature in a yield of 95% after only 1 h. Given the higher yield, shorter reaction time, and use of less toxic reagents compared to triphosgene, all subsequent dehydrations of 6a-b were carried out using this method yielding 10a in 73% and 10b in 95% (Scheme 2). The successful outcome of the dehydrations could be monitored by means of IR-spectroscopy. The IR-stretching mode of the formamidyl-groups of 6a-b vanished, while the characteristic IR-stretching mode of the generated isonitrile group appeared at *ν̃* = 2109 cm^−1^ for 10a and *ν̃* = 2108 cm^−1^ for 10b.

**Scheme 3:**
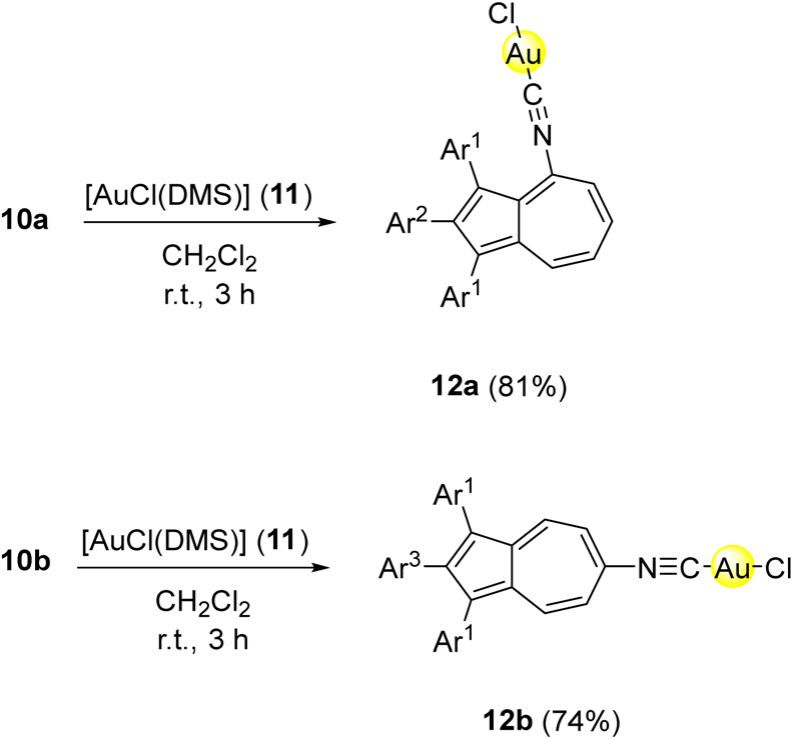
Synthesis of azulene-tethered gold(i) isonitrile complexes 12a-b from azulene-tethered isonitriles 10a-b and [AuCl(DMS)] (11). Conditions: 10a-b (1.00 eq.), 11 (1.00 eq.), CH_2_Cl_2_, r.t., 3 h. Ar^1^ = *para*-methoxyphenyl-, Ar^2^ = *ortho*-fluorophenyl-, Ar^3^ = *para*-fluorophenyl-.

**Fig. 3:**
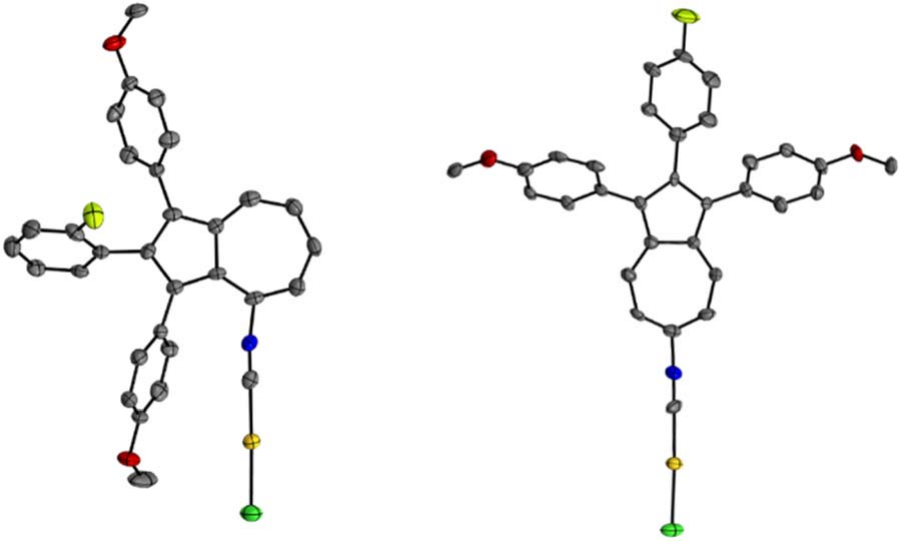
ORTEP molecular solid-state structure of azulene-tethered gold(i) isonitrile complexes 12a (CCDC 2449314) and 12b (CCDC 2449315). Thermal ellipsoids are shown with a 50% probability. Hydrogen atoms were omitted for clarity.

Subsequently, isonitriles 10a and 10b were reacted with the commercially available [AuCl(DMS)] (11) in dichloromethane to afford the corresponding gold(i) complexes *via* ligand exchange. Full conversion of the starting material was observed after only 1 h by analysis of TLC. After purification by flash column chromatography, the corresponding gold(i) isonitrile complexes 12a and 12b were isolated in yields of 81% and 74%, respectively (Scheme 3). By slow diffusion of *n*-pentane into saturated solutions of 12a-b, single crystals suitable for an X-ray single crystal structure analysis were obtained. Both molecular structures in the solid state show the successful coordination of the gold(i) metal centre to the isonitrile ligands 10a-b in the linear coordination geometry typical for gold(i) (Fig. 3). However, no intermolecular aurophilic interactions^61–63^ were observed in 12a and **12b**.

**Scheme 4:**
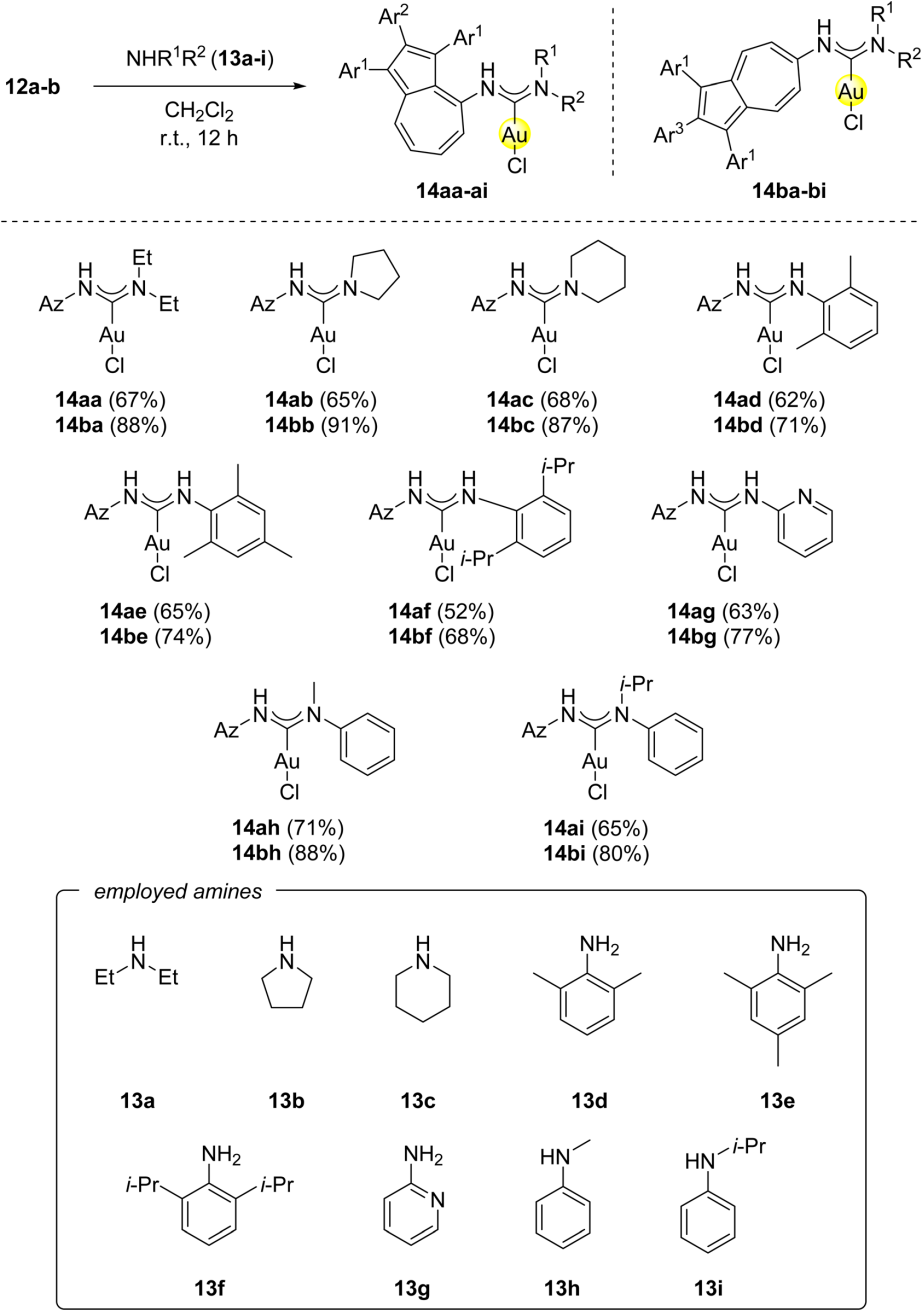
Synthesis of azulene-tethered gold(i) *N*-acyclic carbene complexes 14aa–ai and 14ba–bi from azulene-tethered gold(i) isonitrile complexes 12a-b and primary or secondary amines 13a–i. Conditions: 12a-b (1.00 eq.), 13a–i (1.05–1.50 eq.), CH_2_Cl_2_, r.t., 12 h. Ar^1^ = *para*-methoxyphenyl-, Ar^2^ = *ortho*-fluorophenyl-, Ar^3^ = *para*-fluorophenyl-.

The gold(i) isonitrile complexes 12a and 12b were then reacted with 1.05 eq. of various aliphatic and aromatic amines 13a-i in dichloromethane at room temperature, affording the azulene-tethered gold(i) *N*-acyclic carbene complexes 14aa–ai and 14ba–bi in moderate to good yields (Scheme 4). The reactivity trends reflect differences in nucleophilicity and steric demand of the amines, as well as the steric properties of complexes 12a and 12b. Complex 12a, where the azulenyl-ligand is tethered to the 4-position, is sterically more hindered than 12b, resulting in lower yields of 14aa–ai with 52–71% compared to 14ba–bi with 68–91%. Nucleophilic and sterically less demanding amines, such as diethylamine (13a), pyrrolidine or piperidine (13c), gave the highest yields. In contrast, sterically encumbered aromatic amines like 2,6-dimethylaniline (13d), mesitylamine (13e) and 2,6-diisopropylaniline (13f) showed a decreased reactivity, leading to lower yields of 52–65% for 14ad–af and 68–74% for 14bd–bf. 2-Aminopyridine (13g) as a sterically not demanding but weakly nucleophilic amine, gave moderate yields of 63% for 14ag and 77% for 14bg. *N*-alkylated amines (13h–i) displayed enhanced nucleophilicity, improving the obtained yields relative to their primary analogues. The sterically demanding *iso*-propyl substituent in 13i lowered yields with 65% for 14ai and 80% for 14bi, compared to the methyl-substituted analogue 13h with yields of 71% for 14ah and 88% for 14bh.

**Fig. 4:**
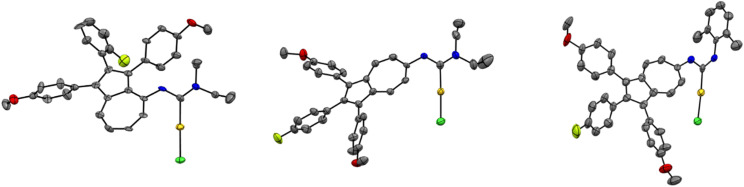
ORTEP molecular solid-state structure of azulene-tethered gold(i) *N*-acyclic carbene complexes 14aa (left) (CCDC 2449316), 14ba (middle) (CCDC 2449317) and 14bd (right) (CCDC 2449318). Thermal ellipsoids are shown with a 50% probability. Hydrogen atoms were omitted for clarity.

The successful outcome of all reactions was assessed analytically by means of NMR, IR and UV-Vis spectroscopy and mass spectrometry. Bulk purity was determined to be over 95% of 14aa–ai and 14ba–bi by elemental analysis. While the starting materials 12a-b both possessed an isonitrile group which depicted a characteristic band in the IR-spectra, after the addition of the amines in the IR-spectra of the gold(i) carbene complexes no signal belonging to the isonitrile moiety was observed. However, in the ^13^C NMR spectra of 14aa–ai, as well as 14ba–bi, resonances between *δ* = 188.81 ppm and 194.04 ppm were observed, that indicate the formation of a carbene centre. Furthermore, the resonances of the protons in the ^1^H NMR spectra, as well as the high-resolution mass spectra indicate the successful formation of the desired gold(i) carbene complexes. Diffusion of *n*-pentane into saturated solutions of 14aa, 14ba and 14bd led to single crystals suitable for X-ray diffraction (Fig. 4), that on top of the previously presented analytics confirm the proper connectivity of all atoms and hence prove the successful outcome of the reaction. The molecular structures in the solid state of 14aa, 14ba and 14bd show the typical linear coordination geometry of gold(i) metal centres (Fig. 4). While no intermolecular aurophilic interactions^61–63^ were observed for complexes 14aa and 14ba, compound 14bd displayed a Au–Au distance of 3.2315(10) Å.

**Scheme 5:**
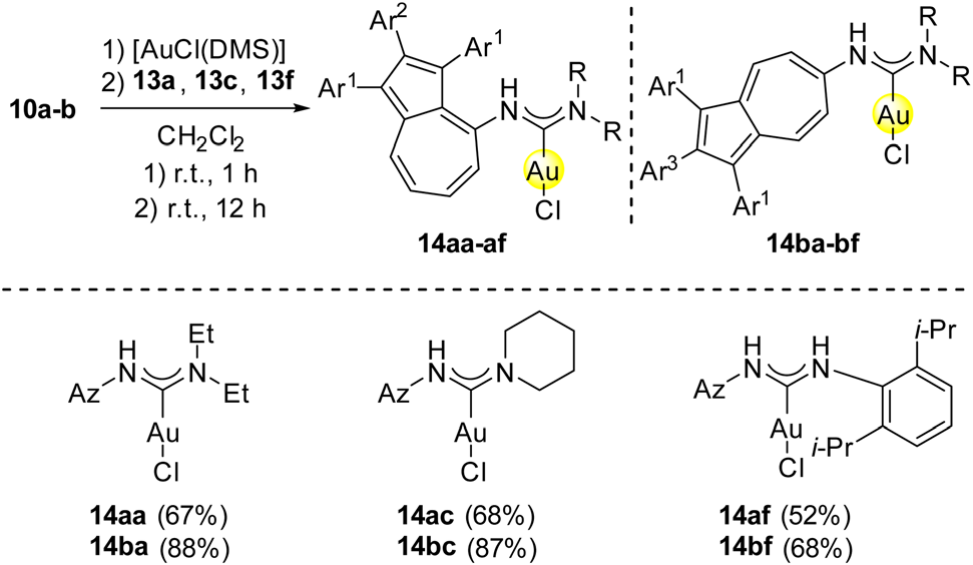
One-pot synthesis of azulene-tethered gold(i) *N*-acyclic carbene complexes 14aa, 14ba, 14ac, 14bc, 14af and 14bf from [AuCl(DMS)] (11) azulene-tethered isonitriles 10a-b and diethylamine (13a), piperidine (13c) and 2,6-diisopropylaniline (13f). Conditions: 11 (1.00 eq.), 10a-b (1.00 eq.), 13a (1.05 eq.), CH_2_Cl_2_, r.t., 13 h. Ar^1^ = *para*-methoxyphenyl, Ar^2^ = *ortho*-fluorophenyl, Ar^3^ = *para*-fluorophenyl.

**Scheme 6:**
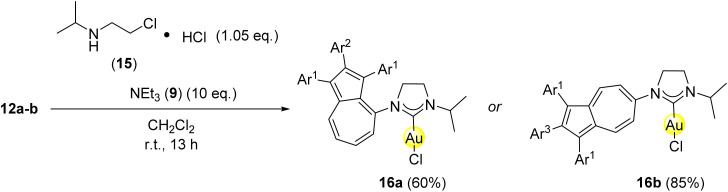
Synthesis of azulene-tethered gold(i) saturated *N*-heterocyclic carbenes 16a-b from azulene tethered isonitrile complexes 12a-b, *N*-(2-chloroethyl)propan-2-amine hydrochloride (15) and triethylamine (9). Conditions: 12a-b (1.00 eq.), 15 (1.05 eq.), 9 (10.0 eq.), CH_2_Cl_2_, r.t., 12 h. Ar^1^ = *para*-methoxyphenyl-, Ar^2^ = *ortho*-fluorophenyl-, Ar^3^ = *para*-fluorophenyl-.

After the successful multistep synthesis of the azulenyl-substituted gold(i) carbene complexes 14aa–ai and 14ba–bi, the feasibility of a one-pot protocol was explored. This would offer a direct approach to the final products starting from the commercially available [AuCl(DMS)] (11), azulene containing isonitriles 10a-b and aliphatic, as well as aromatic amines and consequently offer a greener approach to the formation of the desired reaction products, since workup steps of intermediates including organic solvents would be avoided. The feasibility of a one-pot approach was investigated with diethylamine 13a and piperidine 13c as examples of aliphatic amines, as well as 2,6-diisopropylaniline 13f as an aromatic amine. Therefore, [AuCl(DMS)] 11 was firstly reacted with either 10a or 10b in dichloromethane for 1 h at room temperature. Afterwards, the respective amine was added to the reaction mixture. In all cases the full consumption of the starting materials was observed *via* analysis of TLC after an additional reaction time of 12 h. With the one-pot protocol, the azulenyl-substituted gold(i) carbene complexes 14aa, 14ba, 14ac, 14bc, 14af and 14bf were formed in similar yields compared to the multistep approach (Scheme 5). Overall, the one-pot approach offers a valuable, greener and more time economic alternative to a multistep synthesis towards the reaction products. After the successful multistep, as well as one-pot synthesis of azulene tethered gold(i) *N*-acyclic complexes 14aa, 14ba, 14ac, 14bc, 14af and 14bf, next an approach towards azulene-substituted gold(i) saturated *N*-heterocyclic carbene complexes^46–48^ was evaluated. For this reason, the gold(i) isonitrile complexes 12a-b were reacted with 1.05 equivalents of *N*-(2-chloroethyl)propan-2-amine hydrochloride (15), that was converted *in situ* to the reactive *N*-(2-chloroethyl)propan-2-amine by addition of triethylamine (9) as a base in dichloromethane for 12 h. The corresponding gold(i) saturated *N*-heterocyclic carbene complexes 16a-b were obtained in moderate to good yields of 60% and 85%, respectively (Scheme 6). The discrepancy of the yields is again attributed to the steric demand of the azulenyl-tethered isonitrile at the starting material 12a-b. UV-Vis absorption spectra of all and cyclic voltammetry of selected complexes were obtained and are available in the SI of this manuscript.

### Anticancer activity of azulene-substituted gold(i)-carbene complexes

As anticipated in the introduction section, over the past two decades, interest in gold-based therapeutics has grown significantly, driven by their unique chemical properties and modes of action that differ markedly from traditional platinum-based drugs like cisplatin.^28–38^ Unlike platinum complexes, which typically bind DNA directly, gold compounds tend to target proteins and enzymes, making them attractive for tackling cancers that have developed resistance to DNA-damaging agents. In particular, the high affinity of gold(i) for thiol and selenol groups renders it highly effective in targeting cysteine- and selenocysteine-containing proteins, which play central roles in redox regulation and cancer cell survival.^39,40^

Several gold compounds, such as auranofin, originally approved for the treatment of rheumatoid arthritis, have shown promising anticancer activity in both *in vitro* and *in vivo* models, prompting renewed interest in the medicinal chemistry of gold. Recent efforts have focused on the development of gold complexes with tailored ligands to modulate pharmacokinetic properties, cellular uptake, and target specificity.^28–38^ Among these, *N*-heterocyclic carbene (NHC) ligands have emerged as particularly valuable due to their strong σ-donating ability, resistance to oxidation, and ease of structural modification.

Gold NHC complexes can be finely tuned to enhance lipophilicity, improve cellular permeability, or incorporate targeting moieties, thus enabling the development of more selective and less toxic anticancer agents.^28–38^ Moreover, the modularity of carbene ligands allows for the rational design of compounds with dual or multi-target activity, which is increasingly recognized as a key strategy in addressing cancer heterogeneity and drug resistance.

With the aim of determining whether the wide range of azulenyl-substituted gold(i) carbene complexes synthesized in this work exhibit potential cytotoxicity, their *in vitro* anticancer activity was evaluated across a panel of human cancer cell lines. Prior to these investigations, the stability of representative complexes was tested in the cell medium. Exemplarily, ^1^H NMR spectra of 14ab and 14ba in a 100 mM saline solution in D_2_O and DMSO-d_6_ did not show signs of decomposition after 96 h at room temperature. This ensured that the subsequent tests reflected the properties of intact complexes. The investigated cell lines included A2780 (cisplatin-sensitive ovarian carcinoma), A2780*cis* (cisplatin-resistant ovarian carcinoma), MDA-MB-231 (triple-negative breast cancer), and U87 (glioblastoma).

For comparison, the cytotoxic profile of cisplatin, a widely used platinum-based chemotherapeutic, was determined under the same conditions.

These cell lines were selected to cover a range of cancer types with distinct biological and clinical relevance. A2780 and its cisplatin-resistant variant A2780*cis* provided a model to assess both chemosensitive and chemoresistant ovarian cancer phenotypes. The triple-negative breast cancer cell line MDA-MB-231 was included as a representative of an aggressive breast cancer subtype characterized by poor prognosis and limited therapeutic options. In addition, U87 cells were chosen as a well-established glioblastoma model, allowing us to explore the applicability of our approach in a central nervous system tumor setting. Taken together, this panel of cell lines was chosen to investigate the potential efficacy of our strategy across different tumor origins and resistance profiles.

The data obtained in this work, expressed as IC_50_ values and summarized in Table 1, clearly demonstrate that only a subset of the tested complexes exhibit significant cytotoxic activity, highlighting the substantial influence of carbene ligand decoration on antiproliferative effects. This observation suggests that subtle changes in ligand structure can markedly impact the biological performance of these gold(i) carbene complexes.

**Table 1:** Antiproliferative activity on A2780, A2780*cis*, MDA-MB-231 and U87 cancer cell lines^a^

Compound	IC_50_ (μM)			
	A2780	A2780*cis*	MDA-MB-231	U87
Cisplatin	2.5 ± 0.2	70 ± 10	60 ± 10	30 ± 10
14aa	3.4 ± 0.3	2.0 ± 0.6	0.95 ± 0.05	1.8 ± 0.2
14ba	7 ± 2	3 ± 1	1.4 ± 0.2	5.4 ± 0.2
14ab	3.5 ± 0.1	1.7 ± 0.2	1.7 ± 0.3	2.7 ± 0.3
14bb	80 ± 10	70 ± 10	70 ± 10	90 ± 10
14ac	4.1 ± 0.4	3 ± 1	3.2 ± 0.3	4.9 ± 0.1
14bc	70 ± 20	>100	25 ± 2	10 ± 2
14ad	90 ± 10	>100	>100	>100
14bd	>100	>100	>100	>100
14ae	>100	>100	>100	>100
14be	>100	>100	>100	>100
14af	>100	>100	>100	>100
14bf	>100	>100	>100	>100
14ag	80 ± 10	>100	80 ± 20	80 ± 10
14bg	>100	>100	40 ± 10	80 ± 20
14ah	70 ± 10	50 ± 20	9 ± 2	6.3 ± 0.2
14bh	70 ± 10	>100	10 ± 3	5.2 ± 0.3
14ai	80 ± 10	7.1 ± 0.1	3.8 ± 0.7	17 ± 4
14bi	80 ± 10	30 ± 1	15 ± 3	14 ± 1
16a	4.7 ± 0.2	1.1 ± 0.4	1.0 ± 0.2	1.5 ± 0.5
16b	8 ± 1	3.3 ± 0.7	3 ± 1	6.2 ± 0.3

a Data after 96 h of incubation. Stock solutions in DMSO for all complexes; stock solutions in H_2_O for cisplatin. A2780 (cisplatin-sensitive ovarian cancer cells), A2780*cis* (cisplatin-resistant ovarian cancer cells), MDA-MB-231 (triple-negative breast cancer) and U87 (glioblastoma). Data are expressed as mean values ± standard deviation (SD) from measurements performed in triplicate.

Focusing specifically on the series of complexes bearing *N*-acyclic carbene ligands, keeping the substituents R^1^ and R^2^ on the carbene nitrogen constant, the most active derivatives were those featuring the azulene moiety attached to the carbene nitrogen atom in the 4-position of azulene featuring an *ortho*-fluorophenyl substituent (*e.g.*, 14aa*vs.*14ba, 14ab*vs.*14bb, 14ac*vs.*14bc). When the azulene moiety remains constant, the substituents on the opposite nitrogen that most enhance cytotoxicity include: (i) two ethyl groups (14aa and 14ba), (ii) a pyrrolidine ring (14ab), and (iii) a piperidine ring (14ac).

Both complexes containing *N*-heterocyclic carbene (NHC) ligands (16a and 16b) also showed high cytotoxicity across the various cancer cell lines tested. Their activity was comparable to that of the most potent *N*-acyclic derivatives (14aa, 14ba, 14ab, and 14ac). More specifically, these six lead compounds displayed cytotoxic profiles similar to those of cisplatin in the cisplatin-sensitive ovarian cancer cell line (A2780). Importantly, our compounds demonstrated significantly higher activity than cisplatin against aggressive and treatment-resistant cancer models, including glioblastoma (U87), triple-negative breast cancer (MDA-MB-231), and cisplatin-resistant ovarian cancer (A2780*cis*).

In these models, our six compounds were at least an order of magnitude more active than cisplatin, underscoring their potential to overcome limitations of current chemotherapeutics in hard-to-treat cancers.

Excluding the six previously mentioned compounds that exhibited activity across all tested cancer cell lines, many of the remaining complexes were essentially inactive against tumour cells. Nearly all of these inactive compounds share a common structural feature: the presence of a hydrogen atom bound to both carbene nitrogen atoms (*e.g.*, 14ad, 14bd, 14ae, 14be, 14af, 14bf, 14ag, and 14bg).

Interestingly, four other complexes, namely 14ah, 14bh, 14ai, and 14bi, each containing one *N*-alkyl group such as methyl or *iso*-propyl and one phenyl group attached to the carbene nitrogen, showed selective cytotoxicity in only two or three of the tested cancer cell lines. Notably, their activity was predominantly observed in the more cisplatin-resistant models. This selective behaviour toward hard-to-treat cancers highlights their potential as promising candidates for further development, alongside the six broadly active lead compounds.

## Conclusions

Herein, we describe the synthesis of twenty new azulene-tethered gold(i) *N*-acyclic carbene complexes by the template-assisted addition of primary or secondary amines onto gold(i) isonitrile complexes. Alternatively, the synthesis could be performed in a one-pot reaction using commercially available [AuCl(DMS)], an azulene-tethered isonitrile, and an amine. The required isonitriles were prepared from an azulene derivative obtained by gold-catalyzed dimerization of push–pull bisarylalkynes. The optoelectronic features of these complexes were studied by means of UV-Vis spectroscopy, as well as cyclovoltammetry. The newly synthesized azulene-tethered gold carbene complexes were tested for *in vitro* cytotoxicity against a panel of human cancer cell lines, including both cisplatin-sensitive and -resistant ovarian carcinoma, triple-negative breast cancer, and glioblastoma. A subset of the compounds showed significant anticancer activity, with structure–activity relationship analysis revealing that specific ligand modifications-such as introducing the azulene moiety in its 4-position to the nitrogen atom bound to the carbene unit, the position of the fluorine in the ligand scaffold and ethyl or cyclic groups on the carbene nitrogen-are critical for effectiveness. Six leading compounds, including both *N*-acyclic and *N*-heterocyclic carbene derivatives (14aa, 14ba, 14ab, 14ac, 16a and 16b) demonstrated cytotoxicity comparable to cisplatin in sensitive models and superior activity-up to an order of magnitude higher-against resistant and aggressive tumours. In contrast, most inactive compounds shared the presence of a hydrogen atom on the carbene nitrogen. Four additional derivatives (14ah, 14bh, 14ai, and 14bi) exhibited selective cytotoxicity in only the more treatment-resistant lines, highlighting potential for targeted action. These ten compounds represent the most promising candidates for further investigation in more complex biological systems, such as patient-derived organoids and animal models; alongside the development of gold carbene complexes with similar or enhanced anticancer selectivity.

## Author contributions

Martin C. Dietl: conceptualization, investigation, methodology, formal analysis and validation of analytical data, visualisation, writing-original draft; Christopher Hüßler: investigation; Matthias Scherr: investigation; Zoé M. Frederiksen: investigation; Jürgen Graf: data curation, formal analysis; Frank Rominger: data curation, formal analysis, validation and visualisation of X-ray Single Crystal Data; Matthias Rudolph: project administration; Isabella Caligiuri: investigation; Laura Tripodi: investigation; Flavio Rizzolio: investigation, methodology formal analysis and validation of analytical data; Pablo A. Nogara: data curation, formal analysis, validation and visualisation of molecular docking data, writing-review and editing; Laura Orian: data curation, formal analysis, validation and visualisation of DFT data, writing-review and editing; Thomas Scattolin: supervision, writing-original draft, data curation; A. Stephen K. Hashmi: supervision, project administration, resources.

## Conflicts of interest

There are no conflicts to declare.

## Supplementary Material

RA-015-D5RA07020A-s001

RA-015-D5RA07020A-s002

## Data Availability

CCDC 2449313, 2449314, 2449315, 2449316, 2449317 and 2449318 contain the supplementary crystallographic data for this paper.^64*a*–*f*^ The authors declare that all the data used for this manuscript can be found in its supplementary information (SI). Supplementary information is available. See DOI: https://doi.org/10.1039/d5ra07020a.
