# Supplementary material for: Synthesis of azulenyl-substituted gold(i)-carbene complexes and investigation of their anticancer activity
Source: RSC Adv. 2025 Oct 28;15(49):41260–9. doi: 10.1039/d5ra07020a (PMC12560230; doi:10.1039/d5ra07020a)
Supplement: RA-015-D5RA07020A-s001 [file RA-015-D5RA07020A-s001.pdf]

Supplementary Information (SI) for RSC Advances.
This journal is © The Royal Society of Chemistry 2025

Supporting Information for

Synthesis of Azulenyl-Substituted Gold(I)-Carbene Complexes and Investigation of their Anticancer Activity

Martin C. Dietl,[a] Christopher Hüßler,[a] Matthias Scherr,[a] Zoé Frederiksen,[a] Jürgen Graf,[a] Frank Rominger,[a] Matthias Rudolph,[a] Isabella Caligiuri,[b] Laura Tripodi,[c] Flavio Rizzolio,[b,c] Thomas Scattolin,[d]* A. Stephen K. Hashmi[a],[e]*

[a] Organisch-Chemisches Institut, Heidelberg University, Im Neuenheimer Feld 270, 69120 Heidelberg, Germany, Fax: (+49)-6221-54-4205; e-mail: hashmi@hashmi.de (homepage: http://www.hashmi.de)

[b] Pathology Unit, Department of Molecular Biology and Translational Research, Centro di Riferimento Oncologico di Aviano (CRO), IRCCS, via Franco Gallini 2, 33081, Aviano, Italy.

[c] Dipartimento di Scienze Molecolari e Nanosistemi, Università Ca' Foscari, Campus Scientifico Via Torino 155, 30174, Venezia-Mestre, Italy

[d] Dipartimento di Scienze Chimiche, Università degli Studi di Padova, via Marzolo 1, 35131 Padova, Italy. e-mail: thomas.scattolin@unipd.it

[e] Chemistry Department, Faculty of Science, King Abdulaziz University, Jeddah 21589, Saudi Arabia

Table of Contens

List of Abbreviations 2

1 General Remarks 3

2 Synthesis of Compounds 5

3 Cell viability assay 46

4 NMR Spectra 48

5 IR Spectra 92

6 Single Crystal Structures 106

7 IC_{50} plots for complex 14aa 113

8 Stability of complexes 14ab and 14ba 115

9 References 117

List of Abbreviations

ATR	Attenuated Total Reflectance
Calcd.	Calculated
DCM	Dichloromethane
EA	Ethyl Acetate
HR-MS	High Resolution Mass Spectrometry
IR	Infrared Spectroscopy
J	Scalar coupling
K	Kelvin
MALDI	Matrix-Assisted Laser Desorption /Ionization
MHz	Megahertz
MP	Melting Point
NMR	Nuclear Magnetic Resonance
PE	Petroleum Ether
r.t.	Room Temperature
UV-Vis	Ultraviolet-Visible Spectroscopy

1 General Remarks

All chemicals and solvents were purchased from the commercial suppliers ABCR, BLDPharm, Chempur, Fluorochem, TCI or Sigma-Aldrich. All purchased substances were used without prior purification. Deuterated solvents were purchased at Deutero, Eurisotop or Sigma-Aldrich. Absolute reagent grade solvents were filled in Schlenk flasks under nitrogen atmosphere from the solvent purification system MB-SPS-800 Benchtop or purchased from the aforementioned commercial suppliers. Reactions under inert conditions were conducted in flame-dried Schlenk flasks under nitrogen atmosphere. Analytical thin layer chromatography was performed on precoated Macherey-Nagel POLYGRAM® SIL G/UV254 or Merck TLC Silica gel Gel 60 F254 aluminium TLC plates. Flash column chromatography was performed using silica gel (0.04 – 0.063 mm / 230 – 400 mesh ASTM) obtained from Macherey-Nagel as a stationary phase. Solvent mixtures of petroleum ether (PE), ethyl acetate, dichloromethane (DCM) or Methanol (MeOH) were used as mobile phases. Melting points were measured with a BÜCHI B-540 melting point apparatus. NMR spectra were obtained at the spectrometers Bruker Avance III 300, Bruker Fourier 300, Bruker Advance DRX 300, Bruker Advance III 400, Bruker Advance III 500, Bruker Advance III 600 and Bruker Avance Neo 700. All chemical shifts δ are given in parts per million (ppm) and coupling constants J are given in Hertz (Hz). ^{1}H, and ^{13}C NMR spectra were calibrated relatively to residual solvent signals, such as $CDCl_3$ (7.26 ppm, 77.16 ppm), CD_2Cl_2 (5.32 ppm, 53.84 ppm), or C_6D_6 (δ 7.16 ppm, 128.06 ppm). The following abbreviations were used to indicate multiplicities in the ^{1}H NMR spectra: s (singlet), d (doublet), t(triplet), q (quartet), m (multiplet). The following abbreviations were used to indicate multiplicities in the ^{13}C NMR spectra: s (quartenary carbon atom, CR_4), d (tertiary carbon atom, CHR_3), t (secondary carbon atom, CH_2R_2), q (primary carbon atom CH_3R). Multiplicities were determined by ^{13}C DEPT and 2D ^{1}H, ^{13}C NMR experiments. All ^{13}C NMR Spectra were recorded with decoupling of the ^{1}H and ^{19}F nucleus All ^{19}F NMR spectra were recorded with decoupling of the ^{1}H nucleus. All raw data was processed using the software MestReNova 14.3. Infrared spectra were obtained from a FT-IR Bruker LUMOS with a Germanium ATR crystal. Mass spectra were measured at the MS facility of chemistry department of Heidelberg University. Electron Ionization (EI) spectra were measured an on JEOL JMS-700 or on a JEOL AccuTOF GCx spectrometer. Matrix-assisted laser desorption/ionization (MALDI) spectra were obtained from a Bruker ApexQe FT-ICR-MS or from a Bruker Autoflex Speed time-of-flight spectrometer. UV/Vis spectra were recorded on a JASCO V-670 spectrophotometer in the specified solvents. Single-crystal XRD analyses were performed at the chemistry department of Heidelberg University. The XRD data was collected

from a Stoe Stradivari with a Cu-microsource and Pilatus detector or from a BRUKER APEX II Quazar with a Mo-microsource. Single crystal structures were processed using the software Mercury 3.10.3. Cyclic voltammograms were measured on a VERSASTAT3-200 potiontiostat with a glassy carbon working electrode, a silver reference electrode and a platinum coated titanium counter electode. All measurements were performed in a 0.1M Solution o0f tetrabutylammonium hexafluorophosphate in degassed dichloromethane with ferrocene as an internal standard.

2 Synthesis of Compounds

2.1 General Procedure for the Synthesis of Azulene-tethered Carbene Gold(I) Complexes from Isonitrile Gold(I) Complexes (GP1)

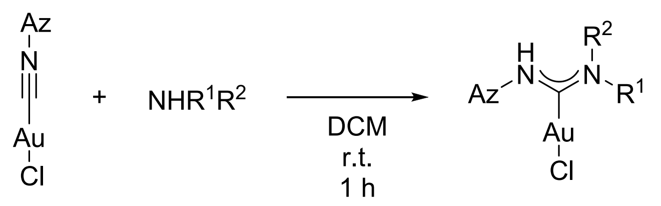

Az =

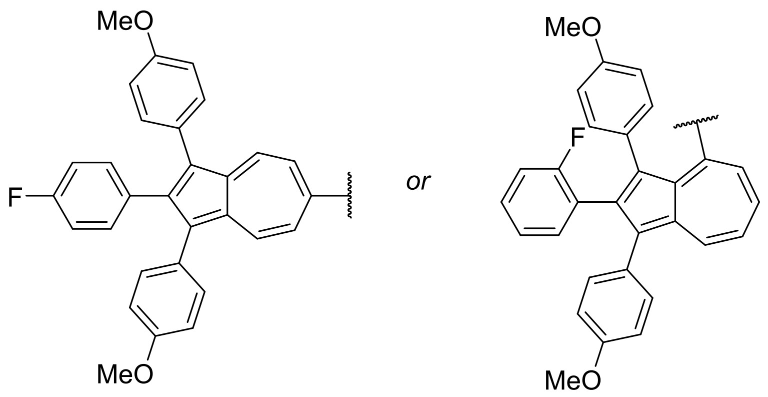

In a round bottom flask, the isonitrile gold(I) complex (1.00 eq.) was dissolved in reagent grade dichloromethane. Afterwards the appropriate amine (1.00 eq.) was added to the reaction mixture which was then stirred upon full consumption of the starting material. The crude reaction mixture was purified by flash column chromatography to obtain the corresponding azulene-tethered carbene gold(I) complex.

2.2 General Procedure for the One-Pot Synthesis of Azulene-tethered Carbene Gold(I) Complexes from [AuCl(DMS)] (GP2)

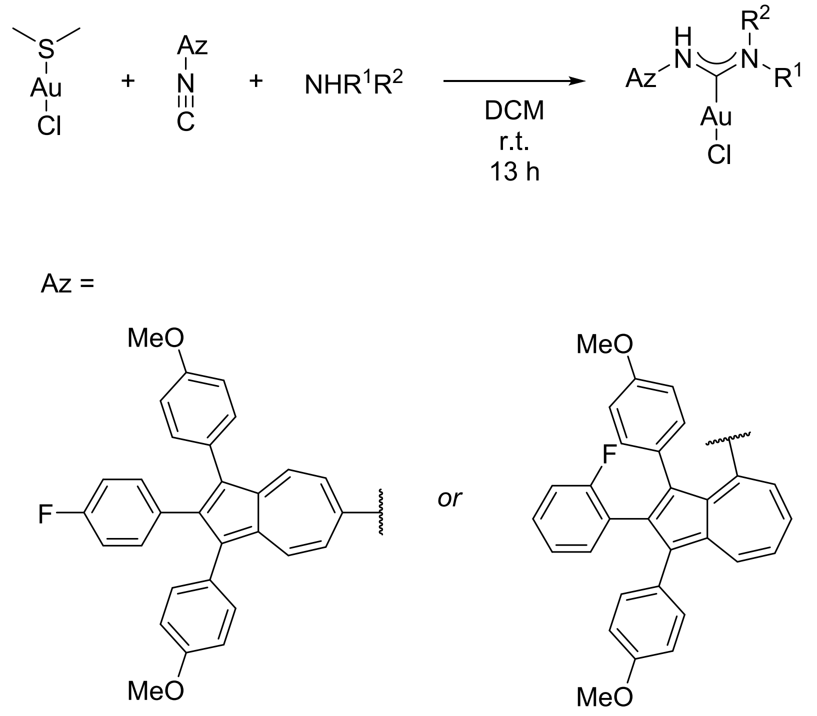

In a round bottom flask, [AuCl(DMS)] (1.00 eq.) was dissolved in reagent grade dichloromethane. Thereafter, the appropriate isonitrile (1.00 eq.) was added. After stirring the reaction mixture for one hour at room temperature, the amine (1.05 eq.) was added and the reaction was again stirred at room temperature upon completion. The crude reaction mixture was purified by flash column chromatography to obtain the corresponding azulene-tethered carbene gold(I) complex.

Synthesis of 2,6-dibenzhydryl-4-methylaniline

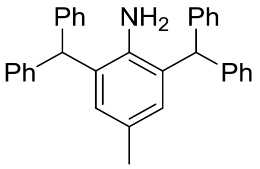

According to a literature procedure,[1] *p*-toluidine (8.72 g, 81.4 mmol, 1.00 eq.), diphenylmethanol (30.0 g, 163 mmol, 2.00 eq.) were melted together at 80 °C. A solution of zinc chloride (5.55 g, 40.7 mmol, 50.0 mol%.) and hydrochloric acid (36% in H_2O, 6.80 mL, 79.1 mmol, 97.0 mol%) was then added dropwise to the eutectic melt. Afterwards, the reaction mixture was stirred at 160 °C for 4 hours. After cooling to room temperature, the solidified reaction medium was taken up in dichloromethane (4 x 50 mL). The organic solution was washed subsequently with saturated solutions of ammonium chloride (200 mL) and brine (200 mL). The combined organic layers were dried over K_2CO_3. Evaporation of the solvent yielded the title compound as an off-white solid (35.3 g, 80.4 mmol,98%).

The obtained ^{1}H NMR spectrum matched the reported literature data.[1]

^{1}H NMR ($CDCl_3$, 300 MHz): δ [ppm] = 7.32 – 7.27 (m, 8H, 8x$C_{Ar}\underline{H}$), 7.25 – 7.21 (m, 4H, 4x$C_{Ar}\underline{H}$), 7.13 – 7.08 (m, 8H, 8x$C_{Ar}\underline{H}$), 6.12 (s, 2H, 2x$C_{Ar}\underline{H}$), 5.60 (s, 2H, -N$\underline{H_2}$), 2.05 (s, 3H, -C$\underline{H_3}$).

Synthesis of *N,N'*-bis(2,6-dibenzhydryl-4-methylphenyl)ethane-1,2-diimine

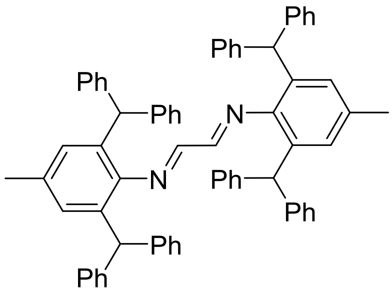

According to a literature procedure, 2,6-dibenzhydryl-4-methylaniline (17.0 g, 38.7 mmol, 2.00 eq.) was dissolved in acetonitrile (400 mL). Afterwards glyoxal (40% in H_2O, 2.81 g, 19.3 mmol, 1.00 eq.) and four drops of formic acid were added to the solution. The reaction mixture stirred for one week at 60 °C, at that the crude product participated which was filtered off and was washed subsequently with acetonitrile (3 x 50 mL) and concentrated *in vacuo*. The title compound was yielded as a yellow solid (11.0 g, 12.2 mmol, 63%).

The obtained ^{1}H NMR spectrum matched the reported literature data. [1]

^{1}H NMR ($CDCl_3$, 300 MHz, 295 K) δ [ppm] = 7.25 – 6.95 (m, 44H, 44x$C_{Ar}\underline{H}$), 6.60 – 6.47 (m, 4H, 4x$C_{Ar}\underline{H}$), 2.11 (s, 6H, 2x-$C\underline{H_3}$).

Synthesis of 1,3-bis(2,6-bis(diphenylmethyl)-4-methylphenyl)-imidazolium chloride (IPr* HCl)

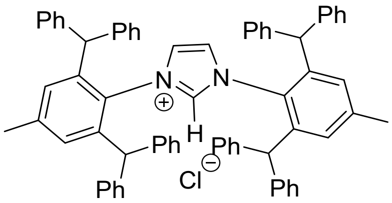

According to a literature procedure, *N,N'*-bis(2,6-dibenzhydryl-4-methylphenyl)ethane-1,2-diimine (7.00 g, 7.78 mmol, 1.00 eq.), $ZnCl_2$ (1.06 g, 7.77 mmol, 1.00 eq.) and paraformaldehyde (233 mg, 7.77 mmol, 1.00 eq.) were dissolved in chloroform (70.0 mL). The reaction mixture was stirred for 1 h at 60°C, afterwards the reaction mixture was concentrated *in vacuo*. The residue was taken up in ethyl acetate (70.0 mL), then TMSCl (844 mg, 7.77 mmol, 1.00 eq, dissolved in 5.00 mL EtOAc) was added to the solution. The reaction mixture was stirred overnight at 80 °C. Subsequently the solution was washed with water (2 x 50 mL) and brine (50 mL). The combined organic layers were dried over anhydrous $MgSO_4$ and then evaporated onto Celite ®. Purification of the crude product was obtained by flash column chromatography (Silica, dichloromethane → dichloromethane : MeOH = 95 :5). The title compound was obtained as an off-white solid (4.17 g, 4.40 mmol, 59%).

The obtained ^{1}H NMR spectrum matched the reported literature data.[1]

^{1}H NMR ($CDCl_3$, 300 MHz,295 K) δ [ppm] = 12.89 (s, 1H, $C_{Carbene}\underline{H}$), 7.26 – 7.10 (m, 32H, 32x$C_{Ar}\underline{H}$), 6.81 – 6.76 (m, 12H, 12x$C_{Ar}\underline{H}$), 5.50 (s, 2H, 2x$C_{Ar}\underline{H}$), 5.28 (s, 4H, 4x$C_{Ar}\underline{H}$), 2.19 (s, 6H, 2x-$C\underline{H_3}$).

Synthesis of (1,3-bis(2,6-dibenzhydryl-4-methylphenyl)-imidazol-2-ylidene)gold(I) chloride (IPr*AuCl)

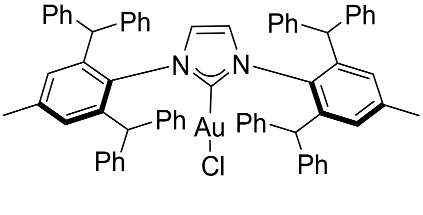

According to a literature procedure, [AuCl(DMS)] (200 mg, 679 µmol, 1.00 eq.), IPr* HCl (709 mg, 747 µmol, 1.10 eq.) and K_2CO_3 (141 mg, 1.02 mmol, 1.50 eq.) were suspended in acetone (15.0 mL) and stirred at room temperature overnight. The reaction mixture was concentrated *in*

vacuo and then evaporated onto Celite ®. Purification of the crude product was obtained by flash column chromatography (Silica, dichloromethane) to obtain the title compound as a colorless solid (651 mg, 569 µmol, 84%).

The obtained ^{1}H NMR spectrum matched the reported literature data.[2]

^{1}H NMR (CD_2Cl_2, 300 MHz, 295 K) δ [ppm] = 7.27 – 7.01 (m, 32H), 6.98 – 6.82 (m, 12H), 5.85 (s, 2H), 5.27 (s, 4H), 2.17 (s, 6H)

Synthesis of [(IPr*)Au(NCMe)]SbF$_6$

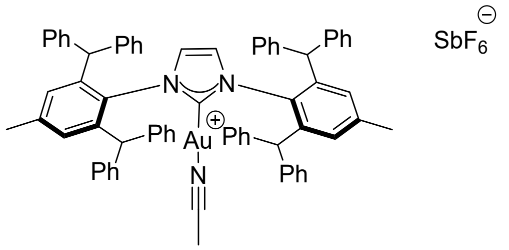

Based on a literature procedure, [AuCl(IPr*)] (170 mg, 148 µmol, 1.00 eq.) was dissolved in dry acetonitrile (3.00 mL), afterwards $AgSbF_6$ (53.5 mg, 156 µmol, 1.05 eq.) was added to the solution. The reaction mixture was stirred for 30 min at room temperature, then all the solvent was removed *in vacuo*. The residue was taken up in dichloromethane and filtered with dichloromethane (4 mL) through a plug of silica. Concentration *in vacuo* yielded the title compound as a colorless solid (199 mg, 143 µmol, 97%).

The obtained ^{1}H NMR spectrum matched the reported literature data.[3]

^{1}H NMR (CD_2Cl_2, 300 MHz, 295 K) δ [ppm] = 7.30 – 7.15 (m, 24H, 24x$C_{Ar}\underline{H}$), 6.95 – 6.86 (m, 20H, 20x$C_{Ar}\underline{H}$), 6.11 (s, 2H, 2x$C_{Ar}\underline{H}$), 5.08 (s, 4H, 4x$C_{Ar}\underline{H}$), 2.44 (s, 3H, NC$C\underline{H}_3$), 2.26 (s, 6H, 2x-$C\underline{H}_3$).

Synthesis of 1-fluoro-2-((4-methoxyphenyl)ethynyl)benzene (1a)

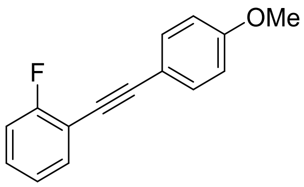

In a flame dried Schlenk-flask, 1-fluoro-2-iodobenzene (5.00 g, 22.5 mmol, 1.00 eq.), 1-ethynyl-4-methoxybenzene (3.57 g, 27.0 mmol, 1.20 eq.), bis(triphenylphosphine)palladium chloride (395 mg, 563 µmol, 2.50 mol%) and copper iodide (214 mg, 1.13 mmol, 5.00 mol%) were stirred at 60 °C in degassed triethylamine overnight. Afterwards, the reaction mixture was

purified by flash column chromatography (Silica, PE : EA = 30 :1) to yield the title compound as a colourless solid (4.54 g, 20.1 mmol, 89%).

The obtained ^{1}H NMR spectrum matched the reported literature data.[4]

^{1}H NMR ($CDCl_3$, 300 MHz, 295 K) δ [ppm] = 7.54 – 7.48 (m, 3H, 3xC_{Ar}H), 7.34 – 7.26 (m, 1H, C_{Ar}H), 7.16 – 7.06 (m, 2H, 2xC_{Ar}H), 6.93- 6.87 (m, 2H, 2xC_{Ar}H), 3.85 (s, 3H, -OC$\underline{H}_3$).

Synthesis of 1-fluoro-4-((4-methoxyphenyl)ethynyl)benzene (1b)

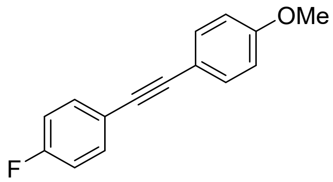

In a flame dried Schlenk-flask, 1-iodo-4-methoxybenzene (3.25 g, 13.9 mmol, 1.0 eq.), 1-ethynyl-4-fluorobenzene (2.00 g, 16.7 mmol, 1.2 eq.), bis(triphenylphosphine)palladium chloride (243 mg, 347 µmol, 2.5 mol%) and copper iodide (132 mg, 643 µmol, 5.0 mol%) were stirred at 80 °C in degassed triethylamine over night. Afterwards, the reaction mixture was purified by flash column chromatography (Silica, PE : EA = 100 : 1) to yield the title compound as a colourless solid (1.41 g, 72%).

The obtained ^{1}H NMR spectrum matched the reported literature data.[4]

^{1}H NMR ($CDCl_3$, 300 MHz, 295 K): δ [ppm] = 7.54 – 7.43 (m, 4H, 4x$C_{Ar}\underline{H}$), 7.10 – 6.99 (m, 2H, 2x$C_{Ar}\underline{H}$), 6.93 – 6.85 (m, 2H, 2x$C_{Ar}\underline{H}$), 3.84 (s, 3H, -OC$\underline{H}_3$).

Synthesis of 4-fluoro-2-(2-fluorophenyl)-1,3-bis(4-methoxyphenyl)azulene (2a)

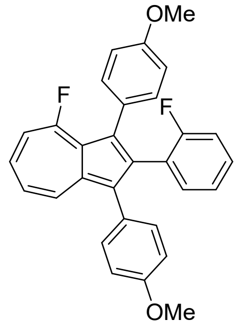

In a round bottom flask, 1-fluoro-2-((4-methoxyphenyl)ethynyl)benzene (**1a**) (3.50 g, 15.5 mmol, 2.0 eq.) and [(IPr*)Au(NCMe)]SbF_6 (536 mg, 387 µmol, 5.0 mol%) were stirred in toluene at 100 °C overnight. Afterwards, the reaction mixture was purified by flash column chromatography (Silica, petroleum ether : ethyl acetate = 50 : 1) to yield the title compound as a blue solid (2.87 g, 6.34 mmol, 82%).

The obtained ^{1}H NMR spectrum matched the reported literature data.[4]

^{1}H{^{19}F} NMR (300 MHz, $CDCl_3$, 300 K) δ [ppm] = 8.26 (d, J = 9.9 Hz, 1H, $C_{Ar}H$), 7.42 (td, J = 10.28 Hz, 4.06 Hz, 1H, $C_{Ar}H$), 7.14 – 7.09 (m, 4H, 4x$C_{Ar}H$), 7.01 – 6.95 (m, 1H, $C_{Ar}H$), 6.92 (dd, J = 11.1 Hz, 3.4 Hz, 1H, $C_{Ar}H$), 6.88 – 6.82 (m, 3H, 3x$C_{Ar}H$), 6.82 – 6.78 (m, 3H, 3x$C_{Ar}H$), 6.71 (d, J = 8.67 Hz, 2H, 2x$C_{Ar}H$), 3.74 (s, 3H, -OC$\underline{H_3}$), 3.73 (s, 3H, -OC$\underline{H_3}$).

Synthesis of 6-fluoro-2-(4-fluorophenyl)-1,3-bis(4-methoxyphenyl)azulene (2b)

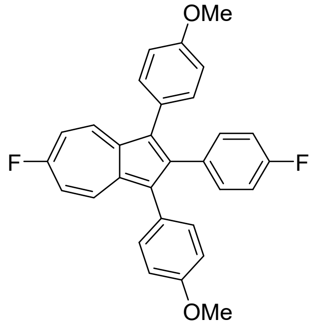

In a round bottom flask, 1-fluoro-4-((4-methoxyphenyl)ethynyl)benzene (**1b**) (3.70 g, 16.4 mmol, 2.00 eq.) and [(IPr*)Au(NCMe)]SbF_6 (567 mg, 408 µmol, 5.00 mol%) were stirred at 100 °C in toluene overnight. Afterwards, the reaction mixture was purified by flash column chromatography (Silica, PE : EA = 100 : 1) to yield the title compound as a blue solid (2.30 g, 62%).

The obtained ^{1}H NMR spectrum matched the reported literature data.[4]

^{1}H{^{19}F} NMR (300 MHz, $CDCl_3$, 300 K) δ [ppm] = 8.30 – 8.15 (m, 2H, 2x$C_{Ar}\underline{H}$), 7.24 – 7.11 (m, 4H, 4x$C_{Ar}\underline{H}$), 7.10 – 6.78 (m, 10H, 10x$C_{Ar}\underline{H}$), 3.86 (s, 6H, 2x-OC$\underline{H_3}$).

Synthesis of 2-(2-fluorophenyl)-1,3-bis(4-methoxyphenyl)azulen-4-amine (4a)

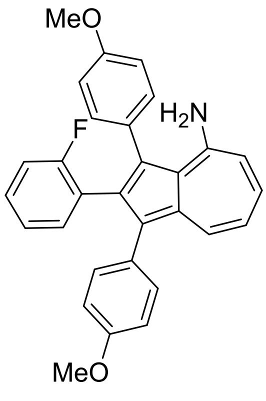

In a pressure vial, 4-fluoro-2-(2-fluorophenyl)-1,3-bis(4-methoxyphenyl)azulene (**2a**) (2.87 g, 6.34 mmol, 1.00 eq.) and ammonia (**3**) (21.58 g, 316 mmol, 50 eq., 25.0% in aq) were stirred

in acetonitrile at 100 °C for 12 h. Afterwards the solution was washed with brine (50 ml), the aqueous layer was extracted with dichloromethane (3x 30 mL). The combined organic layers were dried over anhydrous magnesium sulphate, filtered and concentrated *in vacuo*. Purification by flash column chromatography (Silica, PE : EA = 5 : 1) yielded the desired compound as a red (2.25 g, 5.01 mmol, 79%).

MP= 127 – 132 °C; **^{1}H{^{19}F} NMR** (500 MHz, C_6D_6, 295 K) δ [ppm] = 8.14 (d, *J* = 10.0 Hz, 1H; $C_{Ar}\underline{H}$), 7.18 (d, *J* = 8.8 Hz, 3H, 3x $C_{Ar}\underline{H}$), 7.04 – 6.99 (m, 1H, $C_{Ar}\underline{H}$), 6.95 (s, 1H, $C_{Ar}\underline{H}$), 6.77 (ddd, *J* = 10.9, 9.1, 1.3 Hz, 1H, $C_{Ar}\underline{H}$), 6.53 (d, *J* = 8.9 Hz, 2H, 2x$C_{Ar}\underline{H}$), 6.50 – 6.47 (m, 2H, 2x$C_{Ar}\underline{H}$), 6.47 – 6.37 (m, 3H, 3x$C_{Ar}\underline{H}$), 6.26 (t, *J* = 9.5 Hz, 1H, $C_{Ar}\underline{H}$), 5.57 (d, *J* = 11.1 Hz, 1H, $C_{Ar}\underline{H}$), 4.35 (s, 2H, -N$\underline{H_2}$), 3.02 (s, 3H, -OC$\underline{H_3}$), 2.96 (s, 3H, -OC$\underline{H_3}$); **^{13}C{^{19}F, ^{1}H} NMR** (126 MHz, C_6D_6, 295 K) δ [ppm] = 161.06 (s, 1C, $\underline{C_{Ar}}$), 158.98 (s, 1C, $\underline{C_{Ar}}$), 158.66 (s, 1C, $\underline{C_{Ar}}$), 154.61 (s, 1C, $\underline{C_{Ar}}$), 137.96 (s, 1C, $\underline{C_{Ar}}$), 137.14 (d, 1C, $\underline{C_{Ar}}\underline{H}$), 136.97 (d, 1C, $\underline{C_{Ar}}\underline{H}$), 134.56 (s, 1C, $\underline{C_{Ar}}$), 133.63 (d, 1C, $\underline{C_{Ar}}\underline{H}$), 133.27 (d, 1C, $\underline{C_{Ar}}\underline{H}$), 132.99 (d, 1C, $\underline{C_{Ar}}\underline{H}$), 132.83 (d, 1C, $\underline{C_{Ar}}\underline{H}$), 131.88 (s, 1C, $\underline{C_{Ar}}$), 131.17 (s, 1C, $\underline{C_{Ar}}$), 130.07 (d, 2C, 2x$\underline{C_{Ar}}$H), 128.59 (d, 1C, $\underline{C_{Ar}}\underline{H}$), 128.44 (d, 1C, $\underline{C_{Ar}}\underline{H}$), 127.46 (s, 1C, $\underline{C_{Ar}}$), 126.79 (s, 1C, $\underline{C_{Ar}}$), 123.40 (s, 1C, $\underline{C_{Ar}}$), 117.55 (s, 1C, $\underline{C_{Ar}}$), 116.57 (d, 1C, $\underline{C_{Ar}}\underline{H}$), 116.08 (d, 1C, $\underline{C_{Ar}}\underline{H}$), 115.38 (d, 1C, $\underline{C_{Ar}}$H), 113.88 (d, 2C, 2x$\underline{C_{Ar}}$H), 113.37 (d, 1C, $\underline{C_{Ar}}\underline{H}$), 54.61 (q, 1C, -O$\underline{C}H_3$), 54.58 (q, 1C, -O$\underline{C}H_3$); **^{19}F{^{1}H} NMR** (471 MHz, C_6D_6, 295 K) δ [ppm] = -111.74 (s, 1F, $C_{Ar}F$); **IR** (ATR) $\tilde{\nu}$ [cm^{-1}] = 3478 (m), 3452 (m), 3368 (m), 3360 (m), 3228 (m), 3001 (w), 2931 (w), 2836 (w), 1635 (m), 1606 (m), 1576 (m), 1546 (s), 1486 (m), 1430 (m), 1386 (w), 1302 (m), 1280 (m), 1232 (s), 1175 (s), 1155 (s), 1106 (s), 1038 (s), 949 (w), 889 (w), 840 (w), 811 (w), 774 (w), 726 (w), 624 (w); **HR-MS** (MALDI): $C_{30}H_{24}FNO_2$ $[M]^+$ calcd.: 449.1791, found.: 449.1795, Δm/z = 0.891 ppm; **UV/VIS** (CH_2Cl_2) λ_{max} (ε) [nm ($l*mol^{-1}*cm^{-1}$)] = 265 (39517), 313 (32571), 333 (28472), 398 (3093), 538 (1443).

Synthesis of 2-(4-fluorophenyl)-1,3-bis(4-methoxyphenyl)azulen-6-amine (4b)

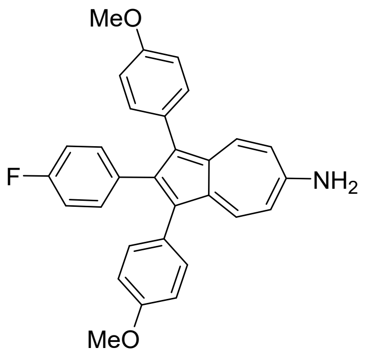

In a pressure vial, 6-fluoro-2-(4-fluorophenyl)-1,3-bis(4-methoxyphenyl)azulene (**2b**) (4.00 g, 8.84 mmol, 1.00 eq.) and ammonia (**3**) (30.1 g, 442 mmol, 50.0 eq., 25.0% in aq.) were stirred

in acetonitrile at 100 °C for 12 h. Afterwards the solution was washed with brine (50 ml), the aqueous layer was extracted with dichloromethane (3x 30 mL). The combined organic layers were dried over anhydrous magnesium sulphate, filtered and concentrated *in vacuo*. Purification by flash column chromatography (Silica, PE : EA = 3 : 1) yielded the desired compound as a red solid (3.07 g, 77%).

MP = 200 – 205 °C; **^{1}H{^{19}F} NMR** (600 MHz, C_6D_6, 295 K) δ [ppm] = 8.17 (d, *J* = 11.0 Hz, 2H, 2x-$C_{Ar}\underline{H}$), 7.33 (d, *J* = 8.8 Hz, 4H, 4x-$C_{Ar}\underline{H}$), 7.21 (dd, *J* = 8.8, 5.5 Hz, 2H, 2x-$C_{Ar}\underline{H}$), 6.68 (t, *J* = 8.8 Hz, 4H, 4x-$C_{Ar}\underline{H}$), 5.77 (d, *J* = 11.0 Hz, 2H, 2x-$C_{Ar}\underline{H}$), 3.33 (s, 6H, 2x-$OC\underline{H_3}$), 3.21 (s, 2H, -$N\underline{H_2}$); **^{13}C{^{19}F, ^{1}H} NMR** (151 MHz, C_6D_6, 295 K) δ [ppm] = 162.64 (s, 1C, -$\underline{C_{Ar}}$), 161.02 (s, 1C, -$\underline{C_{Ar}}$), 158.73 (d, 2C, 2x-$\underline{C_{Ar}}$H), 157.01 (d, 2C, 2x-$\underline{C_{Ar}}$H), 140.22 (s, 1C, -$\underline{C_{Ar}}$), 136.98 (s, 2C, 2x-$\underline{C_{Ar}}$), 134.41 (s, 1C, -$\underline{C_{Ar}}$), 134.39 (s, 1C, -$\underline{C_{Ar}}$), 133.42 (d, 1C, -$\underline{C_{Ar}}$H), 133.37 (d, 1C, -$\underline{C_{Ar}}$H), 132.97 (d, 2C, 2x-$\underline{C_{Ar}}$H), 131.92 (s, 1C, -$\underline{C_{Ar}}$), 130.38 (s, 1C, -$\underline{C_{Ar}}$), 129.90 (s, 1C, -$\underline{C_{Ar}}$), 128.59 (s, 1C, -$\underline{C_{Ar}}$), 128.35 (s, 1C, -$\underline{C_{Ar}}$), 115.05 (d, 2C, 2x-$\underline{C_{Ar}}$H), 114.91 (d, 2C, 2x-$\underline{C_{Ar}}$H), 114.13 (d, 2C, 2x-$\underline{C_{Ar}}$H), 109.96 (d, 2C, 2x-$\underline{C_{Ar}}$H), 54.76 (q, 2C, 2x-$O\underline{C}H_3$); **^{19}F{^{1}H} NMR** (283 MHz, C_6D_6, 295 K) δ [ppm] = -116.74 (s, 1F, $C_{Ar}\underline{F}$); **IR** (ATR) $\tilde{\nu}$ [cm^{-1}] = 3361 (m), 3219 (w), 2960 (m), 2931 (m), 2874 (w), 2836 (w), 1581 (s), 1516 (s), 1463 (m), 1434 (m), 1407 (m), 1391 (m), 1287 (m), 1242 (s), 1221 (s), 1175 (s), 1156 (m), 1108 8w), 1033 (m), 898 (w), 833 (m), 794 (m), 751 (w), 701 (w), 634 (w); **HR-MS** (EI+): $C_{30}H_{24}FNO_2$ $[M]^+$ calcd.: 449.17911, found.: 449.17946, Δm/z = 0.779 ppm; **UV/VIS** (CH_2Cl_2) λ_{max} (ε) [nm (l*mol^{-1}*cm^{-1})] = 270 (1914), 343 (7614), 388 (806), 416 (31188), 479 (14373).

Synthesis of acetic formic anhydride (5)

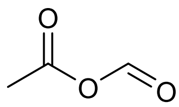

According to a literature procedure, sodium formate (22.0 g, 324 mmol, 1.20 eq.) was suspended in absolute diethylether (20.0 mL). Then acetic chloride (21.2 g, 270 mmol, 1.00 eq.) was added to dropwise. The reaction mixture was stirred over night at room temperature, afterwards the solid was filtered off. The crude product was concentrated *in vacuo* and was used without further purification. The reaction yielded the title compound as a colorless fluid (18.5 g, 78%).

The obtained ^{1}H NMR spectrum matched the reported literature data.[5]

^{1}H NMR ($CDCl_3$, 300 MHz): δ [ppm] = 9.03 (s, 1H), 2.21 (s, 3H).

Synthesis of N-(2-(2-fluorophenyl)-1,3-bis(4-methoxyphenyl)azulen-4-yl)formamide (6a)

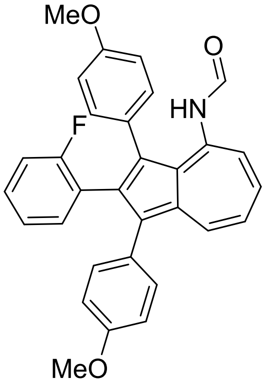

In a round bottom flask, 2-(2-fluorophenyl)-1,3-bis(4-methoxyphenyl)azulen-4-amine (**4a**) (2.10 g, 4.67 mmol, 1.00 eq.) was dissolved in anhydrous dichloromethane, afterwards acetic formic anhydride (**5**) (411 mg, 4.67 mmol, 1.00 eq.) was added dropwise to the solution. The reaction was stirred at room temperature for 1 h. Afterwards, the reaction mixture was quenched by the addition of aqueous sodium carbonate and the aqueous layer was extracted with dichloromethane (3x50 mL). The combined organic layers were dried over anhydrous magnesium sulphate, filtered and concentrated *in vacuo*. Purification by flash column chromatography (Silica, dichloromethane : petroleum ether = 1:1) yielded the desired product as a green solid (1.67 g, 3.50 mmol, 75%).

MP = 173 – 178 °C; **$^{1}H\{^{19}F\}$ NMR** (700 MHz, $C_2D_2Cl_4$, 400 K) δ [ppm] = 8.42 (s, 1H, -NHC$\underline{H}$O), 8.34 (d, *J* = 9.5 Hz, 1H, $C_{Ar}\underline{H}$), 8.08 – 8.05 (m, 1H, -N$\underline{H}$CHO), 7.70 (s, 1H, $C_{Ar}\underline{H}$), 7.58 (t, *J* = 10.3 Hz, 1H, $C_{Ar}\underline{H}$), 7.29 (d, *J* = 8.4 Hz, 2H, 2x$C_{Ar}\underline{H}$), 7.25 (d, *J* = 8.6 Hz, 2H, $C_{Ar}\underline{H}$), 7.15 (m, 1H, $C_{Ar}\underline{H}$), 7.07 (t, *J* = 9.6 Hz, 1H, $C_{Ar}\underline{H}$), 7.01 – 6.97 (m, 1H, $C_{Ar}\underline{H}$), 6.95 – 6.90 (m, 5H, 5x$C_{Ar}\underline{H}$), 6.88 (t, *J* = 9.0 Hz, 1H, $C_{Ar}\underline{H}$), 3.87 (s, 6H, 2x-C$\underline{H_3}$); **$^{13}C\{^{1}H\}$ NMR** (176 MHz, $C_2D_2Cl_4$, 400 K) δ [ppm] =160.70 (s, 1C, $\underline{C_{Ar}}$), 159.74 (d, 1C, -NH$\underline{C}$HO), 159.29 (s, 1C, $\underline{C_{Ar}}$), 158.56 (s, 1C, $\underline{C_{Ar}}$), 143.24 (s, 1C, $\underline{C_{Ar}}$), 142.75 (s, 1C, $\underline{C_{Ar}}$), 138.09 (s, 1C, $\underline{C_{Ar}}$), 136.82 (d, 1C, $\underline{C_{Ar}}$H), 136.45 (d, 1C, $\underline{C_{Ar}}$H), 132.40 (d, 1C, $\underline{C_{Ar}}$H, d, *J* = 3.7 Hz), 132.36 (d, 1C, $\underline{C_{Ar}}$H), 131.99 (d, 1C, $\underline{C_{Ar}}$H), 131.74 (s, 1C, $\underline{C_{Ar}}$), 129.14 (s, 1C, $\underline{C_{Ar}}$), 128.49 (d, 1C, $\underline{C_{Ar}}$H, d, *J* = 7.8 Hz), 128.27 (s, 1C, $\underline{C_{Ar}}$), 127.21 (s, 1C, $\underline{C_{Ar}}$), 124.80 (s, 1C, $\underline{C_{Ar}}$, d, *J* = 16.5 Hz), 123.46 (s, 1C, $\underline{C_{Ar}}$), 122.79 (d, 1C, $\underline{C_{Ar}}$H, d, *J* = 3.4 Hz), 121.29 (d, 1C, $\underline{C_{Ar}}$H), 116.69 (d, 1C, $\underline{C_{Ar}}$H), 114.76 (d, 1C, $\underline{C_{Ar}}$H), 114.64 (d, 1C, $\underline{C_{Ar}}$H, *J* = 22.3 Hz), 113.79(d, 1C, $\underline{C_{Ar}}$H), 113.67(d, 1C, $\underline{C_{Ar}}$H), 55.27 (q, 1C, -OCH_3), 55.15 (q, 1C, -OCH_3); **$^{19}F\{^{1}H\}$ NMR** (471 MHz, C_6D_6, 295 K) δ [ppm] = -111.83 (s, 1F, $C_{Ar}\underline{F}$); **IR** (ATR) $\tilde{\nu}$ [cm^{-1}] = 3354 (m), 2933 (w), 2836 (w), 1691 (s), 1607 (m), 1577 (m), 1547 (s), 1454 (m), 1263 (s), 1242 (s), 1177 (s), 1107 (s), 1030 (s), 949 (w), 933 (w), 833 (w), 752 (w), 736 (w), 667 (w); **HR-MS** (EI+): $C_{31}H_{24}FNO_3$ $[M]^+$ calcd.:

477.17402, found.: 477.17237, Δm/z = 3.457 ppm ; **UV/VIS** (CH_2Cl_2) λ_{max} (ε) [nm (l*mol^{-1}*cm^{-1})] = 270 (101373), 319 (89643), 369 (91319), 590 (1198), 638 (184).

Synthesis of N-(2-(4-fluorophenyl)-1,3-bis(4-methoxyphenyl)azulen-6-yl)formamide (6b)

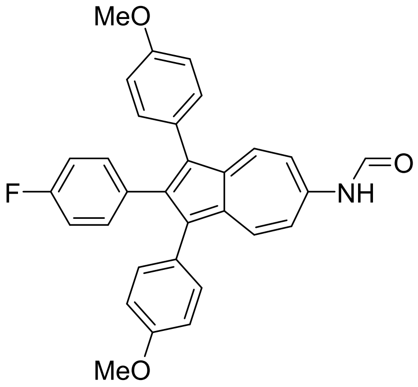

In a round bottom flask, 2-(4-fluorophenyl)-1,3-bis(4-methoxyphenyl)azulen-6-amine (**4b**) (6.00 g, 13.4 mmol, 1.00 eq.) was dissolved in anhydrous dichloromethane, afterwards acetic formic anhydride (**5**) (1.18 g, 13.4 mmol, 1.00 eq.) was added dropwise to the solution. The reaction was stirred at room temperature for 1 h. Afterwards, the reaction mixture was quenched by the addition of aqueous sodium carbonate and the aqueous layer was extracted with dichloromethane (3x50 mL). The combined organic layers were dried over anhydrous magnesium sulphate, filtered and concentrated *in vacuo*. Purification by flash column chromatography (Silica, dichloromethane : petroleum ether = 1:1) yielded the desired product as a green solid (5.70 g, 11.9 mmol, 89%).

MP = 231 – 236 °C; **^{1}H{^{19}F} NMR** (700 MHz, $C_2D_2Cl_4$, 295 K) δ [ppm] = 8.88 (d, *J* = 10.8 Hz, 1H, -NHCHO, major rotamer), 8.43 (s, 1H, -NHCHO, minor rotamer), 8.19 (t, *J* = 10.6 Hz, 1H, 2xC_{Ar}H), 7.98 (d, *J* = 10.8 Hz, 1H, -NHCHO, major rotamer), 7.40 (s, 1H, -NHCHO, minor rotamer), 7.35 (d, *J* = 10.8 Hz, 1H, C_{Ar}H), 7.17 (d, *J* = 8.7 Hz, 4H, 4xC_{Ar}H), 7.00 – 6.96 (m, 2H, 2xC_{Ar}H), 6.92 (d, *J* = 8.6 Hz, 4H, 4xC_{Ar}H), 6.86 (t, *J* = 8.8 Hz, 2H, 2xC_{Ar}H), 6.75 (d, *J* = 11.0 Hz, 1H, C_{Ar}H), 3.85 (s, 6H, 2x-OCH_3); **^{13}C{^{1}H} NMR** (151 MHz, CD_2Cl_2, 295 K) δ [ppm] = (176 MHz, $C_2D_2Cl_4$, 400 K) δ [ppm] = 162.16 (d, 1C, -NHCHO, major rotamer), 162.09 (s, 1C, C_{Ar}), 160.67 (s, 1C, C_{Ar}d, *J* = 7.8 Hz), 159.27 (d, 1C, -NHCHO, minor rotamer), 158.03 (s, 1C, C_{Ar}), 157.91 (s, 1C, C_{Ar}), 145.30 (s, 1C, C_{Ar}), 145.17 (s, 1C, C_{Ar}), 144.85 (s, 1C, C_{Ar}), 144.16 (s, 1C, C_{Ar}), 135.35 (d, 1C, C_{Ar}H), 135.16 (d, 1C, C_{Ar}H), 134.55 (s, 1C, C_{Ar}), 134.26 (s, 1C, C_{Ar}), 132.72 (d, 1C, C_{Ar}H, d, *J* = 7.4 Hz), 132.29 (d, 1C, C_{Ar}H), 132.26 (d, 2C, 2xC_{Ar}H), 132.14 (d, 1C, C_{Ar}H), 130.60 (d, 1C, C_{Ar}H), 129.76 (d, 1C, C_{Ar}H), 127.77 (s, 1C, C_{Ar}), 127.55 (d, 1C, C_{Ar}H), 120.18 (s, 1C, C_{Ar}), 115.28 (d, 1C, C_{Ar}H), 114.69 (d, 1C, C_{Ar}H, *J* = 9.8 Hz),

114.57 (d, 1C, C̲Ar̲H, d, J = 9.8 Hz), 113.65 (d, 1C, C̲Ar̲H), 113.58 (d, 1C, C̲Ar̲H), 113.16 (d, 1C, C̲Ar̲H), 55.24 (q, 2C, 2x-OC̲H$_3$); **^{19}F{^{1}H} NMR** (283 MHz, CD_2Cl_2, 295 K) δ [ppm] = -116.37 (s, 1F, C_{Ar}F̲); **IR** (ATR) $\tilde{\nu}$ [cm^{-1}] = 3006 (w), 2959(w), 2927 (w), 2853 (w), 1741 (w), 1684 (s), 1608 (w), 1572 (m), 1548 (m), 1519 (s), 1441 (m), 1407 (w), 1388 (w), 1288 (s), 1245 (s), 1221 (s), 1175 (m), 1156 (m), 1108 (w), 1093 (w), 1033 (m), 909 (w), 826 (m), 796 (m), 751 (w), 637 (w); **HR-MS** (EI+): $C_{31}H_{24}FNO_3$ [M]$^+$ calcd.: 477.17402, found.: 477.17208, Δm/z = 4.07 ppm.

Synthesis of 2-(2-fluorophenyl)-4-isocyano-1,3-bis(4-methoxyphenyl)azulene (10a)

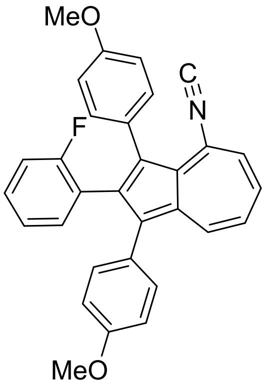

In a round bottom flask, *N*-(2-(4-fluorophenyl)-1,3-bis(4-methoxyphenyl)azulen-6-yl)formamide (**6a**) (1.16 g, 2.43 mmol, 1.00 eq.) was dissolved in reagent grade dichloromethane. Afterwards, triphenylphosphine (**7**) (1.91 g, 7.29 mmol, 3.00 eq.) and iodine (**8**) (1.85 g, 7.29 mmol, 3.00 eq.) were added to the solution. To the solution triethylamine (**9**) (1.47 g, 14.6 mmol, 6.00 eq.) was added dropwise at room temperature. After stirring for 3 h at room temperature, the reaction mixture was quenched by the addition of an aqueous sodium thiosulphate solution. The aqueous layer was extracted with dichloromethane (3x50 mL). The combined organic layers were then dried over anhydrous magnesium sulphate, filtered and concentrated *in vacuo*. Purification by flash column chromatography (Silica, PE : NEt_3 = 95 : 5) yielded the desired product as a green solid (825 mg, 1.80 mmol, 73%).

MP= 116 – 121 °C; **^{1}H{^{19}F} NMR** (500 MHz, C_6D_6, 295 K) δ [ppm] = 8.23 (dd, J = 9.5, 1.1 Hz, 1H, C_{Ar}H̲), 7.39 – 7.32 (m, 1H, C_{Ar}H̲), 7.25 (d, J = 8.6 Hz, 3H, 3xC_{Ar}H̲), 7.05 (dd, J = 7.5, 1.9 Hz, 1H, C_{Ar}H̲), 6.89 – 6.83 (m, 1H, C_{Ar}H̲), 6.82 – 6.74 (m, 4H, 4xC_{Ar}H̲), 6.73 – 6.69 (m, 1H, C_{Ar}H̲), 6.68 (s, 1H, C_{Ar}H̲), 6.66 (s, 1H, C_{Ar}H̲), 6.65 – 6.61 (m, 1H, C_{Ar}H̲), 6.53 (t, J = 9.6 Hz, 1H, C_{Ar}H̲), 3.25 (s, 3H, -OCH̲$_3$), 3.23 (s, 3H, -OCH̲$_3$); **^{13}C{^{19}F, ^{1}H} NMR** (101 MHz, C_6D_6, 295 K) δ [ppm] = 174.88 (s, 1C, C̲Ar̲), 161.74 (s, 1C, C̲Ar̲), 159.67 (s, 1C, C̲Ar̲), 159.38 (s, 1C, C̲Ar̲), 159.29 (s, 1C, C̲Ar̲), 147.53 (s, 1C, C̲Ar̲), 139.31 (s, 1C, C̲Ar̲), 136.88 (d, 1C, C̲Ar̲H), 135.73

(d, 1C, $\underline{C_{Ar}}$H), 133.11 (d, 1C, $\underline{C_{Ar}}$H), 132.82 (d, 1C, $\underline{C_{Ar}}$H), 132.78 (d, 1C, $\underline{C_{Ar}}$H), 132.62 (s, 1C, $\underline{C_{Ar}}$), 132.51 (d, 1C, $\underline{C_{Ar}}$H), 131.41 (s, 1C, $\underline{C_{Ar}}$), 130.92 (s, 1C, $\underline{C_{Ar}}$), 129.63 (d, 1C, $\underline{C_{Ar}}$H), 129.55 (d, 1C, $\underline{C_{Ar}}$H), 128.59 (d, 1C, $\underline{C_{Ar}}$H), 127.27 (s, 1C, $\underline{C_{Ar}}$), 125.26 (s, 1C, $\underline{C_{Ar}}$), 125.09 (s, 1C, $\underline{C_{Ar}}$), 124.31 (d, 1C, $\underline{C_{Ar}}$H), 123.92 (d, 1C, $\underline{C_{Ar}}$H), 123.66 (d, 1C, $\underline{C_{Ar}}$H), 115.72 (d, 1C, $\underline{C_{Ar}}$H), 115.50 (d, 1C, $\underline{C_{Ar}}$H), 114.21 (d, 1C, $\underline{C_{Ar}}$H), 113.23 (d, 1C, $\underline{C_{Ar}}$H), 54.72 (q, 1C, -O$\underline{C}H_3$), 54.57; **^{19}F{^{1}H} NMR** (471 MHz, C_6D_6, 295 K) δ [ppm] = -111.65 (s, 1F, $C_{Ar}\underline{F}$); **IR** (ATR) $\tilde{\nu}$ [cm^{-1}] = 2966 (w), 2925 (w), 2835 (w), 2535 (w), 2109 (s), 1949 (w), 1763 (w), 1688 (m), 1639 (m), 1588 (m), 1578 (m), 1544 (s), 1447 (m), 1407 (m), 1365 (w), 1265 (s), 1242 (s), 1226 (s), 1176 (s), 1127 (s), 1105 (s), 1039 (s), 980 (m), 938 (w), 880 (w), 828 (w), 802 (w), 761 (w), 739 (w), 721 (w), 693 (w), 619 (w); **HR-MS** (EI+): $C_{31}H_{22}FNO_2$ $[M]^+$ calcd.: 459.16346, found.: 459.16318, Δm/z = 0.610 ppm; **UV/VIS** (CH_2Cl_2) λ_{max} (ε) [nm (l*mol^{-1}*cm^{-1})] = 259 (49396), 294 (64330), 324 (90751), 412 (10339), 647 (574).

Synthesis of 2-(4-fluorophenyl)-6-isocyano-1,3-bis(4-methoxyphenyl)azulene (10b)

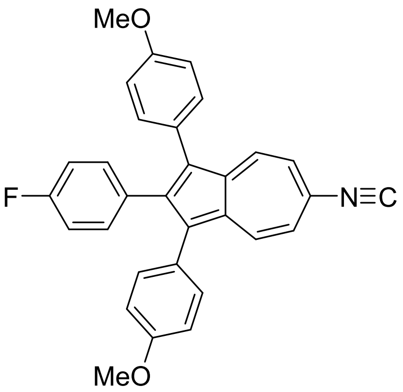

In a round bottom flask, *N*-(2-(4-fluorophenyl)-1,3-bis(4-methoxyphenyl)azulen-6-yl)formamide (**6b**) (5.70 g, 11.9 mmol, 1.00 eq.) was dissolved in reagent grade dichloromethane. Afterwards, triphenylphosphine (**7**) (9.39 g, 35.8 mmol, 3.00 eq.) and iodine (**8**) (9.09 g, 35.8 mmol, 3.00 eq.) were added to the solution. To the solution triethylamine (**9**) (7.25 g, 71.6 mmol, 6.00 eq.) was added dropwise at room temperature. After stirring for 3 h at room temperature, the reaction mixture was quenched by the addition of an aqueous sodium thiosulphate solution. The aqueous layer was extracted with dichloromethane (3x50 mL). The combined organic layers were then dried over anhydrous magnesium sulphate, filtered and concentrated *in vacuo*. Purification by flash column chromatography (Silica, PE : NEt_3 = 95 : 5) yielded the desired product as a green solid (5.20 g, 11.3 mmol, 95%).

MP = 127 – 132 °C; **^{1}H{^{19}F} NMR** (400 MHz, CD_2Cl_2, 295 K) δ [ppm] = 8.15 (d, *J* = 10.7 Hz, 2H, 2x$C_{Ar}\underline{H}$), 7.15 (d, *J* = 8.9 Hz, 4H, 4x$C_{Ar}\underline{H}$), 7.07 (d, *J* = 10.8 Hz, 2H, 2x$C_{Ar}\underline{H}$), 7.03 (dd, *J*

= 9.0, 5.5 Hz, 2H, 2x$C_{Ar}\underline{H}$), 6.93 (d, *J* = 8.8 Hz, 4H, 4x$C_{Ar}\underline{H}$), 6.86 (t, *J* = 8.9 Hz, 2H, 2x$C_{Ar}\underline{H}$), 5.33 (s, 3H, -OC$\underline{H}_3$), 3.84 (s, 3H, -OC$\underline{H}_3$); **^{13}C{^{19}F, ^{1}H} NMR** (101 MHz, CD_2Cl_2, 295 K) δ [ppm] = 165.35 (s, 1C, $\underline{C}_{Ar}$), 163.68 (s, 1C, $\underline{C}_{Ar}$), 161.22 (s, 1C, $\underline{C}_{Ar}$), 159.28 (s, 2C, 2x$\underline{C}_{Ar}$), 149.40 (s, 1C, $\underline{C}_{Ar}$), 137.28 (s, 1C, $\underline{C}_{Ar}$), 134.36 (d, 2C, 2x$\underline{C}_{Ar}$H), 133.37 (d, 2C, 2x$\underline{C}_{Ar}$H), 133.29 (d, 2C, 2x$\underline{C}_{Ar}$H), 132.79 (d, 2C, 2x$\underline{C}_{Ar}$H), 132.56 (s, 2C, 2x$\underline{C}_{Ar}$), 132.52 (s, 1C, $\underline{C}_{Ar}$), 131.76 (s, 1C, $\underline{C}_{Ar}$), 127.65 (s, 2C,2x $\underline{C}_{Ar}$), 121.40 (d, 2C, 2x$\underline{C}_{Ar}$H), 115.29 (d, 2C, 2x$\underline{C}_{Ar}$H), 115.08 (d, 2C, 2x$\underline{C}_{Ar}$H), 114.23 (d, 2C, 2x$\underline{C}_{Ar}$H), 55.65 (q, 2C, 2x-O$\underline{C}H_3$); **^{19}F{^{1}H} NMR** (283 MHz, CD_2Cl_2, 298 K) δ [ppm] = -115.27 (s, 1F, -$C_{Ar}\underline{F}$); **IR** (ATR) $\tilde{\nu}$ [cm^{-1}] = 2957 (w), 2929 (w), 2852 (w), 2836 (w), 2108 (s), 1727 (w), 1605 (m), 1573 (m), 1537 (w), 1517 (s), 1441 (s), 1406 (m), 1385 (w), 1287 (m), 1244 (s), 1174 (s), 1157 (m), 1132 (w), 1107 (w), 1031 (s), 951 (w), 834 (s), 793 (s), 753 (w), 657 (w); **HR-MS** (EI+): $C_{31}H_{22}FNO_2$ $[M]^+$ calcd.: 459.16346, found.: 459.16382, Δm/z = 0.784 ppm; **UV/VIS** (CH_2Cl_2) λ_{max} (ε) [nm ($l*mol^{-1}*cm^{-1}$)] = 259 (49396), 294 (64330), 324 (90751), 412 (10339), 647 (574).

Synthesis of 2-(2-fluorophenyl)-4-isocyano-1,3-bis(4-methoxyphenyl)azulene gold(I)chloride (12a)

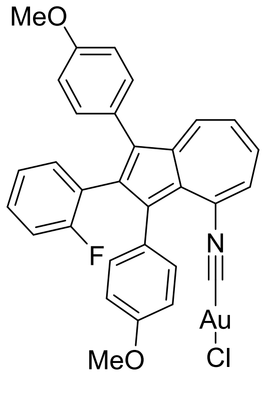

In a round bottom flask, [AuCl(DMS)] (**11**) (224 mg, 762 µmol, 1.00 eq.) and 2-(2-fluorophenyl)-4-isocyano-1,3-bis(4-methoxyphenyl)azulene (**10a**) (350 mg, 762 µmol, 1.00 eq.) were dissolved in reagent grade dichloromethane. The reaction mixture was stirred at room temperature for 1 h. Purification by flash column chromatography (Silica, dichloromethane : petroleum ether = 1 : 1) yielded the desired product as a green solid (427 mg, 617 µmol, 81%).

MP = 147 – 152 °C; **^{1}H{^{19}F} NMR** (700 MHz, C_6D_6, 295 K) δ [ppm] = 8.13 (d, *J* = 9.5 Hz, 1H), 7.20 (d, *J* = 8.3 Hz, 2H), 7.09 – 7.03 (m, 1H), 7.00 (t, *J* = 7.4 Hz, 1H), 6.96 – 6.92 (m,

1H), 6.85 – 6.81 (m, 1H), 6.75 (d, *J* = 7.8 Hz, 3H), 6.69 (t, *J* = 10.1 Hz, 2H), 6.62 (q, *J* = 8.4 Hz, 2H), 6.55 (t, *J* = 9.8 Hz, 1H), 6.23 (d, *J* = 10.2 Hz, 1H), 3.69 (s, 6H), 3.25 (s, 6H); **^{13}C{^{19}F, ^{1}H} NMR** (176 MHz, C_6D_6, 295 K) δ [ppm] = 160.97 (s, 1C), 160.72 (s, 1C), 159.58 (s, 1C), 148.41 (s, 1C), 145.83 (s, 1C), 139.20 (s, 1C), 137.01 (d, 1C), 135.20 (d, 1C), 133.04 (s, 1C), 132.67 (d, 1C), 132.41 (d, 1C), 131.24 (s, 1C), 130.07 (d, 1C), 130.03 (d, 1C), 128.59 (d, 1C), 128.35 (d, 1C), 127.31 (s, 1C), 127.26 (s, 1C), 127.07 (s, 1C), 125.33 (d, 1C), 124.43 (s, 1C), 124.34 (s, 1C), 123.81 (d, 1C), 123.79 (d, 1C), 123.02 (d, 1C), 115.80 (d, 1C), 115.68 (d, 1C), 114.32 (d, 1C), 114.13 (d, 1C), 55.27 (q, 1C), 54.74 (q, 1C); **^{19}F{^{1}H} NMR** (283 MHz, C_6D_6, 295 K) δ [ppm] = -111.67; **IR** (ATR) $\tilde{\nu}$ [cm^{-1}] = 2956, 2932, 2904, 2835, 2534, 2204, 1608, 1561, 1522, 1481, 1449, 1405, 1357, 1288, 1245, 1208, 1175, 1107, 1093, 1030, 941, 864, 830, 792, 760, 728, 701, 628; **HR-MS** (MALDI): $C_{31}H_{22}Au^{35}ClFNO_2$ $[M]^+$ calcd.: 691.0989, found.: 691.1006, Δm/z = 2.460 ppm; **UV/VIS** (CH_2Cl_2) λ_{max} (ε) [nm (l*mol^{-1}*cm^{-1})] = 269 (69771), 329 (61698), 664 (6285), 711 (1649), 800 (254).

Synthesis of 2-(4-fluorophenyl)-6-isocyano-1,3-bis(4-methoxyphenyl)azulene gold(I)chloride (12b)

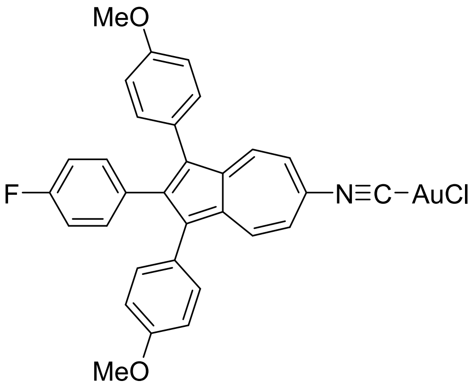

In a round bottom flask, [AuCl(DMS)] (**11**) (641 mg, 2.18 mmol, 1.00 eq.) and 2-(4-fluorophenyl)-6-isocyano-1,3-bis(4-methoxyphenyl)azulene (**10b**) (1.00 g, 2.18 mmol, 1.00 eq.) were dissolved in reagent grade dichloromethane. The reaction mixture was stirred at room temperature for 1 h. Purification by flash column chromatography (Silica, dichloromethane : petroleum ether = 1 : 1) yielded the desired product as a green solid (1.12 g, 1.62 mmol, 74%).

MP = 151 – 156 °C;**^{1}H{^{19}F} NMR** (400 MHz, CD_2Cl_2, 295 K) δ [ppm] = 8.19 (d, *J* = 10.7 Hz, 2H, 2x$C_{Ar}\underline{H}$), 7.18 – 7.13 (m, 5H, 5x$C_{Ar}\underline{H}$), 7.12 (s, 1H, $C_{Ar}\underline{H}$), 7.03 (dd, *J* = 9.0, 5.5 Hz, 2H, 2x$C_{Ar}\underline{H}$), 6.94 (d, *J* = 8.9 Hz, 4H, 4x$C_{Ar}\underline{H}$), 6.87 (t, *J* = 8.9 Hz, 2H, 2x$C_{Ar}\underline{H}$), 3.84 (s, 6H, 2x-$OC\underline{H_3}$); **^{13}C{^{19}F, ^{1}H} NMR** (101 MHz, CD_2Cl_2, 295 K) δ [ppm] = 163.89 (s, 1C, $\underline{C}$N), 161.43

(s, 2C, 2x$\underline{C}_{Ar}$), 159.51 (s, 2C, 2x$\underline{C}_{Ar}$), 151.32 (s, 2C, 2x$\underline{C}_{Ar}$), 137.90 (s, 2C, 2x$\underline{C}_{Ar}$), 134.01 (d, 2C, 2x$\underline{C}_{Ar}$H), 133.40 (d, 2C, 2x$\underline{C}_{Ar}$H), 133.32 (d, 2C, 2x$\underline{C}_{Ar}$H), 132.91 (s, 2C, 2x$\underline{C}_{Ar}$), 132.78 (d, 2C, 2x$\underline{C}_{Ar}$H), 127.14 (s, 2C, 2x$\underline{C}_{Ar}$), 120.96 (d, 2C, 2x$\underline{C}_{Ar}$H), 115.45 (d, 2C, 2x$\underline{C}_{Ar}$H), 115.23 (d, 2C, 2x$\underline{C}_{Ar}$H), 114.36 (d, 2C, 2x$\underline{C}_{Ar}$H), 55.69 (q, 2C, 2x-O$\underline{C}H_3$); **^{19}F{^{1}H} NMR** (283 MHz, CD_2Cl_2, 298 K) δ [ppm] = -114.59 (s, 1F, $C_{Ar}\underline{F}$); **IR** (ATR) $\tilde{\nu}$ [cm^{-1}] = 3028 (w), 3005 (w), 2962 (w), 2931 (w), 2904 (w), 2834 (w), 2207 (m), 2045 (w), 1732 (m), 1605 (m), 1569 (w), 1514 (s), 1442 (m), 1403 (m), 1289 (m), 1247 (s), 1227 (s), 1173 (s), 1156 (m), 1108 (m), 1029 (s), 948 (w), 853 (s), 838 (s), 827 (s), 794 (s), 739 (s), 697 (w), 631 (w), 609 (w); **HR-MS** (MALDI): $C_{31}H_{22}AuClFNO_2$ $[M]^+$ calcd.: 691.0989, found.: 691.0991, Δm/z = 0.289 ppm; **UV/VIS** (CH_2Cl_2) λ_{max} (ε) [nm (l*mol^{-1}*cm^{-1})] = 263 (27258), 334 (59758), 432 (8177), 683 (325), 741 (283).

Synthesis of ((diethylamino)((2-(2-fluorophenyl)-1,3-bis(4-methoxyphenyl)azulen-4-yl)amino)methylene)gold(I) chloride (14aa)

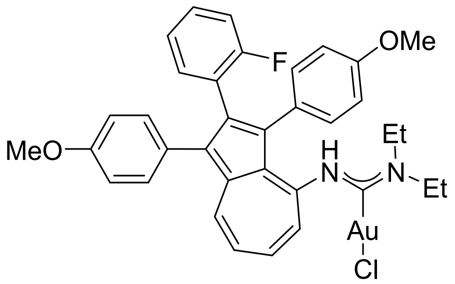

According **GP1** 2-(2-fluorophenyl)-4-isocyano-1,3-bis(4-methoxyphenyl)azulene gold(I)chloride (**12a**) (50.0 mg, 72.3 µmol, 1.0 eq.) and diethylamine (**13a**) (5.55 mg, 75.9 µmol, 1.05 eq.) was stirred in dichloromethane at room temperature 12 hours. Purification by flash column chromatography (Silica, dichloromethane) yielded the title compound as a blue solid (37.0 mg, 72.3 µmol, 67%).

According to **GP2** [AuCl(DMS)] (**11**) (52.6 mg, 179 µmol, 1.00 eq.) and 2-(2-fluorophenyl)-4-isocyano-1,3-bis(4-methoxyphenyl)azulene (**10a**) (82.0 mg, 178 µmol, 1.00 eq.) were stirred in dichloromethane for 1 hour at room temperature. Afterwards, diethylamine (**13a**) (13.7 mg, 187 µmol, 1.05 eq.) weas added, the mixture stirred at room temperature for additional 12 hours. Purification by flash column chromatography (Silica, dichloromethane) yielded the title compound as a blue solid (91.0 mg, 119 µmol, 67%).

MP = 189 – 194 °C;**^{1}H{^{19}F} NMR** (500 MHz, C_6D_6, 295 K) δ [ppm] = 8.11 (dd, *J* = 9.6, 1.1 Hz, 1H, $C_{Ar}\underline{H}$), 7.69 (d, *J* = 10.6 Hz, 1H, $C_{Ar}\underline{H}$), 7.15 (d, *J* = 8.4 Hz, 2H, 2x$C_{Ar}\underline{H}$), 7.10 – 7.03 (m, 1H, $C_{Ar}\underline{H}$), 6.99 (s, 1H, N$\underline{H}$), 6.82 (ddd, *J* = 9.4, 7.9, 2.0 Hz, 2H, 2x$C_{Ar}\underline{H}$), 6.73 (dd, *J* =

8.4, 2.3 Hz, 1H, $C_{Ar}\underline{H}$), 6.60 (d, J = 8.9 Hz, 2H, 2x$C_{Ar}\underline{H}$), 6.56 (ddd, J = 8.7, 7.0, 1.7 Hz, 1H, $C_{Ar}\underline{H}$), 6.53 – 6.42 (m, 3H, 3x$C_{Ar}\underline{H}$), 6.28 (dd, J = 8.5, 2.9 Hz, 1H, $C_{Ar}\underline{H}$), 6.23 (dd, J = 8.5, 2.9 Hz, 1H, $C_{Ar}\underline{H}$), 3.43 (dq, J = 14.2, 7.2 Hz, 1H, -C$\underline{H}_2$-), 3.13 (dt, J = 13.5, 7.1 Hz, 1H, -C$\underline{H}_2$-), 3.09 (s, 3H, -OC$\underline{H}_3$), 2.98 (s, 3H, -OC$\underline{H}_3$), 1.47 (dq, J = 14.8, 7.3 Hz, 1H, -C$\underline{H}_2$-), 1.35 (dq, J = 14.6, 7.2 Hz, 1H, -C$\underline{H}_2$-), 0.62 (t, J = 7.2 Hz, 3H, -C$\underline{H}_3$), 0.03 (t, J = 7.3 Hz, 3H, -C$\underline{H}_3$); **^{13}C{^{19}F, ^{1}H} NMR** (126 MHz, C_6D_6, 295 K) δ [ppm] = 191.52 (s, 1C, $\underline{C}_{Carbene}$), 161.68 (s, 1C, $\underline{C}_{Ar}$), 159.72 (s, 1C, $\underline{C}_{Ar}$), 159.22 (s, 1C, $\underline{C}_{Ar}$), 158.80 (s, 1C, $\underline{C}_{Ar}$), 146.41 (s, 1C, $\underline{C}_{Ar}$), 143.66 (s, 1C, $\underline{C}_{Ar}$), 138.88 (s, 1C, $\underline{C}_{Ar}$), 137.43 (d, 1C, $\underline{C}_{Ar}$H), 136.60 (d, 1C, $\underline{C}_{Ar}$H), 134.23 (d, 1C, $\underline{C}_{Ar}$H), 133.10 (d, 1C, $\underline{C}_{Ar}$H), 132.99 (d, 1C, $\underline{C}_{Ar}$H), 132.59 (d, 1C, $\underline{C}_{Ar}$H), 132.41 (s, 1C, $\underline{C}_{Ar}$), 129.55 (s, 1C, $\underline{C}_{Ar}$), 129.30 (d, 1C, $\underline{C}_{Ar}$H), 128.59 (d, 1C, $\underline{C}_{Ar}$H), 127.47 (s, 1C, $\underline{C}_{Ar}$), 126.64 (s, 1C, $\underline{C}_{Ar}$), 125.75 (s, 1C, $\underline{C}_{Ar}$), 123.78 (d, 1C, $\underline{C}_{Ar}$H), 123.53 (d, 1C, $\underline{C}_{Ar}$H), 123.21 (d, 1C, $\underline{C}_{Ar}$H), 115.68 (d, 1C, $\underline{C}_{Ar}$H), 115.51 (d, 1C, $\underline{C}_{Ar}$H), 114.20 (d, 1C, $\underline{C}_{Ar}$H), 113.59 (d, 1C, $\underline{C}_{Ar}$H), 113.47 (d, 1C, $\underline{C}_{Ar}$H), 54.83 (q, 1C, -O$\underline{C}H_3$), 54.74 (q, 1C, -O$\underline{C}H_3$), 54.02 (t, 1C, -$\underline{C}H_2$-), 40.15 (t, 1C, -$\underline{C}H_2$-), 14.39 (q, 1C, -CH_3), 11.09 (q, 1C, -CH_3); **^{19}F{^{1}H} NMR** (471 MHz, C_6D_6, 295 K) δ [ppm] = -112.06 (s, 1F, $C_{Ar}\underline{F}$); **IR** (ATR) $\tilde{\nu}$ [cm^{-1}] = 3317, 2973, 2932, 2834, 1731, 1607, 1568, 1539, 1516, 1479, 1450, 1383, 1345, 1324, 1285, 1243, 1210, 1175, 1106, 1092, 1030, 934, 832, 812, 792, 764, 754, 730, 638; **HR-MS** (MALDI): $C_{35}H_{33}AuClFN_2O_2$ $[M]^+$ calcd.: 764.1880, found.: 764.1875, Δm/z = 0.654 ppm; **UV/VIS** (CH_2Cl_2) λ_{max} (ε) [nm (l*mol^{-1}*cm^{-1})] = 277 (865184), 319 (765080), 381 (779381), 652 (2045), 606 (315); **Elemental Analysis** calcd. for $C_{35}H_{33}AuClFN_2O_2$: C: 54.95, H: 4.35, N : 3.66, found C: 54.49, H: 4.81, N: 3.07.

Synthesis of (((2-(2-fluorophenyl)-1,3-bis(4-methoxyphenyl)azulen-4-yl)amino)(pyrrolidin-1-yl)methyl)gold(I) chloride (14ab)

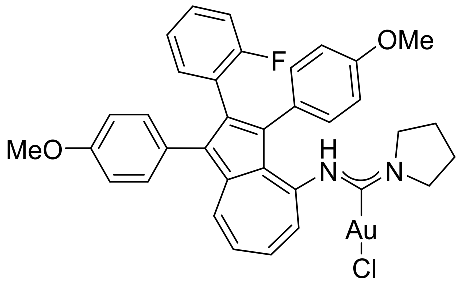

According to **GP1** 2-(2-fluorophenyl)-4-isocyano-1,3-bis(4-methoxyphenyl)azulene gold(I)chloride (**12a**) (50.0 mg, 72.3 µmol, 1.00 eq.) and pyrrolidine (**13b**) (5.40 mg, 75.9 µmol, 1.05 eq.) was stirred in dichloromethane at room temperature 12 hours. Purification by flash column chromatography (Silica, dichloromethane) yielded the title compound as a blue solid (36.0 mg, 47.2 µmol, 65%).

MP = 213 – 218 °C; **^{1}H{^{19}F} NMR** (301 MHz, C_6D_6, 295 K) δ [ppm] = 8.33 (dd, *J* = 9.7, 1.2 Hz, 1H, $C_{Ar}\underline{H}$), 8.13 (d, *J* = 10.6 Hz, 1H, N$\underline{H}$), 7.35 (d, *J* = 8.5 Hz, 2H, 2x$C_{Ar}\underline{H}$), 7.32 – 7.27 (m, 1H, $C_{Ar}\underline{H}$), 7.08 – 6.98 (m, 3H, 3x$C_{Ar}\underline{H}$), 6.93 (dd, *J* = 8.4, 2.2 Hz, 1H, $C_{Ar}\underline{H}$), 6.80 (d, *J* = 8.9 Hz, 2H, 2x$C_{Ar}\underline{H}$), 6.76 – 6.62 (m, 4H, 4x$C_{Ar}\underline{H}$), 6.44 (ddd, *J* = 16.8, 8.5, 2.8 Hz, 2H, 2x$C_{Ar}\underline{H}$), 3.75 – 3.42 (m, 2H, -C$\underline{H_2}$-), 3.30 (s, 3H, -OC$\underline{H_3}$), 3.15 (s, 3H, -OC$\underline{H_3}$), 1.64 – 1.42 (m, 1H, -C$\underline{H_2}$-), 1.30 (q, *J* = 5.4 Hz, 1H, -C$\underline{H_2}$-), 1.19 – 1.02 (m, 4H, 2x-C$\underline{H_2}$-); **^{13}C{^{19}F, ^{1}H} NMR** (176 MHz, C_6D_6, 295 K) δ [ppm] = 189.84 (s, 1C, $\underline{C_{Carbene}}$), 160.03 (s, 1C, $\underline{C_{Ar}}$), 159.32 (s, 1C, $\underline{C_{Ar}}$), 158.81 (s, 1C, $\underline{C_{Ar}}$), 146.37 (s, 1C, $\underline{C_{Ar}}$), 143.52 (s, 1C, $\underline{C_{Ar}}$), 138.95 (s, 1C, $\underline{C_{Ar}}$), 137.56 (d, 1C, $\underline{C_{Ar}}$H), 136.86 (d, 1C, $\underline{C_{Ar}}$H), 134.71 (d, 1C, $\underline{C_{Ar}}$H), 133.25 (d, 1C, $\underline{C_{Ar}}$H), 133.10 (d, 1C, $\underline{C_{Ar}}$H), 132.58 (d, 1C, $\underline{C_{Ar}}$H), 132.43 (s, 1C, $\underline{C_{Ar}}$), 129.62 (s, 1C, $\underline{C_{Ar}}$), 129.30 (d, 1C, $\underline{C_{Ar}}$H), 129.25 (d, 1C, $\underline{C_{Ar}}$H), 127.30 (s, 1C, $\underline{C_{Ar}}$), 126.60 (s, 1C, $\underline{C_{Ar}}$), 125.74 (s, 1C, $\underline{C_{Ar}}$), 125.66 (s, 1C, $\underline{C_{Ar}}$), 123.77 (d, 1C, $\underline{C_{Ar}}$H), 123.57 (d, 1C, $\underline{C_{Ar}}$H), 122.78 (d, 1C, $\underline{C_{Ar}}$H), 115.63 (d, 1C, $\underline{C_{Ar}}$H), 115.59 (d, 1C, $\underline{C_{Ar}}$H), 114.22 (d, 1C, $\underline{C_{Ar}}$H), 113.13 (d, 1C, $\underline{C_{Ar}}$H), 113.01 (d, 1C, $\underline{C_{Ar}}$H), 56.34 (t, 1C, -$\underline{C}H_2$-), 54.78 (q, 1C, -O$\underline{C}H_3$), 54.71 (q, 1C, -O$\underline{C}H_3$), 44.06 (t, 1C, -$\underline{C}H_2$-), 24.75 (t, 1C, -$\underline{C}H_2$-), 23.91 (t, 1C, -$\underline{C}H_2$-); **^{19}F{^{1}H} NMR** (283 MHz, C_6D_6, 295 K) δ [ppm] = -112.00 (s, 1F, $C_{Ar}\underline{F}$); **IR** (ATR) $\tilde{\nu}$ [cm^{-1}] = 3319 (m), 2935 (w), 2835 (w), 1708 (s), 1607 (m), 1589 (m), 1538 (s), 1459 (m), 1449 (m), 1364 (w), 1294 (m), 1264 (s), 1224 (s), 1176 (s), 1124 (s), 1108 (s), 950 (w), 929 (w), 825 (w), 775 (w), 728 (w), 638 (w); **HR-MS** (MALDI): $C_{35}H_{31}AuClFN_2O_2$ $[M]^+$ calcd.: 762.1724, found.: 762.1729, Δm/z = 0.656 ppm ; **UV/VIS** (CH_2Cl_2) λ_{max} (ε) [nm (l*mol^{-1}*cm^{-1})] = 277 (37270), 321 (37270), 380 (7043), 608 (704); **Elemental Analysis** calcd. for $C_{35}H_{31}AuClFN_2O_2$: C: 55.09, H: 4.10, N : 3.67, found C: 55.05, H: 4.33, N: 3.58.

Synthesis of (((2-(2-fluorophenyl)-1,3-bis(4-methoxyphenyl)azulen-4-yl)amino)(piperidin-1-yl)methylene)gold(I) chloride (14ac)

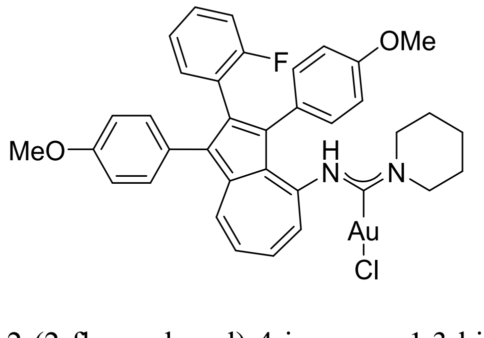

According to **GP1** 2-(2-fluorophenyl)-4-isocyano-1,3-bis(4-methoxyphenyl)azulene gold(I)chloride (**12a**) (50.0 mg, 72.3 µmol, 1.00eq.) and piperidine (**13c**) (6.46 mg, 75.9 µmol, 1.05 eq.) was stirred in dichloromethane at room temperature 12 hours. Purification by flash

column chromatography (Silica, dichloromethane) yielded the title compound as a blue solid (38.0 mg, 48.9 µmol, 68%).

According to **GP2** [AuCl(DMS)] (52.6 mg, 179 µmol, 1.00 eq.) and 2-(2-fluorophenyl)-4-isocyano-1,3-bis(4-methoxyphenyl)azulene (82.0 mg, 178 µmol, 1.00 eq.) were stirred in dichloromethane for 1 hour at room temperature. Afterwards, piperidine (16.0 mg, 187 µmol, 1.05 eq.) was added, the mixture stirred at room temperature for additional 12 hours. Purification by flash column chromatography (Silica, dichloromethane) yielded the title compound as a blue solid (90.0 mg, 116 µmol, 65%).

MP = 164 – 169 °C; **^{1}H{^{19}F} NMR** (600 MHz, C_6D_6, 295 K) δ [ppm] = 8.32 (dd, *J* = 9.6, 1.2 Hz, 1H, NH), 7.91 (d, *J* = 10.5 Hz, 1H, $C_{Ar}H$), 7.36 (d, *J* = 8.0 Hz, 1H, $C_{Ar}H$), 7.26 (ddd, *J* = 10.7, 9.8, 1.2 Hz, 1H, $C_{Ar}H$), 7.05 (s, 1H, $C_{Ar}H$), 7.03 – 6.97 (m, 2H, 2x$C_{Ar}H$), 6.91 (dd, *J* = 8.4, 2.3 Hz, 1H, $C_{Ar}H$), 6.82 – 6.78 (m, 2H, 2x$C_{Ar}H$), 6.78 – 6.71 (m, 2H, 2x$C_{Ar}H$), 6.69 (td, *J* = 9.6, 0.7 Hz, 1H, $C_{Ar}H$), 6.67 – 6.63 (m, 1H, $C_{Ar}H$), 6.43 (dd, *J* = 8.4, 2.8 Hz, 1H, $C_{Ar}H$), 6.39 (dd, *J* = 8.4, 2.9 Hz, 1H, $C_{Ar}H$), 4.01 – 3.82 (m, 1H, -CH_2-), 3.64 (ddd, *J* = 13.1, 7.2, 4.6 Hz, 1H, -CH_2-), 3.28 (s, 3H, -OCH_3), 3.14 (s, 3H, -OCH_3), 1.78 (ddd, *J* = 13.3, 7.0, 4.0 Hz, 1H, -CH_2-), 1.64 (ddd, *J* = 12.9, 7.8, 4.1 Hz, 1H, -CH_2-), 1.06 (dp, *J* = 7.4, 3.1 Hz, 2H, -CH_2-), 0.95 – 0.82 (m, 2H, -CH_2-), 0.55 (dddd, *J* = 14.2, 11.3, 7.5, 4.2 Hz, 2H, -CH_2-); **^{13}C{^{19}F, ^{1}H} NMR** (151 MHz, C_6D_6, 295 K) δ [ppm] = 190.88 (s, 1C, $C_{Carbene}$), 161.56 (s, 1C, C_{Ar}), 159.92 (s, 1C, C_{Ar}), 159.23 (s, 1C, C_{Ar}), 158.86 (s, 1C, C_{Ar}), 146.23 (s, 1C, C_{Ar}), 143.47 (s, 1C, C_{Ar}), 138.87 (s, 1C, C_{Ar}), 137.50 (s, 1C, C_{Ar}), 136.68 (d, 1C, $C_{Ar}H$), 134.73 (d, 1C, $C_{Ar}H$), 133.05 (d, 1C, $C_{Ar}H$), 132.60 (d, 1C, $C_{Ar}H$), 132.30 (s, 1C, C_{Ar}), 129.29 (d, 1C, $C_{Ar}H$), 129.25 (d, 1C, $C_{Ar}H$), 127.61 (s, 1C, C_{Ar}), 127.41 (s, 1C, C_{Ar}), 126.65 (d, 1C, $C_{Ar}H$), 125.77 (s, 1C, C_{Ar}), 123.80 (d, 1C, $C_{Ar}H$), 123.78 (d, 1C, $C_{Ar}H$), 123.58 (d, 1C, $C_{Ar}H$), 122.74 (d, 1C, $C_{Ar}H$), 115.70 (d, 1C, $C_{Ar}H$), 115.55 (d, 1C, $C_{Ar}H$), 114.21 (d, 1C, $C_{Ar}H$), 113.51 (d, 1C, $C_{Ar}H$), 113.05 (d, 1C, $C_{Ar}H$), 58.28(t, 1C, -CH_2-), 54.83 (q, 1C, -OCH_3), 54.71 (q, 1C, -OCH_3), 44.66 (t, 1C, -CH_2-), 25.83 (t, 1C, -CH_2-), 24.81 (t, 1C, -CH_2-), 23.55 (t, 1C, -CH_2-); **^{19}F{^{1}H} NMR** (283 MHz, C_6D_6, 295 K) δ [ppm] = -112.17; **IR** (ATR) $\tilde{\nu}$ [cm^{-1}] = 3318, 2930, 2853, 2836, 1607, 1568, 1538, 1515, 1480, 1450, 1353, 1318, 1284, 1243, 1175, 1105, 1029, 950, 834, 807, 753, 729, 634; **HR-MS** (MALDI): $C_{36}H_{33}AuClFN_2O_2$ $[M]^+$ calcd.: 776.1880, found.: 776.1875, Δm/z = 0.644 ppm; **UV/VIS** (CH_2Cl_2) λ_{max} (ε) [nm ($l*mol^{-1}*cm^{-1}$)] = 278 (53730), 323 (47514), 381 (48402), 607 (1270); **Elemental Analysis** calcd. for $C_{36}H_{33}AuClFN_2O_2$: C: 55.64, H: 4.28, N : 3.61, found C: 55.68, H: 4.59, N: 3.57.

Synthesis of (((2,6-dimethylphenyl)amino)((2-(2-fluorophenyl)-1,3-bis(4-methoxyphenyl)azulen-4-yl)amino)methylene)gold(III) chloride (14ad)

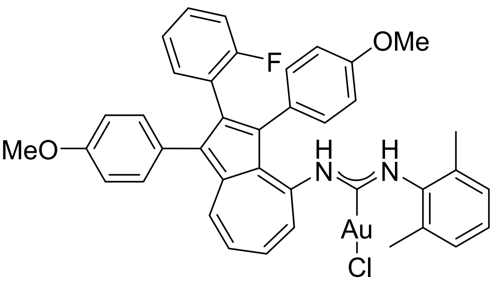

According to **GP1** 2-(2-fluorophenyl)-4-isocyano-1,3-bis(4-methoxyphenyl)azulene gold(I)chloride (**12a**) (50.0 mg, 72.3 µmol, 1.00 eq.) and 2,6-dimethylaniline (**13d**) (9.19 mg, 75.9 µmol, 1.05 eq.) was stirred in dichloromethane at room temperature 12 hours. Purification by flash column chromatography (Silica, dichloromethane) yielded the title compound as a blue solid (36.0 mg, 44.3 µmol, 62%).

MP = 222 – 227 °C;**^{1}H{^{19}F} NMR** (301 MHz, C_6D_6, 295 K) δ [ppm] = 10.28 (s, 1H, N$\underline{H}$), 8.31 – 8.28 (m, 1H, N$\underline{H}$), 8.27 (d, *J* = 2.5 Hz, 1H, $C_{Ar}\underline{H}$), 8.14 (s, 1H, $C_{Ar}\underline{H}$), 7.37 – 7.29 (m, 1H, $C_{Ar}\underline{H}$), 7.26 (d, *J* = 8.8 Hz, 2H, 2x$C_{Ar}\underline{H}$), 6.91 (d, *J* = 7.6 Hz, 1H, $C_{Ar}\underline{H}$), 6.84 (td, *J* = 7.4, 1.8 Hz, 2H, 2x$C_{Ar}\underline{H}$), 6.74 (s, 1H, $C_{Ar}\underline{H}$), 6.71 (s, 2H, 2x$C_{Ar}\underline{H}$), 6.69 (s, 1H, $C_{Ar}\underline{H}$), 6.65 (s, 1H, $C_{Ar}\underline{H}$), 6.63 (s, 1H, $C_{Ar}\underline{H}$), 6.60 (d, *J* = 1.8 Hz, 1H, $C_{Ar}\underline{H}$), 6.57 (s, 1H, $C_{Ar}\underline{H}$), 6.54 (dd, *J* = 7.2, 1.6 Hz, 1H, $C_{Ar}\underline{H}$), 6.20 (dd, *J* = 8.3, 2.8 Hz, 1H, $C_{Ar}\underline{H}$), 6.11 (dd, *J* = 8.4, 2.9 Hz, 1H, $C_{Ar}\underline{H}$), 3.24 (s, 3H, -OC$\underline{H_3}$), 3.15 (s, 3H, -OC$\underline{H_3}$), 1.93 (s, 3H, -C$\underline{H_3}$), 1.87 (s, 3H, -C$\underline{H_3}$); **^{13}C{^{19}F, ^{1}H} NMR** (176 MHz, C_6D_6, 295 K) δ [ppm] = 188.81 (s, 1C, $\underline{C_{Carbene}}$), 161.22 (s, 1C, $\underline{C_{Ar}}$), 159.80 (s, 1C, $\underline{C_{Ar}}$), 159.13 (s, 1C, $\underline{C_{Ar}}$), 158.33 (s, 1C, $\underline{C_{Ar}}$), 146.15 (s, 1C, $\underline{C_{Ar}}$), 144.40 (s, 1C, $\underline{C_{Ar}}$), 143.30 (s, 1C, $\underline{C_{Ar}}$), 138.88 (s, 1C, $\underline{C_{Ar}}$), 137.49 (d, 1C, $\underline{C_{Ar}}$H), 136.45 (d, 1C, $\underline{C_{Ar}}$H), 134.32 (s, 1C, $\underline{C_{Ar}}$), 134.11 (s, 1C, $\underline{C_{Ar}}$), 132.73 (d, 1C, $\underline{C_{Ar}}$H), 132.57 (d, 1C, $\underline{C_{Ar}}$H), 132.44 (s, 1C, $\underline{C_{Ar}}$), 131.84 (d, 1C, $\underline{C_{Ar}}$), 131.59 (d, 1C, $\underline{C_{Ar}}$H), 129.28 (d, 1C, $\underline{C_{Ar}}$H), 128.59 (d, 1C, $\underline{C_{Ar}}$H), 128.35 (d, 1C, $\underline{C_{Ar}}$H), 128.31 (s, 1C, $\underline{C_{Ar}}$), 125.60 (s, 1C, $\underline{C_{Ar}}$), 125.49 (s, 1C, $\underline{C_{Ar}}$), 125.21 (s, 1C, $\underline{C_{Ar}}$), 123.42 (d, 1C, $\underline{C_{Ar}}$H), 121.60 (d, 1C, $\underline{C_{Ar}}$H), 121.40 (d, 1C, $\underline{C_{Ar}}$H), 118.18 (d, 1C, $\underline{C_{Ar}}$H), 115.40 (d, 1C, $\underline{C_{Ar}}$H), 115.28 (d, 1C, $\underline{C_{Ar}}$H), 114.10 (d, 1C, $\underline{C_{Ar}}$H), 113.96 (d, 1C, $\underline{C_{Ar}}$H), 113.84 (d, 1C, $\underline{C_{Ar}}$H), 113.63 (d, 1C, $\underline{C_{Ar}}$H), 54.67 (q, 1C, -O$\underline{C}H_3$), 54.35 (q, 1C, -O$\underline{C}H_3$), 19.07 (q, 1C, -$\underline{C}H_3$), 19.00 (q, 1C, -$\underline{C}H_3$);**^{19}F{^{1}H} NMR** (283 MHz, C_6D_6, 295 K) δ [ppm] = -111.85 (s, 1F, $C_{Ar}\underline{F}$); **IR** (ATR) $\tilde{\nu}$ [cm^{-1}] = 3259 (m), 3204 (m), 2965 (w), 2834 (w), 1607 (m), 1569 (m), 1515 (s), 1473 (m), 1449 (m), 1386 (w), 1264 (s), 1224 (s), 1175 (s), 1107 (s), 1030 (s), 831 (w), 753 (w), 739 (w), 617 (w); **HR-MS** (MALDI): $C_{39}H_{33}AuClFN_2O_2$ $[M]^+$ calcd.: 812.1880, found.: 812.1879, Δm/z = 0.123 ppm; **UV/VIS** (CH_2Cl_2) λ_{max} (ε) [nm (l*mol^{-1}*cm^{-1})] = 279 (23461),

321 (23461), 385 (3871), 613 (607); **Elemental Analysis** calcd. for $C_{39}H_{33}AuClFN_2O_2$: C: 57.61, H: 4.09, N : 3.45, found C: 57.04, H: 4.03, N: 3.42.

Synthesis of (((2-(2-fluorophenyl)-1,3-bis(4-methoxyphenyl)azulen-4-yl)amino)(mesitylamino)methylene)gold(I) chloride (14ae)

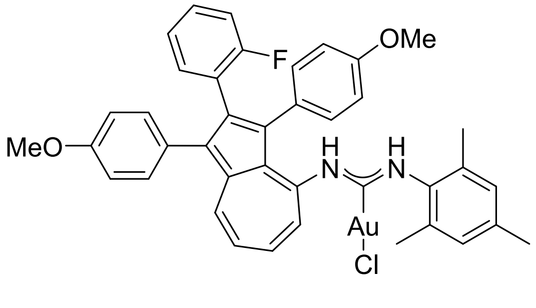

According to **GP1** 2-(2-fluorophenyl)-4-isocyano-1,3-bis(4-methoxyphenyl)azulene gold(I)chloride (**12a**) (50.0 mg, 72.3 μmol, 1.00 eq.) and mesitylamine (**13e**) (10.2 mg, 75.9 μmol, 1.05 eq.) was stirred in dichloromethane at room temperature 12 hours. Purification by flash column chromatography (Silica, dichloromethane) yielded the title compound as a blue solid (39.0 mg, 47.2 μmol, 65%).

MP = 177 – 182 °C;**^{1}H{^{19}F} NMR** (500 MHz, C_6D_6, 295 K) δ [ppm] = 10.08 (s, 1H, N$\underline{H}$), 8.16 (d, *J* = 10.6 Hz, 1H, $C_{Ar}\underline{H}$), 8.11 (dd, *J* = 9.6, 1.3 Hz, 1H, $C_{Ar}\underline{H}$), 7.99 (s, 1H, N$\underline{H}$), 7.16 (ddd, *J* = 10.9, 9.9, 1.3 Hz, 1H, $C_{Ar}\underline{H}$), 7.09 (d, *J* = 8.8 Hz, 2H, 2x$C_{Ar}\underline{H}$), 6.67 (td, *J* = 7.4, 1.8 Hz, 1H, $C_{Ar}\underline{H}$), 6.60 (dd, *J* = 8.5, 2.4 Hz, 1H, $C_{Ar}\underline{H}$), 6.56 (d, *J* = 8.9 Hz, 2H, 2x$C_{Ar}\underline{H}$), 6.54 – 6.51 (m, 1H, $C_{Ar}\underline{H}$), 6.50 (d, *J* = 9.7 Hz, 1H, $C_{Ar}\underline{H}$), 6.46 (ddd, *J* = 8.8, 5.3, 1.6 Hz, 1H, $C_{Ar}\underline{H}$), 6.42 (dd, *J* = 9.4, 1.3 Hz, 1H, $C_{Ar}\underline{H}$), 6.38 (td, *J* = 7.3, 1.3 Hz, 1H, $C_{Ar}\underline{H}$), 6.26 (d, *J* = 12.2 Hz, 2H, 2x$C_{Ar}\underline{H}$), 6.03 (dd, *J* = 8.4, 2.8 Hz, 1H, $C_{Ar}\underline{H}$), 5.95 (dd, *J* = 8.5, 2.7 Hz, 1H, $C_{Ar}\underline{H}$), 3.07 (s, 3H, $-OCH_3$), 2.99 (s, 3H, $-OCH_3$), 1.78 (s, 3H, $-CH_3$), 1.75 (s, 3H, $-CH_3$), 1.69 (s, 3H, $-CH_3$); **^{13}C{^{19}F, ^{1}H} NMR** (101 MHz, C_6D_6, 295 K) δ [ppm] = 188.94 (s, 1C, $\underline{C}_{Carbene}$), 161.78 (s, 1C, $\underline{C}_{Ar}$), 159.33 (s, 1C, $\underline{C}_{Ar}$), 159.15 (s, 1C, $\underline{C}_{Ar}$), 158.49 (s, 1C, $\underline{C}_{Ar}$), 146.32 (s, 1C, $\underline{C}_{Ar}$), 144.35 (s, 1C, $\underline{C}_{Ar}$), 138.91 (s, 1C, $\underline{C}_{Ar}$), 137.70 (s, 1C, $\underline{C}_{Ar}$), 137.46 (d, 1C, $\underline{C}_{Ar}$H), 136.46 (d, 1C, $\underline{C}_{Ar}$H), 133.97 (s, 1C, $\underline{C}_{Ar}$), 133.80 (s, 1C, $\underline{C}_{Ar}$), 132.81 (d, 1C, $\underline{C}_{Ar}$H), 132.78 (d, 1C, $\underline{C}_{Ar}$H), 132.59 (d, 1C, $\underline{C}_{Ar}$H), 132.44 (s, 1C, $\underline{C}_{Ar}$), 131.92 (d, 1C, $\underline{C}_{Ar}$H), 131.73 (d, 1C, $\underline{C}_{Ar}$H), 130.44 (s, 1C, $\underline{C}_{Ar}$), 130.02 (d, 1C, $\underline{C}_{Ar}$H), 129.03 (d, 1C, $\underline{C}_{Ar}$H), 128.70 (s, 1C, $\underline{C}_{Ar}$), 128.51 (s, 1C, $\underline{C}_{Ar}$), 125.73 (s, 1C, $\underline{C}_{Ar}$), 125.56 (s, 1C, $\underline{C}_{Ar}$), 125.16 (d, 1C, $\underline{C}_{Ar}$H), 123.43 (d, 1C, $\underline{C}_{Ar}$H), 123.16 (d, 1C, $\underline{C}_{Ar}$H), 121.62 (d, 1C, $\underline{C}_{Ar}$H), 115.45 (d, 1C, $\underline{C}_{Ar}$H), 115.23 (d, 1C, $\underline{C}_{Ar}$H), 114.10 (d, 1C, $\underline{C}_{Ar}$H), 113.73 (d, 1C, $\underline{C}_{Ar}$H), 113.44 (d, 1C, $\underline{C}_{Ar}$H), 54.68 (q, 1C, $-O\underline{C}H_3$), 54.40 (q, 1C, $-O\underline{C}H_3$), 20.84 (q, 1C, $-\underline{C}H_3$), 19.01 (q, 1C, $-\underline{C}H_3$), 18.95 (q, 1C, $-\underline{C}H_3$); **^{19}F{^{1}H} NMR** (471

MHz, C_6D_6, 295 K) δ [ppm] = -111.80 (s, 1F, $C_{Ar}\underline{F}$); **IR** (ATR) $\tilde{\nu}$ [cm^{-1}] = 3263 (m), 3205 (m), 2965 (w), 2934 (w), 1708 (s), 1608 (m), 1569 (m), 1516 (s), 1475 (m), 1449 (m), 1384 (w), 1264 (s), 1224 (s), 1175 (s), 1107 (s), 1030 (s), 829 (w), 754 (w), 729 (w), 619 (w); **HR-MS** (MALDI): $C_{40}H_{35}AuClFN_2O_2$ $[M]^+$ calcd.: 826.2037, found.: 826.2033, Δm/z = 0.484 ppm; **UV/VIS** (CH_2Cl_2) λ_{max} (ε) [nm ($l*mol^{-1}*cm^{-1}$)] = 281 (42780), 322 (43049), 614 (615); **Elemental Analysis** calcd. for $C_{40}H_{35}AuClFN_2O_2$: C: 58.08, H: 4.27, N : 3.39, found C: 58.95, H: 4.93, N: 3.40.

Synthesis of (((2,6-diisopropylphenyl)amino)((2-(2-fluorophenyl)-1,3-bis(4-methoxyphenyl)azulen-4-yl)amino)methylene)gold(I) chloride (14af)

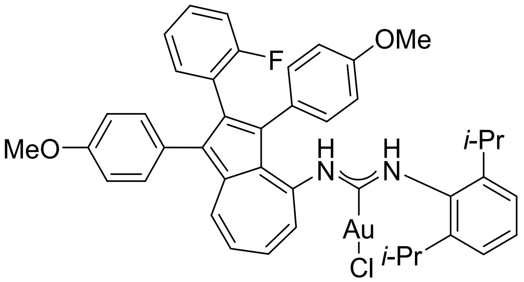

According to **GP1** 2-(2-fluorophenyl)-4-isocyano-1,3-bis(4-methoxyphenyl)azulene gold(I)chloride (**12a**) (50.0 mg, 72.3 µmol, 1.00 eq.) and 2,6-diisopropylaniline (**13f**) (13.5 mg, 75.9 µmol, 1.05 eq.) was stirred in dichloromethane at room temperature 12 hours. Purification by flash column chromatography (Silica, dichloromethane) yielded the title compound as a blue solid (33.0 mg, 38.0µmol, 52%).

According to **GP2** [AuCl(DMS)] (**11**) (52.6 mg, 179 µmol, 1.00 eq.) and 2-(2-fluorophenyl)-4-isocyano-1,3-bis(4-methoxyphenyl)azulene (**10a**) (82.0 mg, 178 µmol, 1.00 eq.) were stirred in dichloromethane for 1 hour at room temperature. Afterwards, 2,6-diisopropylaniline (**13f**) (47.5 mg, 268 µmol, 1.05 eq.) was added, the mixture stirred at room temperature for additional 12 hours. Purification by flash column chromatography (Silica, dichloromethane) yielded the title compound as a blue solid (68.0 mg, 87.5 µmol, 49%).

MP = 169 – 174 °C;**^{1}H{^{19}F} NMR** (500 MHz, C_6D_6, 295 K) δ [ppm] = 10.10 (s, 1H, N$\underline{H}$), 8.33 (s, 1H, N$\underline{H}$), 8.02 (dd, *J* = 9.4, 1.2 Hz, 1H, $C_{Ar}\underline{H}$), 7.79 (d, *J* = 10.6 Hz, 1H, $C_{Ar}\underline{H}$), 7.11 – 7.06 (m, 2H, 2x$C_{Ar}\underline{H}$), 7.01 (d, *J* = 8.8 Hz, 2H, 2x$C_{Ar}\underline{H}$), 6.87 (dd, *J* = 7.4, 1.8 Hz, 1H, $C_{Ar}\underline{H}$), 6.81 (t, *J* = 7.8 Hz, 1H, $C_{Ar}\underline{H}$), 6.76 (s, 1H, $C_{Ar}\underline{H}$), 6.68 (dd, *J* = 7.5, 1.9 Hz, 1H, $C_{Ar}\underline{H}$), 6.63 – 6.58 (m, 2H, 2x$C_{Ar}\underline{H}$), 6.55 – 6.52 (m, 2H, 2x$C_{Ar}\underline{H}$), 6.50 (dd, *J* = 2.9, 1.2 Hz, 1H, $C_{Ar}\underline{H}$), 6.49 – 6.46 (m, 1H, $C_{Ar}\underline{H}$), 6.45 – 6.36 (m, 2H, 2x$C_{Ar}\underline{H}$), 5.96 (d, *J* = 8.8 Hz, 1H, $C_{Ar}\underline{H}$), 3.03 (s, 3H, -OC$\underline{H}_3$), 2.97 (s, 3H, -OC$\underline{H}_3$), 2.82 (p, *J* = 6.8 Hz, 1H, -C$\underline{H}$(CH_3)$_2$), 2.73 (p, *J* = 6.8 Hz, 1H, -

CH(CH$_3$)$_2$), 0.77 (d, J = 6.9 Hz, 3H, -CH(CH$_3$)$_2$), 0.76 – 0.71 (m, 6H, 2x-CH(CH$_3$)$_2$), 0.70 (d, J = 6.9 Hz, 3H, -CH(CH$_3$)$_2$); **^{13}C{^{19}F, ^{1}H} NMR** (126 MHz, C_6D_6, 295 K) δ [ppm] = 191.50 (s, 1C, C$_{Carbene}$), 159.49 (s, 1C, C$_{Ar}$), 157.91 (s, 1C, C$_{Ar}$), 157.01 (s, 1C, C$_{Ar}$), 145.90 (s, 1C, C$_{Ar}$), 143.99 (s, 1C, C$_{Ar}$), 143.95 (s, 1C, C$_{Ar}$), 143.20 (s, 1C, C$_{Ar}$), 139.21 (s, 1C, C$_{Ar}$), 135.79 (d, 1C, C$_{Ar}$H), 134.92 (d, 1C, C$_{Ar}$H), 133.27 (s, 1C, C$_{Ar}$), 131.63 (d, 1C, C$_{Ar}$H), 131.47 (d, 1C, C$_{Ar}$H), 131.34 (d, 1C, C$_{Ar}$H), 130.53 (s, 1C, C$_{Ar}$), 130.43 (d, 1C, C$_{Ar}$H), 129.98 (s, 1C, C$_{Ar}$), 129.56 (s, 1C, C$_{Ar}$), 128.74 (d, 1C, C$_{Ar}$H), 127.91 (d, 1C, C$_{Ar}$H), 127.39 (d, 1C, C$_{Ar}$H), 127.35 (d, 1C, C$_{Ar}$H), 126.21 (d, 1C, C$_{Ar}$H), 124.56 (s, 1C, C$_{Ar}$), 123.47 (d, 1C, C$_{Ar}$H), 123.35 (d, 1C, C$_{Ar}$H), 123.28 (s, 1C, C$_{Ar}$), 122.59 (d, 1C, C$_{Ar}$H), 122.38 (d, 1C, C$_{Ar}$H), 121.69 (s, 1C, C$_{Ar}$), 114.26 (d, 1C, C$_{Ar}$H), 113.35 (d, 1C, C$_{Ar}$H), 113.01 (d, 1C, C$_{Ar}$H), 112.86 (d, 1C, C$_{Ar}$H), 53.46 (q, 1C, -OCH$_3$), 52.99 (q, 1C, -OCH$_3$), 27.97 (d, 1C, -CH(CH$_3$)$_2$), 27.92 (d, 1C, -CH(CH$_3$)$_2$), 23.83 (q, 1C, -CH(CH$_3$)$_2$), 23.78 (q, 1C, -CH(CH$_3$)$_2$), 21.58 (q, 1C, -CH(CH$_3$)$_2$), 21.52 (q, 1C, -CH(CH$_3$)$_2$); **^{19}F{^{1}H} NMR** (471 MHz, C_6D_6, 295 K) δ [ppm] = -112.15 (s, 1F, C$_{Ar}$F); **IR** (ATR) $\tilde{\nu}$ [cm^{-1}] = 3270 (m), 2981 (w), 2929 (w), 2867 (w), 2835 (w), 1609 (m), 1589 (m), 1520 (s), 1450 (m), 1384 (w), 1266 (s), 1214 (s), 1176 (s), 1106 (s), 1031 (s), 933 (w), 831 (w), 725 (w), 728 (w); **HR-MS** (MALDI): $C_{43}H_{41}AuClFN_2O_2$ [M]$^+$ calcd.: 868.2506, found.: 868.2504, Δm/z = 0.230 ppm; **UV/VIS** (CH_2Cl_2) λ_{max} (ε) [nm (l*mol^{-1}*cm^{-1})] = 276 (42104), 319 (44631), 384 (9004), 618 (593); **Elemental Analysis** calcd. for $C_{43}H_{41}AuClFN_2O_2$: C: 59.42, H: 4.75, N : 3.22, found C: 59.90, H: 5.21, N: 3.21.

Synthesis of (((2-(2-fluorophenyl)-1,3-bis(4-methoxyphenyl)azulen-4-yl)amino)(pyridin-2-ylamino)methylene)gold(I) chloride (14ag)

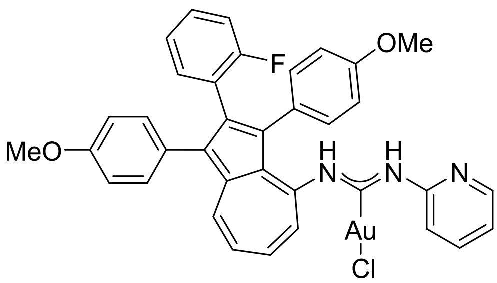

According to **GP1** 2-(2-fluorophenyl)-4-isocyano-1,3-bis(4-methoxyphenyl)azulene gold(I)chloride (**12a**) (50.0 mg, 72.3 µmol, 1.00 eq.) and pyridine-2-amine (**13g**) (7.14 mg, 75.9 µmol, 1.05 eq.) was stirred in dichloromethane at room temperature 12 hours. Purification by flash column chromatography (Silica, dichloromethane) yielded the title compound as a blue solid (36.0 mg, 48.8 µmol, 63%).

MP = 234 – 239 °C; **^{1}H{^{19}F} NMR** (301 MHz, CD_2Cl_2, 295 K) δ [ppm] = 13.82 (s, 1H, N$\underline{H}$), 9.70 (s, 1H, N$\underline{H}$), 8.28 (d, J = 9.5 Hz, 1H, $C_{Ar}\underline{H}$), 7.69 (d, J = 4.3 Hz, 1H, $C_{Ar}\underline{H}$), 7.65 – 7.44 (m, 3H, 3x$C_{Ar}\underline{H}$), 7.19 – 7.03 (m, 5H, 5x$C_{Ar}\underline{H}$), 6.94 – 6.82 (m, 4H, 4x$C_{Ar}\underline{H}$), 6.78 (d, J = 6.7 Hz, 4H, 4x$C_{Ar}\underline{H}$), 6.42 – 6.15 (m, 2H, 2x$C_{Ar}\underline{H}$), 3.72 (s, 3H, -OC$\underline{H_3}$), 3.26 (s, 3H, -OC$\underline{H_3}$); **^{13}C{^{19}F, ^{1}H} NMR** (101 MHz, CD_2Cl_2, 295 K) δ [ppm] = 190.33 (s, 1C, $\underline{C}_{Carbene}$), 158.98 (s, 1C, $\underline{C}_{Ar}$), 157.77 (s, 1C, $\underline{C}_{Ar}$), 154.23 (s, 1C, $\underline{C}_{Ar}$), 146.20 (d, 1C, $\underline{C}_{Ar}$H), 145.90 (s, 1C, $\underline{C}_{Ar}$), 145.03 (s, 1C, $\underline{C}_{Ar}$), 139.03 (s, 1C, $\underline{C}_{Ar}$), 138.91 (d, 1C, $\underline{C}_{Ar}$H), 137.21 (d, 1C, $\underline{C}_{Ar}$H), 136.49 (d, 1C, $\underline{C}_{Ar}$H), 132.99 (d, 1C, $\underline{C}_{Ar}$H), 132.96 (d, 1C, $\underline{C}_{Ar}$H), 132.40 (d, 1C, $\underline{C}_{Ar}$H), 132.26 (s, 1C, $\underline{C}_{Ar}$), 129.59 (s, 1C, $\underline{C}_{Ar}$), 129.33 (d, 1C, $\underline{C}_{Ar}$H), 129.25 (d, 1C, $\underline{C}_{Ar}$H), 128.66 (s, 1C, $\underline{C}_{Ar}$), 128.11 (s, 1C, $\underline{C}_{Ar}$), 127.58 (s, 1C, $\underline{C}_{Ar}$), 125.37 (s, 1C, $\underline{C}_{Ar}$), 125.21 (s, 1C, $\underline{C}_{Ar}$), 124.38 (d, 1C, $\underline{C}_{Ar}$H), 123.83 (d, 1C, $\underline{C}_{Ar}$H), 123.58 (d, 1C, $\underline{C}_{Ar}$H), 123.54 (d, 1C, $\underline{C}_{Ar}$H), 119.86 (d, 1C, $\underline{C}_{Ar}$H), 115.35 (d, 1C, $\underline{C}_{Ar}$H), 115.13 (d, 1C, $\underline{C}_{Ar}$H), 113.77 (d, 1C, $\underline{C}_{Ar}$H), 113.44 (d, 1C, $\underline{C}_{Ar}$H), 112.70 (d, 1C, $\underline{C}_{Ar}$H), 112.47 (d, 1C, $\underline{C}_{Ar}$H), 55.47 (q, 1C, -O$\underline{C}H_3$), 54.87 (q, 1C, -O$\underline{C}H_3$); **^{19}F{^{1}H} NMR** (471 MHz, CD_2Cl_2, 295 K) δ [ppm] = -113.45 (s, 1F, $C_{Ar}\underline{F}$); **IR** (ATR) $\tilde{\nu}$ [cm^{-1}] = 3236 (m), 3132 (m), 3120 (m), 3067 (w), 2965 (w), 2936 (w), 1710 (s), 1685 (m), 1605 (m), 1575 (m), 1457 (m), 1433 (m), 1362 (w), 1294 (m), 1267 (s), 1227 (s), 1174 (s), 1129 (s), 1102 (s), 1062 (s), 1028 (s), 882 (w), 828 (w), 788 (w), 730 (w), 682 (w), 624 (w); **HR-MS** (MALDI): $C_{36}H_{28}AuClFN_2O_2$ $[M]^+$ calcd.: 785.1520, found.: 785.1517, Δm/z = 0.382 ppm; **UV/VIS** (CH_2Cl_2) λ_{max} (ε) [nm ($l*mol^{-1}*cm^{-1}$)] = 263 (39406), 288 (45894), 319 (36926), 619 (696), 654 (661), 732 (276); **Elemental Analysis** calcd. for $C_{36}H_{28}AuClFN_2O_2$: C: 55.01, H: 3.59, N : 5.35, found C: 55.59, H: 3.83, N: 5.31.

Synthesis of (((2-(2-fluorophenyl)-1,3-bis(4-methoxyphenyl)azulen-4-yl)amino)(methyl(phenyl)amino)methylene)gold(I) chloride (14ah)

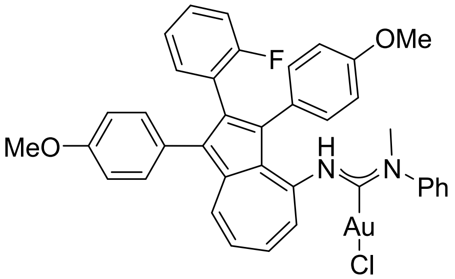

According to **GP1** 2-(2-fluorophenyl)-4-isocyano-1,3-bis(4-methoxyphenyl)azulene gold(I)chloride (**12a**) (50.0 mg, 72.3 µmol, 1.00 eq.) and *N*-methylaniline (**13h**) (8.13 mg, 75.9 µmol, 1.05 eq.) was stirred in dichloromethane at room temperature 12 hours. Purification by flash column chromatography (Silica, dichloromethane) yielded the title compound as a blue solid (41.0 mg, 51.3 µmol, 71%).

MP = 184 – 189 °C; Mixture of Rotamers :Rotamer A : Rotamer B = 1.19 : 1; **^{1}H{^{19}F} NMR** (500 MHz, C_6D_6, 295 K) δ [ppm] = 8.13 (dd, *J* = 9.6, 1.0 Hz, 1H, $N\underline{H}$, Rotamer B), 8.01 (dd, *J* = 9.6, 1.1 Hz, 1H, $N\underline{H}$, Rotamer A), 7.90 (d, *J* = 10.5 Hz, 1H, $C_{Ar}\underline{H}$, Rotamer B), 7.57 (d, *J* = 10.5 Hz, 1H, $C_{Ar}\underline{H}$, Rotamer A), 7.16 (d, *J* = 8.1 Hz, $C_{Ar}\underline{H}$, Rotamer A and Rotamer B), 7.09 (d, *J* = 3.3 Hz, 1H, $C_{Ar}\underline{H}$, Rotamer B), 7.06 (d, *J* = 12.9 Hz, 2H, 2x$C_{Ar}\underline{H}$, Rotamer A), 7.04 – 7.01 (m, 1H, $C_{Ar}\underline{H}$, Rotamer B), 7.00 (s, 1H, $C_{Ar}\underline{H}$, Rotamer B), 6.99 (s, 1H; $C_{Ar}\underline{H}$, Rotamer A), 6.88 (d, *J* = 11.3 Hz, 3H, 3x$C_{Ar}\underline{H}$ Rotamer A), 6.84 (d, *J* = 7.9 Hz, 1H, $C_{Ar}\underline{H}$ Rotamer B), 6.83 – 6.77 (m, 2H, 2x$C_{Ar}\underline{H}$, Rotamer A und B), 6.71 (dd, *J* = 8.4, 2.3 Hz, 1H, $C_{Ar}\underline{H}$, Rotamer B), 6.63 – 6.58 (m, 4H,4x$C_{Ar}\underline{H}$, Rotamer A), 6.55 (td, *J* = 7.6, 1.4 Hz, 6H, 6x$C_{Ar}\underline{H}$, Rotamer B), 6.52 – 6.51 (m, 1H, $C_{Ar}\underline{H}$, Rotamer A), 6.51 – 6.48 (m, 1H, $C_{Ar}\underline{H}$, Rotamer A), 6.48 – 6.46 (m, 1H, $C_{Ar}\underline{H}$, Rotamer B), 6.46 – 6.44 (m, 2H, 2x$C_{Ar}\underline{H}$, Rotamer A), 6.44 – 6.42 (m, 1H, $C_{Ar}\underline{H}$, Rotamer B), 6.39 (td, *J* = 7.4, 1.3 Hz, 1H, $C_{Ar}\underline{H}$, Rotamer B), 6.35 (dd, *J* = 8.4, 2.8 Hz, 1H, $C_{Ar}\underline{H}$, Rotamer A), 6.30 (dd, *J* = 8.5, 2.9 Hz, 1H, $C_{Ar}\underline{H}$, Rotamer B), 6.27 (dd, *J* = 8.5, 2.9 Hz, 1H, $C_{Ar}\underline{H}$, Rotamer B), 6.17 (dd, *J* = 8.5, 2.7 Hz, 1H, $C_{Ar}\underline{H}$, Rotamer A), 5.94 (d, *J* = 7.8 Hz, 1H, $C_{Ar}\underline{H}$, Rotamer A and Rotamer B), 3.14 (s, 3H, $-CH_3$, Rotamer A), 3.08 (s, 3H, $-OC\underline{H}_3$, Rotamer B), 3.06 (s, 6H, 2x$-OC\underline{H}_3$, Rotamer A), 2.94 (s, 3H, $-OC\underline{H}_3$, Rotamer B), 1.57 (s, 3H, $-C\underline{H}_3$, Rotamer B); **^{13}C{^{19}F, ^{1}H} NMR** (101 MHz, C_6D_6, 295 K) δ [ppm] = 194.04 (s, 1C, $\underline{C}_{Carbene}$, Rotamer A), 193.68 (s, 1C, $\underline{C}_{Carbene}$, Rotamer B), 161.98 (s, 1C, $\underline{C}_{Ar}$, Rotamer B), 161.90 (s, 1C, $\underline{C}_{Ar}$, Rotamer A), 159.53 (s, 1C, $\underline{C}_{Ar}$, Rotamer B), 159.45 (s, 1C, $\underline{C}_{Ar}$, Rotamer A), 159.31 (s, 2C, 2x$\underline{C}_{Ar}$, Rotamer A), 159.22 (s, 2C, 2x$\underline{C}_{Ar}$, Rotamer B), 158.99 (s, 2C, 2x$\underline{C}_{Ar}$, Rotamer B), 158.76 (s, 2C, 2x$\underline{C}_{Ar}$, Rotamer A), 148.68 (s, 1C, $\underline{C}_{Ar}$, Rotamer B), 145.68 (s, 1C, $\underline{C}_{Ar}$, Rotamer A), 145.43 (s, 1C, $\underline{C}_{Ar}$, Rotamer A), 144.26 (s, 1C, $\underline{C}_{Ar}$, Rotamer B), 144.06 (s, 2C, 2x$\underline{C}_{Ar}$, Rotamer B), 140.39 (s, 2C, 2x$\underline{C}_{Ar}$, Rotamer A), 139.31 (s, 1C, $\underline{C}_{Ar}$, Rotamer A), 139.12 (s, 1C, $\underline{C}_{Ar}$, Rotamer B), 137.48 (d, 1C, $\underline{C}_{Ar}$H, Rotamer B), 137.18 (d, 1C, $\underline{C}_{Ar}$H, Rotamer A), 136.69 (d, 1C, $\underline{C}_{Ar}$H, Rotamer B), 136.16 (d, 1C, $\underline{C}_{Ar}$H, Rotamer A), 134.67 (d, 1C, $\underline{C}_{Ar}$H, Rotamer B), 134.50 (d, 1C, $\underline{C}_{Ar}$H, Rotamer A), 133.40 (d, 1C, $\underline{C}_{Ar}$H, Rotamer A), 133.22 (d, 1C, $\underline{C}_{Ar}$H, Rotamer B), 133.10 (d, 1C, $\underline{C}_{Ar}$H, Rotamer B), 133.07 (d, 1C, $\underline{C}_{Ar}$H, Rotamer B), 133.04 (d, 2C, 2x$\underline{C}_{Ar}$H, Rotamer A), 133.00 (d, 2C, 2x$\underline{C}_{Ar}$H, Rotamer A), 132.60 (d, 1C, $\underline{C}_{Ar}$H, Rotamer A), 132.52 (d, 1C, $\underline{C}_{Ar}$H, Rotamer A), 132.45 (s, 1C, $\underline{C}_{Ar}$, Rotamer B), 132.38 (s, 1C, $\underline{C}_{Ar}$, Rotamer B), 130.35 (d, 2C, $\underline{C}_{Ar}$H, Rotamer A), 129.66 (d, 1C, $\underline{C}_{Ar}$H, Rotamer B), 129.56 (s, 1C, $\underline{C}_{Ar}$, Rotamer A), 129.40 (d, 1C, $\underline{C}_{Ar}$H, Rotamer B), 129.32 (d, 1C, $\underline{C}_{Ar}$H, Rotamer B), 129.19 (d, 1C, $\underline{C}_{Ar}$H, Rotamer A), 129.11 (d, 1C, $\underline{C}_{Ar}$H, Rotamer A), 128.81 (s, 1C, $\underline{C}_{Ar}$, Rotamer A), 128.59 (d, 2C, 2x$\underline{C}_{Ar}$H, Rotamer B), 127.02 (d, 1C, $\underline{C}_{Ar}$H, Rotamer B), 126.32 (d, 1C, $\underline{C}_{Ar}$H, Rotamer B), 125.68 (d, 1C, $\underline{C}_{Ar}$H, Rotamer A), 123.95 (d, 1C, $\underline{C}_{Ar}$H, Rotamer B),

123.91 (d, 1C, C<u>Ar</u>H, Rotamer A), 123.84(d, 1C, C<u>Ar</u>H, Rotamer A), 123.80 (d, 1C, C<u>Ar</u>H, Rotamer B), 123.64 (d, 1C, C<u>Ar</u>H, Rotamer B), 123.38 (d, 1C, C<u>Ar</u>H, Rotamer A), 115.76 (d, 1C, C<u>Ar</u>H, Rotamer A), 115.59 (d, 1C, C<u>Ar</u>H, Rotamer A), 115.54 (d, 1C, C<u>Ar</u>H, Rotamer B), 115.37 (d, 1C, C<u>Ar</u>H, Rotamer B), 114.26 (d, 1C, C<u>Ar</u>H, Rotamer B), 114.16 (d, 1C, C<u>Ar</u>H, Rotamer A), 113.66 (d, 1C, C<u>Ar</u>H, Rotamer B), 113.58 (d, 1C, C<u>Ar</u>H, Rotamer A), 113.40 (d, 1C, C<u>Ar</u>H, Rotamer B), 113.06 (d, 1C, C<u>Ar</u>H, Rotamer A), 54.98 (q, 1C, -O<u>C</u>H_3, Rotamer A), 54.88 (q, 1C, -O<u>C</u>H_3, Rotamer B), 54.76 (q, 1C, -O<u>C</u>H_3, Rotamer B), 54.72 (q, 1C, -O<u>C</u>H_3, Rotamer A), 48.68 (q, 1C, -<u>C</u>H_3, Rotamer A), 36.56 (q, 1C, -<u>C</u>H_3, Rotamer B); **^{19}F{^{1}H} NMR** (283 MHz, C_6D_6, 295 K) δ [ppm] = -112.04 (s, 1F, C_{Ar}<u>F</u>, Rotamer B), -112.08 (s, 1F, C_{Ar}<u>F</u>, Rotamer A); **IR** (ATR) $\tilde{\nu}$ [cm^{-1}] = 3320 (m), 2929 (w), 2855 (w), 1607 (m), 1539 (s), 1451 (m), 1393 (w), 1334 (m), 1247 (s), 1204 (s), 1149 (s), 1102 (s), 1029 (s), 980 (m), 889 (w), 789 (m), 729 (w), 694 (w), 614 (w); **HR-MS** (MALDI): $C_{38}H_{31}AuClFN_2O_2$ $[M]^+$ calcd.: 798.1724, found.: 798.1727, Δm/z = 0.376 ppm; **UV/VIS** (CH_2Cl_2) λ_{max} (ε) [nm (l*mol^{-1}*cm^{-1})] = 276 (48060), 320 (4950), 385 (7709), 613 (719), 651 (667), 723 (267); **Elemental Analysis** calcd. for $C_{38}H_{31}AuClFN_2O_2$: C: 57.12, H: 3.91, N : 3.51, found C: 57.14, H: 4.34, N: 3.35.

Synthesis of (((2-(2-fluorophenyl)-1,3-bis(4-methoxyphenyl)azulen-4-yl)amino)(isopropyl(phenyl)amino)methylene)gold(I) chloride (14ai)

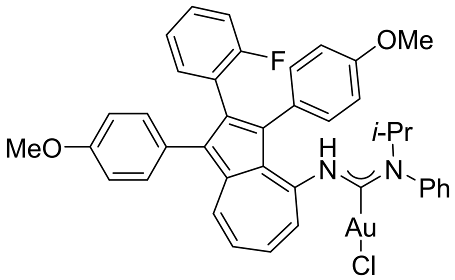

According to **GP1** 2-(2-fluorophenyl)-4-isocyano-1,3-bis(4-methoxyphenyl)azulene gold(I)chloride (**12a**) (50.0 mg, 72.3 μmol, 1.00 eq.) and *N*-isopropylaniline (**13i**) (10.3 mg, 75.9 μmol, 1.05 eq.) was stirred in dichloromethane at room temperature 12 hours. Purification by flash column chromatography (Silica, dichloromethane) yielded the title compound as a blue solid (41.0 mg, 47.2 μmol, 65%).

MP = 210 – 215 °C; **^{1}H{^{19}F} NMR** (500 MHz, C_6D_6, 295 K) δ [ppm] = 8.04 (dd, *J* = 9.6, 1.1 Hz, 1H, N<u>H</u>), 7.55 (d, *J* = 10.5 Hz, 1H, C_{Ar}<u>H</u>), 7.15 (d, *J* = 7.9 Hz, 2H, 2xC_{Ar}<u>H</u>), 7.06 (d, *J* = 9.2 Hz, 1H, C_{Ar}<u>H</u>), 6.89 (dd, *J* = 8.5, 2.3 Hz, 1H, C_{Ar}<u>H</u>), 6.79 (dd, *J* = 8.5, 2.4 Hz, 1H, C_{Ar}<u>H</u>), 6.68 (dd, *J* = 7.6, 1.9 Hz, 1H, C_{Ar}<u>H</u>), 6.66 – 6.59 (m, 5H, 5xC_{Ar}<u>H</u>), 6.60 – 6.55 (m, 3H, 3xC_{Ar}<u>H</u>), 6.54 – 6.49 (m, 1H, C_{Ar}<u>H</u>), 6.47 (ddt, *J* = 7.1, 4.6, 2.3 Hz, 3H, 3xC_{Ar}<u>H</u>), 6.39 (dd, *J* = 8.5, 2.7

Hz, 1H, $C_{Ar}\underline{H}$), 5.99 (dt, J = 6.4, 2.3 Hz, 1H, $C_{Ar}\underline{H}$), 5.69 (d, J = 7.4 Hz, 1H, $C_{Ar}\underline{H}$), 5.51 (p, J = 6.8 Hz, 1H, -C$\underline{H}$(CH$_3$)$_2$), 3.14 (s, 3H, -OC$\underline{H_3}$), 3.13 (s, 3H, -OC$\underline{H_3}$), 0.69 (d, J = 6.8 Hz, 3H, -CH(C$\underline{H_3}$)$_2$), 0.65 (d, J = 6.9 Hz, 3H, -CH(C$\underline{H_3}$)$_2$); **^{13}C{^{19}F, ^{1}H} NMR** (101 MHz, C_6D_6, 295 K) δ [ppm] = 194.01 (s, 1C, $\underline{C}_{Carbene}$), 161.97 (s, 1C, $\underline{C}_{Ar}$), 159.52 (s, 1C, $\underline{C}_{Ar}$), 159.22 (s, 1C, $\underline{C}_{Ar}$), 158.69 (s, 1C, $\underline{C}_{Ar}$), 145.55 (s, 1C, $\underline{C}_{Ar}$), 144.36 (s, 1C, $\underline{C}_{Ar}$), 139.63 (s, 1C, $\underline{C}_{Ar}$), 137.07 (d, 1C, $\underline{C}_{Ar}$H), 136.00 (d, 1C, $\underline{C}_{Ar}$H), 135.36 (s, 1C, $\underline{C}_{Ar}$), 134.92 (d, 1C, $\underline{C}_{Ar}$H), 133.88 (d, 1C, $\underline{C}_{Ar}$H), 133.10 (d, 1C, $\underline{C}_{Ar}$H), 133.06 (d, 1C, $\underline{C}_{Ar}$H), 132.51 (d, 1C, $\underline{C}_{Ar}$H), 132.34 (s, 1C, $\underline{C}_{Ar}$), 130.11 (d, 1C, $\underline{C}_{Ar}$H), 130.00 (d, 1C, $\underline{C}_{Ar}$H), 129.22 (d, 1C, $\underline{C}_{Ar}$H), 129.12 (d, 1C, $\underline{C}_{Ar}$H), 129.02 (s, 1C, $\underline{C}_{Ar}$), 128.83 (d, 1C, $\underline{C}_{Ar}$H), 128.80 (d, 1C, $\underline{C}_{Ar}$H), 126.28 (s, 1C, $\underline{C}_{Ar}$), 125.88 (s, 1C, $\underline{C}_{Ar}$), 125.72 (s, 1C, $\underline{C}_{Ar}$), 124.25 (d, 1C, $\underline{C}_{Ar}$H), 123.75 (d, 1C, $\underline{C}_{Ar}$H), 123.69 (d, 1C, $\underline{C}_{Ar}$H), 123.66 (d, 1C, $\underline{C}_{Ar}$H), 115.64 (d, 1C, $\underline{C}_{Ar}$H), 115.42 (d, 1C, $\underline{C}_{Ar}$H), 114.15 (d, 1C, $\underline{C}_{Ar}$H), 113.58 (d, 1C, $\underline{C}_{Ar}$H), 113.04 (d, 1C, $\underline{C}_{Ar}$H), 61.67 (q, 1C, -$\underline{C}$H(CH$_3$)$_2$), 54.98 (q, 1C, -O$\underline{C}$H$_3$), 54.71 (q, 1C, -O$\underline{C}$H$_3$), 21.59 (q, 1C, -CH($\underline{C}$H$_3$)$_2$), 21.29 (q, 1C, -CH($\underline{C}$H$_3$)$_2$); **^{19}F {^{1}H} NMR** (471 MHz, C_6D_6, 295 K) δ [ppm] = -112.04 (s, 1F, $C_{Ar}\underline{F}$); **IR** (ATR) $\tilde{\nu}$ [cm^{-1}] = 3339 (w), 2969 (w), 2930 (w), 1739 (w), 1609 (m), 1558 (s), 1510 (m), 1450 (m), 1347 (w), 1315 (m), 1283 (m), 1244 (s), 1209 (s), 1173 (w), 1108 (m), 1032 (m), 949 (w), 886 (m), 826 (m), 754 (w), 734 (m), 702 (m), 642 (w), 621 (w); **HR-MS** (MALDI): $C_{48}H_{35}AuClFN_2O_2$ [M]$^+$ calcd.: 826.2037, found.: 826.2038, Δm/z = 0.121 ppm; **UV/VIS** (CH_2Cl_2) λ_{max} (ε) [nm (l*mol^{-1}*cm^{-1})] = 277 (38600), 319 (3885), 383 (6793), 616 (737), 662 (662), 726 (283); **Elemental Analysis** calcd. for $C_{48}H_{35}AuClFN_2O_2$: C: 58.08, H: 4.27, N : 3.39, found C: 57.85, H: 4.66, N: 3.24.

Synthesis of ((diethylamino)((2-(4-fluorophenyl)-1,3-bis(4-methoxyphenyl)azulen-6-yl)amino)methylene)gold(I) chloride (14ba)

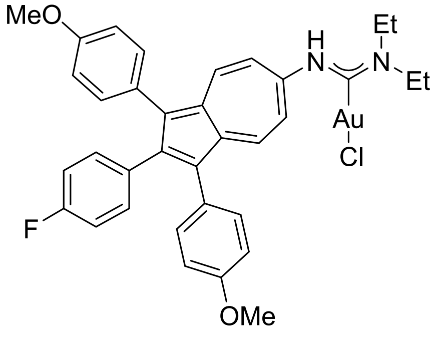

According to **GP1** 2-(4-fluorophenyl)-6-isocyano-1,3-bis(4-methoxyphenyl)azulene gold(I)chloride (**12b**) (50.0 mg, 72.3 µmol, 1.00 eq.) and diethylamine (**13a**) (5.55 mg, 75.9 µmol, 1.05 eq.) was stirred in dichloromethane at room temperature 12 hours. Purification by flash column chromatography (Silica, dichloromethane) yielded the title compound as a green solid (49.0 mg, 64.0 µmol, 88%).

According to **GP2** [AuCl(DMS)] (**11**) (52.6 mg, 178.6 µmol, 1.00 eq.) and 2-(4-fluorophenyl)-6-isocyano-1,3-bis(4-methoxyphenyl)azulene (**10b**) (82.0 mg, 178 µmol, 1.00 eq.) were stirred in dichloromethane for 1 hour at room temperature. Afterwards, diethylamine (**13a**) (13.7 mg, 187 µmol, 1.05 eq.) was added, the mixture stirred at room temperature for additional 12 hours. Purification by flash column chromatography (Silica, dichloromethane) yielded the title compound as a green solid (123 mg, 161 µmol, 90%).

MP = 213 – 218 °C; **^{1}H{^{19}F} NMR** (600 MHz, C_6D_6, 295 K) δ [ppm] = 8.34 (d, *J* = 11.0 Hz, 2H, 2x$C_{Ar}\underline{H}$), 8.06 (s, 1H, N$\underline{H}$), 7.27 (d, *J* = 8.8 Hz, 4H, 4x$C_{Ar}\underline{H}$), 7.23 (d, *J* = 11.0 Hz, 2H, 2x$C_{Ar}\underline{H}$), 7.14 – 7.09 (m, 2H, 2x$C_{Ar}\underline{H}$), 6.87 (d, *J* = 8.5 Hz, 4H, 4x$C_{Ar}\underline{H}$), 6.71 – 6.63 (m, 2H, 2x$C_{Ar}\underline{H}$), 3.38 (s, 6H, 2xOC$\underline{H_3}$), 3.38 – 3.33 (m, 2H, -C$\underline{H_2}$-), 2.72 (q, *J* = 7.4 Hz, 2H, -C$\underline{H_2}$-), 0.73 (t, *J* = 7.2 Hz, 3H, -C$\underline{H_3}$), 0.59 (t, *J* = 7.2 Hz, 3H, -C$\underline{H_3}$); **^{13}C{^{19}F, ^{1}H} NMR** (151 MHz, C_6D_6, 295 K) δ [ppm] = 190.67 (s, 1C, $\underline{C_{Carbene}}$), 163.16 (s, 2C, 2x$\underline{C_{Ar}}$), 161.52 (s, 1C, $\underline{C_{Ar}}$), 159.16 (s, 2C, 2x$\underline{C_{Ar}}$), 148.68 (s, 1C, $\underline{C_{Ar}}$), 146.66 (s, 1C, $\underline{C_{Ar}}$), 136.38 (s, 2C, 2x$\underline{C_{Ar}}$), 134.54 (d, 2C, 2x$\underline{C_{Ar}}$H), 133.46 (d, 2C, 2x$\underline{C_{Ar}}$H), 133.40 (d, 1C, $\underline{C_{Ar}}$H), 133.23 (d, 2C, 2x$\underline{C_{Ar}}$H), 132.93 (d, 1C, $\underline{C_{Ar}}$H), 130.71 (s, 1C, $\underline{C_{Ar}}$), 128.59 (s, 2C, 2x$\underline{C_{Ar}}$), 120.92 (d, 2C, 2x$\underline{C_{Ar}}$H), 115.41 (d, 2C, 2x$\underline{C_{Ar}}$H), 115.27 (d, 2C, 2x$\underline{C_{Ar}}$H), 114.46 (d, 2C, 2x$\underline{C_{Ar}}$H), 54.93 (q, 2C, 2x-O$\underline{C}H_3$), 54.20 (t, 1C, -$\underline{C}H_2$-), 42.21 (t, 1C, -$\underline{C}H_2$-), 14.40 (q, 1C, -$\underline{C}H_3$), 12.21 (q, 1C, -$\underline{C}H_3$); **^{19}F{^{1}H} NMR** (283 MHz, C_6D_6, 295 K) δ [ppm] = -114.74 (s, 1F, $C_{Ar}\underline{F}$); **IR** (ATR) $\tilde{\nu}$ [cm^{-1}] = 3249 (w), 2973 (w), 2931 (w), 2834 (w), 2038 (w), 1888 (w), 1736 (w), 1605 (m), 1573 (w), 1534 (s), 1517 (s), 1441(m), 1406 (w), 1386 (w), 1346 (w), 1324 (m), 1287 (m), 1243 (s), 1174 (s), 1157 (m), 1094 (w), 1031 (m), 952 (w), 826 (s), 793 (s), 737 (m), 665 (m); **HR-MS** (MALDI): $C_{35}H_{33}AuClFN_2O_2$ $[M]^+$ calcd.: 764.1880, found: 764.1875, Δm/z = 0.654 ppm ; **UV/VIS** (CH_2Cl_2) λ_{max} (ε) [nm (l*mol^{-1}*cm^{-1})] =335(609044), 386 (538576), 422 (548643), 490 (1440), 620 (221), 667 (292), 742 (242) ; **Elemental Analysis** calcd. for $C_{35}H_{33}AuClFN_2O_2$: C: 54.95, H: 4.35, N : 3.66, found C: 54.90, H: 4.33, N: 3.67.

Synthesis of (((2-(4-fluorophenyl)-1,3-bis(4-methoxyphenyl)azulen-6-yl)amino)(pyrrolidin-1-yl)methylene)gold(I) chloride (14bb)

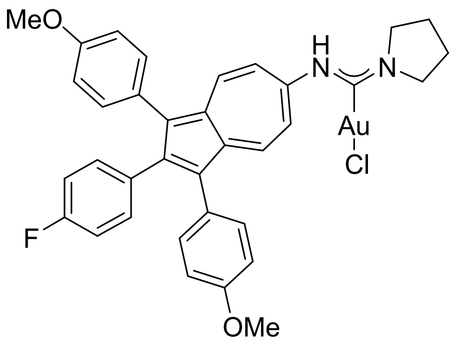

According to **GP1** 2-(4-fluorophenyl)-6-isocyano-1,3-bis(4-methoxyphenyl)azulene gold(I)chloride (**12b**) (50.0 mg, 72.3 µmol, 1.00 eq.) and pyrrolidine (**13b**) (5.40 mg, 75.9 µmol, 1.05 eq.) was stirred in dichloromethane at room temperature 12 hours. Purification by flash column chromatography (Silica, dichloromethane) yielded the title compound as a green solid (50.0 mg, 65.5 µmol, 91%).

MP = 186 – 191 °C;**^{1}H{^{19}F} NMR** (400 MHz, CD_2Cl_2, 295 K) δ [ppm] = 8.18 (d, *J* = 10.8 Hz, 2H, 2x$C_{Ar}\underline{H}$), 7.56 (s, 1H, N$\underline{H}$), 7.26 (d, *J* = 10.8 Hz, 2H, 2x$C_{Ar}\underline{H}$), 7.17 (d, *J* = 8.8 Hz, 4H, 4x$C_{Ar}\underline{H}$), 7.03 (dd, *J* = 8.9, 5.6 Hz, 2H, 2x$C_{Ar}\underline{H}$), 6.92 (d, *J* = 8.9 Hz, 4H, 4x$C_{Ar}\underline{H}$), 6.85 (t, *J* = 8.9 Hz, 2H, -C$\underline{H_2}$-), 4.07 (t, *J* = 6.9 Hz, 2H, -C$\underline{H_2}$-), 3.83 (s, 6H, 2x-OC$\underline{H_3}$), 3.41 (t, *J* = 7.1 Hz, 2H, -C$\underline{H_2}$-), 2.23 – 2.11 (m, 2H, -C$\underline{H_2}$-), 1.97 (p, *J* = 7.0 Hz, 2H, -C$\underline{H_2}$-); **^{13}C{^{19}F, ^{1}H} NMR** (101 MHz, CD_2Cl_2, 295 K) δ [ppm] = 189.04 (s, 1C, $\underline{C_{Carbene}}$), 159.05 (s, 2C, 2x$\underline{C_{Ar}}$), 148.05 (s, 2C, 2x$\underline{C_{Ar}}$), 147.07 (s, 2C, 2x$\underline{C_{Ar}}$), 136.01 (s, 2C, 2x$\underline{C_{Ar}}$), 134.67 (d, 1C, $\underline{C_{Ar}}$H), 133.32 (d, 1C, $\underline{C_{Ar}}$H), 133.24 (d, 2C, 2x$\underline{C_{Ar}}$H), 132.85 (d, 2C, 2x$\underline{C_{Ar}}$H), 131.03 (s, 2C, 2x$\underline{C_{Ar}}$), 128.31 (s, 2C, 2x$\underline{C_{Ar}}$), 119.61 (d, 2C, 2x$\underline{C_{Ar}}$H), 115.12 (d, 2C, 2x$\underline{C_{Ar}}$H), 114.91 (d, 2C, 2x$\underline{C_{Ar}}$H), 114.13 (d, 2C, 2x$\underline{C_{Ar}}$H), 57.52 (t, 1C, -$\underline{C}H_2$-), 55.65 (q, 2C, 2x-O$\underline{C}H_3$), 46.81 (t, 1C, -$\underline{C}H_2$-), 25.83 (t, 1C, -$\underline{C}H_2$-), 24.67 (t, 1C, -$\underline{C}H_2$-); **^{19}F{^{1}H} NMR** (283 MHz, CD_2Cl_2, 298 K) δ [ppm] = -116.21 (s, 1F, $C_{Ar}\underline{F}$); **IR** (ATR) $\tilde{\nu}$ [cm^{-1}] = 3352 (w), 2969 (w), 2929 (w), 2871 (w), 2834 (w), 1604 (m), 1573 (w), 1541 (s), 1515 (s), 1442 (m), 1405 (w), 1387 (w), 1351 (m), 1310 (m), 1287 (m), 1238 (s), 1175 (s), 1158 (m), 1109 (w), 1025 (m), 915 (w), 828 (m), 792 (s), 663 (w), 629 (w); **UV/VIS** (CH_2Cl_2) λ_{max} (ε) [nm (l*mol^{-1}*cm^{-1})] = 336 (65237), 390 (9576), 420 (5862), 620 (332), 660 (298), 746 (97); **HR-MS** (MALDI): $C_{35}H_{31}Au^{35}ClFN_2O_2$ $[M]^+$ calcd.: 762.1724, found.: 762.1713, $C_{35}H_{31}Au^{37}ClFN_2O_2$ $[M]^+$ calcd.: 764.1697, found.: 764.1698 , Δm/z = 0.131 ppm; **Elemental Analysis** calcd. for $C_{35}H_{31}AuClFN_2O_2$: C: 55.09, H: 4.10, N : 3.67, found C: 54.65, H: 4.45, N: 3.43.

Synthesis of (((2-(4-fluorophenyl)-1,3-bis(4-methoxyphenyl)azulen-6-yl)amino)(piperidin-1-yl)methylene)gold(I) chloride (14bc)

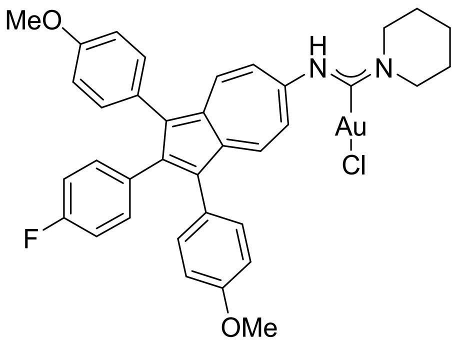

According to **GP1** 2-(4-fluorophenyl)-6-isocyano-1,3-bis(4-methoxyphenyl)azulene gold(I)chloride (**12b**) (50.0 mg, 72.3 µmol, 1.00 eq.) and piperidine (**13c**) (6.46 mg, 75.9 µmol, 1.05 eq.) was stirred in dichloromethane at room temperature 12 hours. Purification by flash column chromatography (Silica, dichloromethane) yielded the title compound as a green solid (49.0 mg, 63.1 µmol, 87%).

According to **GP2** [AuCl(DMS)] (**11**) (52.6 mg, 178.6 µmol, 1.00 eq.) and 2-(4-fluorophenyl)-6-isocyano-1,3-bis(4-methoxyphenyl)azulene (**10b**) (82.0 mg, 178 µmol, 1.00 eq.) were stirred in dichloromethane for 1 hour at room temperature. Afterwards, piperidine (**13c**) (16.0 mg, 187 µmol, 1.05 eq.) was added, the mixture stirred at room temperature for additional 12 hours. Purification by flash column chromatography (Silica, dichloromethane) yielded the title compound as a green solid (115 mg, 148 µmol, 83%).

MP = 174 – 179 °C; **^{1}H{^{19}F} NMR** (500 MHz, THF-d$_8$, 295 K) δ [ppm] = 9.23 (s, 1H, N<u>H</u>), 8.05 (d, *J* = 10.9 Hz, 2H, 2xC$_{Ar}$<u>H</u>), 7.24 – 7.13 (m, 2H, 2xC$_{Ar}$<u>H</u>), 7.04 (d, *J* = 8.7 Hz, 4H, 4xC$_{Ar}$<u>H</u>), 6.91 (dd, *J* = 8.9, 5.6 Hz, 2H, 2xC$_{Ar}$<u>H</u>), 6.80 (d, *J* = 8.8 Hz, 4H, 4xC$_{Ar}$<u>H</u>), 6.74 (t, *J* = 8.9 Hz, 2H, 2xC$_{Ar}$<u>H</u>), 4.04 – 4.01 (m, 2H, -C<u>H</u>$_2$-), 3.68 (s, 6H, 2x-OCH$_3$), 3.53 – 3.48 (m, 2H, -C<u>H</u>$_2$-), 1.52 (p, *J* = 2.9 Hz, 4H, 2x-C<u>H</u>$_2$-), 1.46 (q, *J* = 5.9 Hz, 2H, -C<u>H</u>$_2$-); **^{13}C{^{19}F, ^{1}H} NMR** (126 MHz, THF-d$_8$, 295 K) δ [ppm] = 190.47 (s, 1C, C$_{Carbene}$), 162.66 (s, 1C, C$_{Ar}$), 160.71 (s, 1C, C$_{Ar}$), 158.65 (s, 2C, 2xC$_{Ar}$), 149.56 (s, 1C, C$_{Ar}$), 145.51 (s, 1C, C$_{Ar}$), 135.55 (s, 1C, C$_{Ar}$), 133.96 (d, 2C, 2xC$_{Ar}$H), 133.13 (s, 2C, 2xC$_{Ar}$), 133.10 (s, 1C, C$_{Ar}$), 132.85 (d, 1C, C$_{Ar}$H), 132.79 (d, 1C, C$_{Ar}$H), 132.26 (d, 2C, 2xC$_{Ar}$H), 132.21 (d, 1C, C$_{Ar}$H), 129.72 (s, 1C, C$_{Ar}$), 128.07 (d, 1C, C$_{Ar}$H), 128.01 (s, 1C, C$_{Ar}$), 120.01 (d, 2C, 2xC$_{Ar}$H), 114.50 (d, 2C, 2xC$_{Ar}$H), 114.33 (d, 2C, 2xC$_{Ar}$H), 113.56 (d, 2C, 2xC$_{Ar}$H), 58.55 (t, 1C, -<u>C</u>H$_2$-), 54.38 (q, 2C, -O<u>C</u>H$_3$), 46.53 (t, 1C, -<u>C</u>H$_2$-), 26.51 (t, 1C, -<u>C</u>H$_2$-), 25.75 (t, 1C, -<u>C</u>H$_2$-), 24.84 (t, 1C, -<u>C</u>H$_2$-); **^{19}F{^{1}H} NMR** (471 MHz, THF-d$_8$, 295 K) δ [ppm] = -116.85 (s, 1F, C$_{Ar}$F); **IR** (ATR) $\tilde{\nu}$ [cm^{-1}] = 3261 (w), 3059 (w), 3021 (w), 2943 (w), 2856 (w), 2836 (w), 1605 (m), 1574 (m), 1551(m), 1538 (s), 1516 (s), 1473 (s), 1441 (m), 1405 (m), 1386 (m), 1350 (m), 1318 (w), 1286 (m), 1245(m), 1219 (s), 1175 (s), 1156(s), 1108 (m), 1089 (w), 1021 (s), 945 (w), 877 (w), 852 (m), 833 (m), 816 (m), 793 (s), 731 (s), 702 (w), 666 (m), 644 (m); **HR-MS** (MALDI): $C_{36}H_{33}AuClFN_2O_2$ [M]$^+$ calcd.:776.1880, found.:776.1884, Δm/z = 0.515 ppm; **UV/VIS** (CH_2Cl_2) λ_{max} (ε) [nm (l*mol^{-1}*cm^{-1})] = 257 (1817), 338 (5028), 382 (788), 416 (468), 618 (251), 666 (224), 750 (82) ; **Elemental Analysis** calcd. for $C_{36}H_{33}AuClFN_2O_2$: C: 55.64, H: 4.28, N : 3.61, found C: 55.20, H: 4.72, N: 3.22.

Synthesis of (((2,6-dimethylphenyl)amino)((2-(4-fluorophenyl)-1,3-bis(4-methoxyphenyl)azulen-6-yl)amino)methylene)gold(I) chloride (14bd)

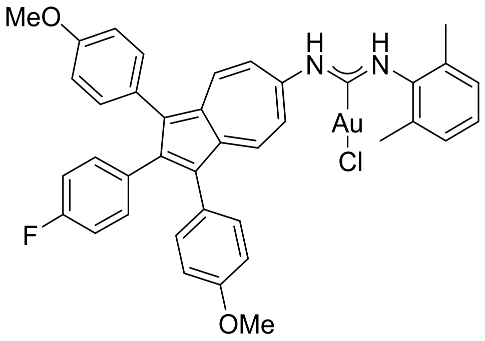

According to **GP1** 2-(4-fluorophenyl)-6-isocyano-1,3-bis(4-methoxyphenyl)azulene gold(I)chloride (**12b**) (50.0 mg, 72.3 µmol, 1.00 eq.) and 2,6-dimethylaniline (**13d**) (9.19 mg, 75.9 µmol, 1.05 eq.) was stirred in dichloromethane at room temperature 12 hours. Purification by flash column chromatography (Silica, dichloromethane) yielded the title compound as a blue solid (42.0 mg, 51.7 µmol, 71%).

MP = 220 – 225 °C;**^{1}H{^{19}F} NMR** (600 MHz, CD_2Cl_2, 295 K) δ [ppm] = 8.19 (d, *J* = 11.0 Hz, 2H, 2x$C_{Ar}\underline{H}$), 7.94 (s, 1H, $C_{Ar}\underline{H}$), 7.20 (s, 1H, $C_{Ar}\underline{H}$), 7.18 (d, *J* = 8.5 Hz, 6H, 6x$C_{Ar}\underline{H}$), 7.02 (dd, *J* = 8.8, 5.8 Hz, 2H, 2x$C_{Ar}\underline{H}$), 6.91 (d, *J* = 8.8 Hz, 5H, 5x$C_{Ar}\underline{H}$), 6.88 – 6.84 (m, 2H, 2x$C_{Ar}\underline{H}$), 3.83 (s, 6H, 2x-OC$\underline{H_3}$), 1.56 (s, 3H, -C$\underline{H_3}$), 1.55 (s, 3H, -C$\underline{H_3}$); **^{13}C{^{19}F, ^{1}H} NMR** (151 MHz, CD_2Cl_2, 295 K) δ [ppm] = 191.59 (s, 1C, $\underline{C_{Carbene}}$), 162.97 (s, 1C, $\underline{C_{Ar}}$), 161.34 (s, 2C, 2x$\underline{C_{Ar}}$), 158.95 (s, 4C, 4x$\underline{C_{Ar}}$), 148.28 (s, 2C, 2x$\underline{C_{Ar}}$), 147.23 (s, 2C, 2x$\underline{C_{Ar}}$), 136.10 (s, 2C, 2x$\underline{C_{Ar}}$), 134.49 (d, 2C, 2x$\underline{C_{Ar}}$H), 133.26 (d, 2C, 2x$\underline{C_{Ar}}$H), 133.21 (d, 1C, $\underline{C_{Ar}}$H), 132.81 (d, 4C, 4x$\underline{C_{Ar}}$H), 130.93 (s, 1C, $\underline{C_{Ar}}$), 128.18 (s, 1C, $\underline{C_{Ar}}$), 120.43 (d, 2C, 2x$\underline{C_{Ar}}$H), 115.06 (d, 2C, 2x$\underline{C_{Ar}}$H), 114.92 (d, 2C, 2x$\underline{C_{Ar}}$H), 114.05 (d, 4C, 4x$\underline{C_{Ar}}$H), 55.59 (q, 2C, 2x-OC$\underline{H_3}$), 21.05 (q, 2C, 2x-C$\underline{H_3}$); **^{19}F{^{1}H} NMR** (283 MHz, CD_2Cl_2, 295 K) δ [ppm]= -116.18 (s, 1F, $C_{Ar}\underline{F}$); **IR** (ATR) $\tilde{\nu}$ [cm^{-1}] = 3302 (w), 3188 (w), 2955 (w), 2928 (w), 2834 (w), 1606 (m), 1536 (s), 1517 (s), 1496 (s), 1470 (m), 1441 (m), 1389 (m), 1347 (m), 1287 (w), 1242 (m), 1195 (s), 1173 (w), 1155 (m), 1106 (w), 1090 (w), 1031(m), 952 (w), 818 (m), 792 (m), 772 (m), 736 (m), 654 (w); **HR-MS** (MALDI): $C_{39}H_{33}AuClFN_2O_2$ $[M+H]^+$ calcd.: 812.1882, found: 812.1880, Δm/z = 0.246 ppm; **UV/VIS** (CH_2Cl_2) λ_{max} (ε) [nm (l*mol^{-1}*cm^{-1})] = 270 (28233), 333 (65501), 388 (9486), 425 (4517), 625 (325), 677 (271) ; **Elemental Analysis** calcd. for $C_{39}H_{33}AuClFN_2O_2$: C: 57.61, H: 4.09, N : 3.45, found C: 56.37, H: 4.20, N: 3.34.

Synthesis of (((2-(4-fluorophenyl)-1,3-bis(4-methoxyphenyl)azulen-6-yl)amino)(mesitylamino)methylene)gold(I) chloride (14be)

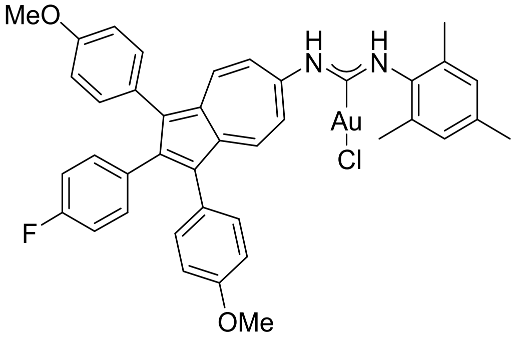

According to **GP1** 2-(4-fluorophenyl)-6-isocyano-1,3-bis(4-methoxyphenyl)azulene gold(I)chloride (**12d**) (50.0 mg, 72.3 µmol, 1.00 eq.) and mesitylamine (**13e**) (10.2 mg, 75.9 µmol, 1.05 eq.) was stirred in dichloromethane at room temperature 12 hours. Purification by flash column chromatography (Silica, dichloromethane) yielded the title compound as a blue solid (44.0 mg, 53.2 µmol, 74%).

MP = 204 – 209 °C;**^{1}H{^{19}F} NMR** (500 MHz, CD_2Cl_2, 295 K) δ 8.86 (s, 1H, N$\underline{H}$), 8.15 (d, *J* = 10.9 Hz, 2H, 2x$C_{Ar}\underline{H}$), 7.89 (s, 1H, N$\underline{H}$), 7.17 – 7.13 (m, 6H, 6x$C_{Ar}\underline{H}$), 7.03 – 7.00 (m, 4H, 4x$C_{Ar}\underline{H}$), 6.91 (d, *J* = 8.7 Hz, 4H, 4x$C_{Ar}\underline{H}$), 6.85 (d, *J* = 8.8 Hz, 2H, 2x$C_{Ar}\underline{H}$), 3.83 (s, 3H, -O$C\underline{H}_3$), 2.32 (s, 3H, -$C\underline{H}_3$), 2.26 (s, 6H, 2x-$C\underline{H}_3$);**^{13}C{^{19}F, ^{1}H} NMR** (126 MHz, CD_2Cl_2, 295 K) δ [ppm] = 190.16 (s, 1C, $C_{Carbene}$), 162.18 (s, 2C, 2x$\underline{C}_{Ar}$), 158.98 (s, 2C, 2x$\underline{C}_{Ar}$), 147.37 (s, 2C, 2x$\underline{C}_{Ar}$), 147.11 (s, 2C, 2x$\underline{C}_{Ar}$), 140.36 (s, 2C, 2x$\underline{C}_{Ar}$), 136.14 (s, 2C, 2x$\underline{C}_{Ar}$), 135.85 (s, 2C, 2x$\underline{C}_{Ar}$), 134.48 (d, 2C, 2x$\underline{C}_{Ar}$H), 133.23 (s, 1C, $\underline{C}_{Ar}$), 132.98 (d, 2C, 2x$\underline{C}_{Ar}$), 132.79 (d, 2C, 2x$\underline{C}_{Ar}$H), 131.08 (s, 1C, $\underline{C}_{Ar}$), 130.48 (d, 2C, 2x$\underline{C}_{Ar}$H), 129.69 (d, 2C, 2x$\underline{C}_{Ar}$H), 128.08 (d, 2C, 2x$\underline{C}_{Ar}$H), 119.57 (d, 2C, 2x$\underline{C}_{Ar}$H), 115.01 (d, 2C, 2x$\underline{C}_{Ar}$H), 114.06 (d, 2C, 2x$\underline{C}_{Ar}$H), 55.59 (q, 2C, 2x-O$\underline{C}H_3$);, 21.19 (q, 1C, -$\underline{C}H_3$), 18.52 (q, 2C, 2x-$\underline{C}H_3$);**^{19}F{^{1}H} NMR** (283 MHz, CD_2Cl_2, 298 K) δ [ppm] = -116.01 (s, 1F, -$C_{Ar}\underline{F}$); **IR** (ATR) $\tilde{\nu}$ [cm^{-1}] = 3201 (w), 2932 (w), 2833 (w), 1606 (m), 1516 (s), 1441 (m), 1388 (m), 1286 (m), 1242 (s), 1173 (s), 1156 (m), 1106 (w), 1031 (m), 833 (m), 792 (m); **UV/VIS** (CH_2Cl_2) λ_{max} (ε) [nm (l*mol^{-1}*cm^{-1})] = 334 (61439), 390 (9378), 414 (5659), 624 (329), 672 (291), 767 (81); **HR-MS** (MALDI): $C_{40}H_{35}Au^{35}ClFN_2O_2$ $[M]^+$ calcd.: 826.2037, found.: 826.2017, Δm/z = 2.421 ppm, $C_{40}H_{35}Au^{37}ClFN_2O_2$ $[M]^+$ calcd.: 828.2007, found.: 828.2010, Δm/z = 0.362 ppm; **Elemental Analysis** calcd. for $C_{40}H_{35}AuClFN_2O_2$: C: 58.08, H: 4.27, N : 3.39, found C: 58.69, H: 4.45, N: 3.51.

Synthesis of (((2,6-diisopropylphenyl)amino)((2-(4-fluorophenyl)-1,3-bis(4-methoxyphenyl)azulen-6-yl)amino)methylene)gold(I) chloride (14bf)

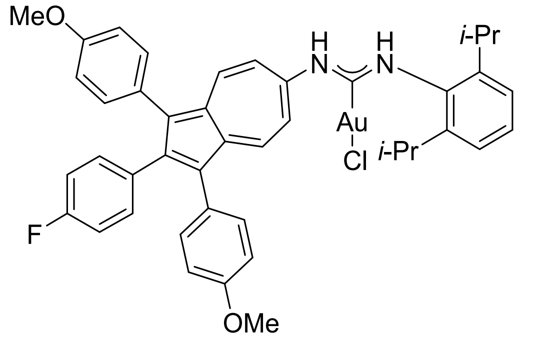

According to **GP1** 2-(4-fluorophenyl)-6-isocyano-1,3-bis(4-methoxyphenyl)azulene gold(I)chloride (**12b**) (50.0 mg, 72.3 µmol, 1.00 eq.) and 2,6-diisopropylaniline (**13f**) (13.5 mg, 75.9 µmol, 1.05 eq.) was stirred in dichloromethane at room temperature 12 hours. Purification by flash column chromatography (Silica, dichloromethane) yielded the title compound as a blue solid (43.0 mg, 49.5 µmol, 68%).

According to **GP2** [AuCl(DMS)] (**11**) (52.6 mg, 178.6 µmol, 1.00 eq.) and 2-(4-fluorophenyl)-6-isocyano-1,3-bis(4-methoxyphenyl)azulene (**10b**) (82.0 mg, 178 µmol, 1.00 eq.) were stirred in dichloromethane for 1 hour at room temperature. Afterwards, 2,6-diisopropylaniline (**13f**) (47.5 mg, 179 µmol, 1.50 eq.) was added, the mixture stirred at room temperature for additional 12 hours. Purification by flash column chromatography (Silica, dichloromethane) yielded the title compound as a green solid (86.0 mg, 111 µmol, 62%).

MP = 184 – 189 °C;**^{1}H{^{19}F} NMR** (500 MHz, CD_2Cl_2, 295 K) δ [ppm] = 9.38 (s, 1H, N$\underline{H}$), 8.18 – 8.12 (m, 2H, 2x$C_{Ar}\underline{H}$), 7.83 (s, 1H, N$\underline{H}$), 7.46 (t, *J* = 7.8 Hz, 1H, $C_{Ar}\underline{H}$), 7.33 (d, *J* = 7.8 Hz, 2H, 2x$C_{Ar}\underline{H}$), 7.19 – 7.10 (m, 6H, 6x$C_{Ar}\underline{H}$), 7.05 – 6.98 (m, 2H, 2x$C_{Ar}\underline{H}$), 6.90 (d, *J* = 8.8 Hz, 4H, 4x$C_{Ar}\underline{H}$), 6.85 (t, *J* = 8.9 Hz, 2H, 2x$C_{Ar}\underline{H}$), 3.82 (s, 6H, 2x-OC$\underline{H_3}$), 3.19 (hept, *J* = 6.9 Hz, 2H, -2x-C$\underline{H}(CH_3)_2$), 1.25 (d, *J* = 7.0 Hz, 6H, -CH$(\underline{CH_3})_2$), 1.16 (d, *J* = 6.9 Hz, 6H, -CH$(\underline{CH_3})_2$); **^{13}C{^{19}F, ^{1}H} NMR** (126 MHz, CD_2Cl_2, 295 K) δ [ppm] = 190.53 (s, 1C; $\underline{C}_{Carbene}$), 163.16 (s, 1C, $\underline{C}_{Ar}$), 161.20 (s, 1C, $\underline{C}_{Ar}$), 158.99 (s, 2C, 2x$\underline{C}_{Ar}$), 147.42 (s, 1C, $\underline{C}_{Ar}$), 147.01 (s, 1C, $\underline{C}_{Ar}$), 146.61 (s, 2C, 2x$\underline{C}_{Ar}$), 136.18 (s, 2C, 2x$\underline{C}_{Ar}$), 134.55 (d, 1C, $\underline{C}_{Ar}$H), 133.26 (d, 1C, $\underline{C}_{Ar}$H), 133.20 (d, 1C, $\underline{C}_{Ar}$H), 132.76 (d, 4C, 4x$\underline{C}_{Ar}$H), 131.14 (s, 2C, 2x$\underline{C}_{Ar}$), 131.04 (d, 1C, $\underline{C}_{Ar}$H), 129.67 (s, 2C, 2x$\underline{C}_{Ar}$), 128.05 (s, 1C, $\underline{C}_{Ar}$), 125.43 (d, 2C, 2x$\underline{C}_{Ar}$H), 119.52 (d, 2C, 2x$\underline{C}_{Ar}$H), 115.09 (d, 2C, 2x$\underline{C}_{Ar}$H), 114.92 (d, 1C, $\underline{C}_{Ar}$H), 114.06 (d, 4C, 4x$\underline{C}_{Ar}$H), 55.60 (q, 2C, 2x-OC$\underline{H_3}$), 29.35 (d, 2C, 2x-$\underline{C}H(CH_3)_2$), 24.58 (q, 2C, 2x-$\underline{C}H(CH_3)_2$), 23.45 (q, 2C, 2x-$\underline{C}H(CH_3)_2$); **^{19}F{^{1}H} NMR** (283 MHz, CD_2Cl_2, 298 K) δ [ppm] = -115.99 (s, 1F, -$C_{Ar}\underline{F}$); **IR**

(ATR) $\tilde{\nu}$ [cm-1] = 3203, 2963, 2933, 2871, 2836, 1662, 1606, 1573, 1518, 1463, 1443, 1386, 1287, 1243, 1175, 1156, 1106, 1059, 1032, 952, 936, 827, 793, 754, 662; **HR-MS** (MALDI):$C_{43}H_{41}AuClFN_2O_2$ $[M]^+$ calcd.: 868.2506, found.: 868.2519, Δm/z = 1.497 ppm; **UV/VIS** (CH_2Cl_2) λ_{max} (ε) [nm ($l*mol^{-1}*cm^{-1}$)] = 264 (17068), 333 (37476), 383 (6679), 417 (3636), 494 (249), 625 (217), 678 (186) ; **Elemental Analysis** calcd. for $C_{43}H_{41}AuClFN_2O_2$: C: 59.42, H: 4.75, N : 3.22, found C: 59.27, H: 5.29, N: 3.14.

Synthesis of (((2-(4-fluorophenyl)-1,3-bis(4-methoxyphenyl)azulen-6-yl)amino)(pyridin-2-ylamino)methylene)gold(I) chloride (14bg)

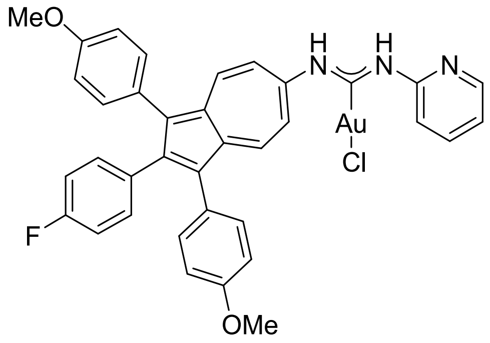

According to **GP1** 2-(4-fluorophenyl)-6-isocyano-1,3-bis(4-methoxyphenyl)azulene gold(I)chloride (**12b**) (50.0 mg, 72.3 μmol, 1.00 eq.) and pyridine-2-amine (**13g**) (7.14 mg, 75.9 μmol, 1.05 eq.) was stirred in dichloromethane at room temperature 12 hours. Purification by flash column chromatography (Silica, dichloromethane) yielded the title compound as a blue solid (44.0 mg, 56.0 μmol, 77%).

MP = 252 – 257 °C;**^{1}H{^{19}F} NMR** (500 MHz, CD_2Cl_2, 295 K) δ [ppm] = 14.79 (s, 1H, N<u>H</u>), 10.25 (s, 1H, N<u>H</u>), 8.28 (d, *J* = 3.2 Hz, 1H, $C_{Ar}\underline{H}$), 8.18 (d, *J* = 10.8 Hz, 2H, 2x$C_{Ar}\underline{H}$), 7.71 – 7.64 (m, 1H, $C_{Ar}\underline{H}$), 7.38 (d, *J* = 11.0 Hz, 2H, 2x$C_{Ar}\underline{H}$), 7.27 (d, *J* = 8.2 Hz, 1H, $C_{Ar}\underline{H}$), 7.14 (d, *J* = 8.7 Hz, 5H, 5x$C_{Ar}\underline{H}$), 7.03 – 6.98 (m, 2H, 2x$C_{Ar}\underline{H}$), 6.90 (d, *J* = 8.8 Hz, 4H, 4x$C_{Ar}\underline{H}$), 6.86 (t, *J* = 8.9 Hz, 2H, 2x$C_{Ar}\underline{H}$), 3.82 (s, 6H, 2x-O$C\underline{H}_3$); **^{13}C{^{19}F, ^{1}H} NMR** (126 MHz, CD_2Cl_2, 295 K) δ [ppm] = 189.82 (s, 1C, $\underline{C}_{Carbene}$), 163.15 (s, 1C, $\underline{C}_{Ar}$), 161.19 (s, 1C, $\underline{C}_{Ar}$), 158.94 (s, 2C, 2x$\underline{C}_{Ar}$), 154.82 (s, 2C, 2x$\underline{C}_{Ar}$), 148.12 (s, 1C, $\underline{C}_{Ar}$), 147.22 (s, 2C, 2x$\underline{C}_{Ar}$), 146.09 (d, 1C, $\underline{C}_{Ar}$H), 139.69 (d, 1C, $\underline{C}_{Ar}$H), 136.13 (s, 2C, 2x$\underline{C}_{Ar}$), 134.68 (d, 2C, 2x$\underline{C}_{Ar}$H), 133.28 (d, 1C, $\underline{C}_{Ar}$H), 133.22 (d, 4C, 4x$\underline{C}_{Ar}$H), 132.80 (d, 1C, $\underline{C}_{Ar}$H), 130.81 (s, 1C, $\underline{C}_{Ar}$), 128.14 (s, 1C, $\underline{C}_{Ar}$), 120.75 (d, 1C, $\underline{C}_{Ar}$H), 119.59 (d, 2C, 2x$\underline{C}_{Ar}$H), 115.10 (d, 1C, $\underline{C}_{Ar}$H), 114.93 (d, 1C, $\underline{C}_{Ar}$H), 114.77 (d, 1C, $\underline{C}_{Ar}$H), 114.07 (d, 4C, 4x$\underline{C}_{Ar}$H), 55.59 (q, 2C, 2x-O$\underline{C}H_3$); **^{19}F{^{1}H} NMR** (283 MHz, CD_2Cl_2, 295 K) δ [ppm] = -116.06 (s, 1F, -$C_{Ar}\underline{F}$); **IR** (ATR) $\tilde{\nu}$ [cm^{-1}] = 3249 (w), 3188 (w), 3116 (w), 3053 (w), 2931 (w), 2833 (w), 1734 (w), 1605 (m), 1515 (s), 1476 (m), 1430 (s),

1371 (m), 1320 (w), 1286 (m), 1242 (s), 1173 (s), 1154 (m), 1106 (w), 1030 (m), 826 (s), 776 (s), 734 (m), 633 (w); **UV/VIS** (CH_2Cl_2) λ_{max} (ε) [nm (l*mol^{-1}*cm^{-1})] = 285 (34968), 342 (64717), 422 (8037), 626 (355), 667 (319), 757 (102); **HR-MS** (MALDI): $C_{36}H_{28}Au^{35}ClFN_3O_2$ $[M]^+$ calcd.: 785.1520, found.: 785.1502, Δm/z = 2.292 ppm , $C_{36}H_{28}Au^{37}ClFN_3O_2$ $[M]^+$ calcd.: 787.1490, found.: 787.1488, Δm/z = 0.254 ppm; **Elemental Analysis** calcd. for $C_{36}H_{28}AuClFN_2O_2$: C: 55.63, H: 4.13, N : 5.27, found C: 55.59, H: 3.83, N: 5.31.

Synthesis of (((2-(4-fluorophenyl)-1,3-bis(4-methoxyphenyl)azulen-6-yl)amino)(methyl(phenyl)amino)methylene)gold(I) chloride (14bh)

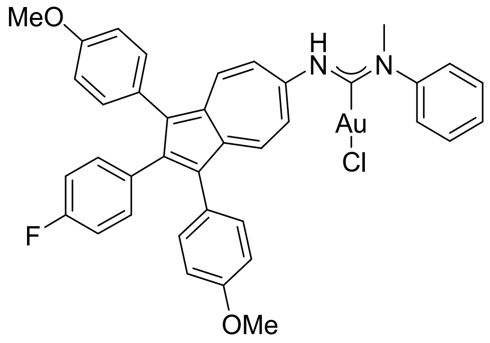

According to **GP1** 2-(4-fluorophenyl)-6-isocyano-1,3-bis(4-methoxyphenyl)azulene gold(I)chloride (**12b**) (50.0 mg, 72.3 µmol, 1.00 eq.) and *N*-methylaniline (**13h**) (8.13 mg, 75.9 µmol, 1.05 eq.) was stirred in dichloromethane at room temperature 12 hours. Purification by flash column chromatography (Silica, dichloromethane) yielded the title compound as a blue solid (51.0 mg, 63.8 µmol, 88%).

MP = 210 – 215 °C; **^{1}H{^{19}F} NMR** (300 MHz, CD_2Cl_2, 295 K) δ [ppm] = 8.04 (d, *J* = 10.8 Hz, 2H, 2x$C_{Ar}\underline{H}$), 7.54 – 7.46 (m, 3H, 3x$C_{Ar}\underline{H}$), 7.46 – 7.38 (m, 1H, $C_{Ar}\underline{H}$), 7.30 (s, 1H, N$\underline{H}$), 7.20 (dd, *J* = 8.3, 1.4 Hz, 2H, 2x$C_{Ar}\underline{H}$), 7.05 (d, *J* = 8.8 Hz, 4H, 4x$C_{Ar}\underline{H}$), 6.99 (s, 1H, $C_{Ar}\underline{H}$), 6.95 – 6.89 (m, 2H, 2x$C_{Ar}\underline{H}$), 6.80 (d, *J* = 8.9 Hz, 4H, 4x$C_{Ar}\underline{H}$), 6.78 – 6.71 (m, 2H, 2x$C_{Ar}\underline{H}$), 3.78 (s, 3H, -C$\underline{H}_3$), 3.72 (s, 6H, 2x-OC$\underline{H}_3$); **^{13}C{^{19}F, ^{1}H} NMR** (75 MHz, CD_2Cl_2, 295 K) δ [ppm] = 193.11 (s, 1C, $\underline{C}_{Carbene}$), 163.82 (s, 1C, $\underline{C}_{Ar}$), 160.56 (s, 1C, $\underline{C}_{Ar}$), 159.00 (s, 2C,2x $\underline{C}_{Ar}$), 148.91 (s, 1C, $\underline{C}_{Ar}$), 147.40 (s, 2C, 2x$\underline{C}_{Ar}$), 140.52 (s, 2C, 2x$\underline{C}_{Ar}$), 136.15 (s, 2C, 2x$\underline{C}_{Ar}$), 134.43 (d, 1C, $\underline{C}_{Ar}$H), 133.29 (d, 1C, $\underline{C}_{Ar}$H), 133.18 (d, 4C, 4x$\underline{C}_{Ar}$H), 132.80 (d, 1C, $\underline{C}_{Ar}$H), 131.79 (d, 2C, 2x$\underline{C}_{Ar}$H), 131.00 (s, 1C, $\underline{C}_{Ar}$), 130.43 (d, 1C, $\underline{C}_{Ar}$H), 129.94 (d, 1C, $\underline{C}_{Ar}$H), 128.13 (s, 1C, $\underline{C}_{Ar}$), 126.72 (d, 2C, 2x$\underline{C}_{Ar}$H), 120.14 (d, 2C, 2x$\underline{C}_{Ar}$H), 115.15 (d, 1C, $\underline{C}_{Ar}$H), 114.86 (d, 1C, $\underline{C}_{Ar}$H), 114.09 (d, 4C, 4x$\underline{C}_{Ar}$H), 55.60 (q, 2C, 2x-O$\underline{C}$H$_3$), 49.56 (q, 1C, -$\underline{C}$H$_3$); **^{19}F{^{1}H} NMR** (283 MHz, CD_2Cl_2, 298 K) δ [ppm] = -116.03 (s, 1F, -$C_{Ar}\underline{F}$); **IR** (ATR) $\tilde{\nu}$ [cm^{-1}] = 3325 (w), 2834 (w), 1732 (w), 1605 (m), 1515 (s), 1492 (m), 1441 (m), 1387 (m), 1342 (m), 1286 (m), 1242

(s), 1174 (s), 1157 (m), 1106 (w), 1030 (s), 831 (s), 793 (m), 768 (m), 698 (m); **UV/VIS** (CH_2Cl_2) λ_{max} (ε) [nm (l*mol^{-1}*cm^{-1})] = 335 (109661), 388 (10282), 423 (20243), 621 (314), 667 (192), 765 (21); **HR-MS** (MALDI): $C_{38}H_{31}Au^{35}ClFN_2O_2$ $[M]^+$ calcd.: 798.1724 found.: 798.1712, Δm/z = 1.503 ppm, $C_{38}H_{31}Au^{37}ClFN_2O_2$ $[M]^+$ calcd.: 800.1694, found.: 800.1700, Δm/z = 0.750 ppm; **Elemental Analysis** calcd. for $C_{38}H_{31}AuClFN_2O_2$: C: 57.12, H: 3.91, N : 3.51, found C: 56.77, H: 4.17, N: 3.47.

Synthesis of (((2-(4-fluorophenyl)-1,3-bis(4-methoxyphenyl)azulen-6-yl)amino)(isopropyl(phenyl)amino)methylene)gold(I) chloride (14bi)

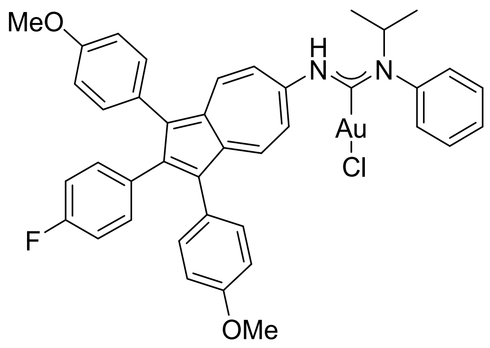

According to **GP1** 2-(4-fluorophenyl)-6-isocyano-1,3-bis(4-methoxyphenyl)azulene gold(I)chloride (**12b**) (50.0 mg, 72.3 µmol, 1.00 eq.) and *N*-isopropylaniline (**13i**) (10.3 mg, 75.9 µmol, 1.05 eq.) was stirred in dichloromethane at room temperature 12 hours. Purification by flash column chromatography (Silica, dichloromethane) yielded the title compound as a blue solid (48.0 mg, 58.0 µmol, 80%).

MP = 196 – 201 °C;**^{1}H{^{19}F} NMR** (500 MHz, CD_2Cl_2, 295 K) δ [ppm] = 8.04 (d, *J* = 11.0 Hz, 2H, 2x$C_{Ar}\underline{H}$), 7.56 – 7.49 (m, 2H, 2x$C_{Ar}\underline{H}$), 7.48 – 7.43 (m, 1H, $C_{Ar}\underline{H}$), 7.32 (s, 1H, N$\underline{H}$), 7.13 (dd, *J* = 8.4, 1.3 Hz, 2H, 2x$C_{Ar}\underline{H}$), 7.05 (d, *J* = 8.7 Hz, 4H, 4x$C_{Ar}\underline{H}$), 7.00 (d, *J* = 11.1 Hz, 2H, 2x$C_{Ar}\underline{H}$), 6.91 (dd, *J* = 8.9, 5.6 Hz, 2H, 2x$C_{Ar}\underline{H}$), 6.80 (d, *J* = 8.8 Hz, 4H, 4x$C_{Ar}\underline{H}$), 6.74 (t, *J* = 8.9 Hz, 2H, 2x$C_{Ar}\underline{H}$), 5.44 (p, *J* = 6.8 Hz, 1H, -C$\underline{H}$(CH_3)$_2$), 3.72 (s, 6H, -OC$\underline{H}_3$), 1.13 (d, *J* = 6.8 Hz, 6H, -CH(C$\underline{H}_3$)$_2$); **^{13}C{^{19}F, ^{1}H} NMR** (126 MHz, CD_2Cl_2, 295 K) δ [ppm] = 192.70 (s, 1C, $\underline{C}_{Carbene}$), 163.11 (s, 1C, $\underline{C}_{Ar}$), 161.16 (s, 1C, $\underline{C}_{Ar}$), 158.93 (s, 2C, 2x$\underline{C}_{Ar}$), 147.85 (s, 2C, 2x$\underline{C}_{Ar}$), 147.24 (s, 2C, 2x$\underline{C}_{Ar}$), 136.11 (s, 2C, 2x$\underline{C}_{Ar}$), 135.33 (s, 1C, $\underline{C}_{Ar}$), 134.39 (d, 2C, 2x$\underline{C}_{Ar}$H), 133.24 (d, 4C, 4x$\underline{C}_{Ar}$H), 133.18 (d, 1C, $\underline{C}_{Ar}$H), 132.78 (d, 1C, $\underline{C}_{Ar}$H), 131.18 (d, 2C, 2x$\underline{C}_{Ar}$H), 130.85 (s, 1C, $\underline{C}_{Ar}$), 130.62 (d, 1C, $\underline{C}_{Ar}$H), 129.60 (d, 2C,2x $\underline{C}_{Ar}$H), 128.12 (s, 1C, $\underline{C}_{Ar}$), 120.40 (d, 2C, 2x$\underline{C}_{Ar}$H), 115.06 (d, 1C, $\underline{C}_{Ar}$H), 114.89 (d, 1C, $\underline{C}_{Ar}$H), 114.05 (d, 4C, 2x$\underline{C}_{Ar}$H), 62.31 (d, 1C, -$\underline{C}$H(CH_3)$_2$), 55.57 (q, 2C, 2x-O$\underline{C}$H$_3$), 21.90 (q, 2C, 2x-CH($\underline{C}$H$_3$)$_2$); **^{19}F{^{1}H} NMR** (283 MHz, CD_2Cl_2, 298 K) δ [ppm] = -116.08 (s, 1F, -$C_{Ar}\underline{F}$); **IR** (ATR) $\tilde{\nu}$ [cm-

^{1}] = 3333 (w), 2970 (w), 2834 (w), 1736 (w), 1604 (w), 1573 (w), 1530 (s), 1513 (s), 1489 (m), 1425 (w), 1357 (m), 1327 (m), 1285 (w), 1243 (s), 1174 (m), 1113 (m), 1028 (m), 851 (m), 792 (s), 702 (m), 662 (w); **UV/VIS** (CH_2Cl_2) λ_{max} (ε) [nm (l*mol^{-1}*cm^{-1})] = 282 (26919), 336 (64306), 385 (10468), 415 (5982), 622 (307), 663 (278), 756 (82); **HR-MS** (MALDI): $C_{40}H_{35}Au^{35}ClFN_2O_2$ $[M]^+$ calcd.: 826.2037, found.: 826.2023, Δm/z = 1.694 ppm, $C_{40}H_{35}Au^{37}ClFN_2O_2$ $[M]^+$ calcd.: 828.2007, found.: 828.2012, Δm/z = 0.604 ppm; **Elemental Analysis** calcd. for $C_{40}H_{35}AuClFN_2O_2$: C: 58.08, H: 4.92, N : 3.39, found C: 58.11, H: 4.66, N: 3.29.

Synthesis of (1-(2-(2-fluorophenyl)-1,3-bis(4-methoxyphenyl)azulen-4-yl)-3-isopropylimidazolidin-2-ylidene)gold(I) chloride (16a)

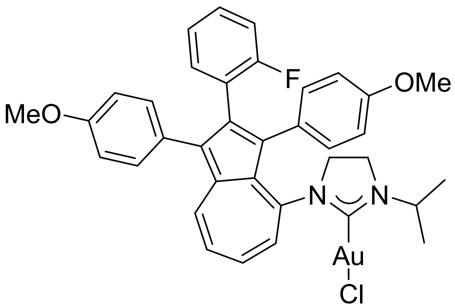

According to **GP1** 2-(2-fluorophenyl)-4-isocyano-1,3-bis(4-methoxyphenyl)azulene gold(I)chloride (**12a**) (50.0 mg, 72.3 μmol, 1.00 eq.) N-(2-chloroethyl)propan-2-amine hydrochloride (**15**) (11.99 mg, 75.9 μmol, 1.05 eq.) and triethylamine (**9**) (73.1 mg, 0.1 mL, 722 μmol, 10.0 eq.)was stirred in dichloromethane at room temperature 12 hours. Purification by flash column chromatography (Silica, dichloromethane) yielded the title compound as a blue solid (35.0 mg, 45.04 μmol, 60%).

MP = 225 – 230 °C;**^{1}H{^{19}F} NMR** (301 MHz, C_6D_6, 295 K) δ [ppm] = 8.21 – 7.98 (m, 1H, $C_{Ar}\underline{H}$), 7.19 – 7.02 (m, 4H, $C_{Ar}\underline{H}$), 6.90 (d, *J* = 10.0 Hz, 1H, $C_{Ar}\underline{H}$), 6.77 – 6.62 (m, 2H, 2x$C_{Ar}\underline{H}$), 6.58 (s, 1H, $C_{Ar}\underline{H}$), 6.57 – 6.51 (m, 3H, 3x$C_{Ar}\underline{H}$), 6.46 (dd, *J* = 11.1, 4.3 Hz, 1H, $C_{Ar}\underline{H}$), 6.40 (dd, *J* = 8.5, 4.3 Hz, 1H, $C_{Ar}\underline{H}$), 6.29 (dd, *J* = 8.7, 2.9 Hz, 1H, $C_{Ar}\underline{H}$), 6.23 (dd, *J* = 8.3, 2.8 Hz, 1H, $C_{Ar}\underline{H}$), 4.40 (p, *J* = 6.7 Hz, 1H, -C$\underline{H}$(CH_3)$_2$), 3.07 (s, 3H, -OC$\underline{H_3}$), 2.98 (s, 3H, -OC$\underline{H_3}$), 2.89 – 2.71 (m, 1H, -C$\underline{H_2}$-), 2.29 (p, *J* = 11.1 Hz, 1H, -C$\underline{H_2}$-), 2.00 – 1.87 (m, 1H, -C$\underline{H_2}$-), 1.41 (q, *J* = 11.0 Hz, 1H, -C$\underline{H_2}$-), 0.50 (d, *J* = 6.9 Hz, 3H, -CH(C$\underline{H_3}$)$_2$), 0.45 (d, *J* = 6.7 Hz, 3H, -CH(C$\underline{H_3}$)$_2$);**^{13}C{^{19}F, ^{1}H} NMR** (101 MHz, C_6D_6, 295 K) δ [ppm] = 194.14 (s, 1C, $\underline{C_{Carbene}}$), 161.94 (s, 1C, $\underline{C_{Ar}}$), 159.50 (s, 1C, $\underline{C_{Ar}}$), 159.33 (s, 1C, $\underline{C_{Ar}}$), 158.31 (s, 1C, $\underline{C_{Ar}}$), 146.42 (s, 1C, $\underline{C_{Ar}}$), 145.26 (s, 1C, $\underline{C_{Ar}}$), 139.66 (s, 1C, $\underline{C_{Ar}}$), 137.20 (d, 1C, $\underline{C_{Ar}}$H), 136.81 (d, 1C, $\underline{C_{Ar}}$H), 136.42 (d, 1C, $\underline{C_{Ar}}$H), 133.30 (d, 1C, $\underline{C_{Ar}}$H), 132.71 (s, 1C, $\underline{C_{Ar}}$), 132.43 (d, 1C, $\underline{C_{Ar}}$H), 130.55

(d, 1C, $\underline{C}_{Ar}$H), 129.41 (s, 1C, $\underline{C}_{Ar}$), 129.28 (d, 1C, $\underline{C}_{Ar}$H), 129.09 (s, 1C, $\underline{C}_{Ar}$), 128.92 (s, 1C, $\underline{C}_{Ar}$), 125.83 (d, 1C, $\underline{C}_{Ar}$H), 125.71 (s, 1C, $\underline{C}_{Ar}$), 125.39 (d, 1C, $\underline{C}_{Ar}$H), 124.45 (d, 1C, $\underline{C}_{Ar}$H), 123.94 (d, 1C, $\underline{C}_{Ar}$H), 115.56 (d, 1C, $\underline{C}_{Ar}$H), 115.34 (d, 1C, $\underline{C}_{Ar}$H), 114.23 (d, 1C, $\underline{C}_{Ar}$H), 113.35 (d, 1C, $\underline{C}_{Ar}$H), 112.32 (d, 1C, $\underline{C}_{Ar}$H), 54.71 (q, 2C, 2x-O$\underline{C}H_3$), 52.92 (t, 1C, -$\underline{C}H_2$-), 52.00 (d, 1C, -CH($CH_3)_2$), 42.15 (t, 1C, -$\underline{C}H_2$-), 20.59 (q, 1C, -CH($\underline{C}H_3)_2$), 19.82 (q, 1C, -CH($\underline{C}H_3)_2$); **^{19}F{^{1}H} NMR** (283 MHz, C_6D_6, 295 K) δ [ppm] = -112.99 (s, 1F, $C_{Ar}\underline{F}$); **IR** (ATR) $\tilde{\nu}$ [cm^{-1}] = 2969 (w), 2931 (w), 2834 (w), 1738 (w), 1712 (w), 1608 (m), 1559 (m), 1504 (s), 1451 (s), 1356 (m), 1323 (m), 1269 (s), 1244 (s), 1204 (m), 1175 (m), 1107 (w), 1031 (m), 945 (w), 828 (m), 760 (m), 732 (w); **HR-MS** (MALDI): $C_{36}H_{33}AuClFN_2O_2$ $[M]^+$ calcd.: 776.1880, found.: 776.1884, Δm/z = 0.515 ppm; **UV/VIS** (CH_2Cl_2) λ_{max} (ε) [nm ($l*mol^{-1}*cm^{-1}$)] = 258 (29555), 293 (37450), 314 (35392), 619 (484), 665 (439), 733 (182); **Elemental Analysis** calcd. for $C_{36}H_{33}AuClFN_2O$: C: 55.64, H: 4.28, N : 3.61, found C: 55.78, H: 4.45, N: 3.70.

Synthesis of (1-(2-(4-fluorophenyl)-1,3-bis(4-methoxyphenyl)azulen-6-yl)-3-isopropylimidazolidin-2-ylidene)gold(I) chloride (16b)

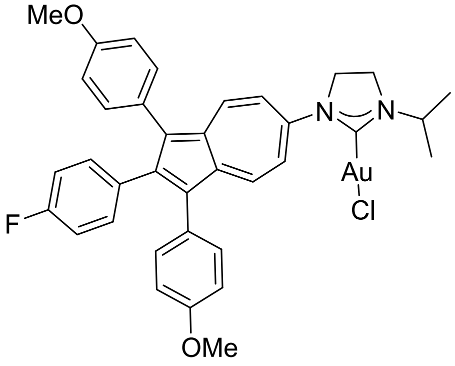

According to **GP1** 2-(4-fluorophenyl)-6-isocyano-1,3-bis(4-methoxyphenyl)azulene gold(I)chloride (**12b**) (50.0 mg, 72.3 µmol, 1.00 eq.) N-(2-chloroethyl)propan-2-amine hydrochloride (**15**) (12.0 mg, 75.9 µmol, 1.05 eq.) and triethylamine (**9**) (73.1 mg, 0.1 mL, 722 µmol, 10.0 eq.)was stirred in dichloromethane at room temperature 12 hours. Purification by flash column chromatography (Silica, dichloromethane) yielded the title compound as a blue solid (48.0 mg, 61.8 µmol, 85%).

MP = 236 – 241 °C;**^{1}H{^{19}F} NMR** (300 MHz, CD_2Cl_2, 295 K) δ [ppm] = 8.19 (d, J = 10.8 Hz, 2H, 2x$C_{Ar}\underline{H}$), 7.26 (d, J = 10.8 Hz, 2H, 2x$C_{Ar}\underline{H}$), 7.17 (d, J = 8.9 Hz, 4H, 4x$C_{Ar}\underline{H}$), 7.03 (dd, J = 8.9, 5.6 Hz, 2H, 2x$C_{Ar}\underline{H}$), 6.92 (d, J = 8.9 Hz, 4H, 4x$C_{Ar}\underline{H}$), 6.85 (t, J = 8.9 Hz, 2H, 2x$C_{Ar}\underline{H}$), 4.95 (hept, J = 6.6 Hz, 1H, -C$\underline{H}$($CH_3)_2$), 4.22 – 4.11 (m, 2H, -C$\underline{H_2}$-), 3.83 (s, 6H, 2x-OC$\underline{H_3}$), 3.80 – 3.70 (m, 2H, -C$\underline{H_2}$-), 1.32 (d, J = 6.7 Hz, 6H, -CH(C$\underline{H_3})_2$); **^{13}C{^{19}F,^{1}H} NMR** (75 MHz, CD_2Cl_2, 295 K) δ [ppm] = 191.19 (s, 1C, $\underline{C}_{Carbene}$), 163.82 (s, 1C, $\underline{C}_{Ar}$), 160.57 (s, 2C, 2x$\underline{C}_{Ar}$),

159.01 (s, 1C, $\underline{C}_{Ar}$), 149.26 (s, 2C, 2x$\underline{C}_{Ar}$), 147.26 (s, 2C, 2x$\underline{C}_{Ar}$), 136.19 (s, 2C, 2x$\underline{C}_{Ar}$), 134.61 (d, 2C, 2x$\underline{C}_{Ar}$H), 133.32 (d, 4C, 4x$\underline{C}_{Ar}$H), 133.21 (d, 1C, $\underline{C}_{Ar}$H), 132.82 (d, 4C, 4x$\underline{C}_{Ar}$H), 130.99 (s, 1C, $\underline{C}_{Ar}$), 128.24 (s, 1C, $\underline{C}_{Ar}$), 119.46 (d, 2C, 2x$\underline{C}_{Ar}$H), 115.14 (d, 2C, 2x$\underline{C}_{Ar}$H), 114.86 (d, 1C, $\underline{C}_{Ar}$H), 114.10 (d, 4C, 2x$\underline{C}_{Ar}$H), 55.62(q, 2C, 2x-O$\underline{C}H_3$); 51.79 (t, 1C, -$\underline{C}H_2$-), 43.72 (t, 1C, -$\underline{C}H_2$-), 20.69 (q, 2C, -CH($\underline{CH_3}$)$_2$) [signal for -$\underline{C}H(CH_3)_2$ was not observed]; **^{19}F{^{1}H} NMR** (283 MHz, CD_2Cl_2, 298 K) δ [ppm] = -116.13 (s, 1F, -$C_{Ar}\underline{F}$); **IR** (ATR) $\tilde{\nu}$ [cm^{-1}] = 2966 (w), 2833 (w), 1605 (m), 1573 (m), 1545 (w), 1510 (s), 1470 (m), 1407 (s), 1349 (w), 1274 (s), 1242 (s), 1173 (s), 1156 (m), 1108 (w), 1031 (m), 949 (w), 828 (s), 792 (s), 662 (w); **UV/VIS** (CH_2Cl_2) λ_{max} (ε) [nm (l*mol^{-1}*cm^{-1})] = 342 (66881), 418 (7236), 623 (295), 665 (277), 757 (80); **HR-MS** (MALDI): $C_{36}H_{33}Au^{35}ClFN_2O_2$ $[M]^+$ calcd.: 776.1880, found.: 776.1870, Δm/z = 1.288 ppm, $C_{36}H_{33}Au^{37}ClFN_2O_2$ $[M]^+$ calcd.: 778.1859, found.: 778.1856, Δm/z = 0.386 ppm; **Elemental Analysis** calcd. for $C_{36}H_{33}AuClFN_2O$: C: 55.64, H: 4.28, N : 3.61, found C: 55.86, H: 4.51, N: 3.70.

3 Cell viability assay

Cancerous (A2780, A2780*cis*, MDA-MB-231 and U87) cell lines were obtained from ATCC and cultured according to the supplier's recommendations. Cells were maintained in specify medium, supplemented with 10% fetal bovine serum (FBS, [supplier]), 1% penicillin-streptomycin , and incubated at 37 °C in a humidified atmosphere containing 5% CO_2.

A total of 1.5 × 10^3 cells per well were seeded in 96-well white opaque plates (Corning, Costar®) and allowed to adhere overnight. Cells were treated with six concentrations (0.001, 0.01, 0.1, 1, 10, and 100 μM) of the gold carbene compounds. These concentrations were obtained by serial dilution in the respective culture medium from 10 mM stock solutions prepared in DMSO. The final DMSO concentration did not exceed 0.1% (v/v) in any well.

Cisplatin was used as a reference compound under identical experimental conditions. A 10 mM cisplatin stock solution was freshly prepared in sterile water before each experiment.

After 96 hours of treatment, cell viability was assessed using the CellTiter-Glo® Luminescent Cell Viability Assay (Promega, Madison, WI, USA), strictly following the manufacturer's technical bulletin. Briefly, an equal volume of CellTiter-Glo reagent was added to each well, followed by 2 minutes of orbital shaking to induce cell lysis, and 10 minutes of incubation at room temperature to stabilize the luminescent signal. Luminescence, which correlates with intracellular ATP levels, was recorded using a Tecan Infinite® M1000 plate reader.

All experiments were performed in triplicate and error bars are standard deviations.

Dose–response curves and IC_{50} values were calculated using nonlinear regression in GraphPad Prism 8 software. An ATP standard curve was included to confirm assay linearity and performance under our experimental conditions.

4 NMR Spectra

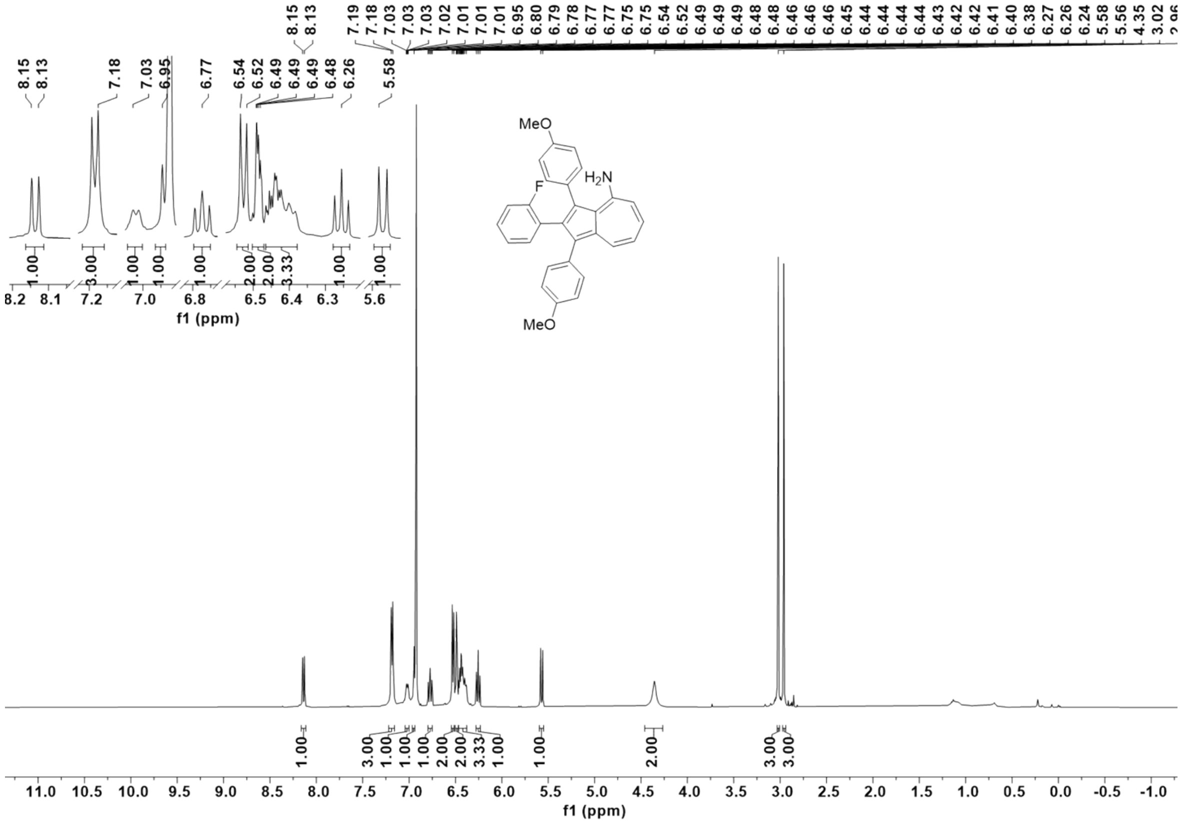

Figure S1. **^{1}H{^{19}F} NMR** Spectrum (500 MHz, C_6D_6, 295 K) of **4a.**

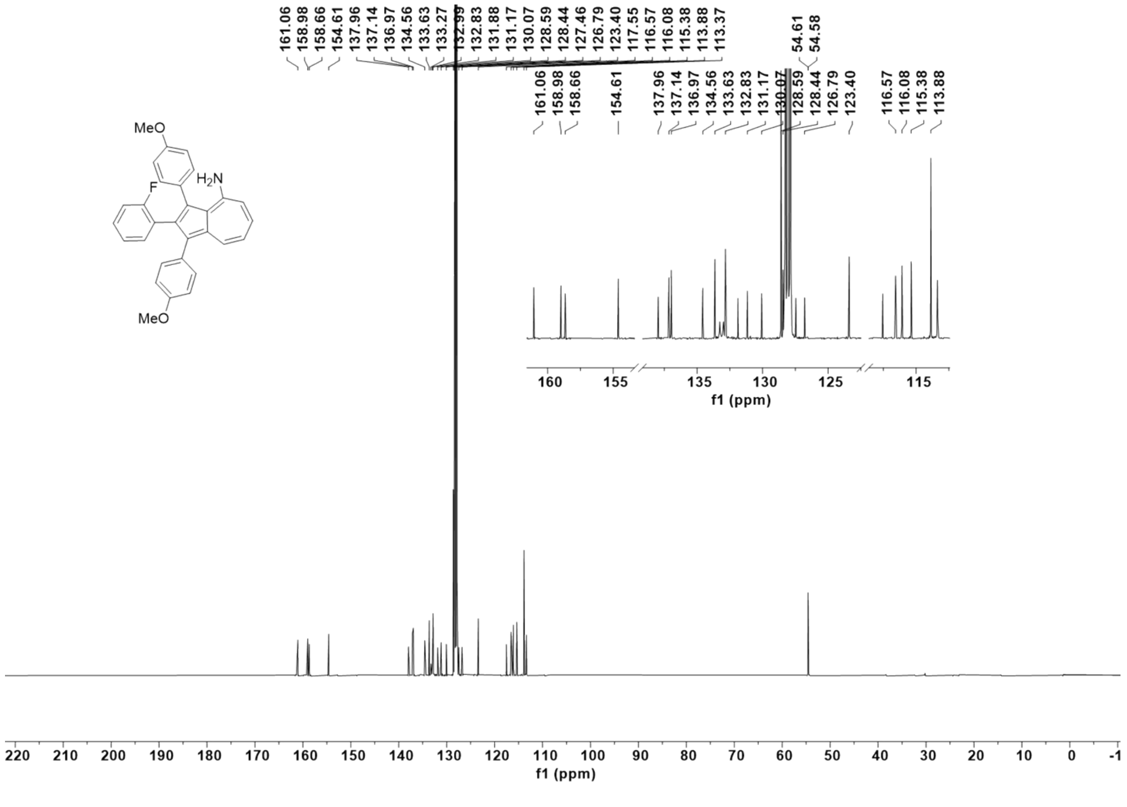

Figure S2. **^{13}C{^{19}F,^{1}H} NMR** Spectrum (126 MHz, C_6D_6, 295 K) of **4a.**

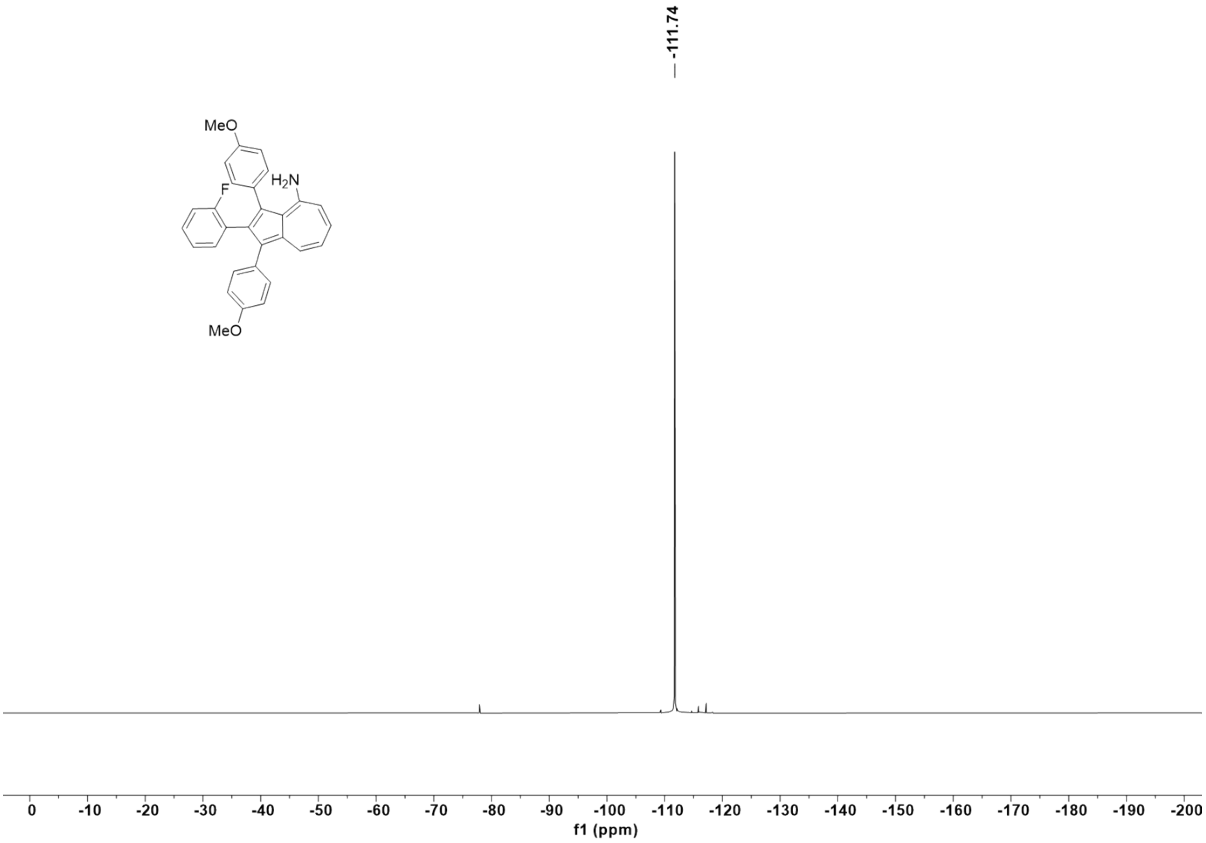

Figure S3. **^{19}F{^{1}H} NMR** Spectrum (471 MHz, C_6D_6, 295 K) of **4a.**

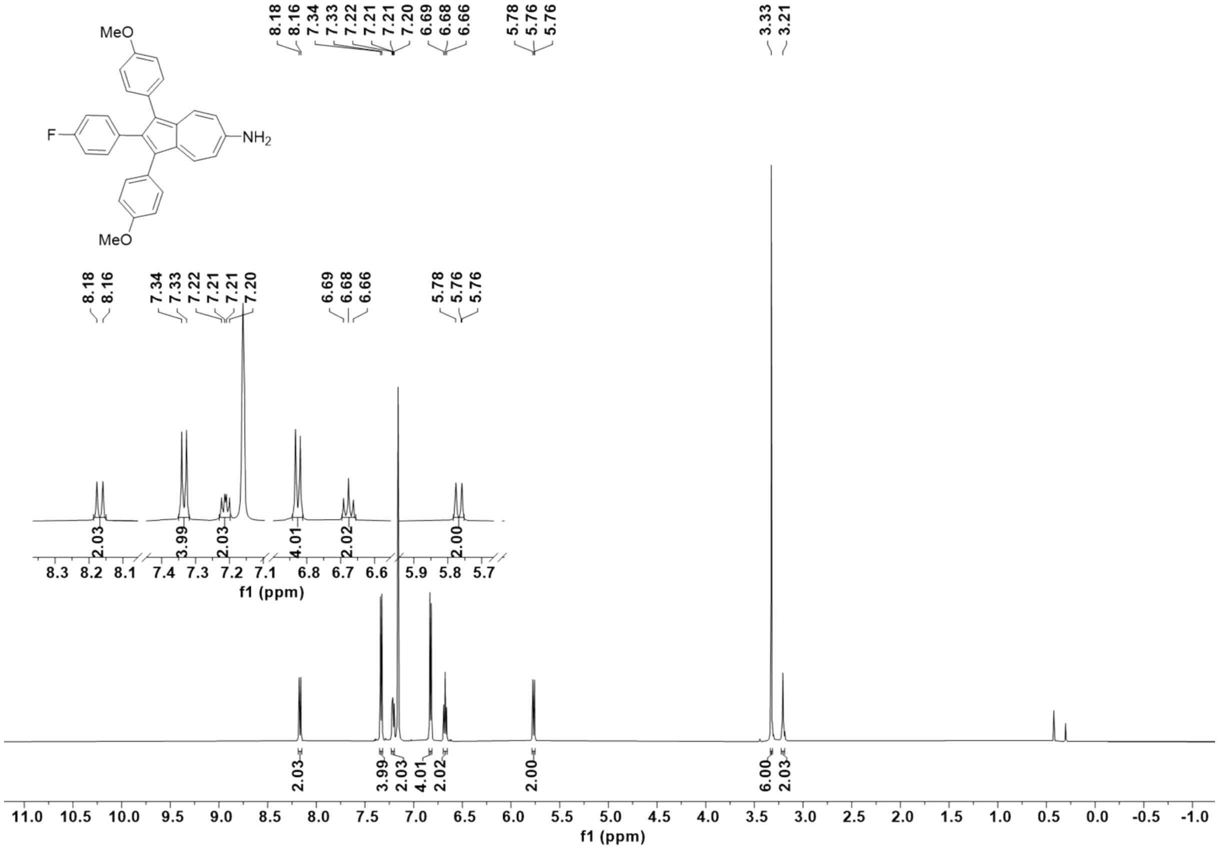

Figure S4. **^{1}H{^{19}F} NMR** Spectrum (600 MHz, C_6D_6, 295 K) of **4b**.

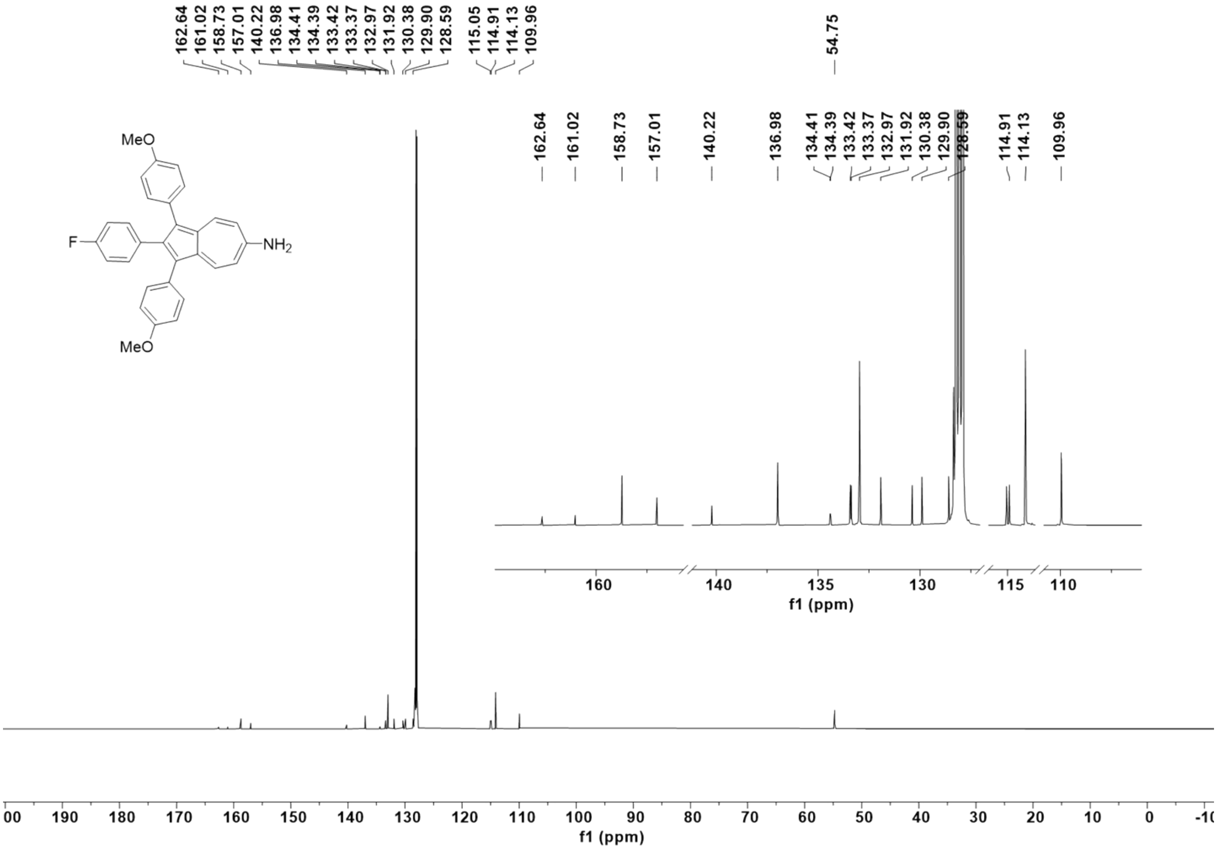

Figure S5. $^{13}C\{^{19}F,^{1}H\}$ **NMR** Spectrum (151 MHz, C_6D_6, 295 K) of **4b**.

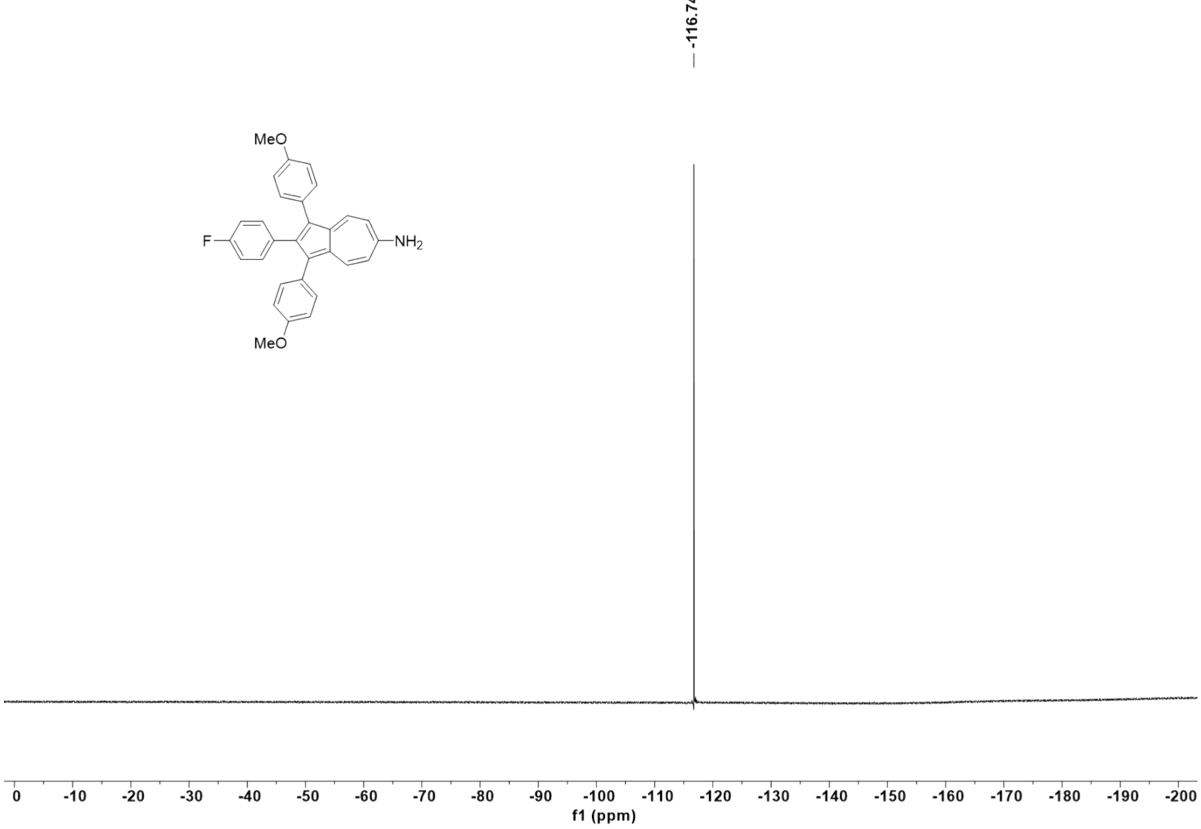

Figure S6. $^{19}F\{^{1}H\}$ **NMR**Spectrum (283 MHz, C_6D_6, 295 K) of **4b**.

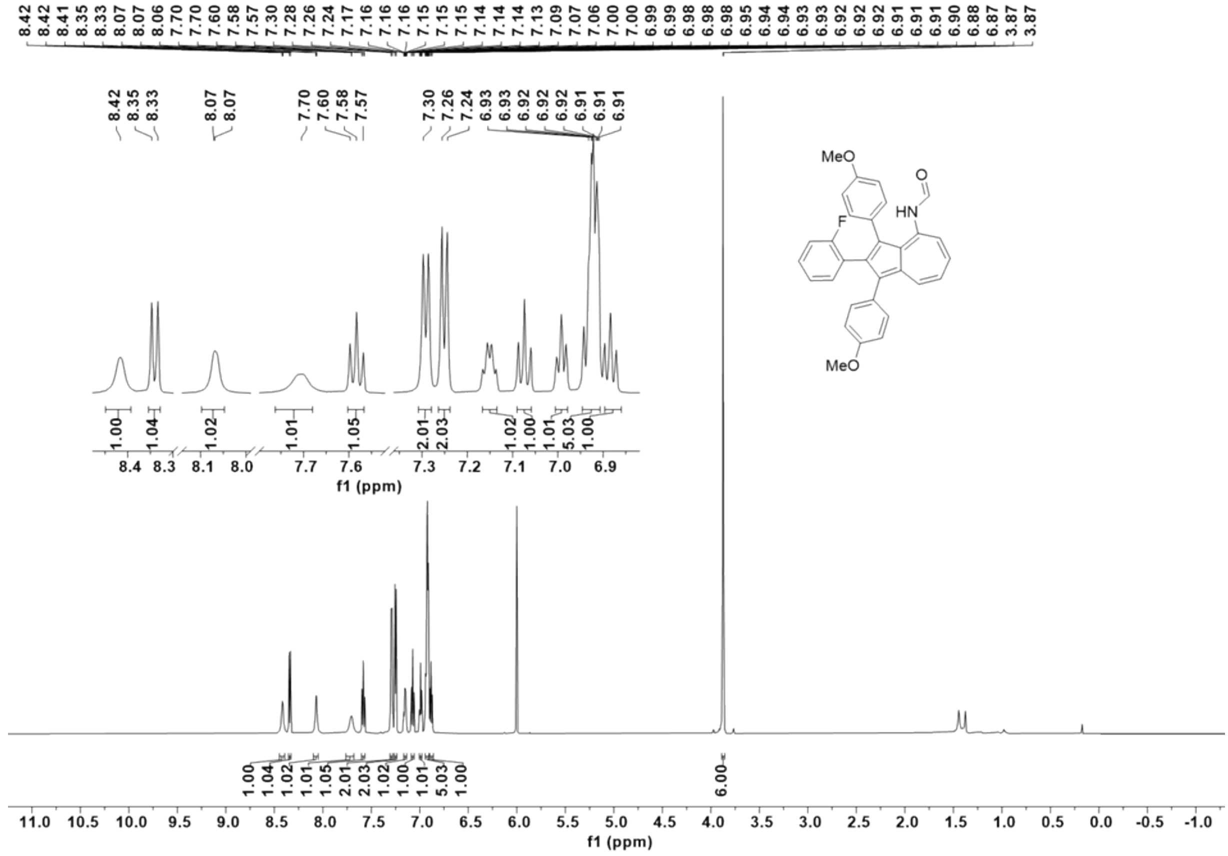

Figure S7. ^{1}**H{**^{19}**F} NMR** Spectrum (700 MHz, $C_2D_2Cl_4$, 400 K) of **6a**.

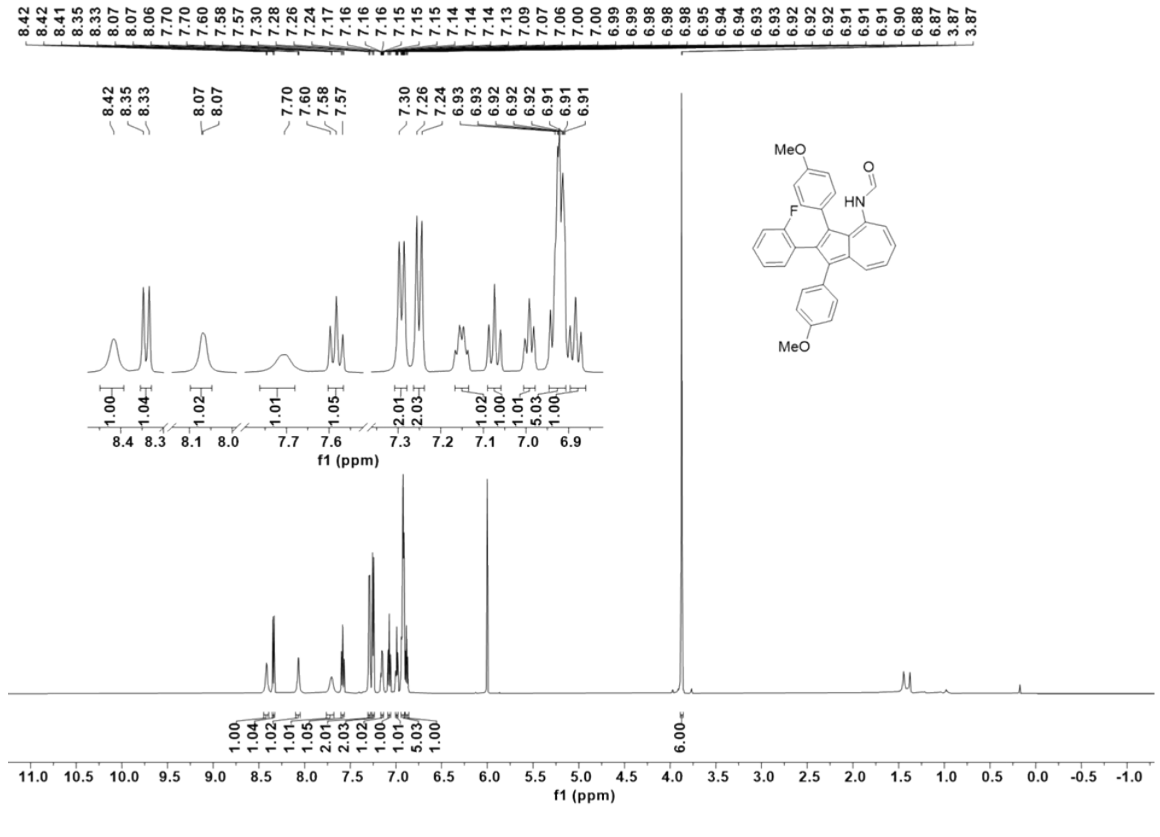

Figure S8. ^{13}**C{**^{1}**H} NMR** Spectrum (176 MHz, $C_2D_2Cl_4$, 400 K) of **6a**.

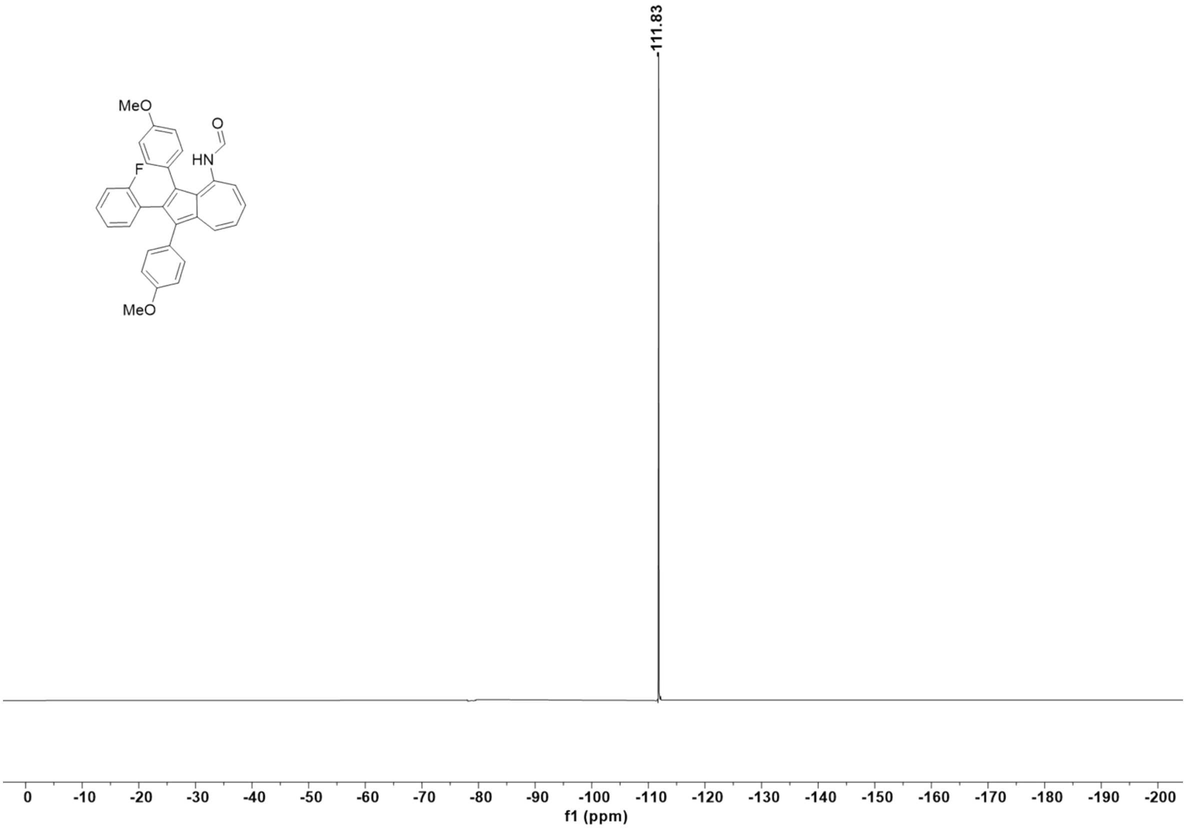

Figure S9. ^{19}**F{**^{1}**H} NMR** Spectrum (471 MHz, C_6D_6, 295 K) of **6a**.

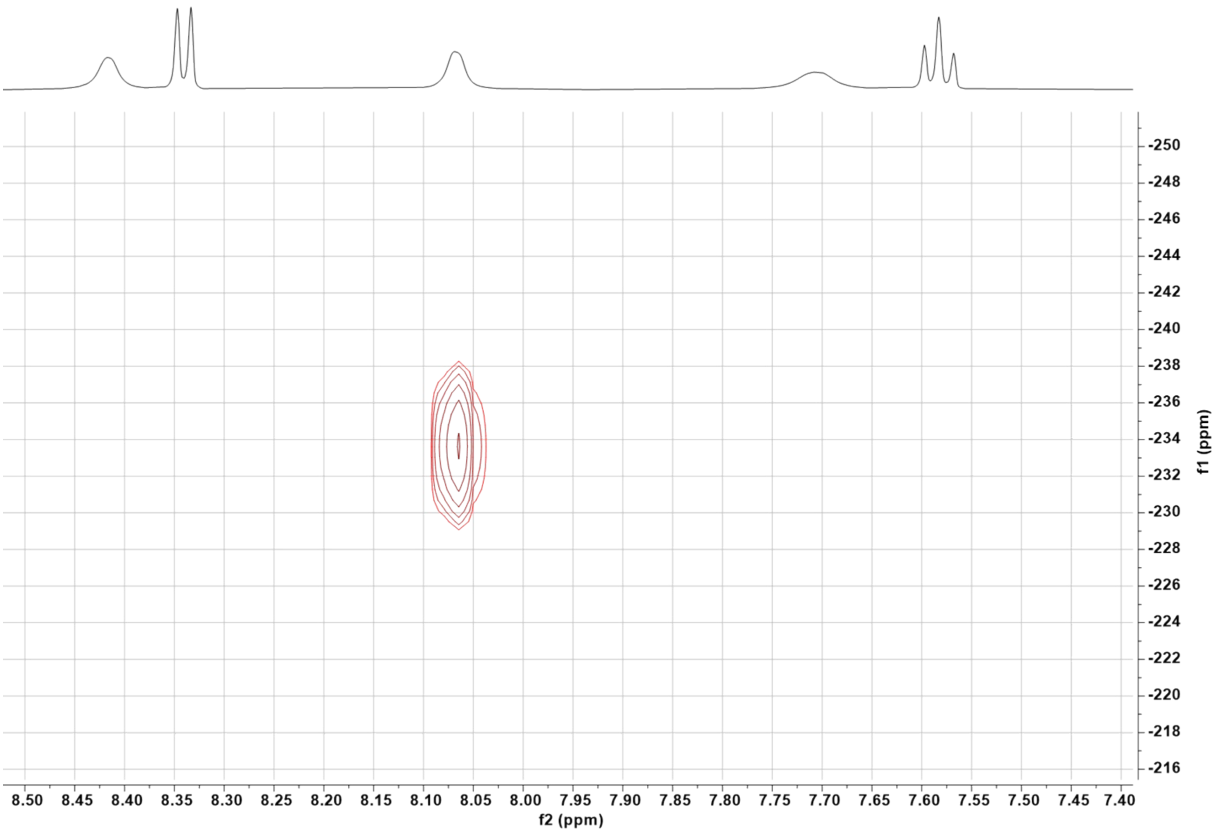

Figure S10. ^{1}H, ^{15}N-HSQC Spectrum of **6a**.

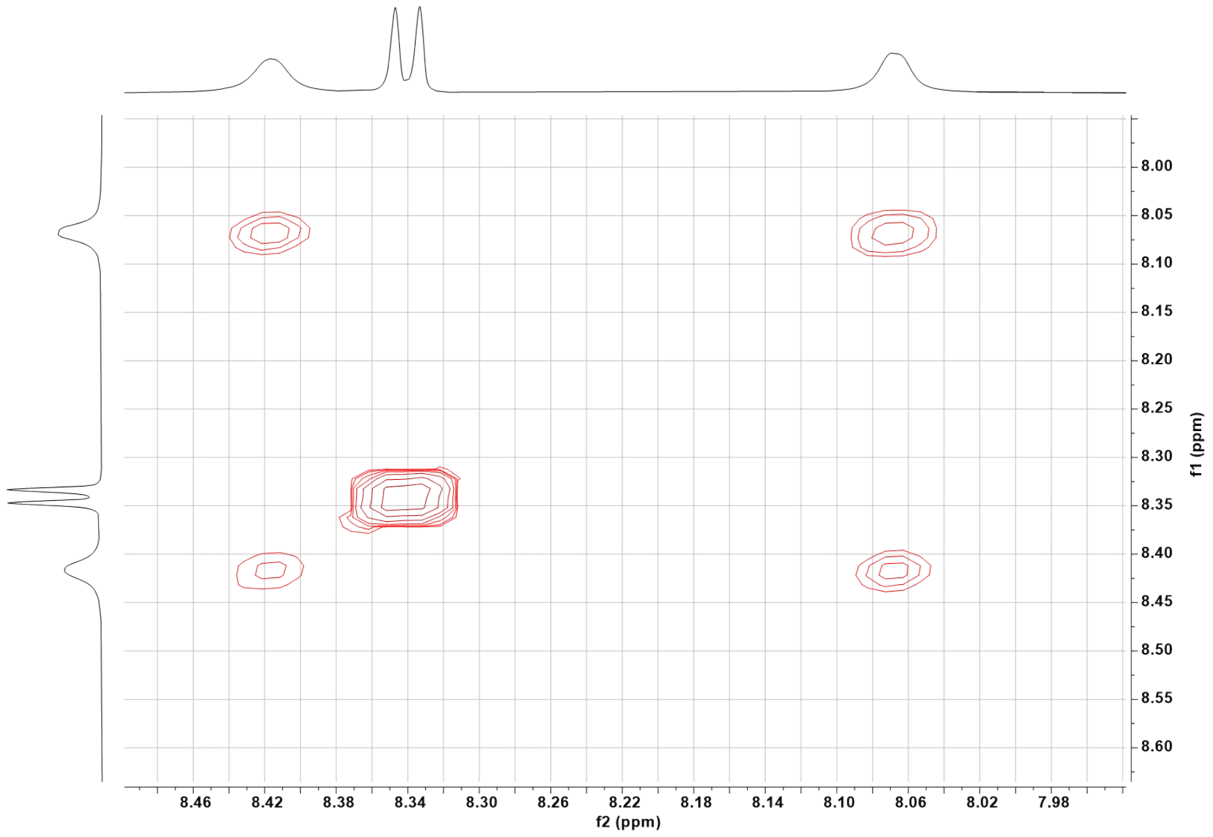

Figure S11. ^{1}H, ^{1}H COSY Spectrum of **6a**.

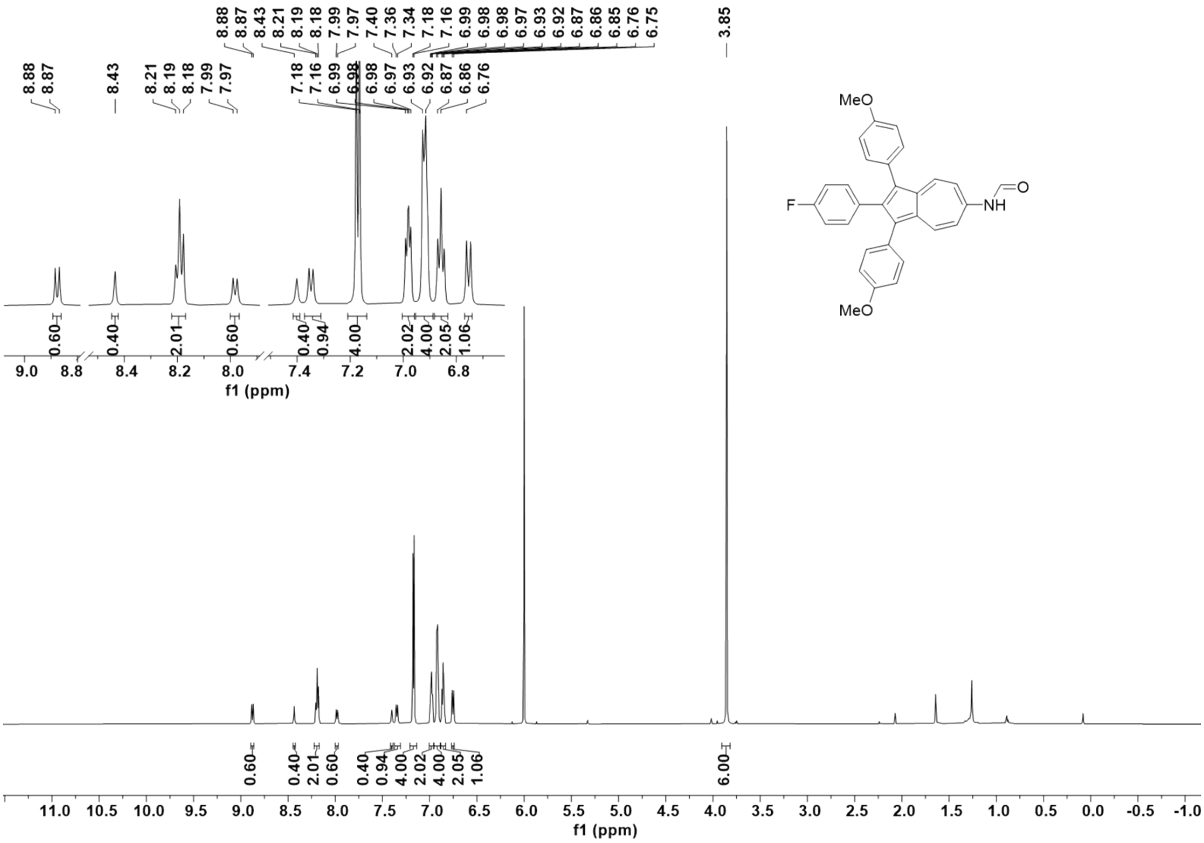

Figure S12. **^{1}H{^{19}F} NMR** Spectrum (700 MHz, $C_2D_2Cl_4$, 295 K of **6b**.

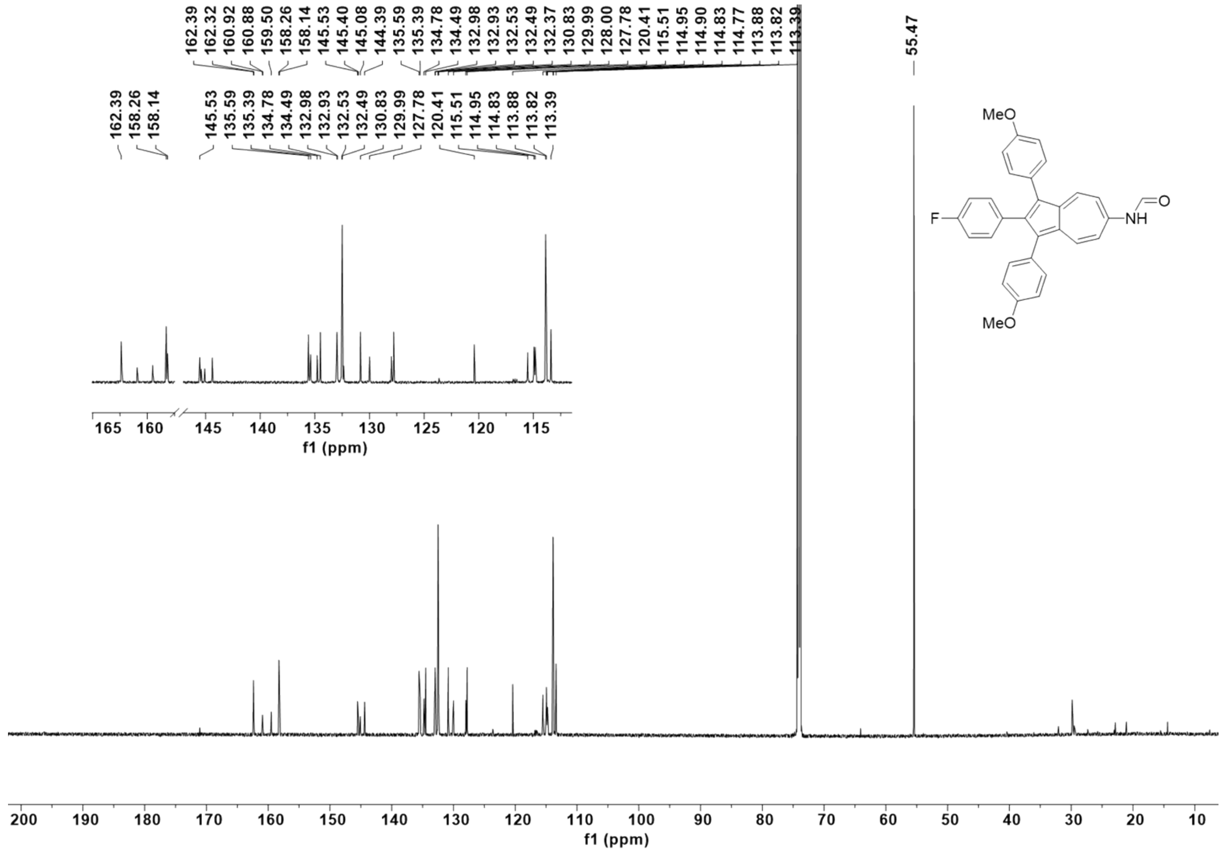

Figure S13. $^{13}C\{^{19}F,^{1}H\}$ **NMR** Spectrum (151 MHz, CD_2Cl_2, 295 K) of **6b**.

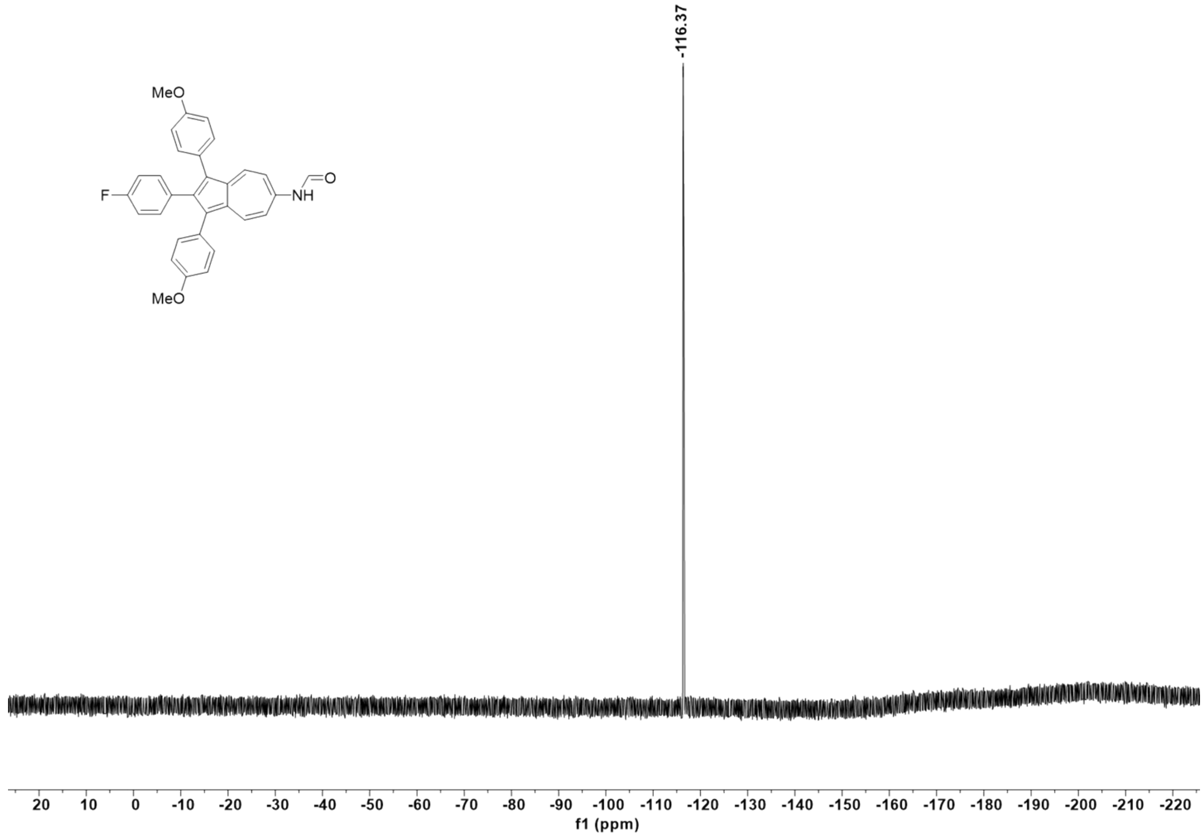

Figure S14. $^{19}F\{^{1}H\}$ **NMR** Spectrum (283 MHz, CD_2Cl_2, 295 K) of **6b**.

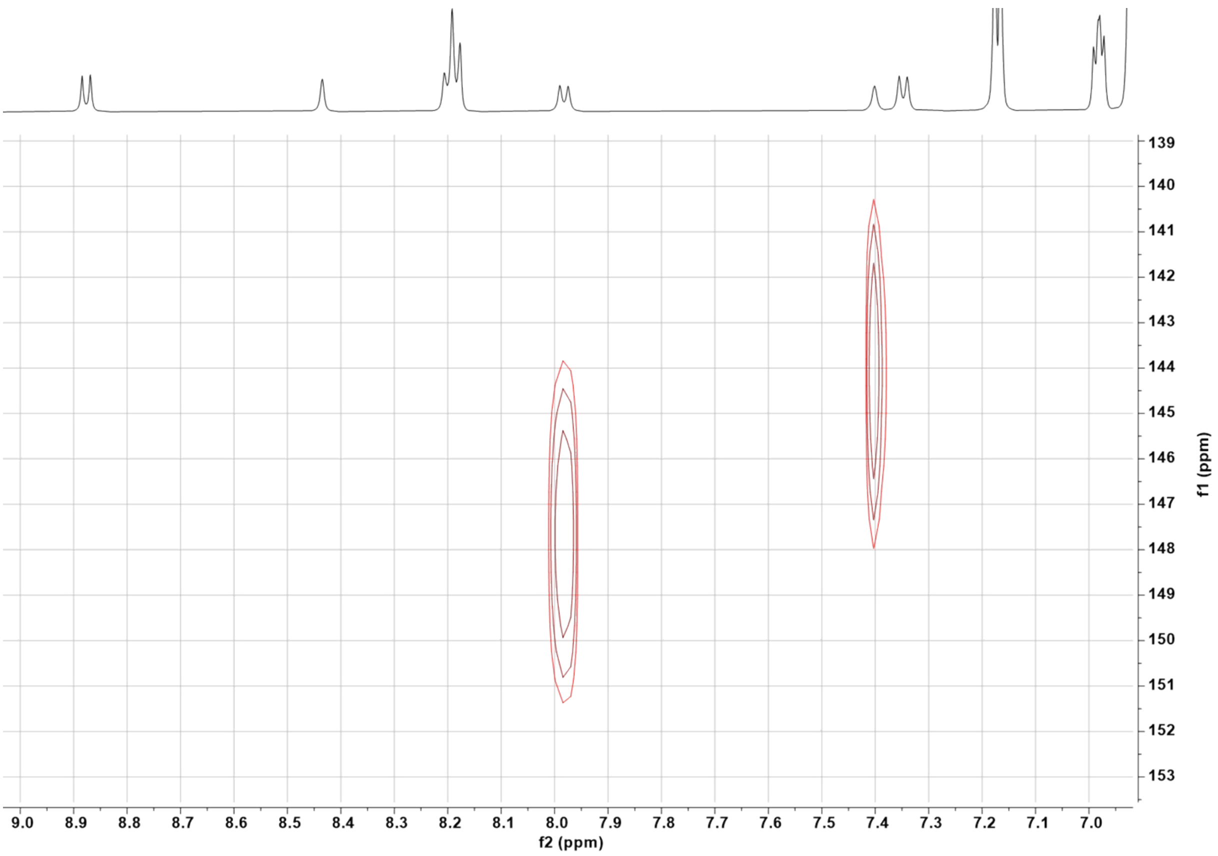

Figure S15. ^{1}H, ^{15}N-HSQC Spectrum of **6b**.

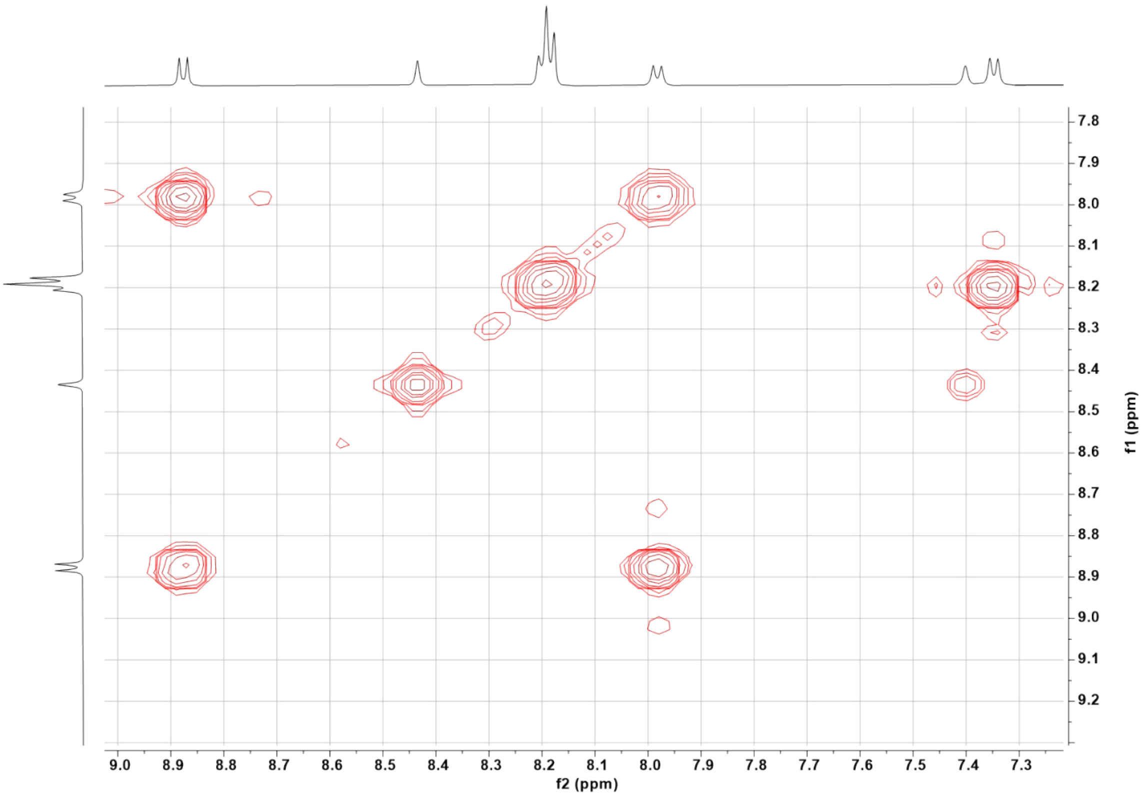

Figure S16. ^{1}H, ^{1}H-COSY Spectrum of **6b**.

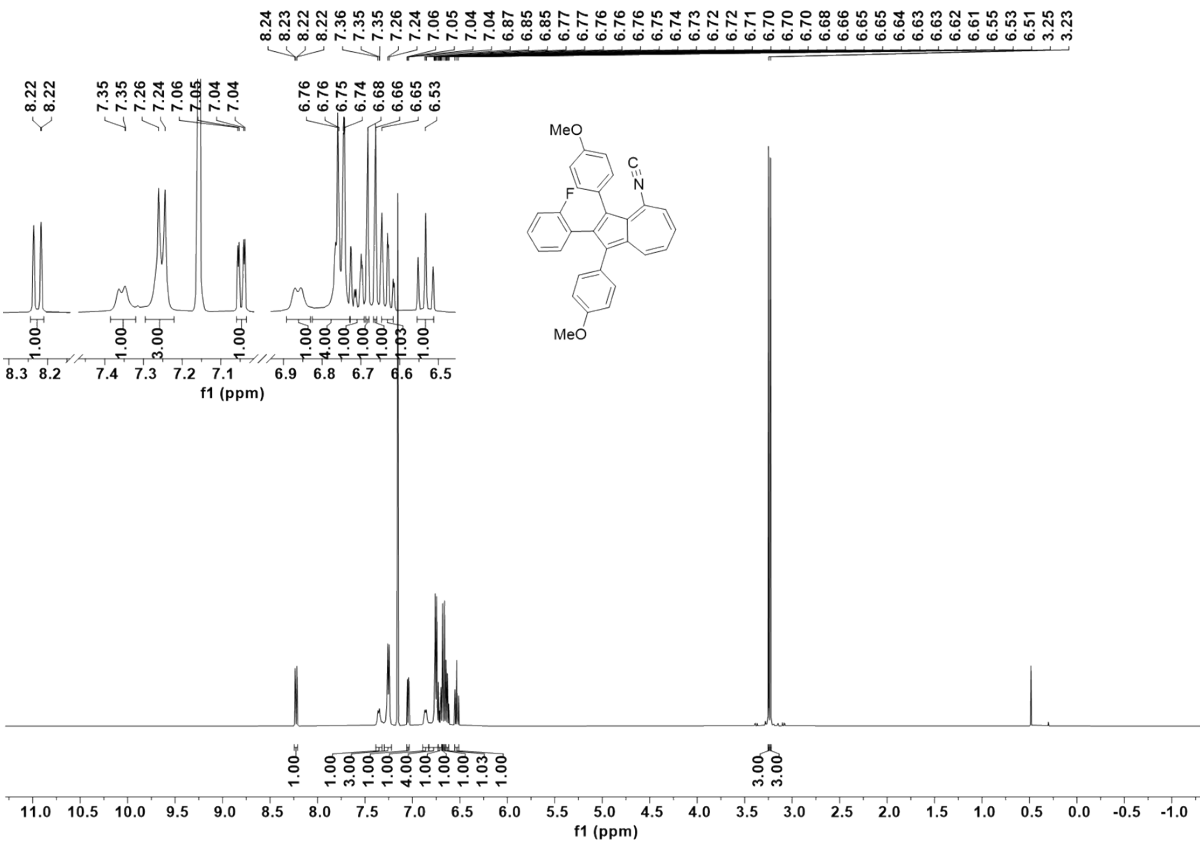

Figure S17. **$^{1}H\{^{19}F\}$ NMR** Spectrum (500 MHz, C_6D_6, 295 K) of **10a**.

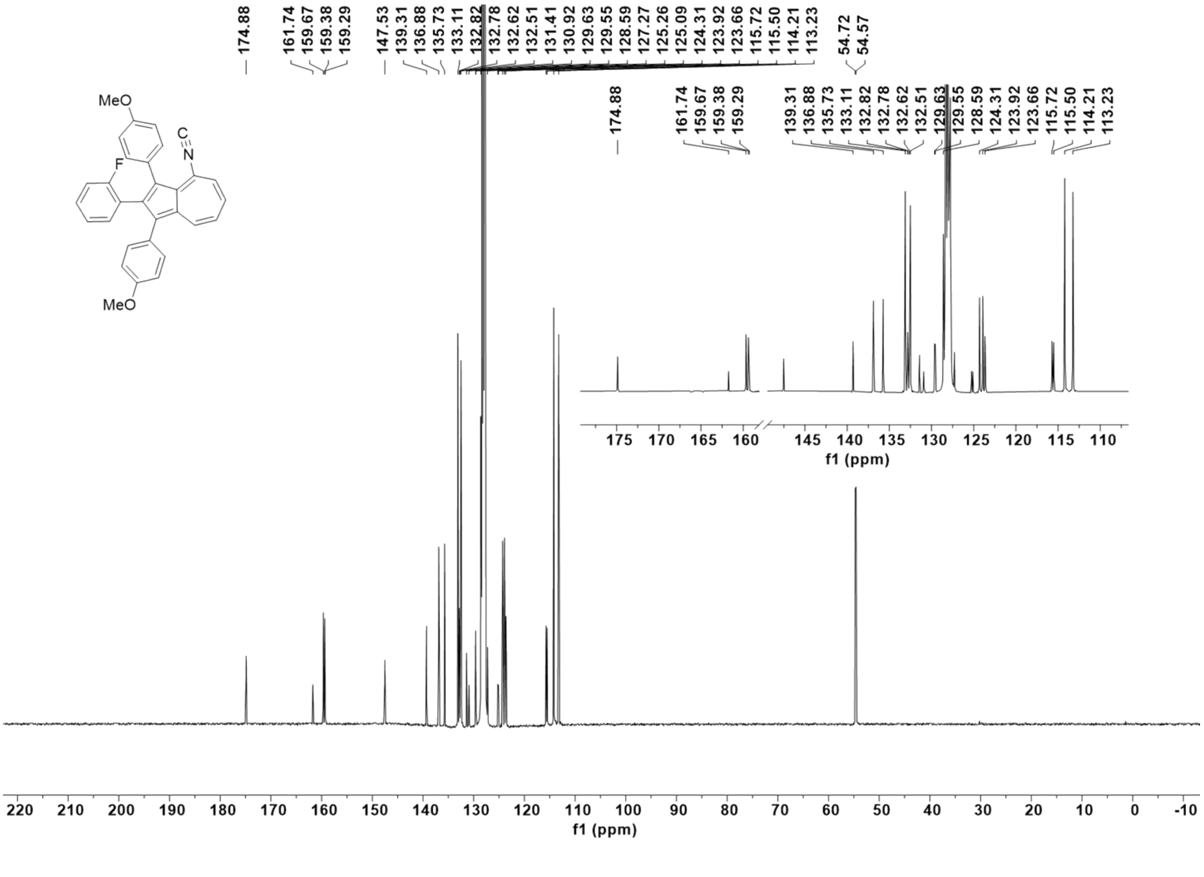

Figure S18. **$^{13}C\{^{19}F,^{1}H\}$ NMR** Spectrum (101 MHz, C_6D_6, 295 K) of **10a**.

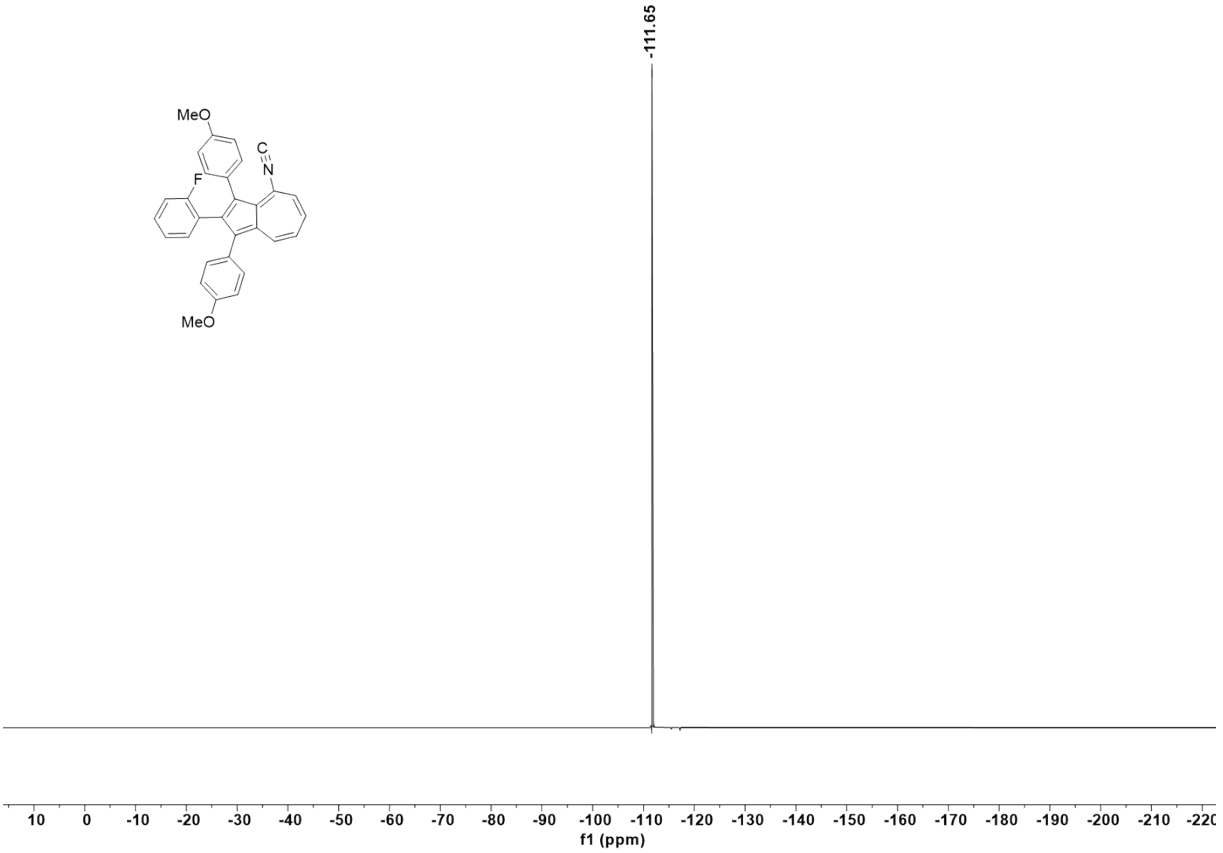

Figure S19. **$^{19}F\{^1H\}$ NMR** Spectrum (471 MHz, C_6D_6, 295 K) of **10a**.

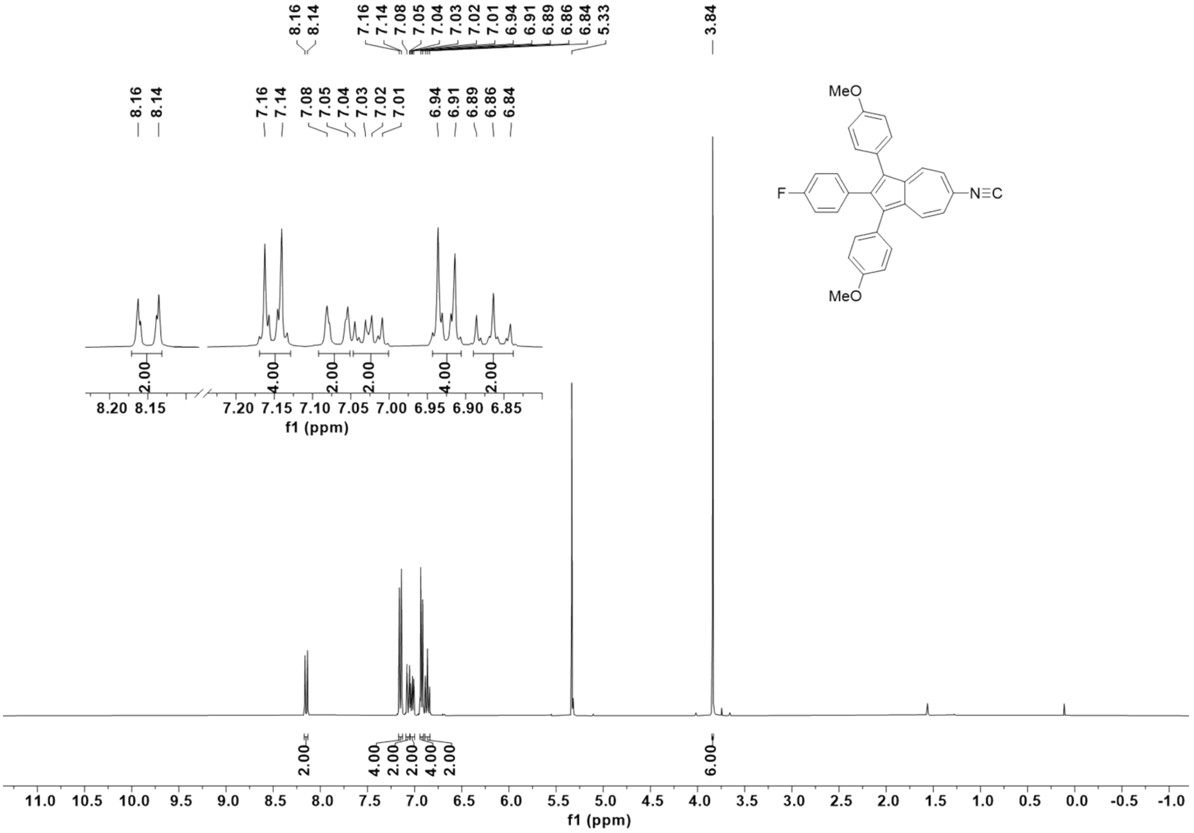

Figure S20. **$^1H\{^{19}F\}$ NMR** Spectrum (400 MHz, CD_2Cl_2, 295 K) of **10b**.

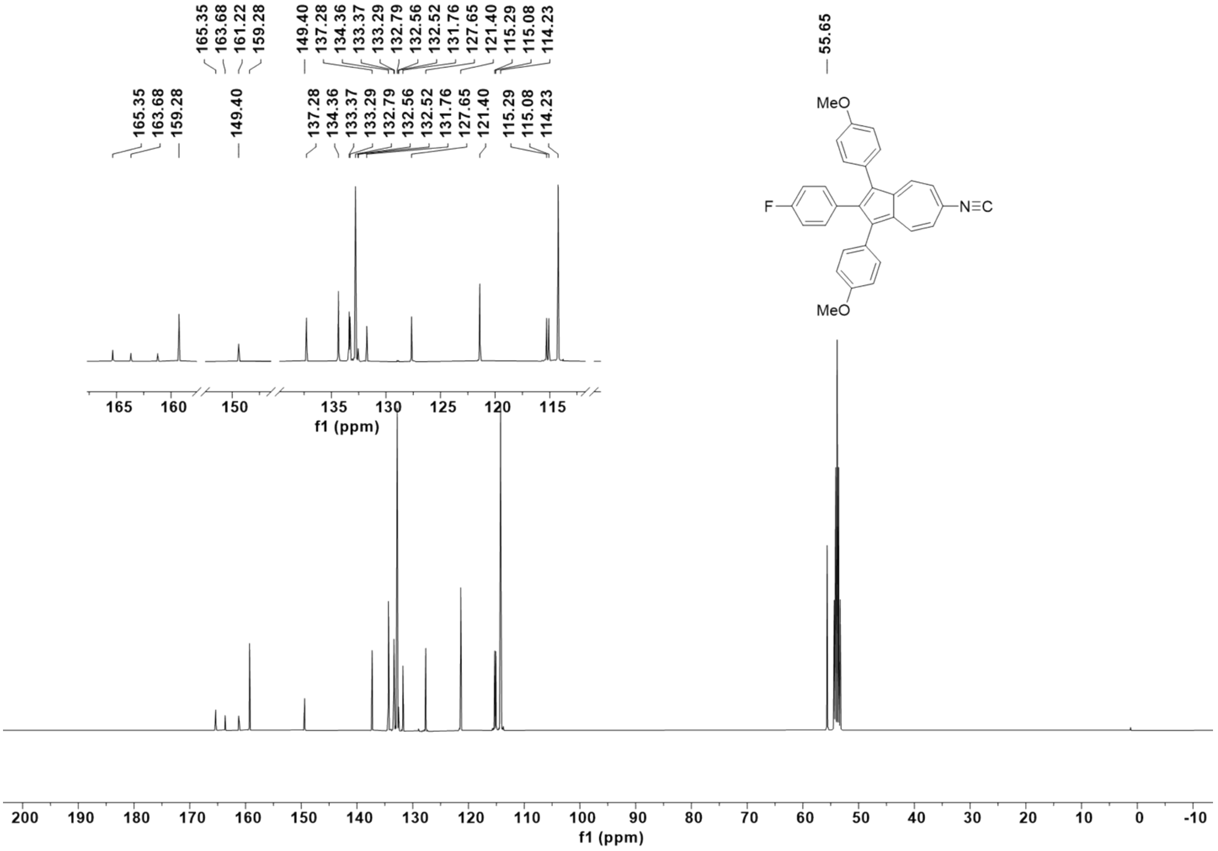

Figure S21. **$^{13}C\{^{19}F,^{1}H\}$ NMR** Spectrum (101 MHz, CD_2Cl_2, 295 K) of **10b**.

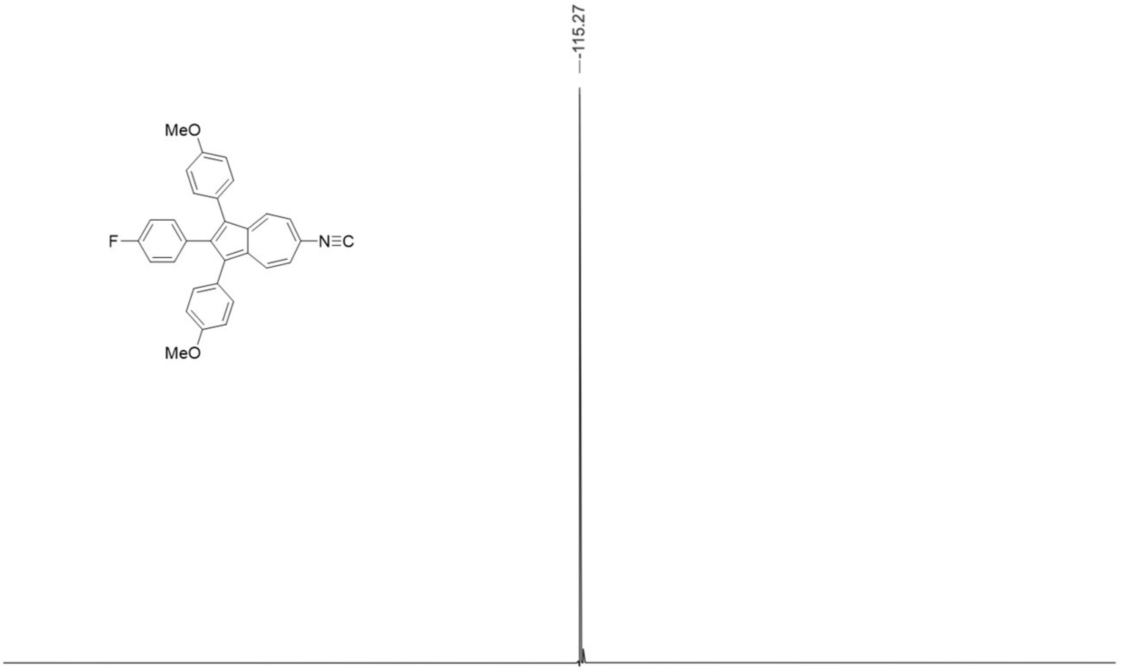

Figure S22. **$^{19}F\{^{1}H\}$ NMR** Spectrum (283 MHz, CD_2Cl_2, 298 K) of **10b**.

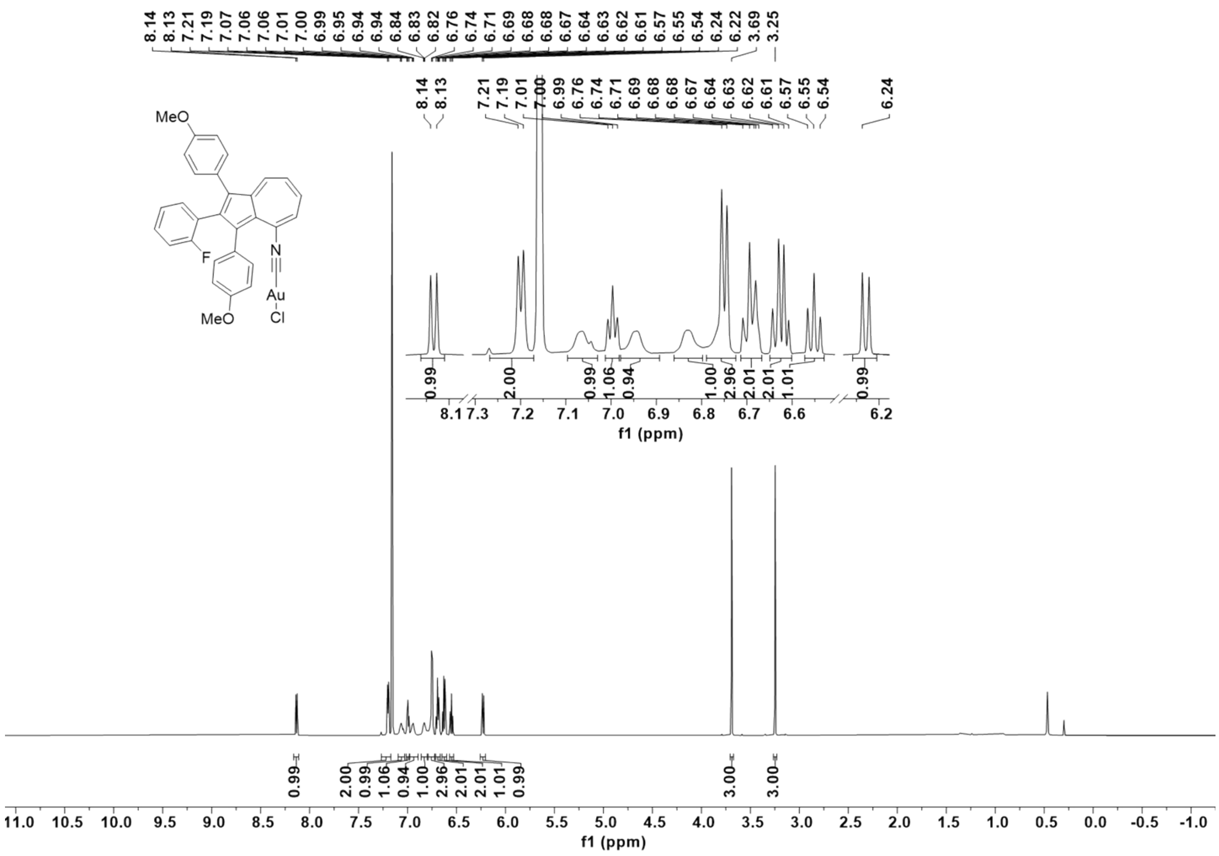

Figure S23. **^{1}H{^{19}F} NMR** Spectrum (700 MHz, C_6D_6, 295 K) of **12a**.

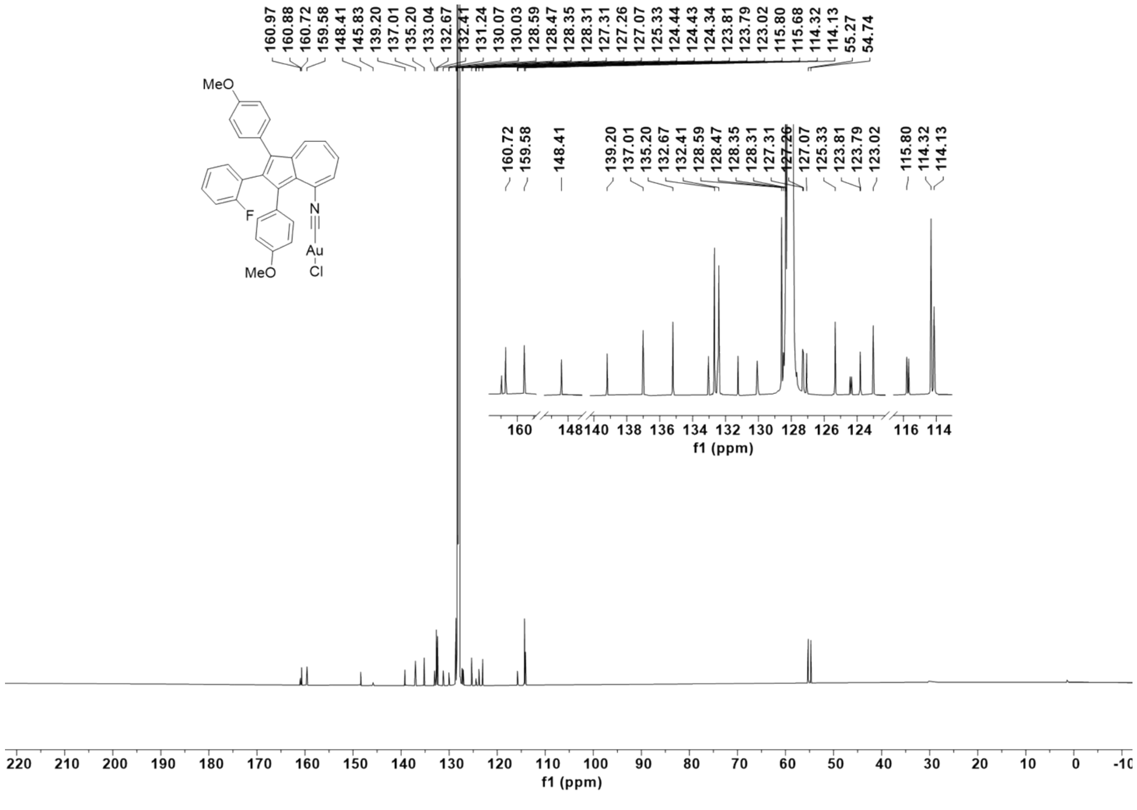

Figure S24. **^{13}C{^{19}F,^{1}H} NMR** Spectrum (176 MHz, C_6D_6, 295 K) of **12a**.

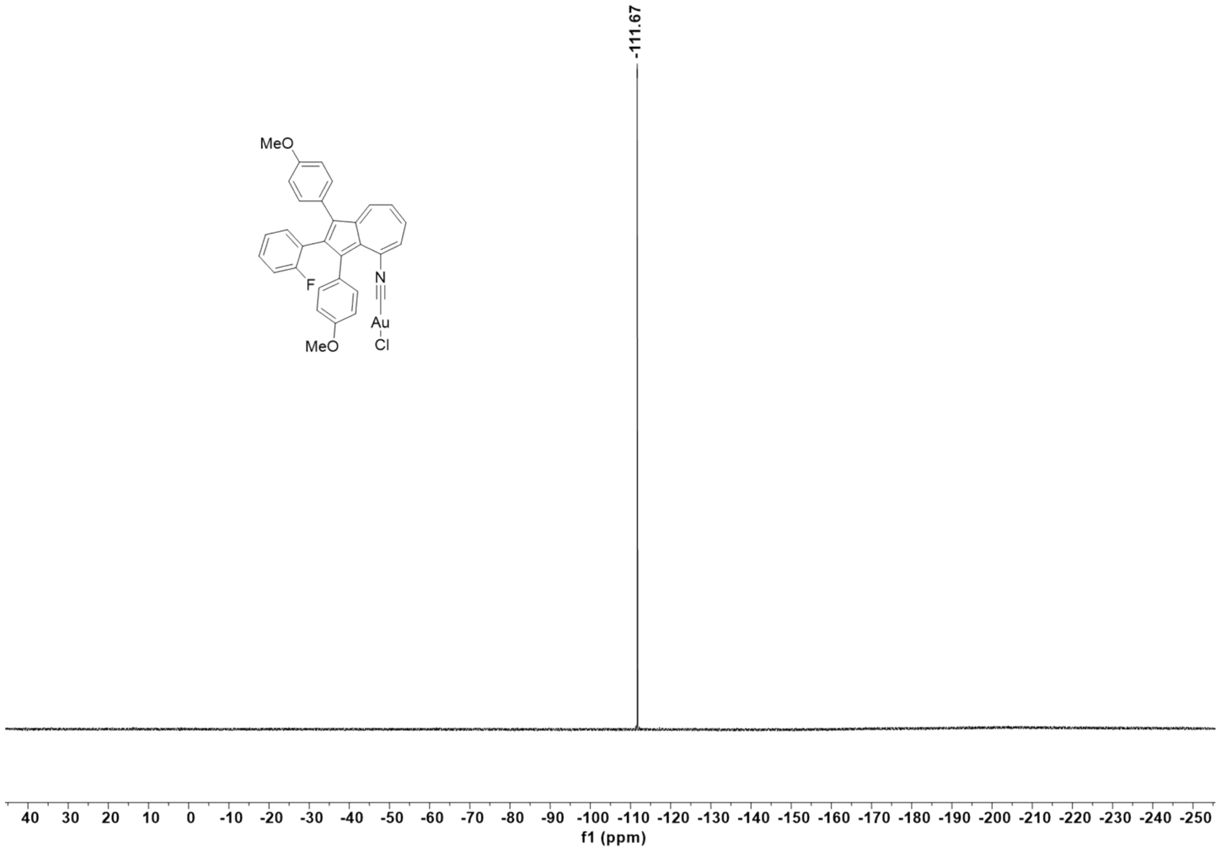

Figure S25. ^{19}**F{**^{1}**H} NMR** Spectrum (283 MHz, C_6D_6, 295 K) of **12a**.

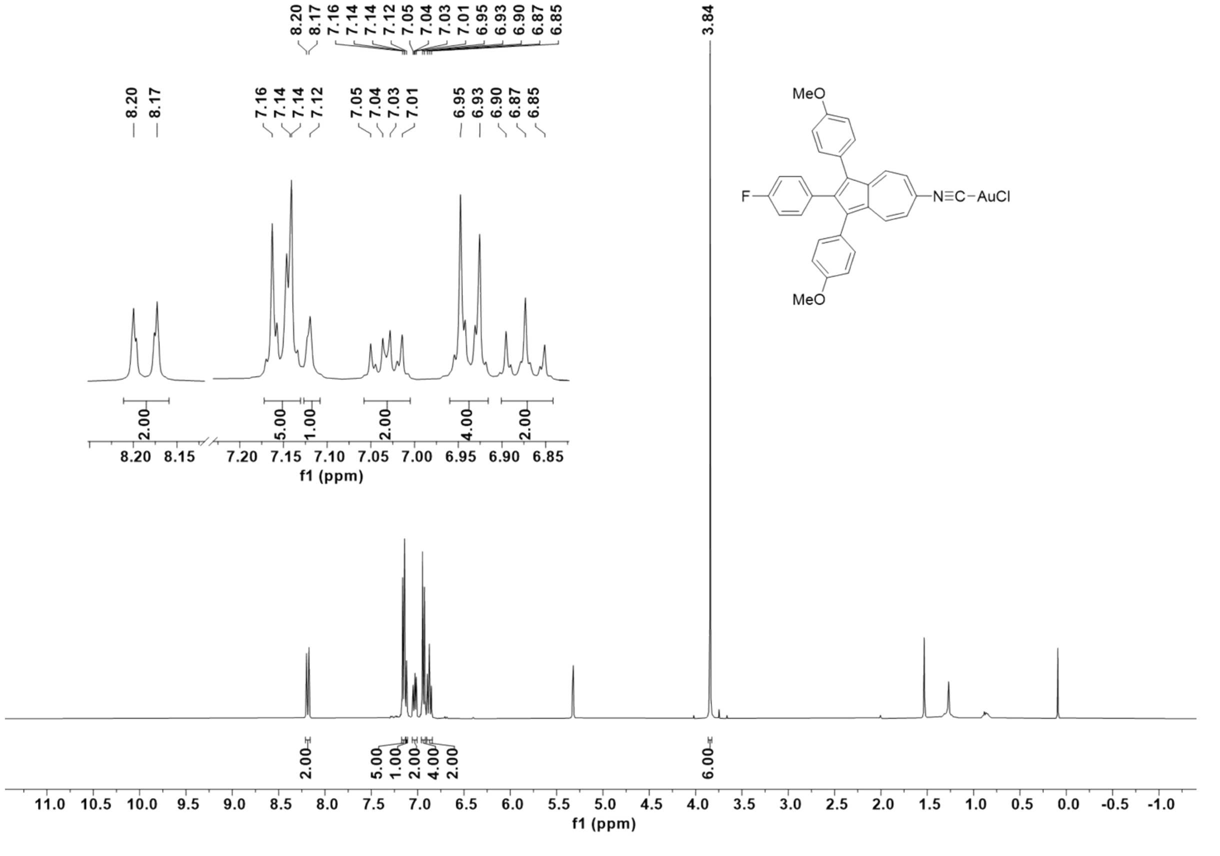

Figure S26. ^{1}**H{**^{19}**F} NMR** Spectrum (400 MHz, CD_2Cl_2, 295 K) of **12b**.

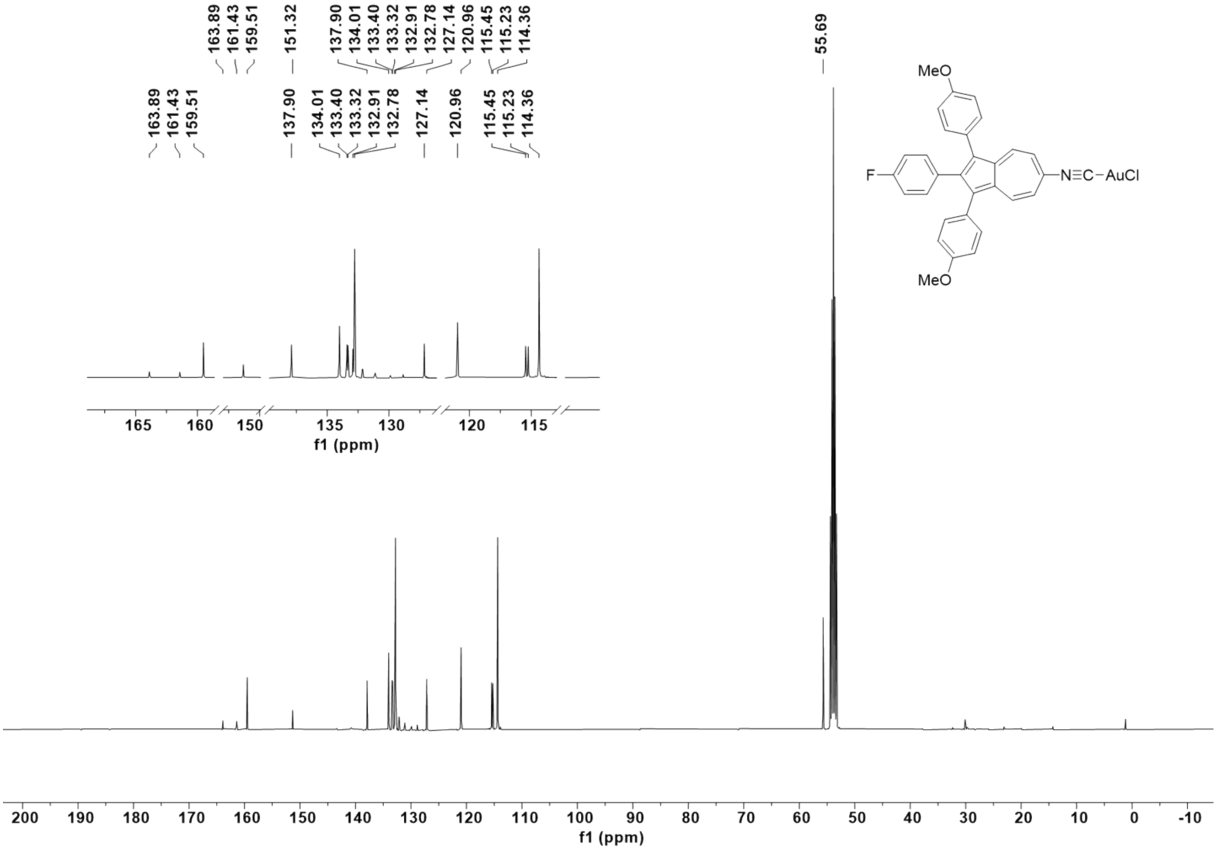

Figure S27. ^{13}**C{**^{19}**F,**^{1}**H} NMR** Spectrum (101 MHz, CD_2Cl_2, 295 K) of **12b**.

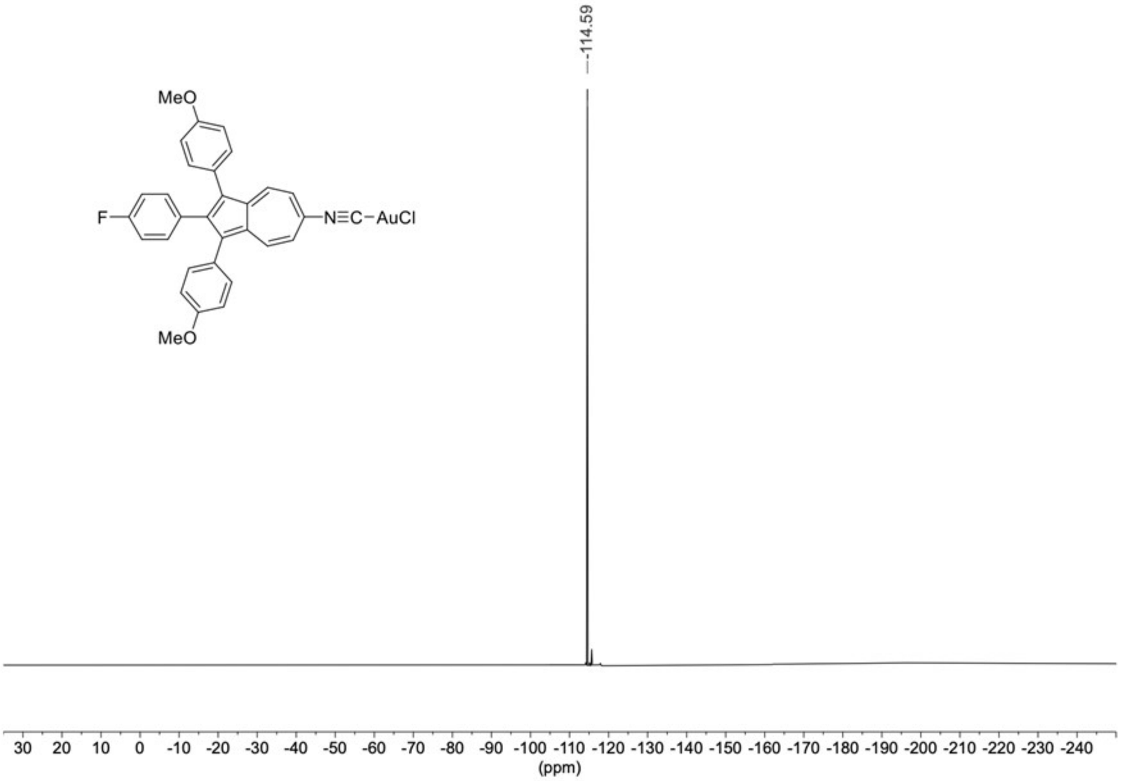

Figure S28. ^{19}**F{**^{1}**H} NMR** Spectrum (283 MHz, CD_2Cl_2, 298 K) of **12b**.

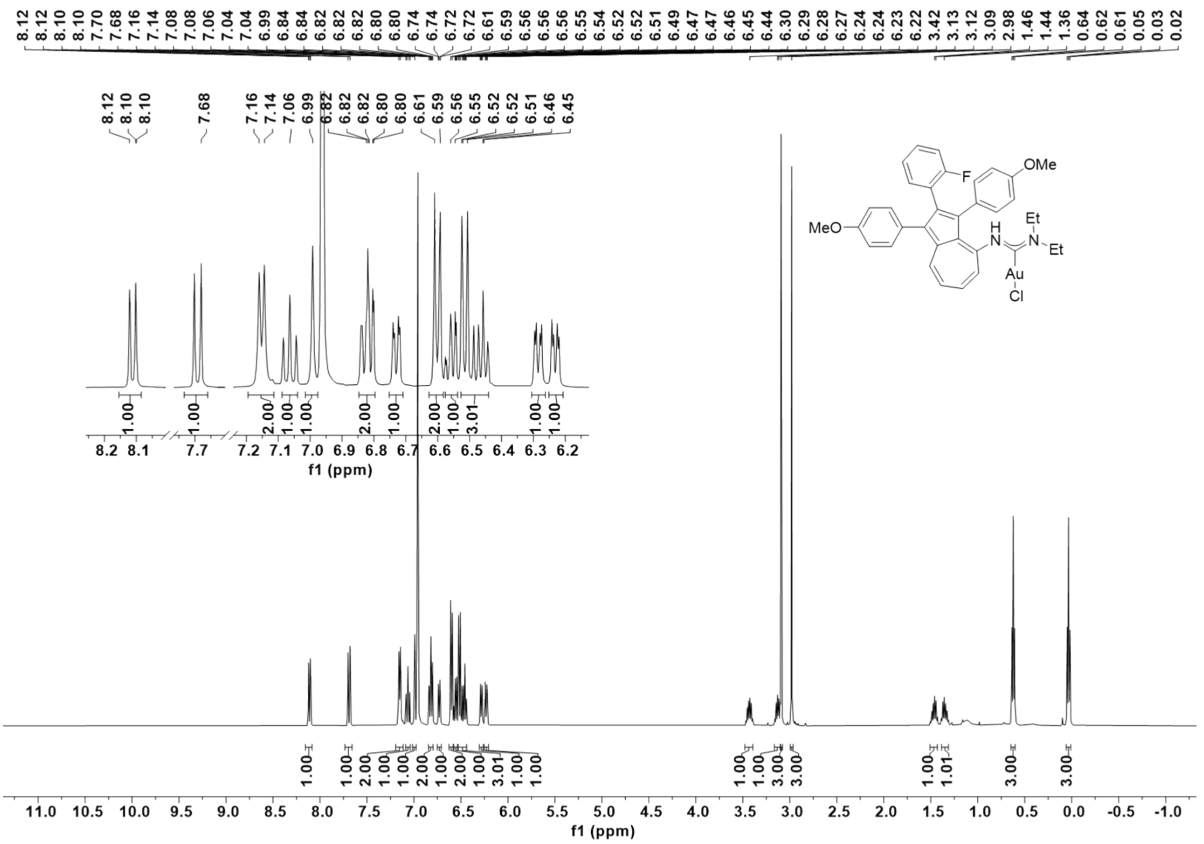

Figure S29. **^{1}H{^{19}F} NMR** Spectrum (500 MHz, C_6D_6, 295 K) of **14aa**.

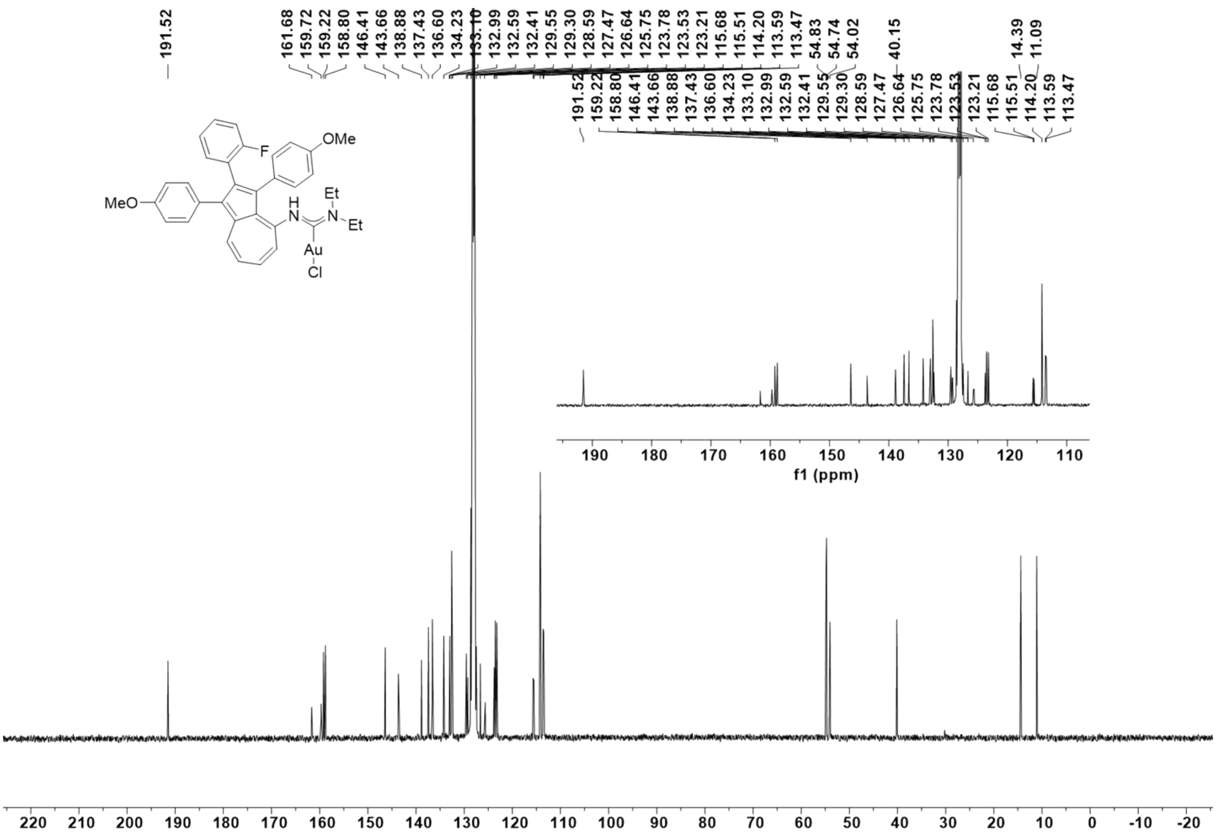

Figure S30. **^{13}C{^{19}F,^{1}H} NMR** Spectrum (126 MHz, C_6D_6, 295 K) of **14aa.**

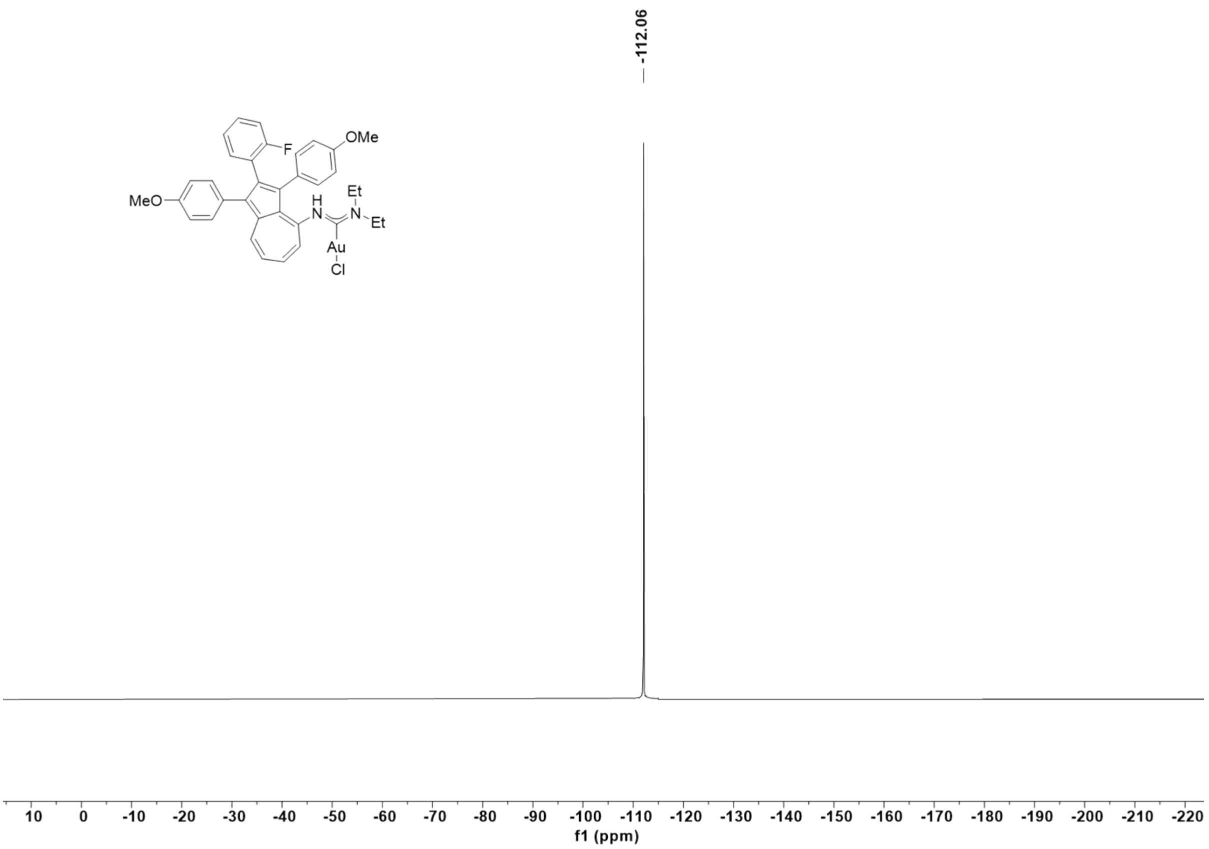

Figure S31. ^{19}**F{**^{1}**H} NMR** Spectrum (471 MHz, C_6D_6, 295 K) of **14aa**.

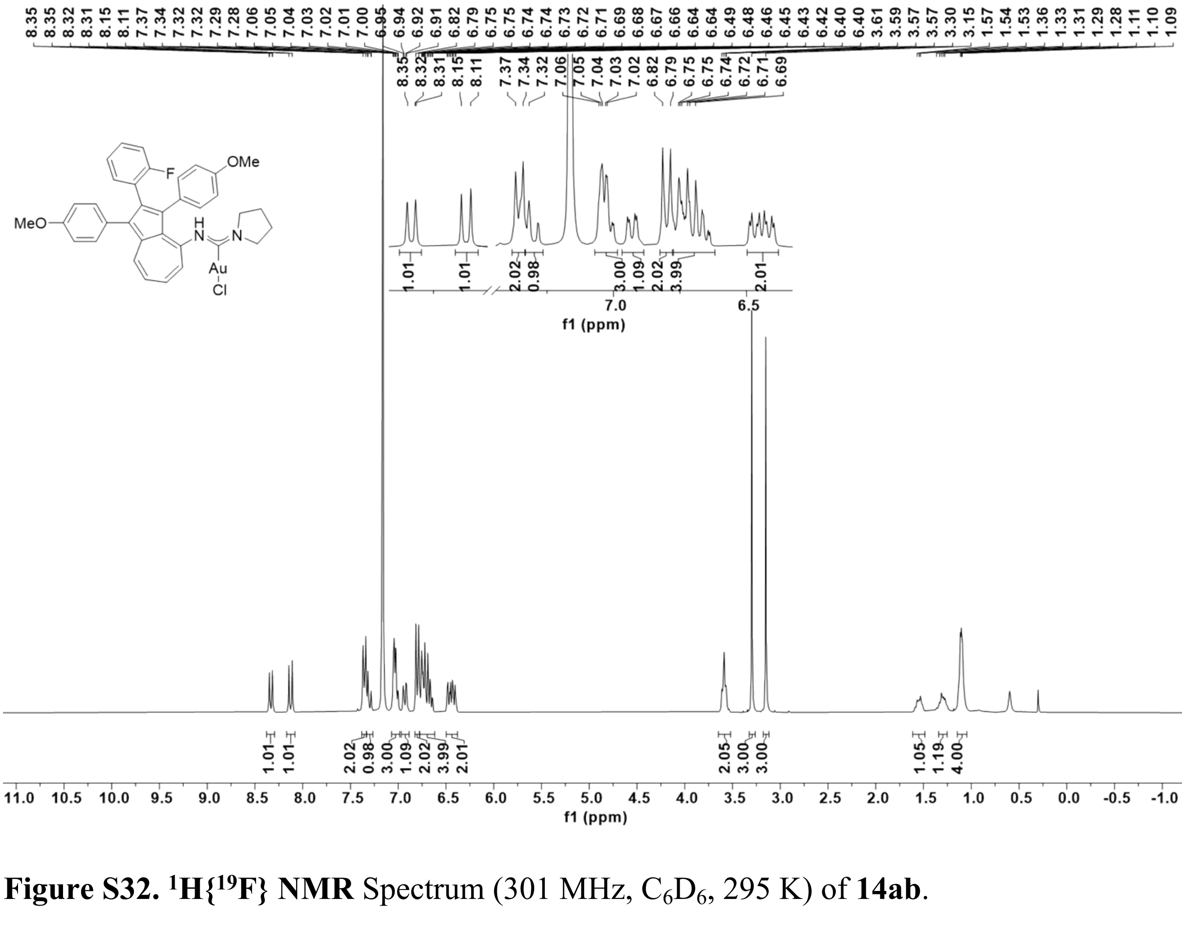

Figure S32. ^{1}**H{**^{19}**F} NMR** Spectrum (301 MHz, C_6D_6, 295 K) of **14ab**.

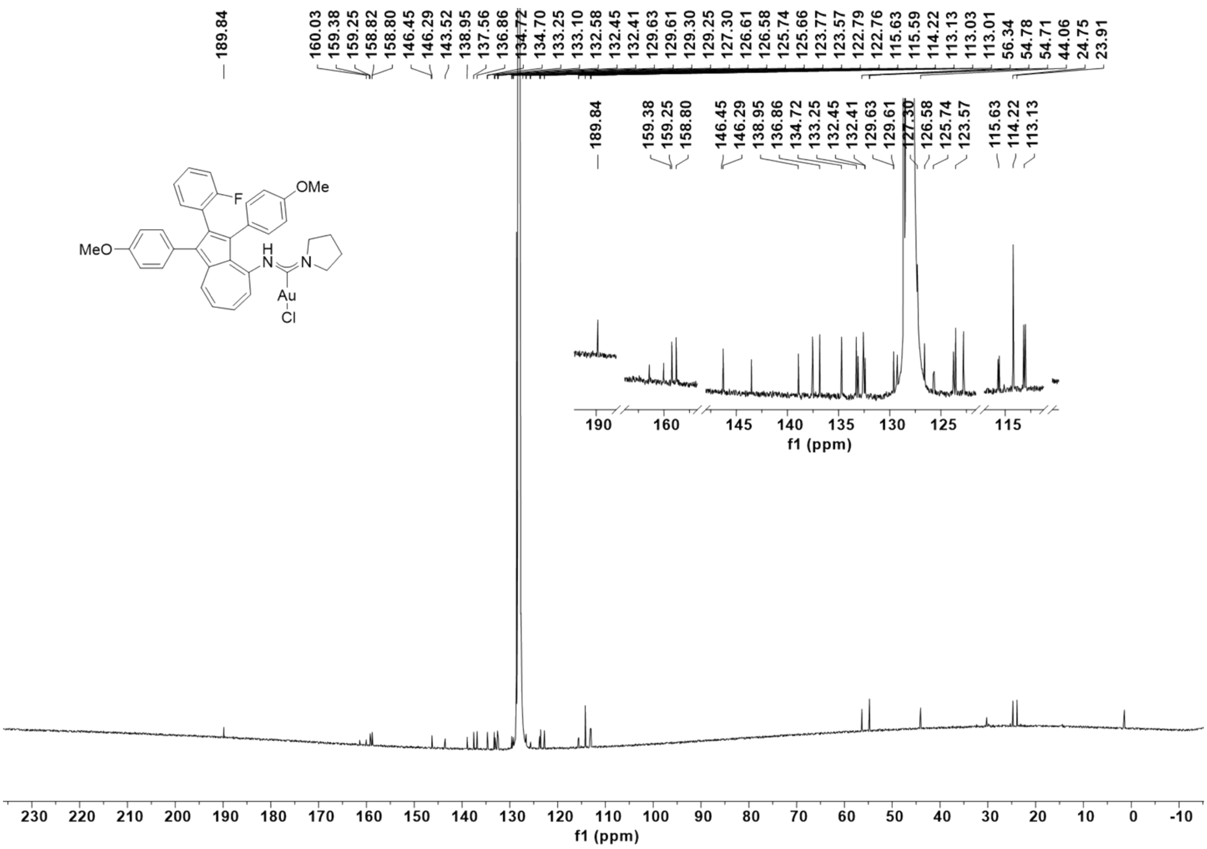

Figure S33. ^{13}**C{**^{19}**F,**^{1}**H} NMR** Spectrum (176 MHz, C_6D_6, 295 K) of **14ab**.

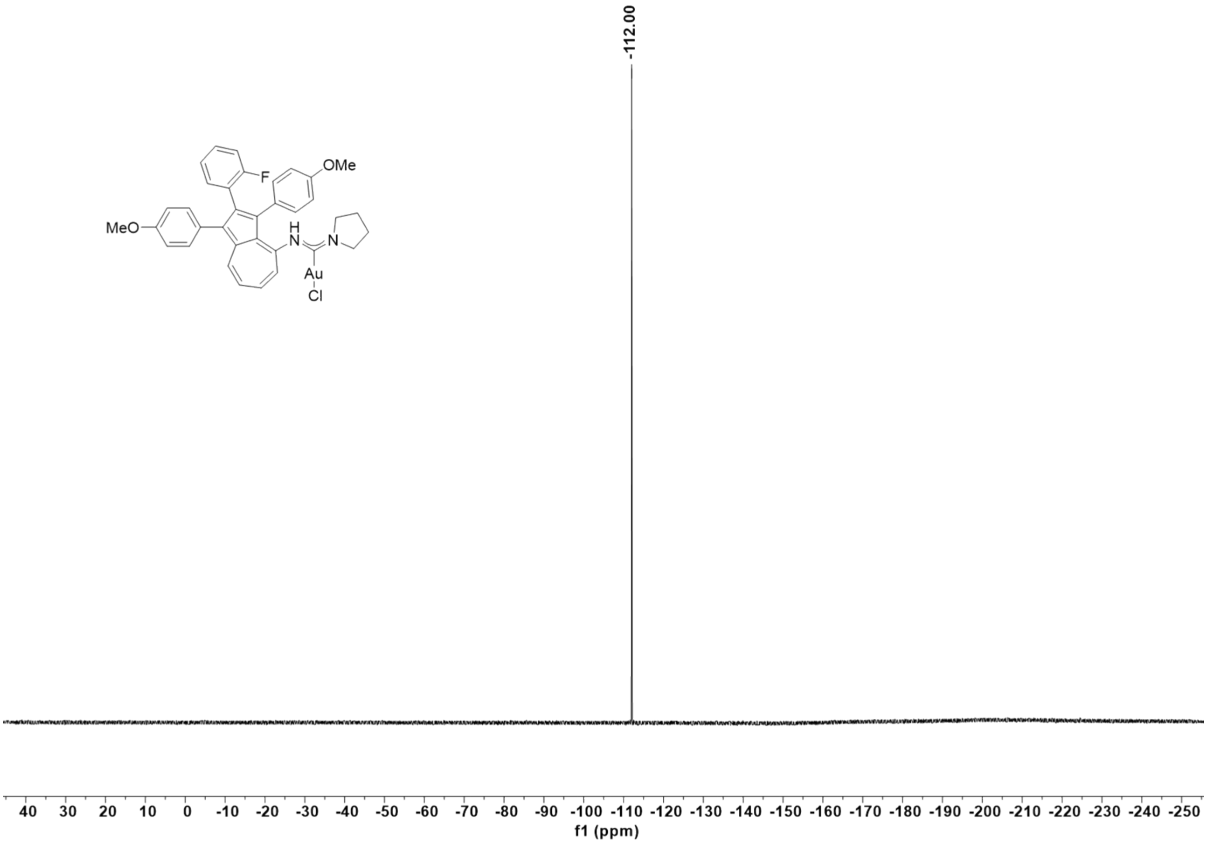

Figure S34. ^{19}**F{**^{1}**H} NMR** Spectrum (283 MHz, C_6D_6, 295 K) of **14ab**.

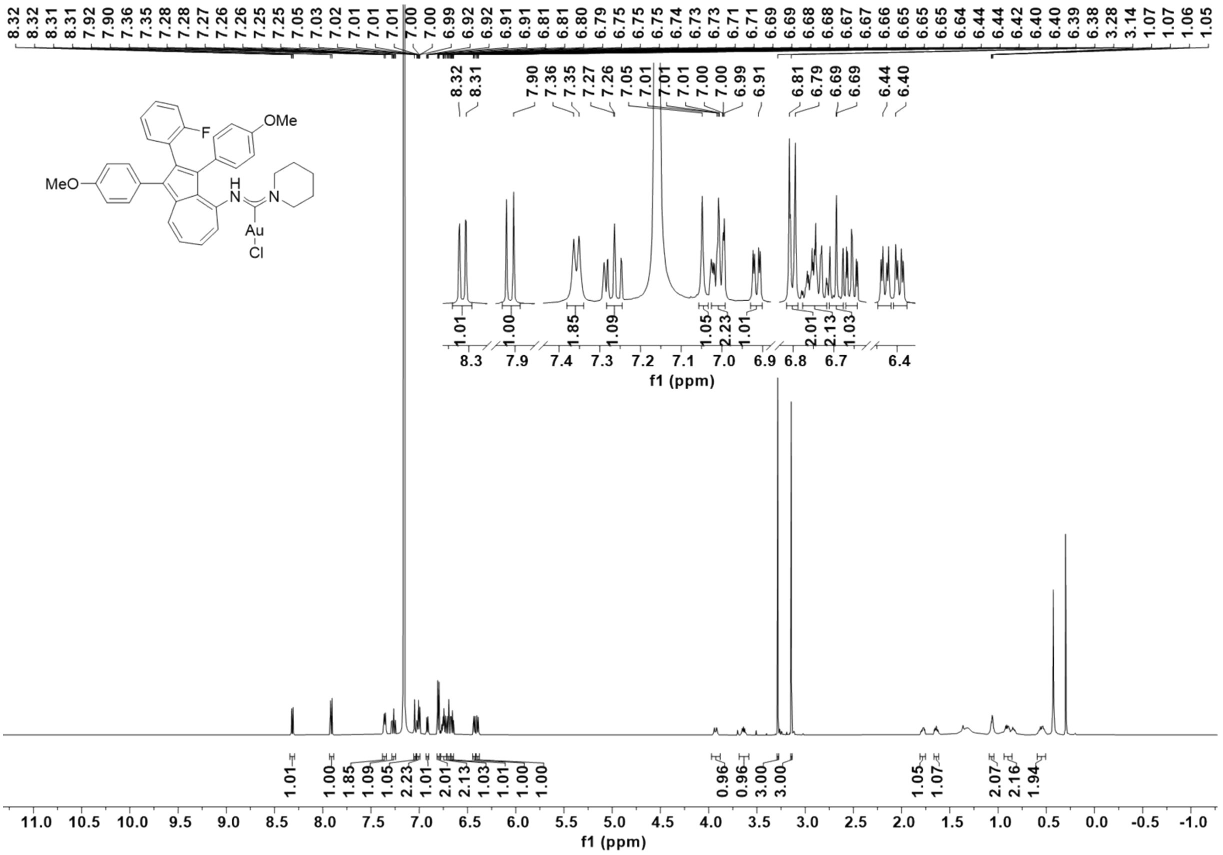

Figure S35. **^{1}H{^{19}F} NMR** Spectrum (600 MHz, C_6D_6, 295 K) of **14ac**.

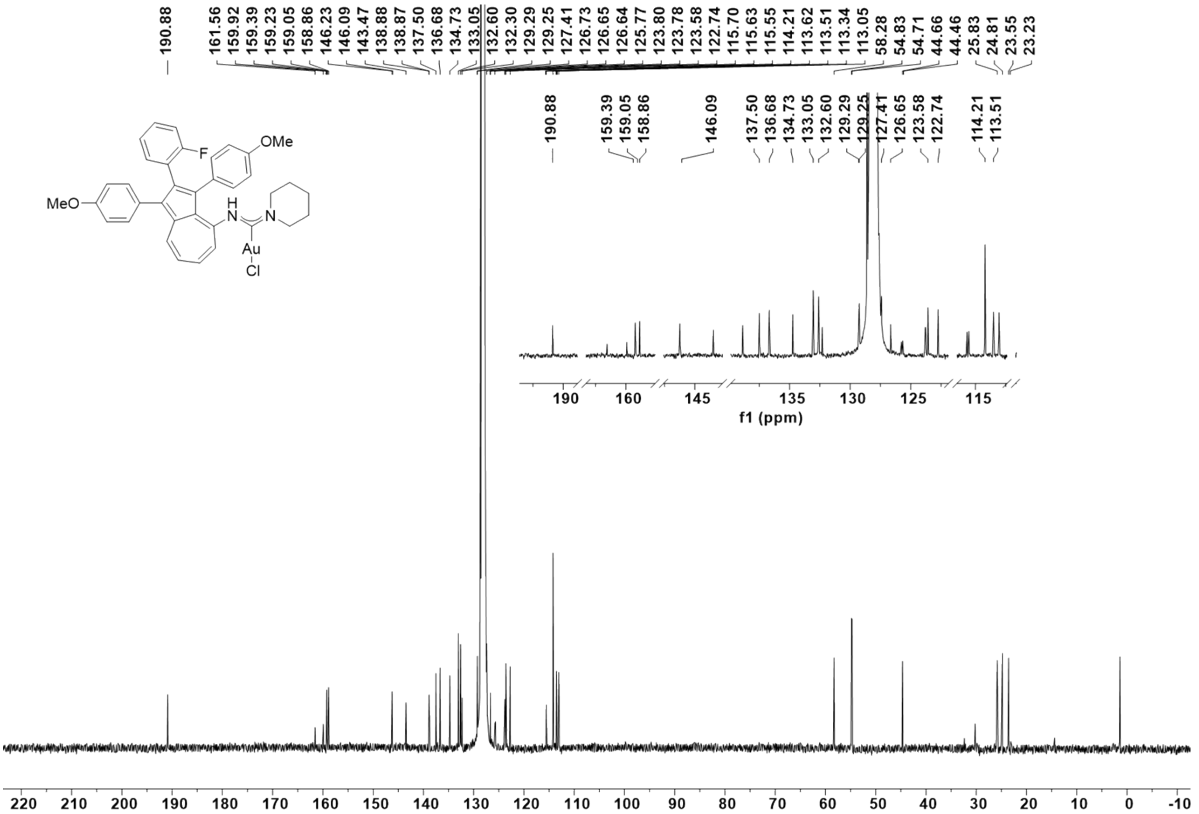

Figure S36. **^{13}C{^{19}F,^{1}H} NMR** Spectrum (151 MHz, C_6D_6, 295 K) of **14ac**.

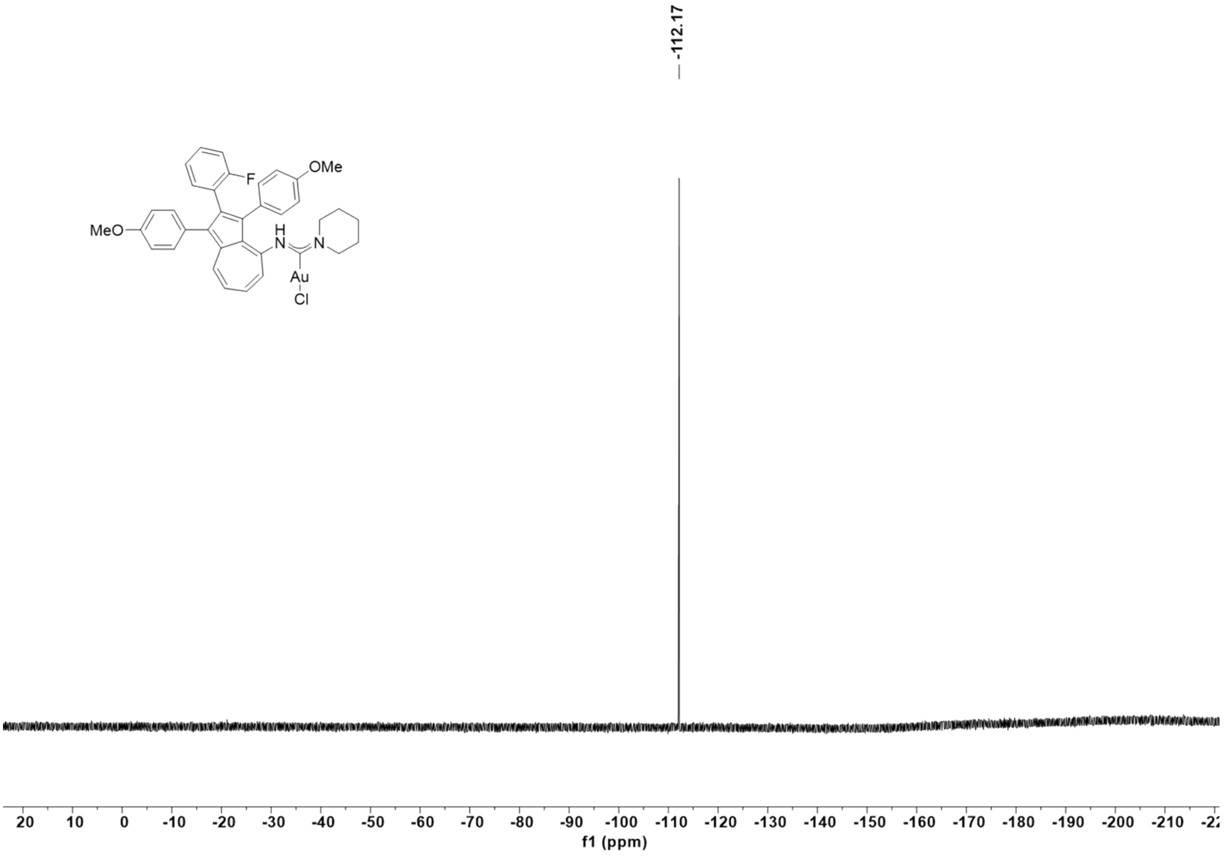

Figure S37. $^{19}F\{^{1}H\}$ NMR Spectrum (283 MHz, C_6D_6, 295 K) of **14ac**.

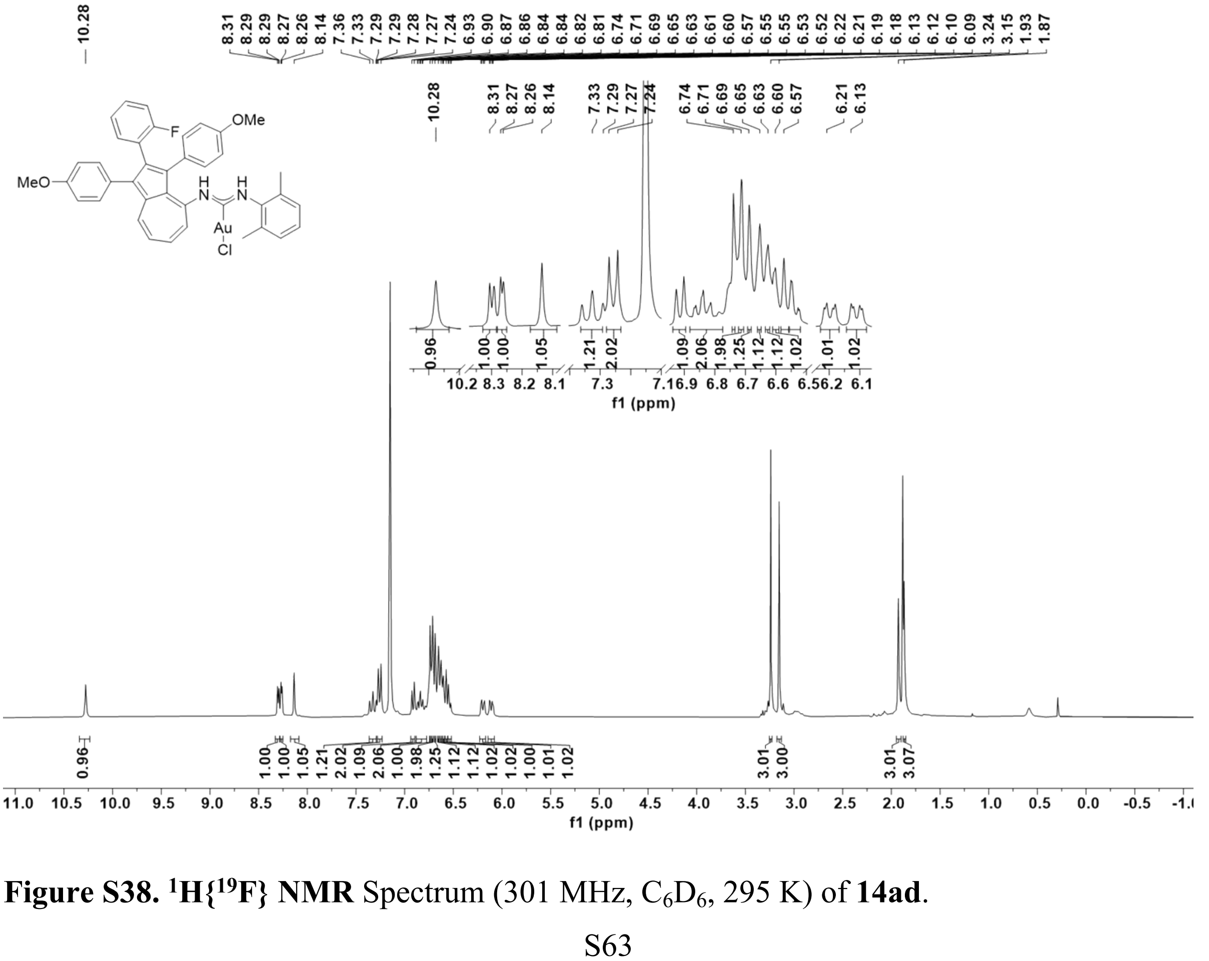

Figure S38. $^{1}H\{^{19}F\}$ NMR Spectrum (301 MHz, C_6D_6, 295 K) of **14ad**.

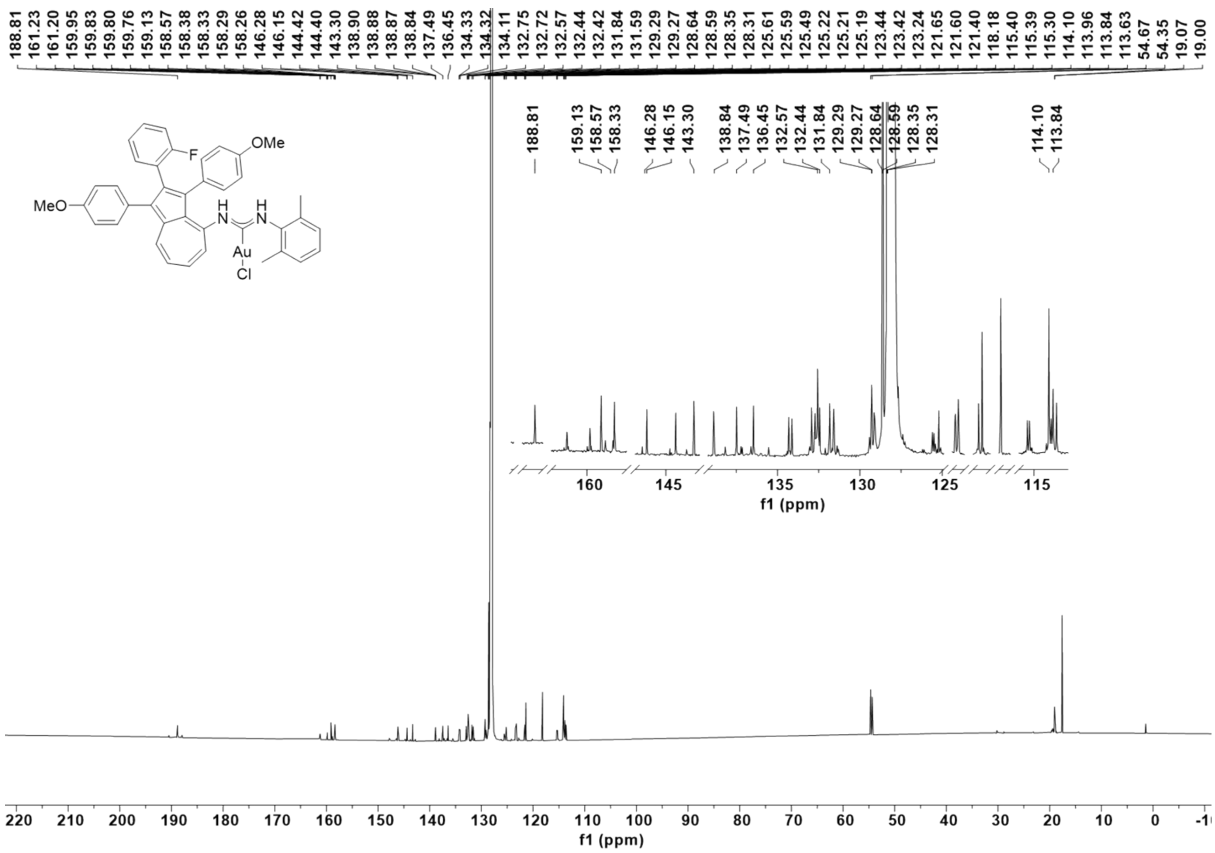

Figure S39. $^{13}C\{^{19}F,^{1}H\}$ **NMR** Spectrum (176 MHz, C_6D_6, 295 K) of **14ad**.

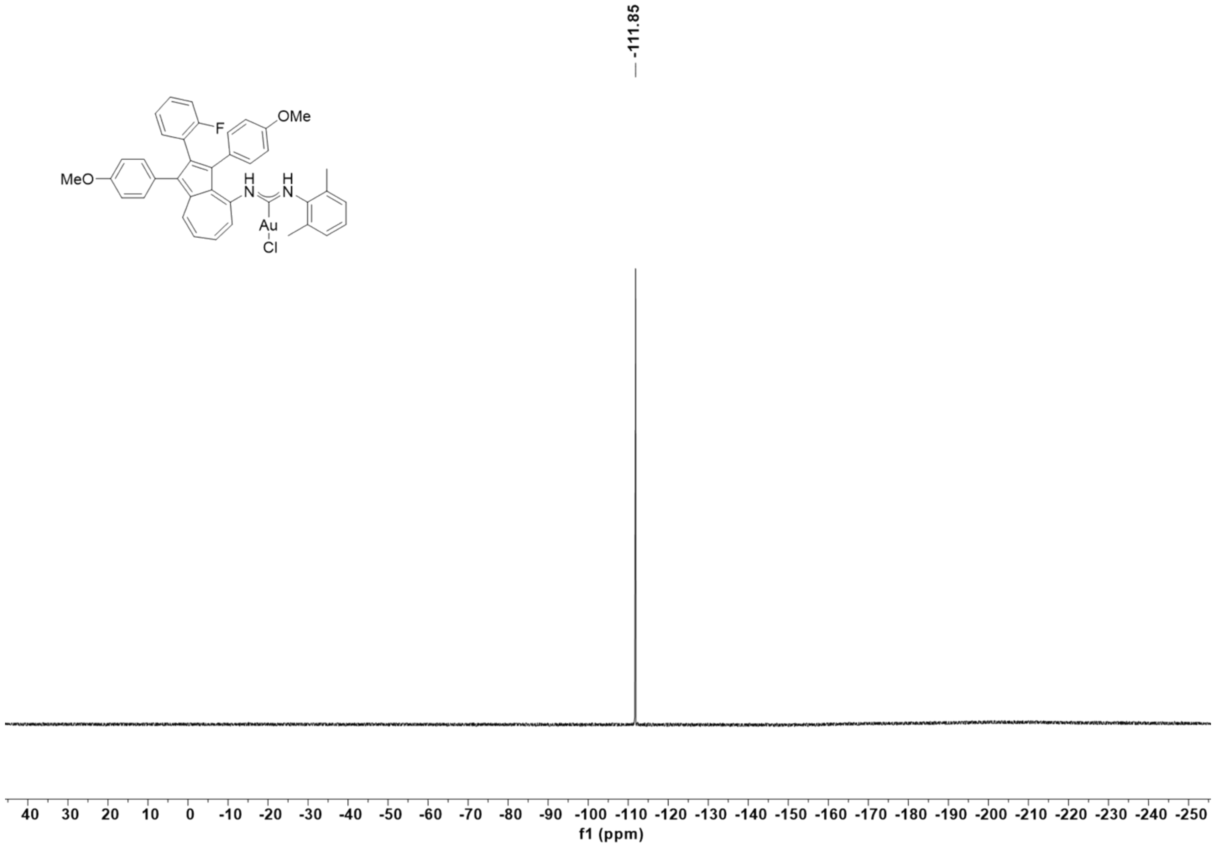

Figure S40. $^{19}F\{^{1}H\}$ **NMR** Spectrum (283 MHz, C_6D_6, 295 K) of **14ad**.

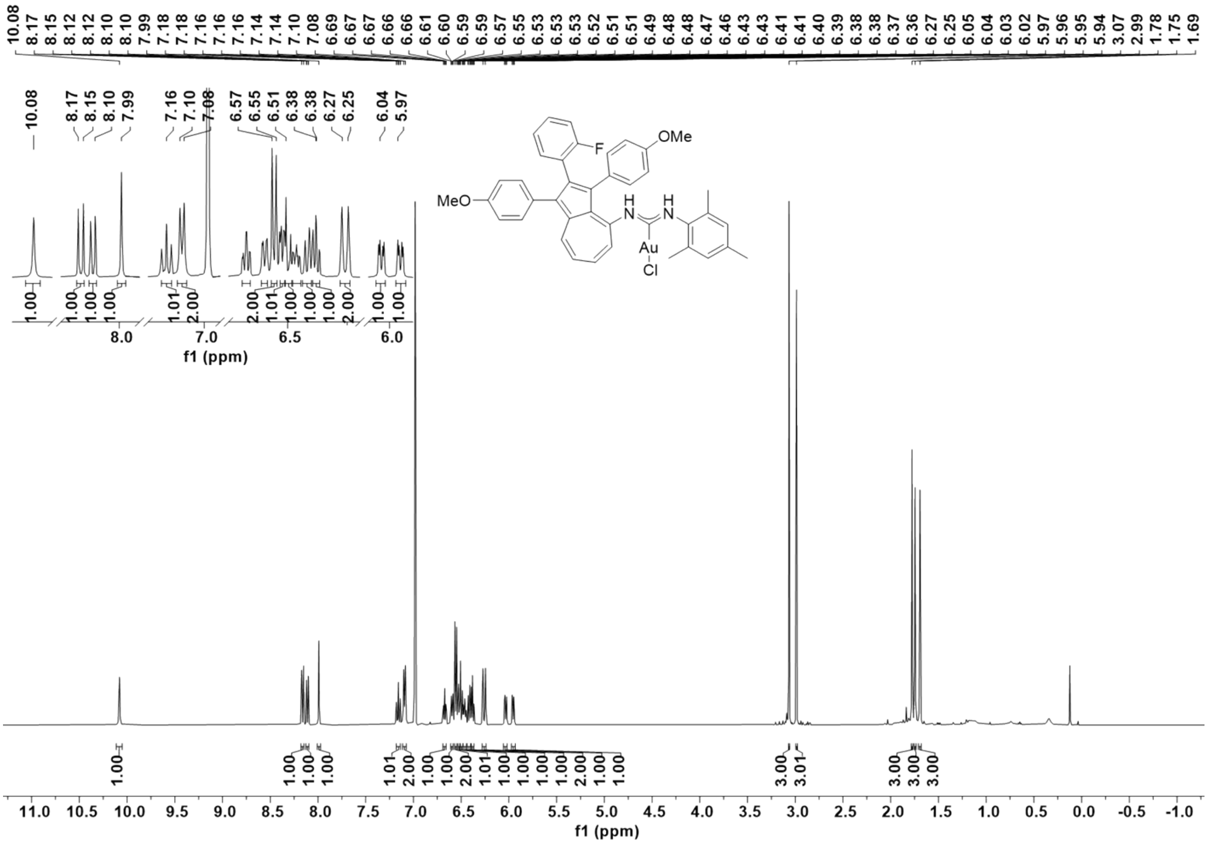

Figure S41. **$^{1}H\{^{19}F\}$ NMR** Spectrum (500 MHz, C_6D_6, 295 K) of **14ae**.

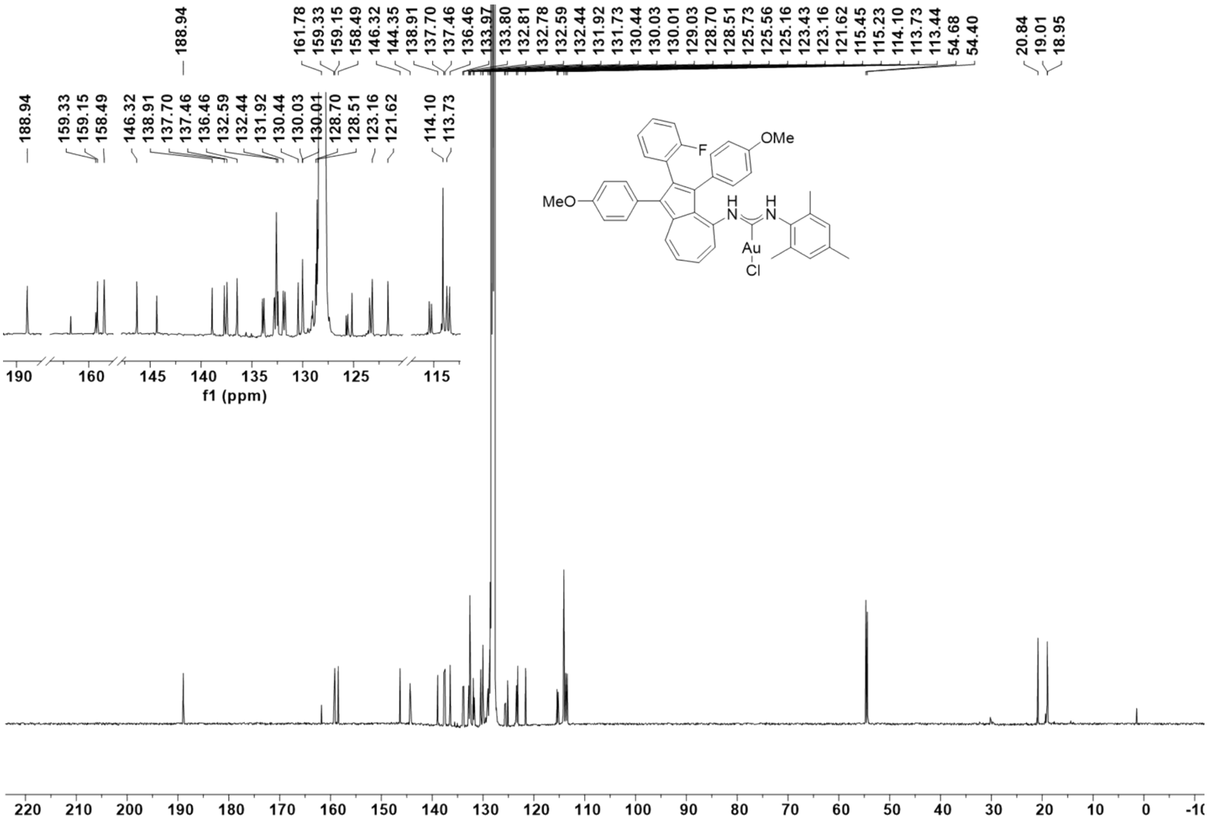

Figure S42. $^{13}C\{^{19}F,^{1}H\}$ NMR Spectrum (101 MHz, C_6D_6, 295 K) of **14ae**.

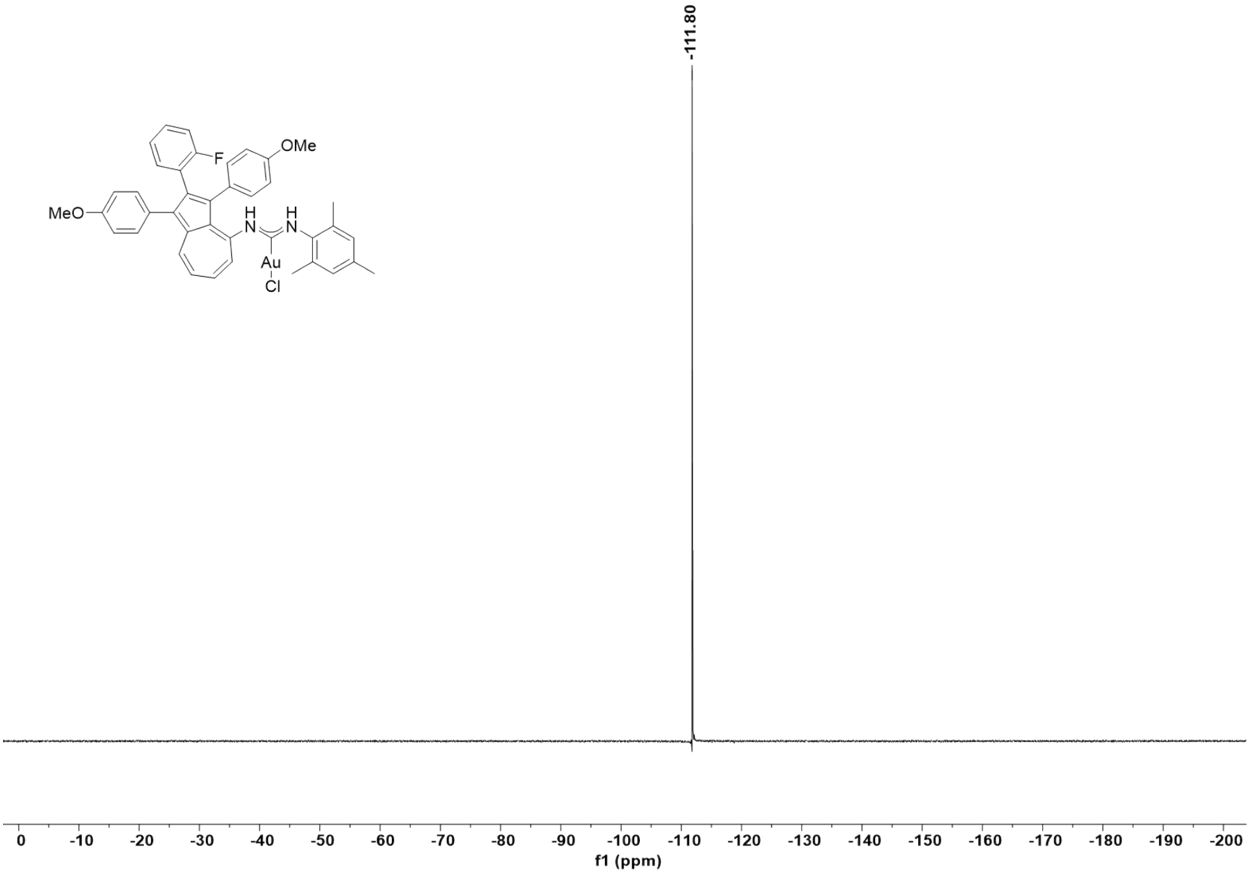

Figure S43. $^{19}F\{^{1}H\}$ **NMR** Spectrum (471 MHz, C_6D_6, 295 K) of **14ae**.

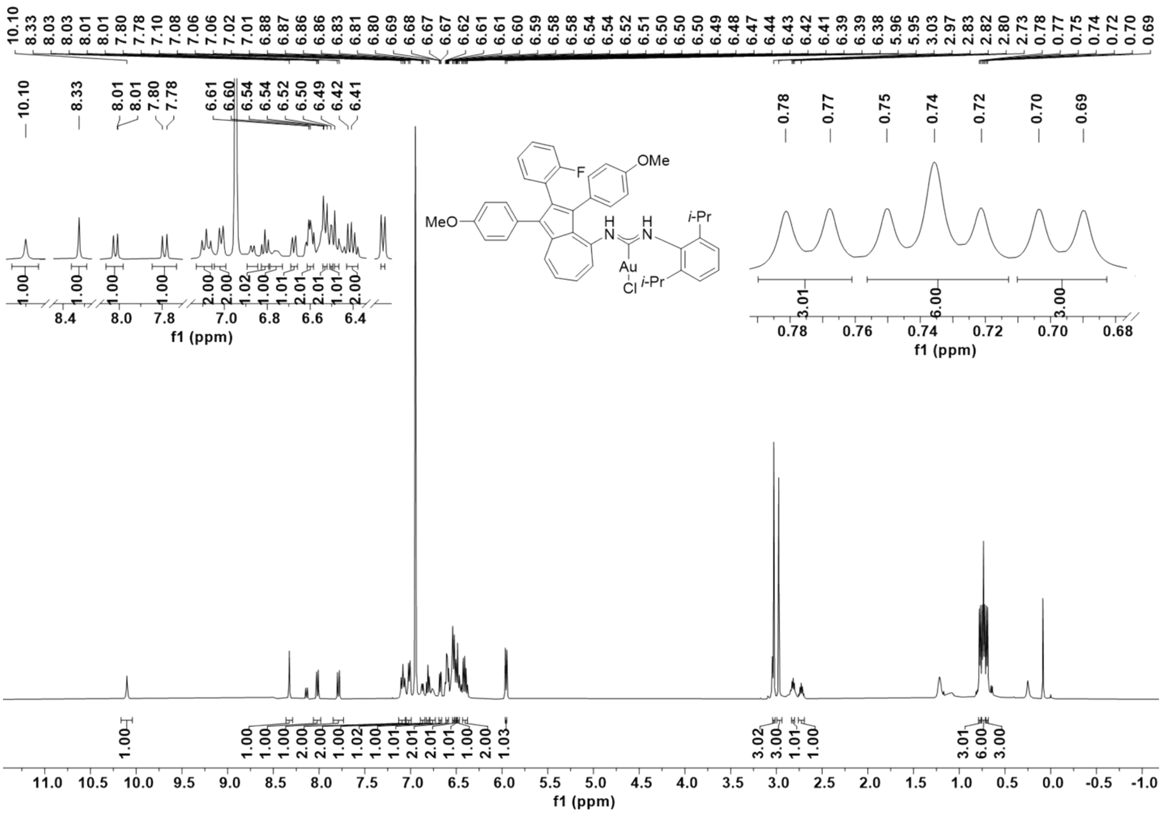

Figure S44. $^{1}H\{^{19}F\}$ **NMR** Spectrum (500 MHz, C_6D_6, 295 K) of **14af**.

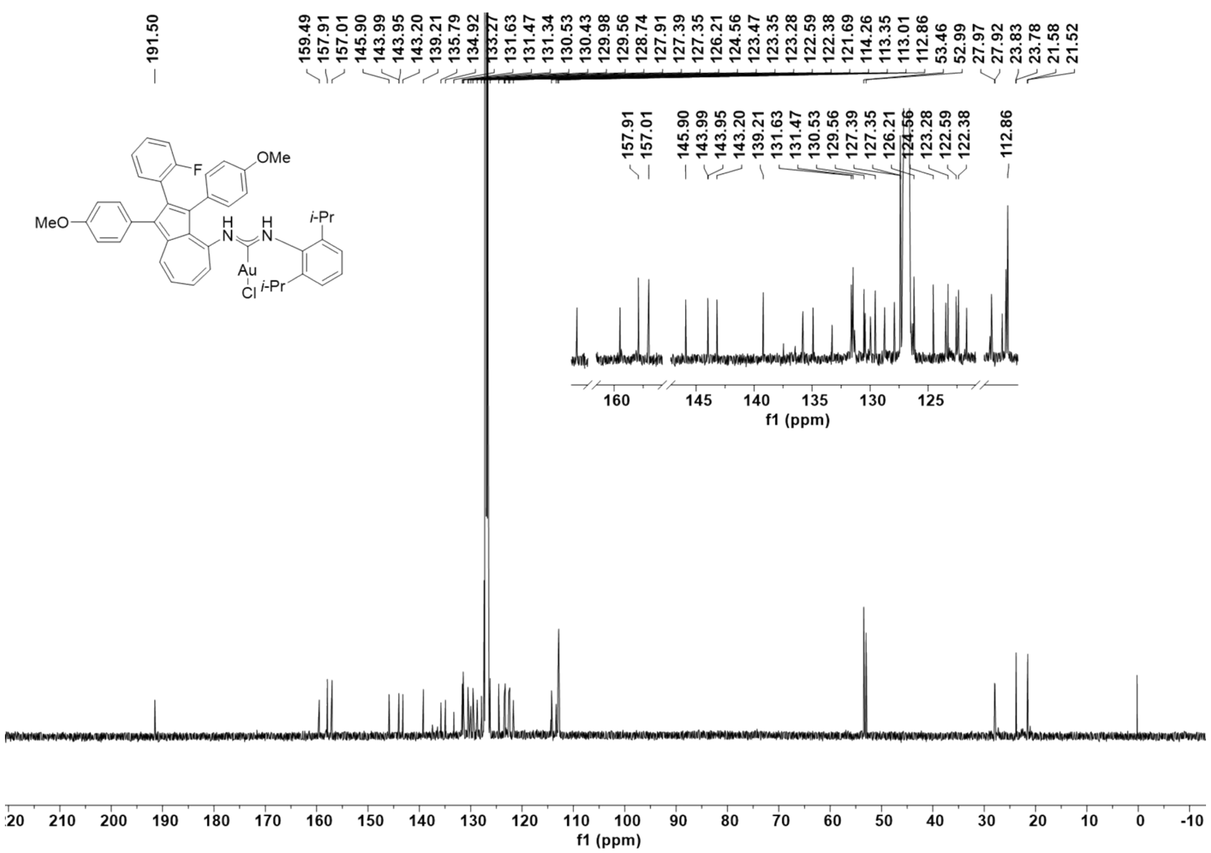

Figure S45. $^{13}C\{^{19}F,^{1}H\}$ **NMR** Spectrum (126 MHz, C_6D_6, 295 K) of **14af**.

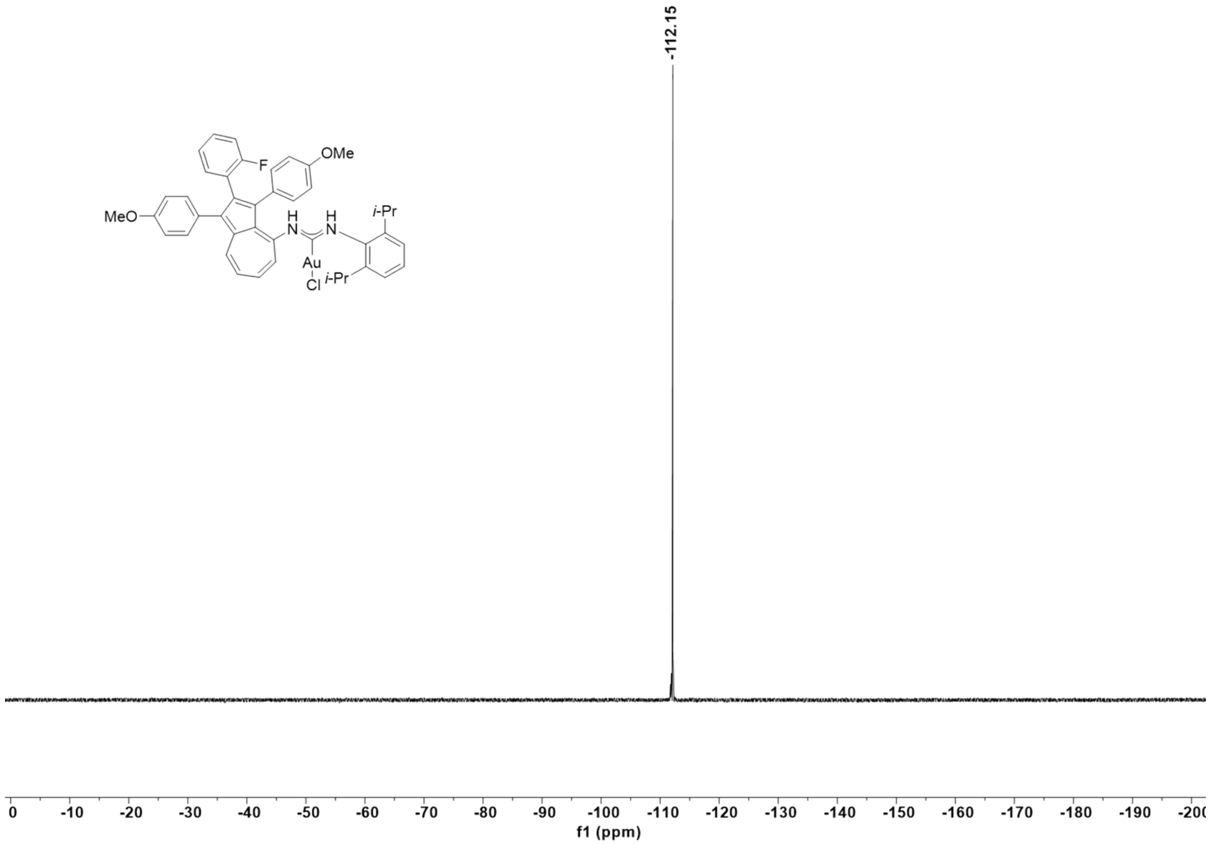

Figure S46. $^{19}F\{^{1}H\}$ **NMR** Spectrum (471 MHz, C_6D_6, 295 K) of **14af**.

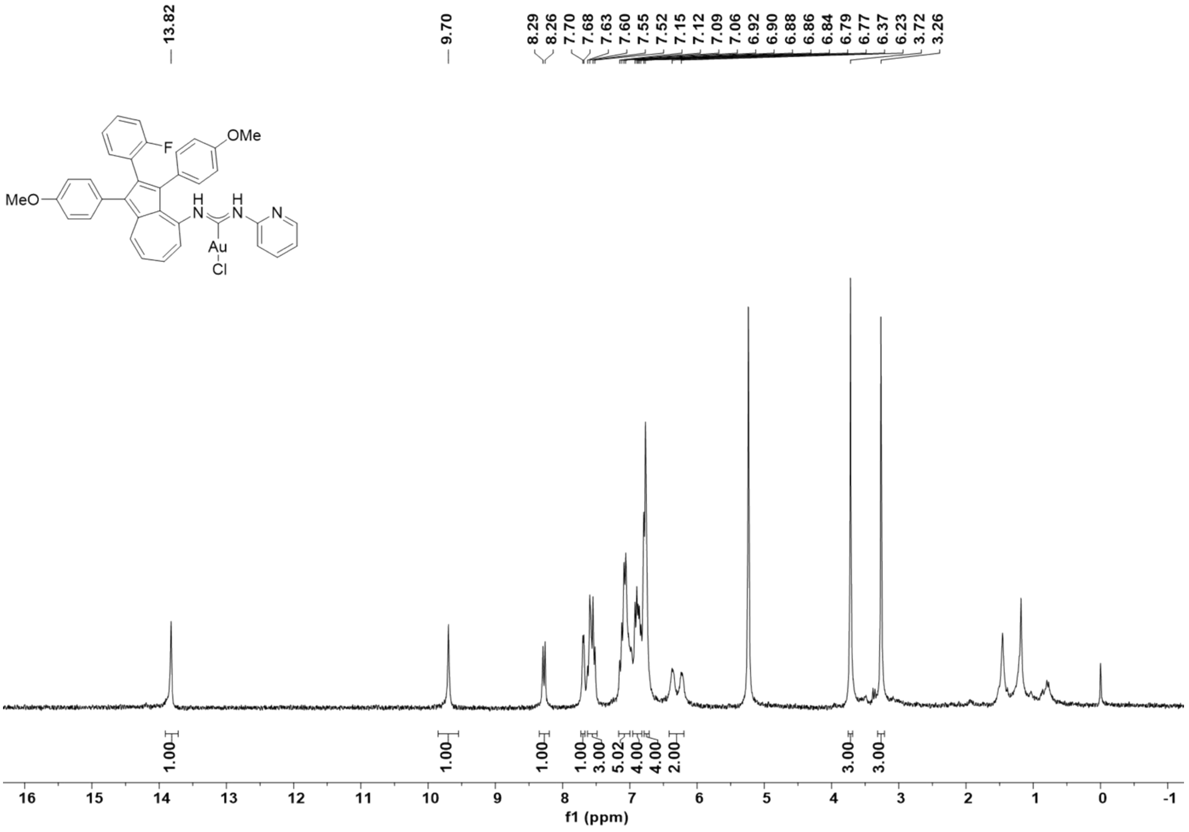

Figure S47. **^{1}H{^{19}F} NMR** Spectrum (301 MHz, CD_2Cl_2, 295 K) of **14ag**.

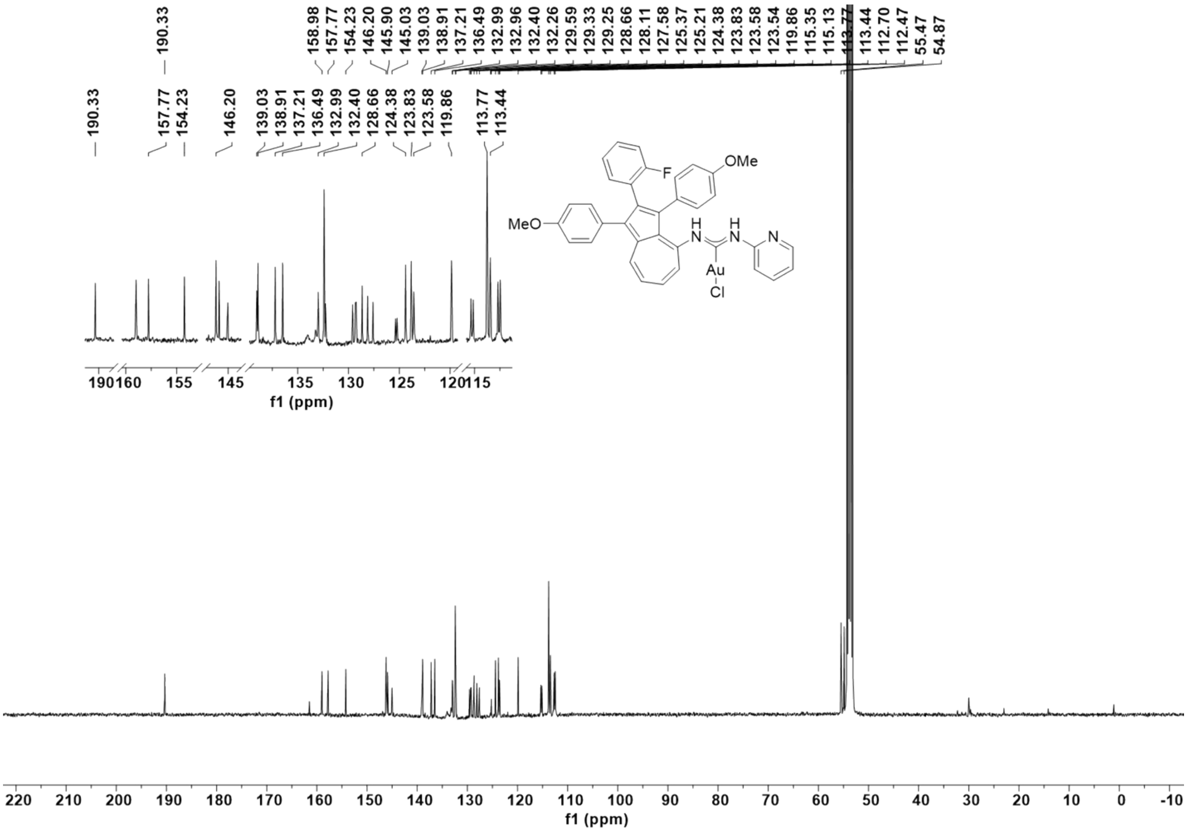

Figure S48. **^{13}C{^{19}F,^{1}H} NMR** Spectrum (101 MHz, CD_2Cl_2, 295 K) of **14ag**.

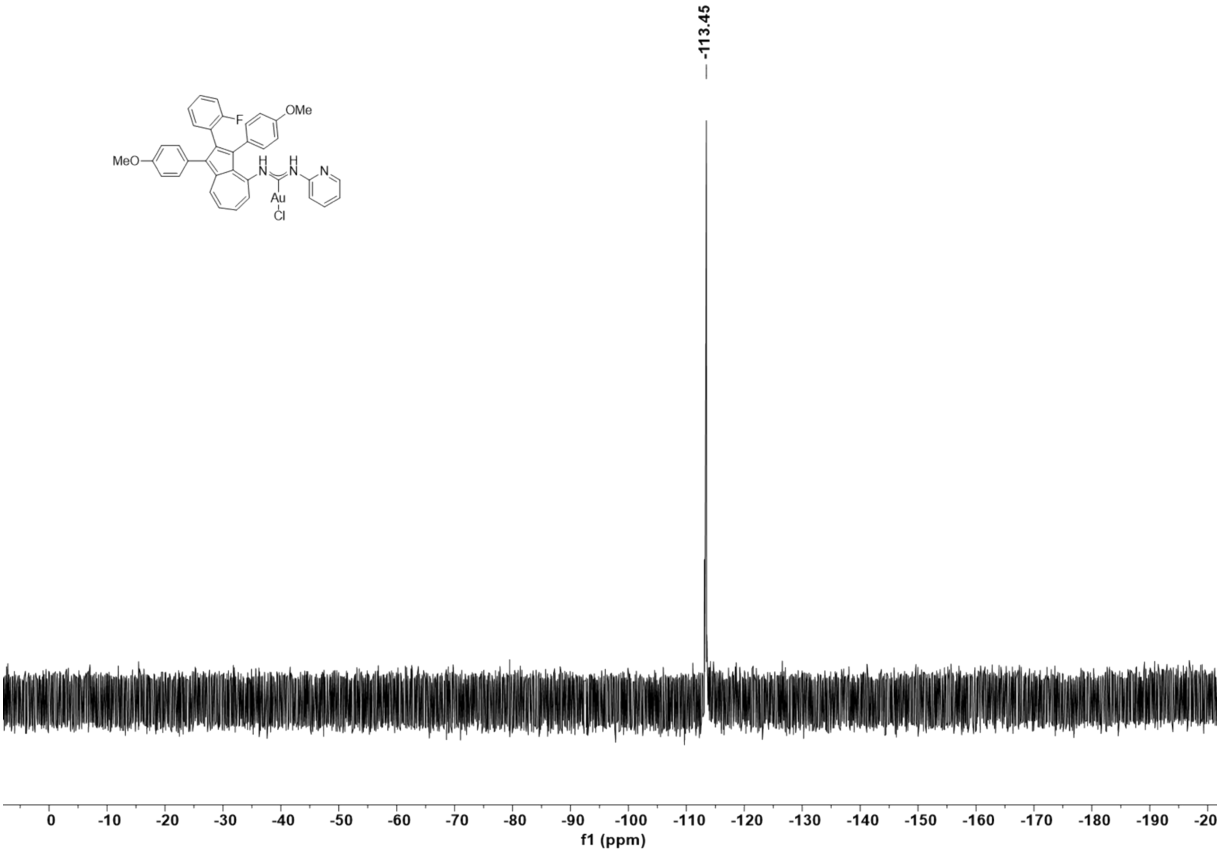

Figure S49. **$^{19}F\{^1H\}$ NMR** Spectrum (471 MHz, CD_2Cl_2, 295 K) of **14ag**.

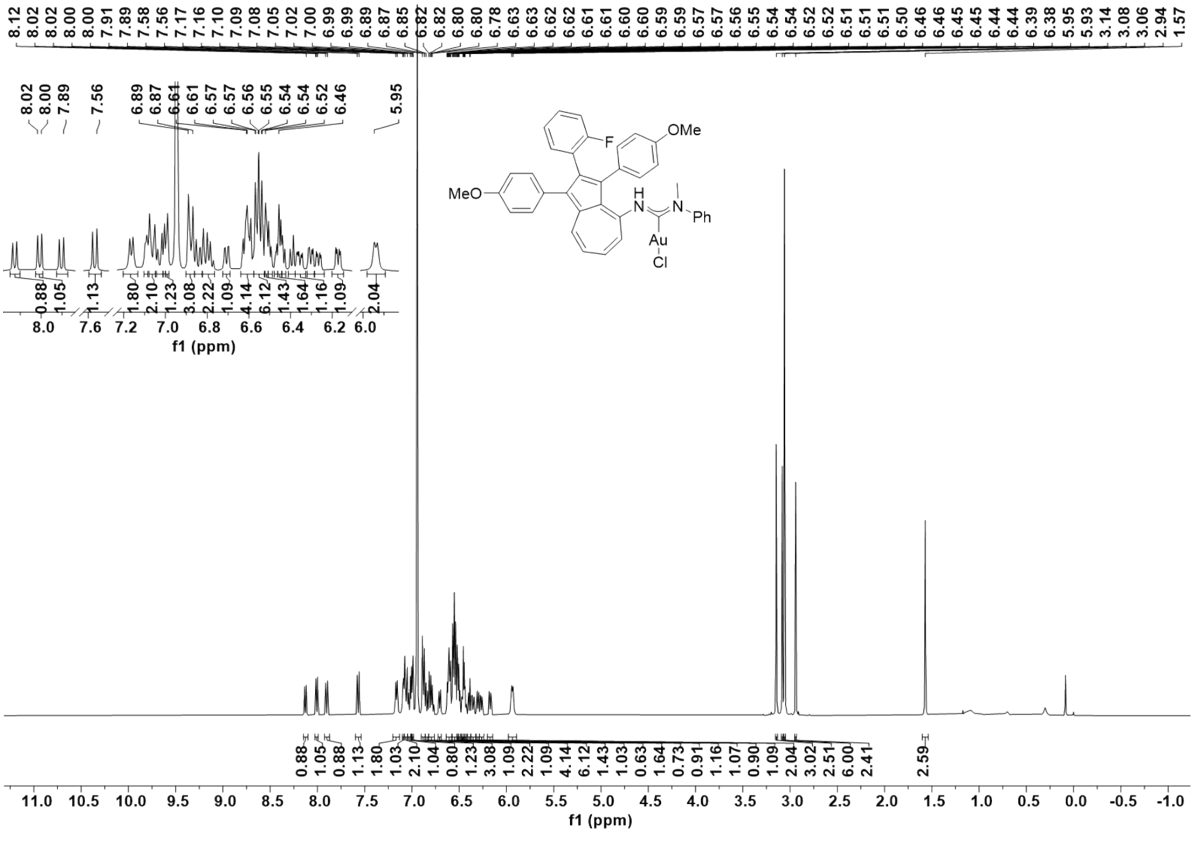

Figure S50. **$^1H\{^{19}F\}$ NMR** Spectrum (500 MHz, C_6D_6, 295 K) of **14ah**.

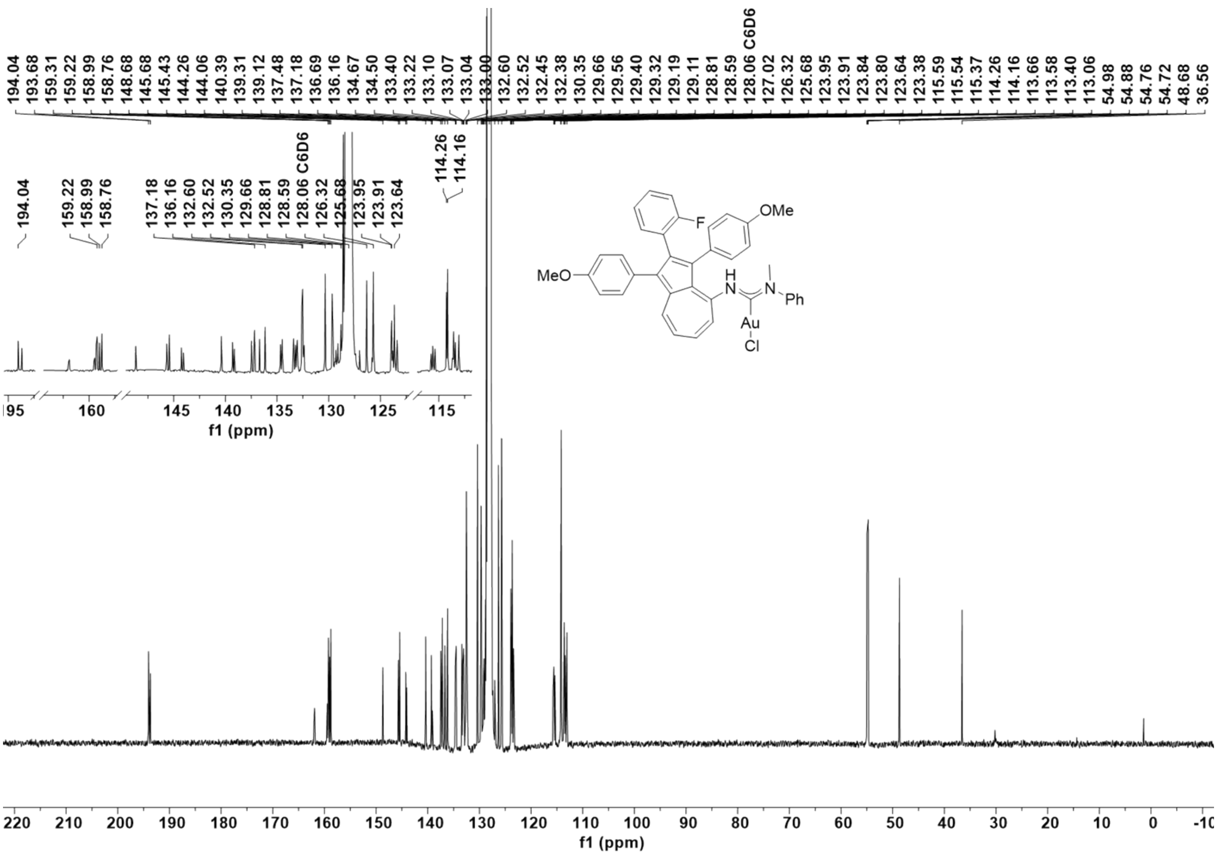

Figure S51.[13]**C{**[19]**F,**[1]**H} NMR** Spectrum (101 MHz, C_6D_6, 295 K) of **14ah**.

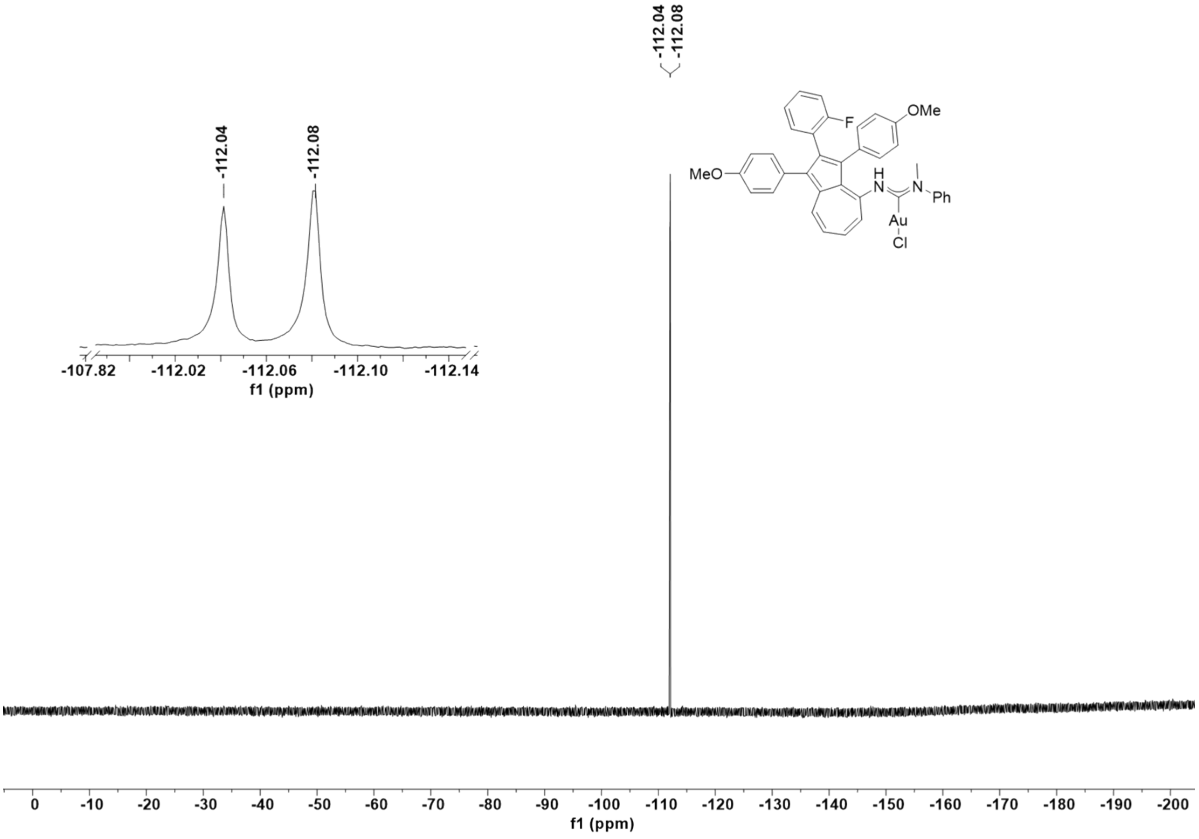

Figure S52. [19]**F{**[1]**H} NMR** Spectrum (283 MHz, C_6D_6, 295 K) of **14ah**.

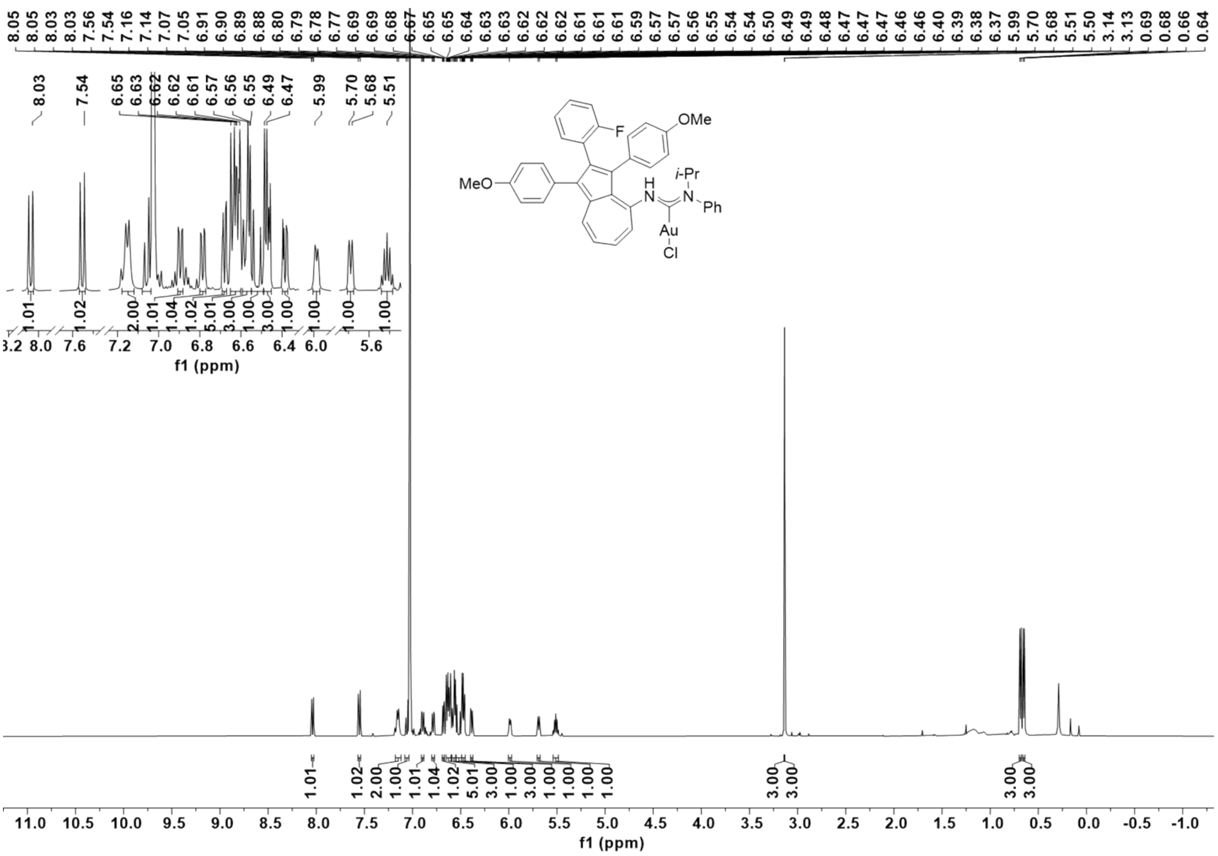

Figure S53. $^{1}H\{^{19}F\}$ NMR Spectrum (500 MHz, C_6D_6, 295 K) of **14ai**.

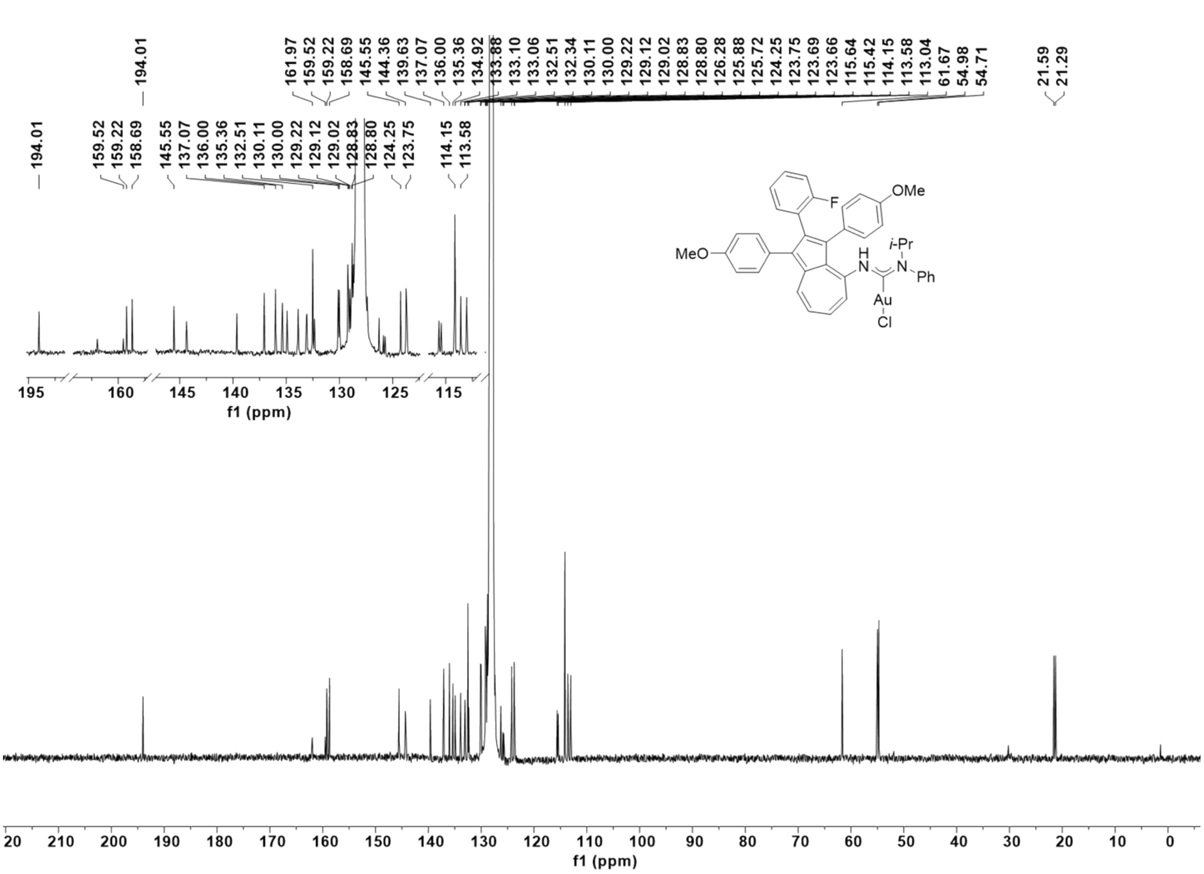

Figure S54. $^{13}C\{^{19}F,^{1}H\}$ NMR Spectrum (101 MHz, C_6D_6, 295 K) of **14ai**.

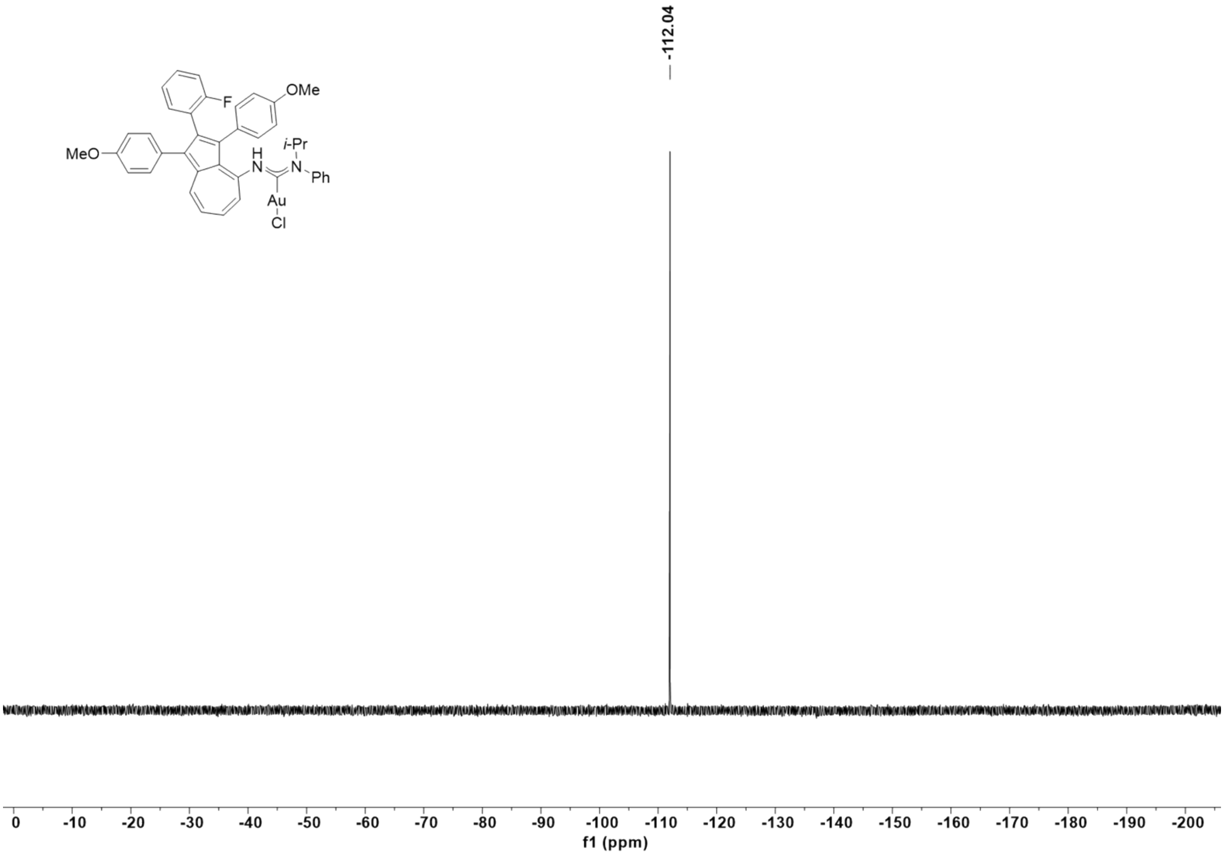

Figure S55. ^{19}**F{**^{1}**H} NMR** Spectrum (471 MHz, C_6D_6, 295 K) of **14ai**.

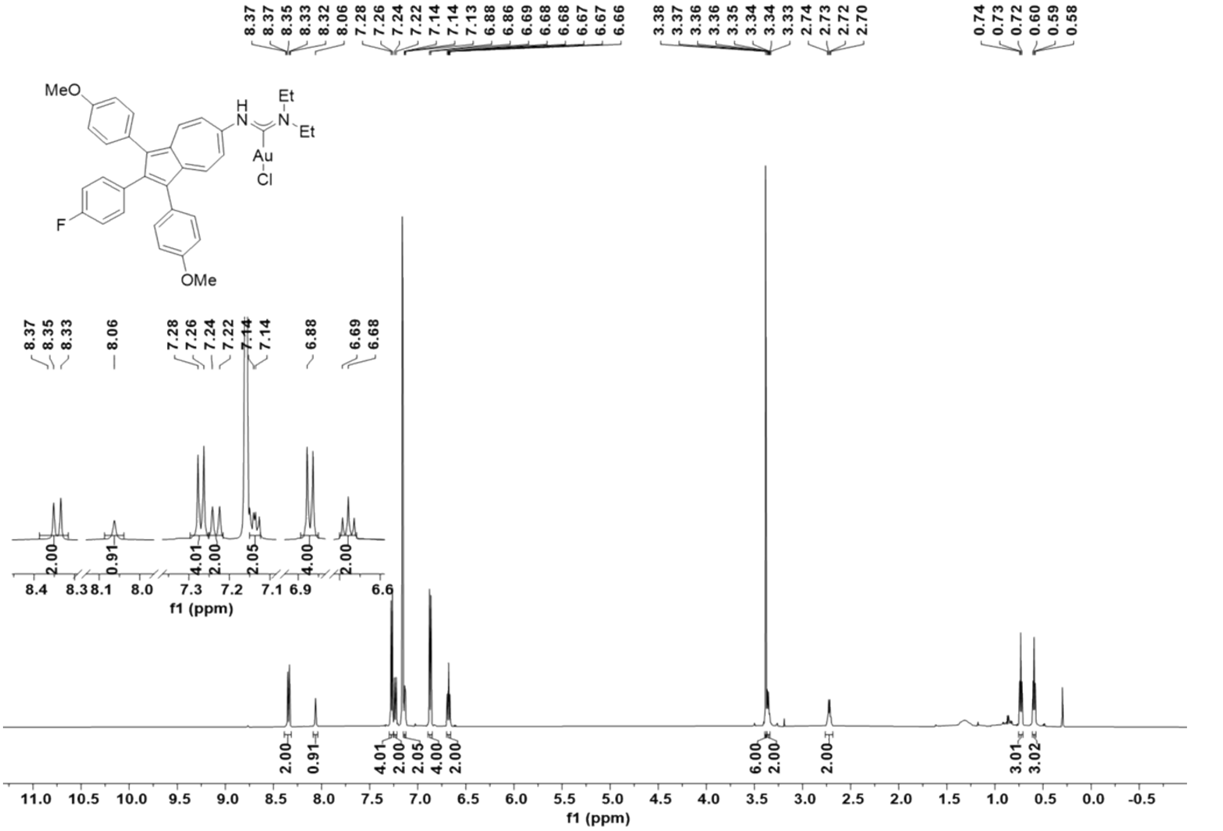

Figure S56. ^{1}**H{**^{19}**F} NMR** Spectrum (600 MHz, C_6D_6, 295 K) of **14ba**.

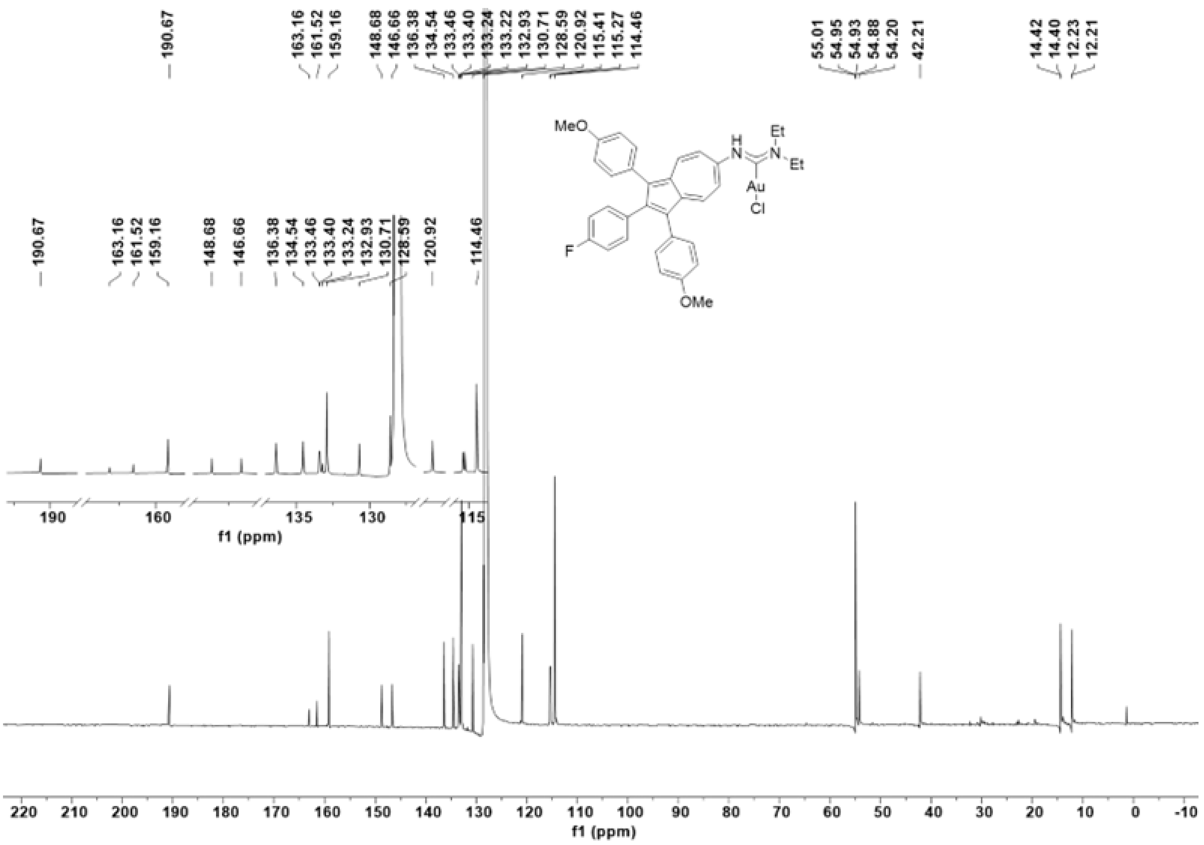

Figure S57. $^{13}C\{^{19}F,^{1}H\}$ **NMR** Spectrum (151 MHz, C_6D_6, 295 K) of **14ba**.

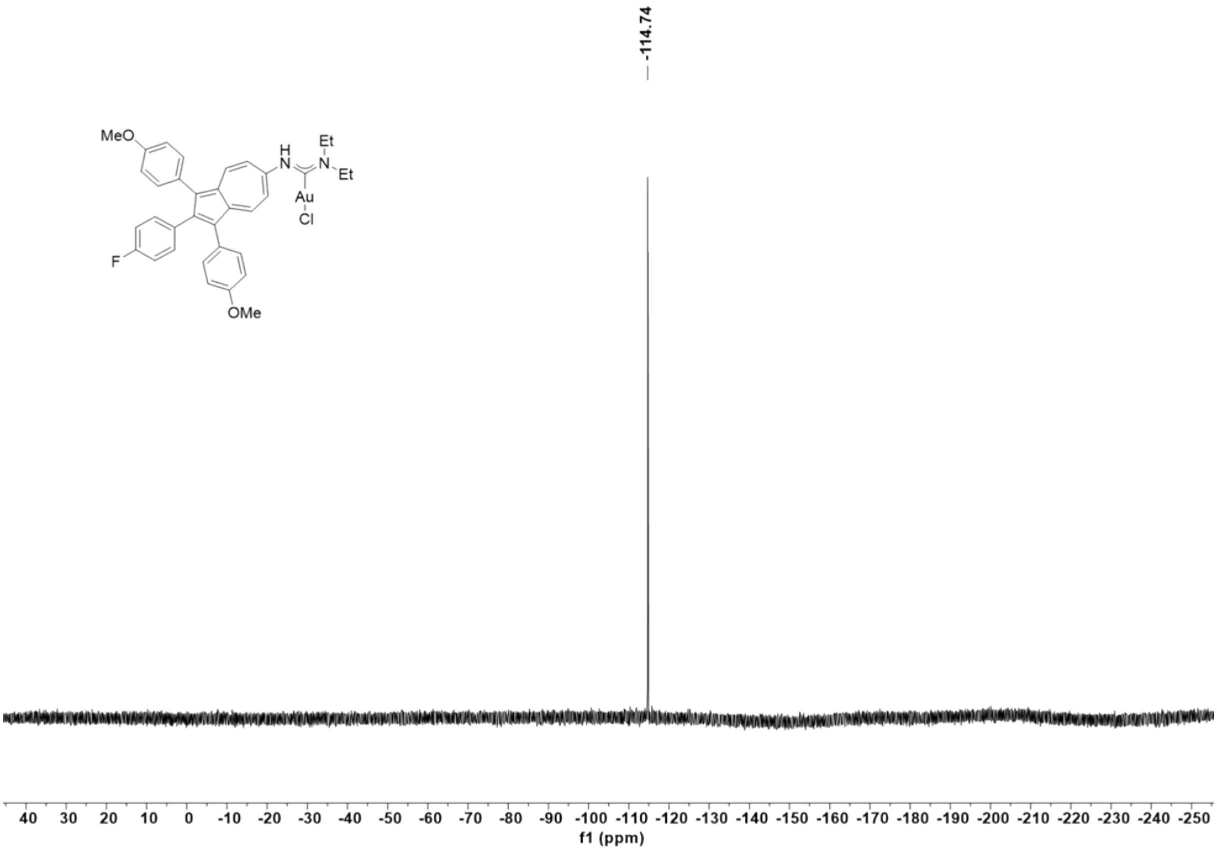

Figure S58. $^{19}F\{^{1}H\}$ **NMR** Spectrum (283 MHz, C_6D_6, 295 K) of **14ba**.

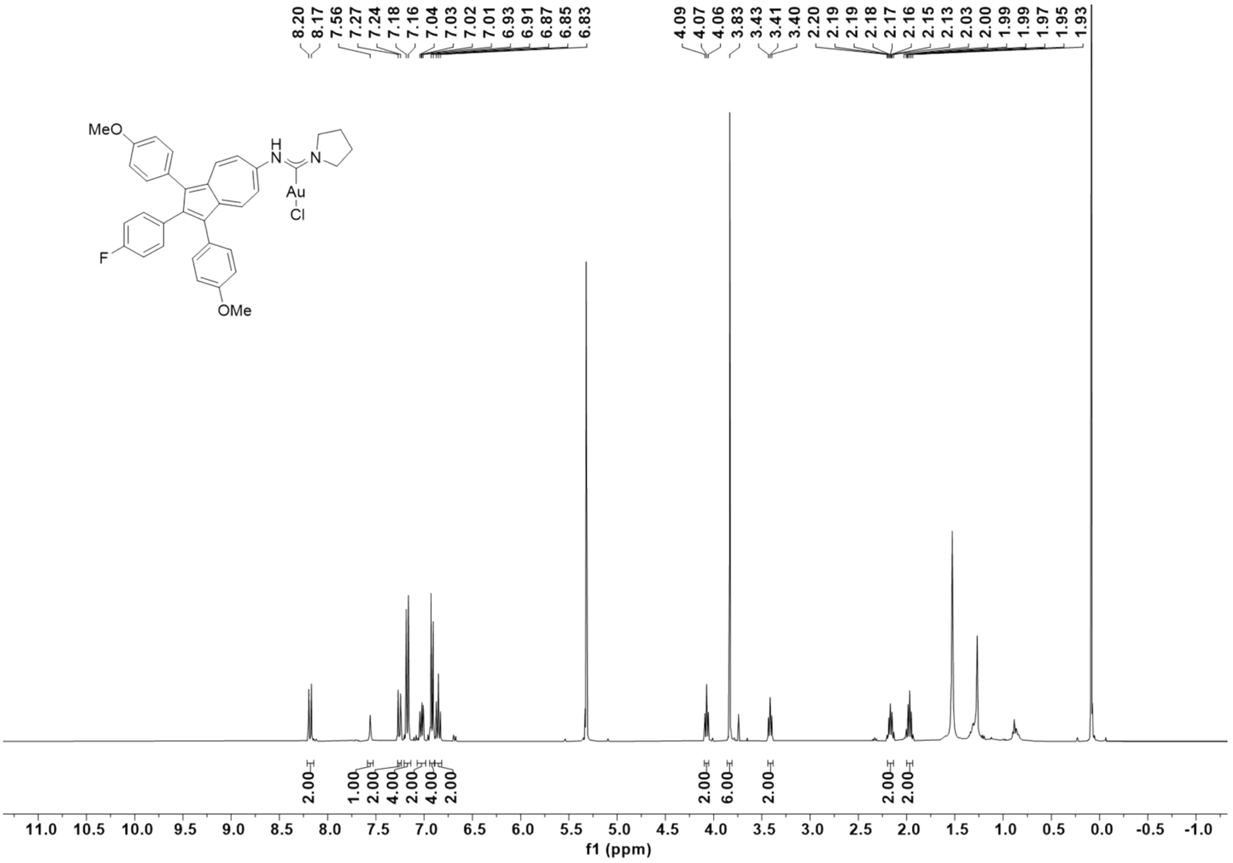

Figure S59. ^{1}**H{**^{19}**F} NMR** Spectrum (400 MHz, CD_2Cl_2, 295 K) of **14bb**.

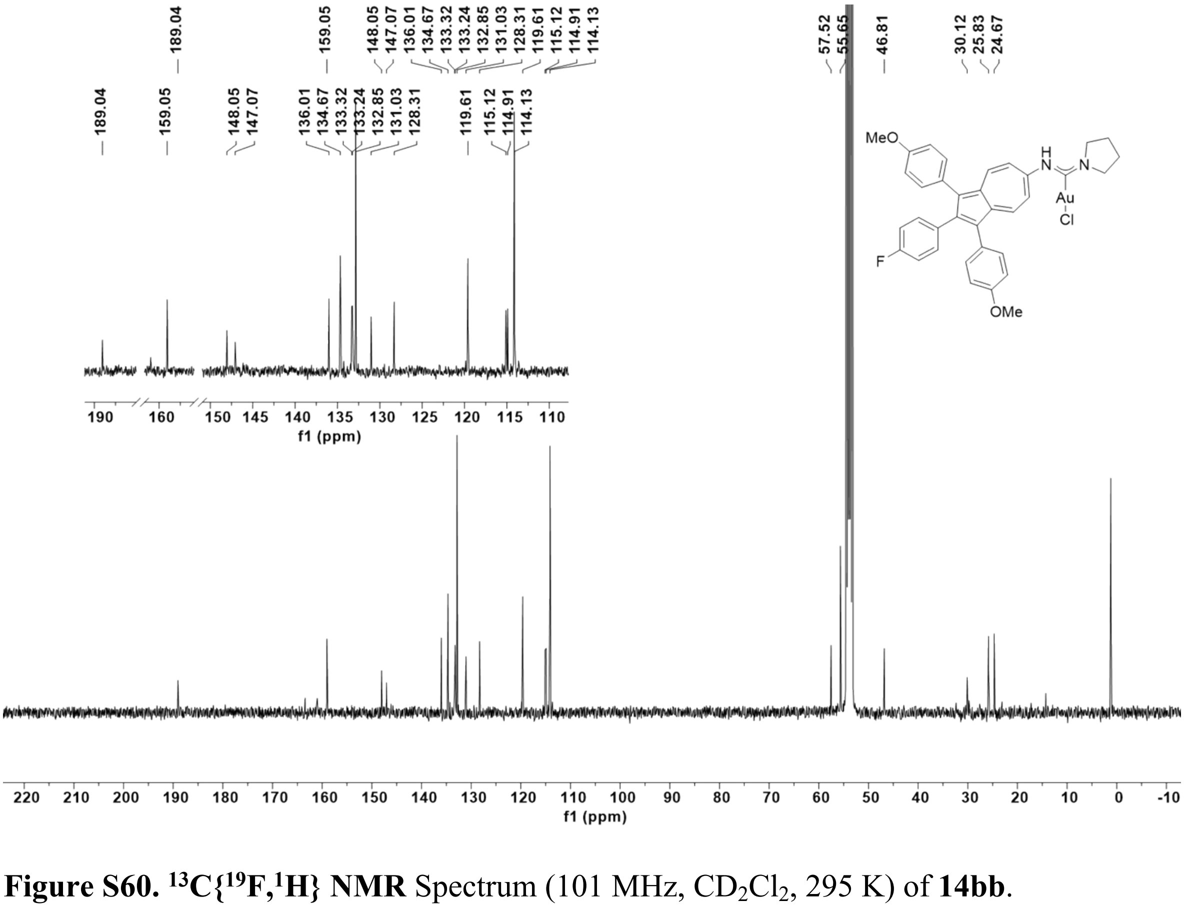

Figure S60. ^{13}**C{**^{19}**F,**^{1}**H} NMR** Spectrum (101 MHz, CD_2Cl_2, 295 K) of **14bb**.

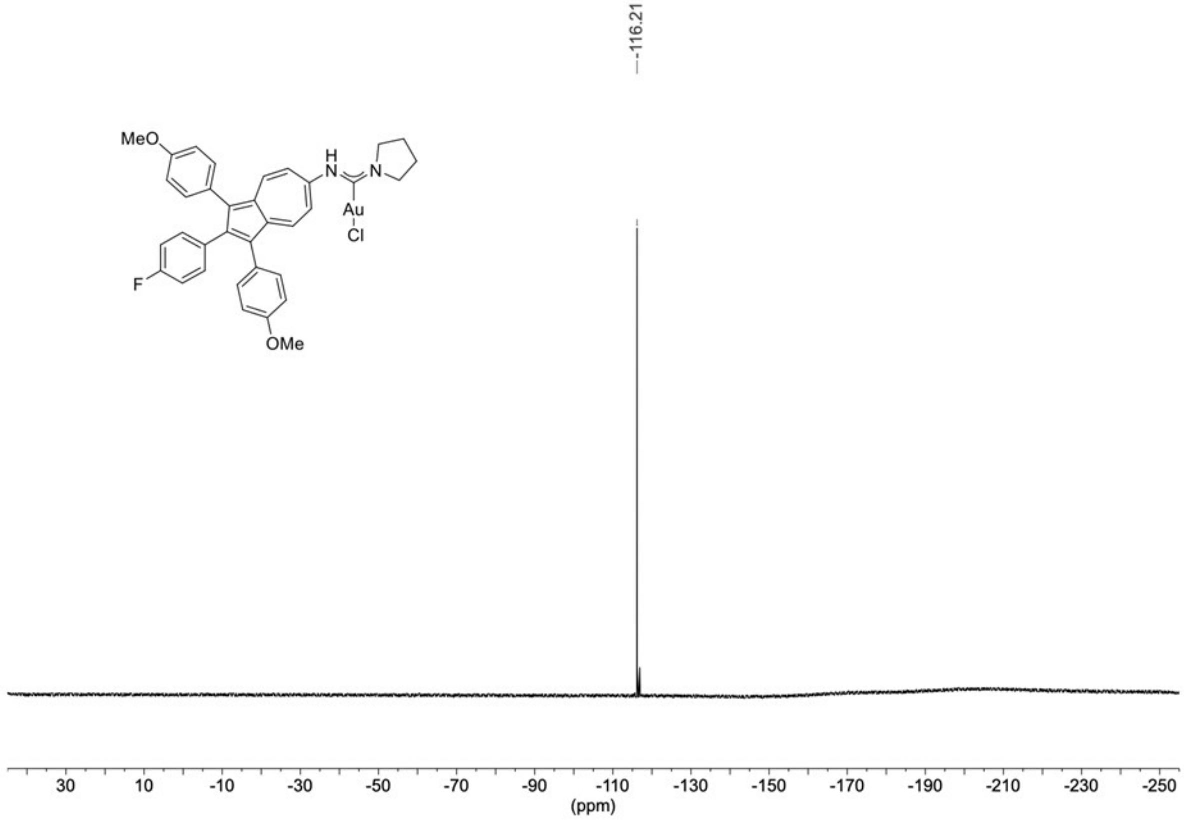

Figure S61. $^{19}F\{^{1}H\}$ NMR Spectrum (283 MHz, CD_2Cl_2, 298 K) of **14bb**.

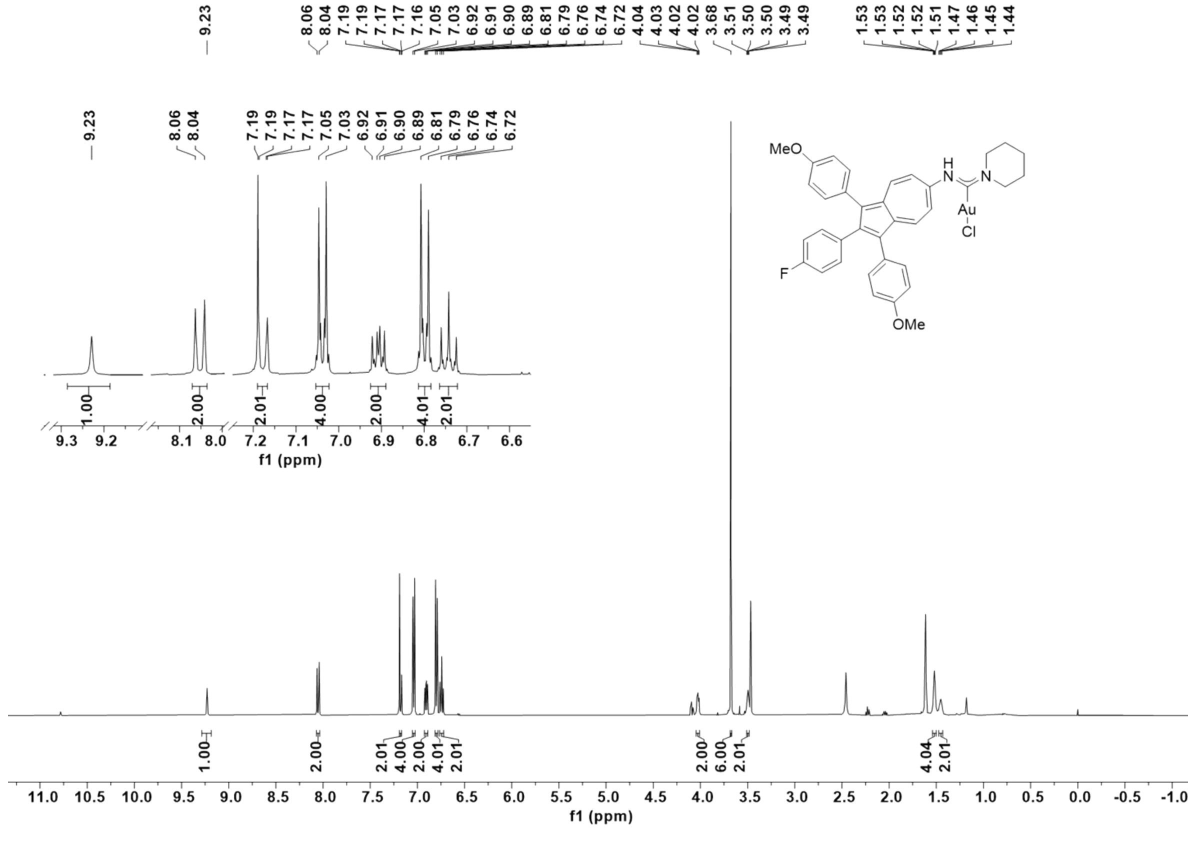

Figure S62. $^{1}H\{^{19}F\}$ NMR Spectrum (500 MHz, THF-d_8, 295 K) of **14bc.**

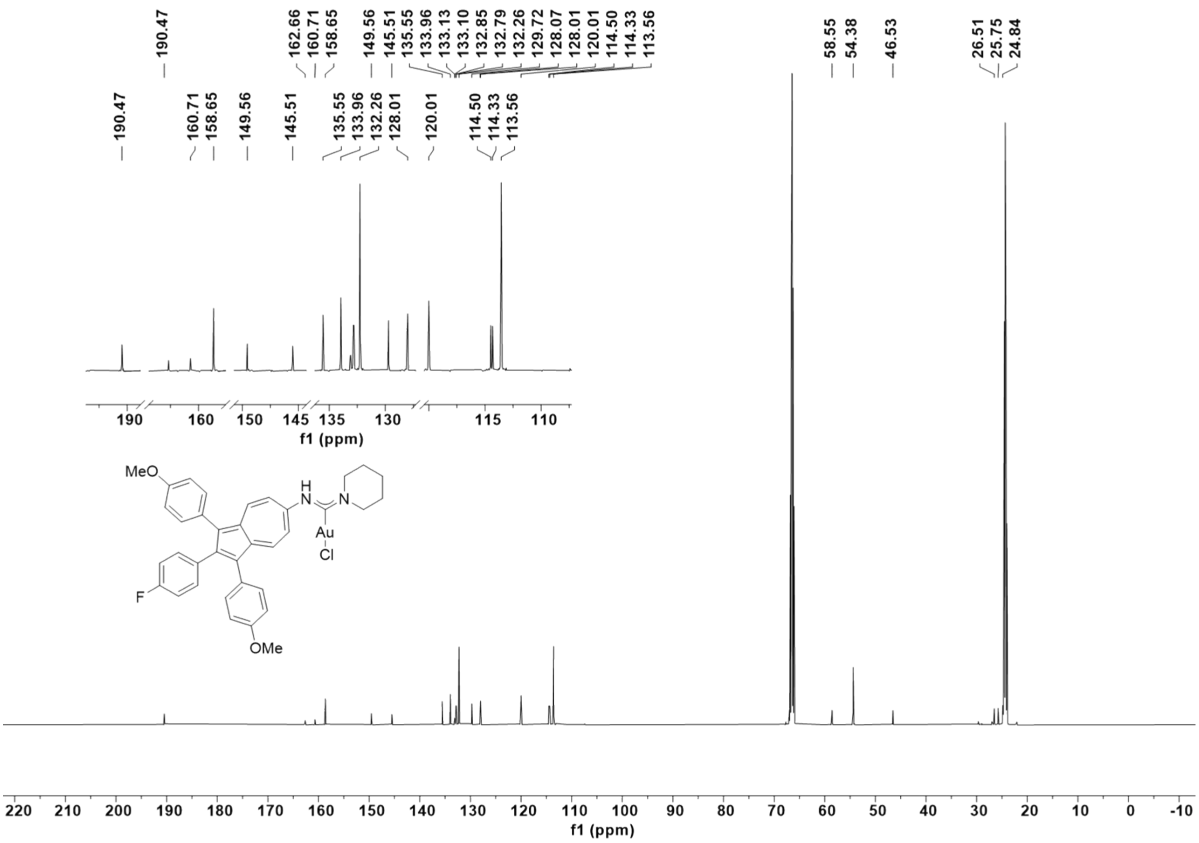

Figure S63. ^{13}**C{**^{19}**F,**^{1}**H} NMR** Spectrum (126 MHz, THF-d_8, 295 K) of **14bc.**

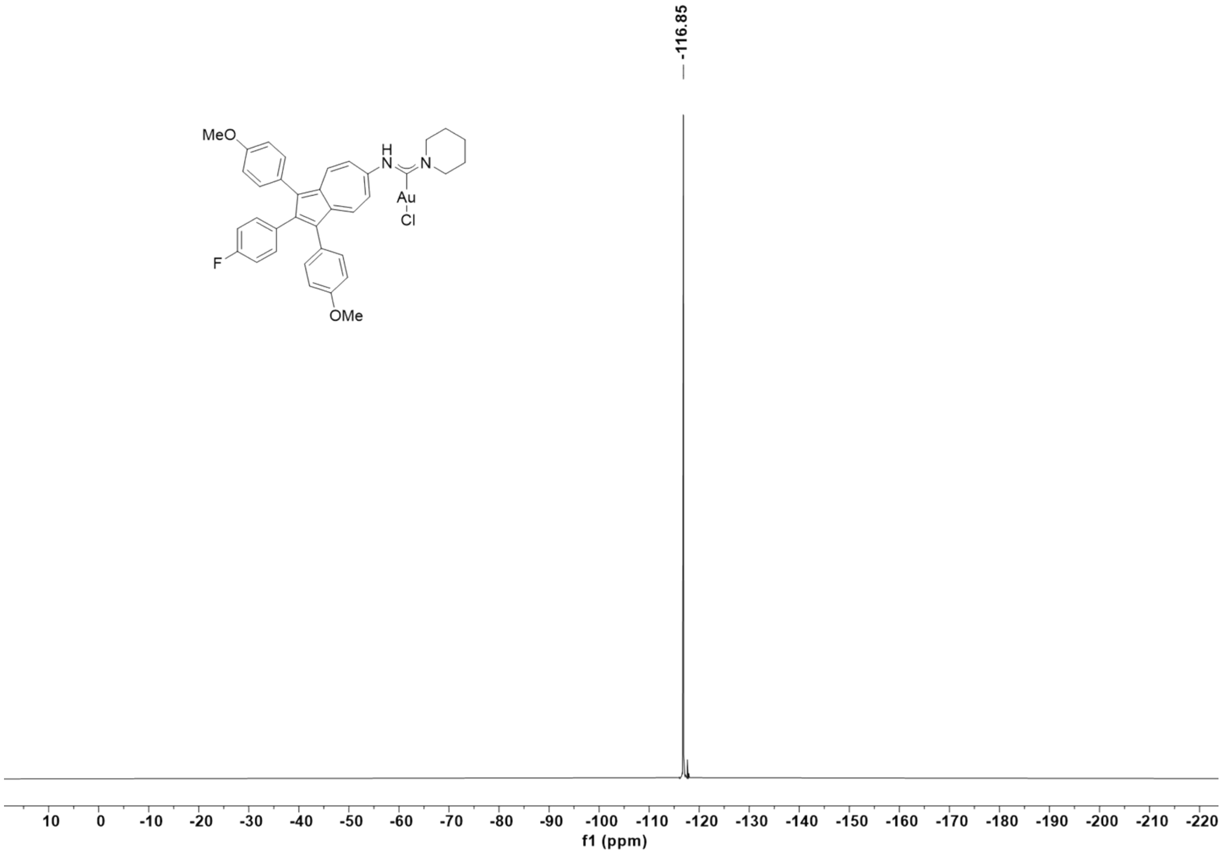

Figure S64.^{19}**F{**^{1}**H} NMR** Spectrum (471 MHz, THF-d_8, 295 K) of **14bc.**

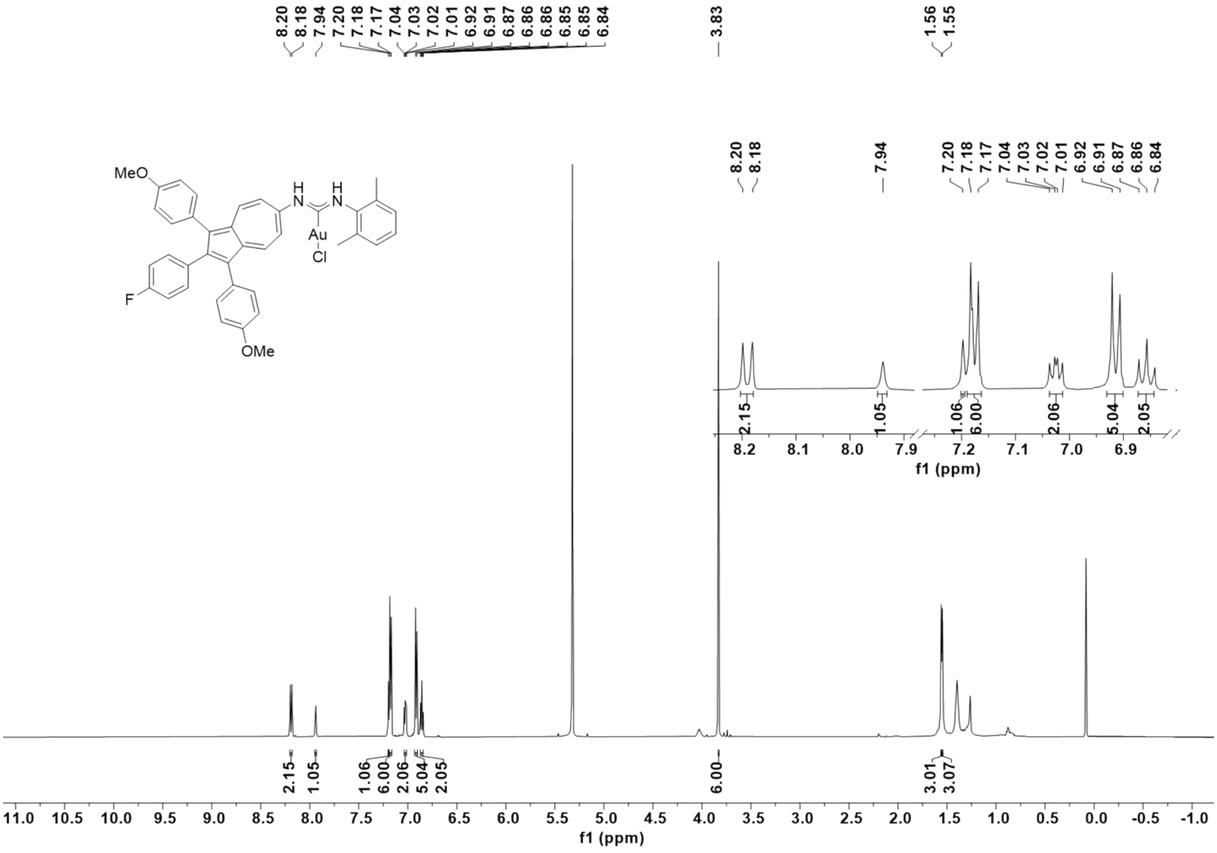

Figure S65. **^{1}H{^{19}F} NMR** Spectrum (600 MHz, CD_2Cl_2, 295 K) of **14bd**.

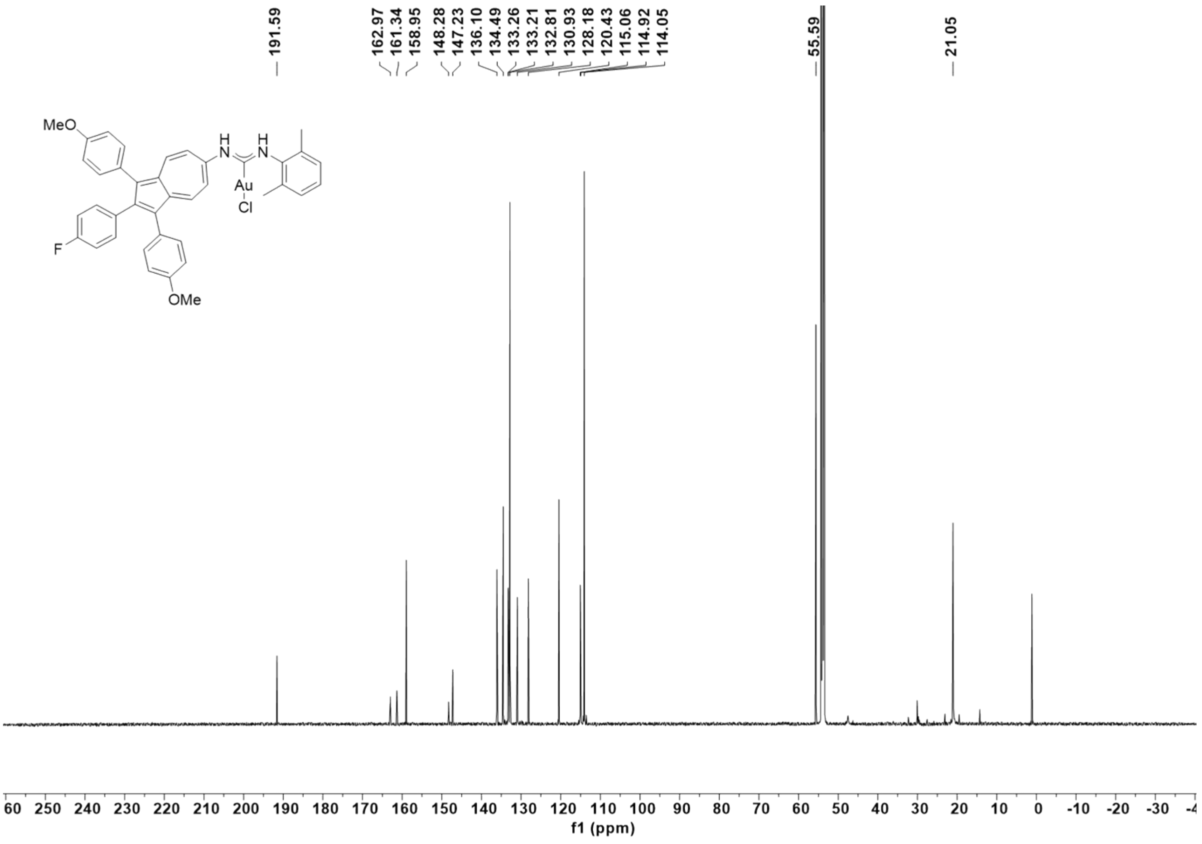

Figure S66. **^{13}C{^{19}F,^{1}H} NMR** Spectrum (151 MHz, CD_2Cl_2, 295 K) of **14bd**.

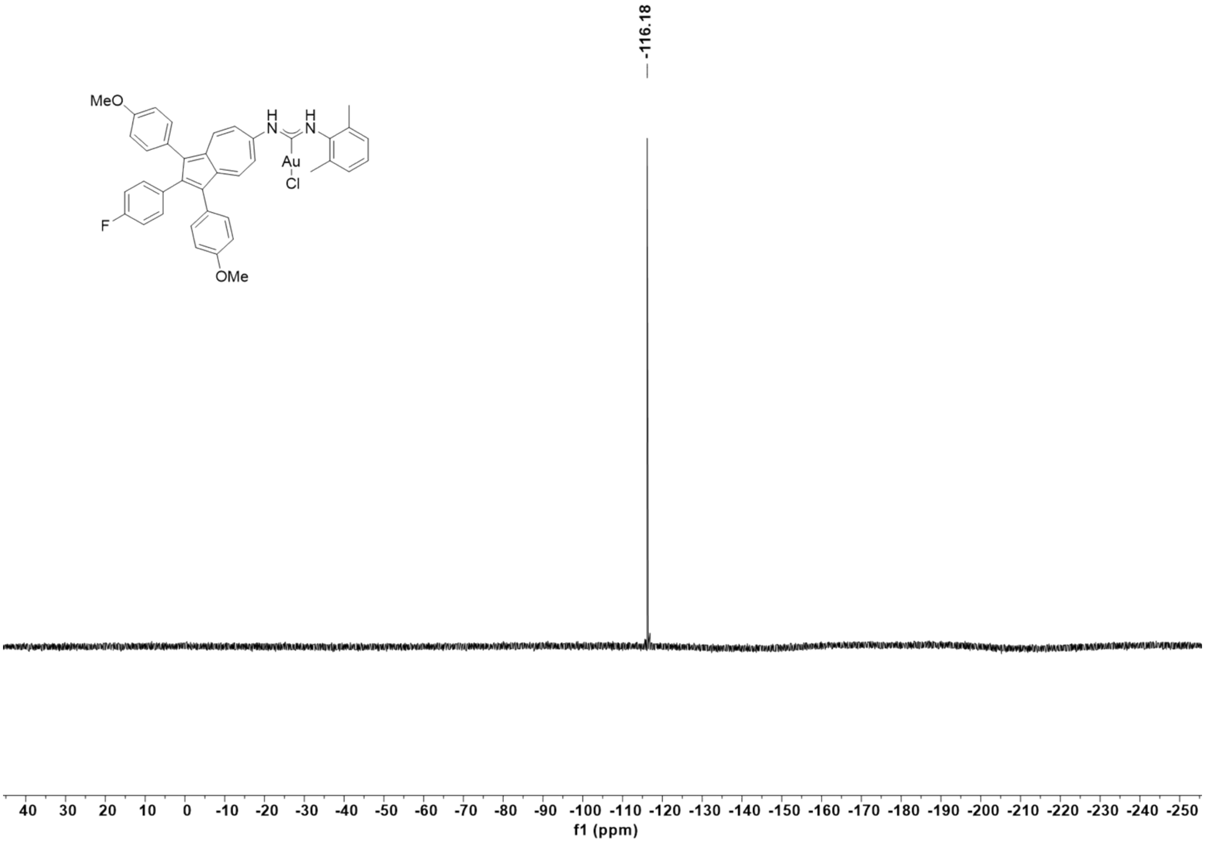

Figure S67. ^{19}F{^{1}H} **NMR** Spectrum (283 MHz, CD_2Cl_2, 295 K) of **14bd**.

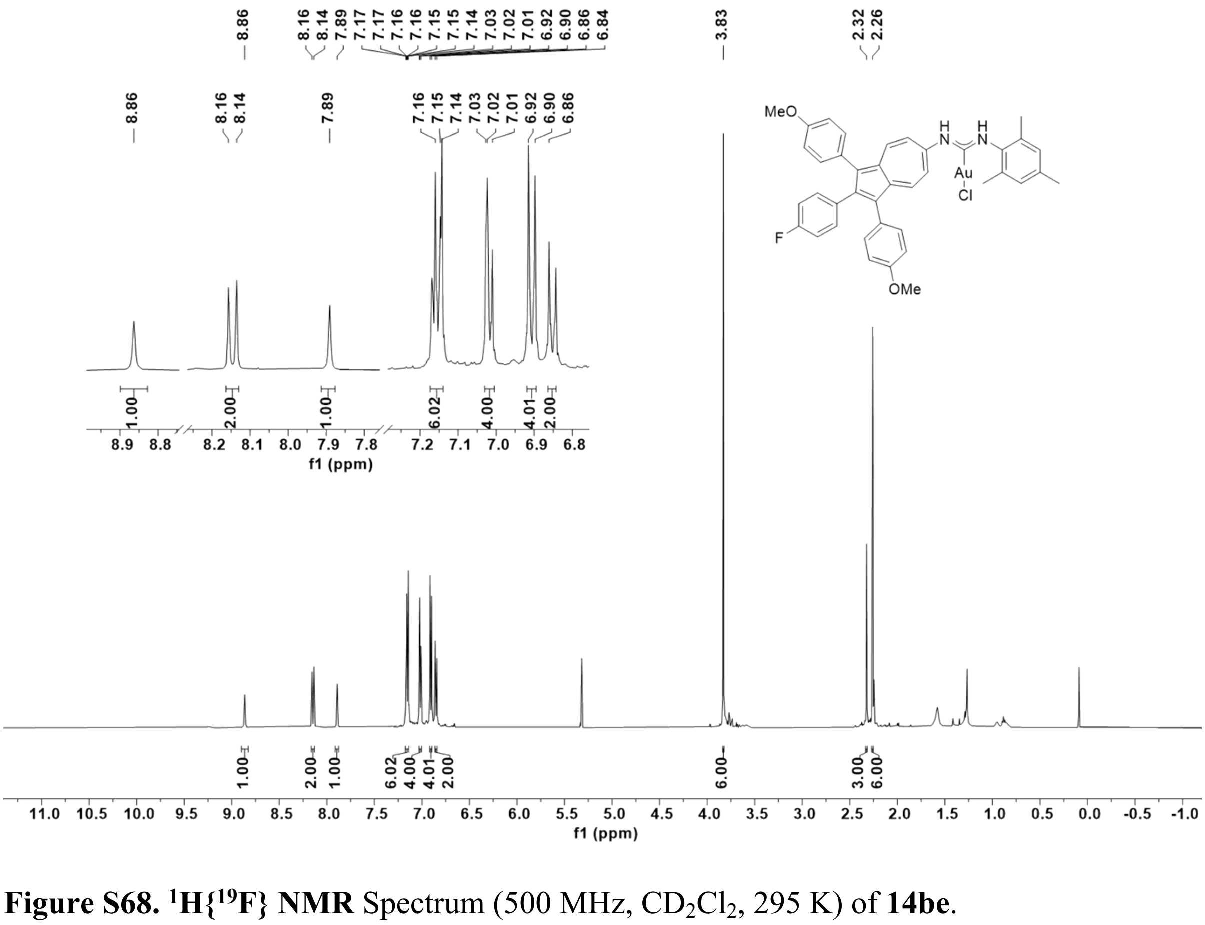

Figure S68. ^{1}H{^{19}F} **NMR** Spectrum (500 MHz, CD_2Cl_2, 295 K) of **14be**.

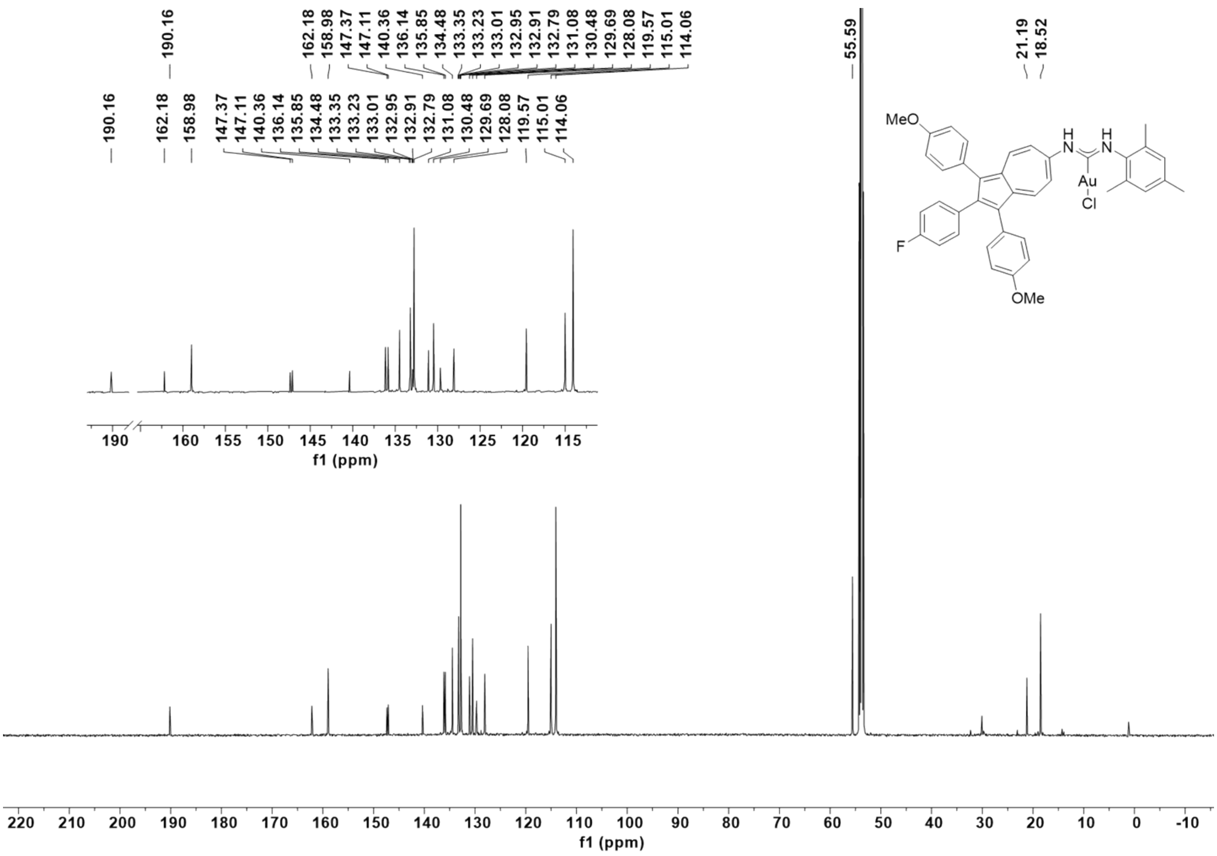

Figure S69. $^{13}C\{^{19}F,^{1}H\}$ **NMR** Spectrum (126 MHz, CD_2Cl_2, 295 K) of **14be**.

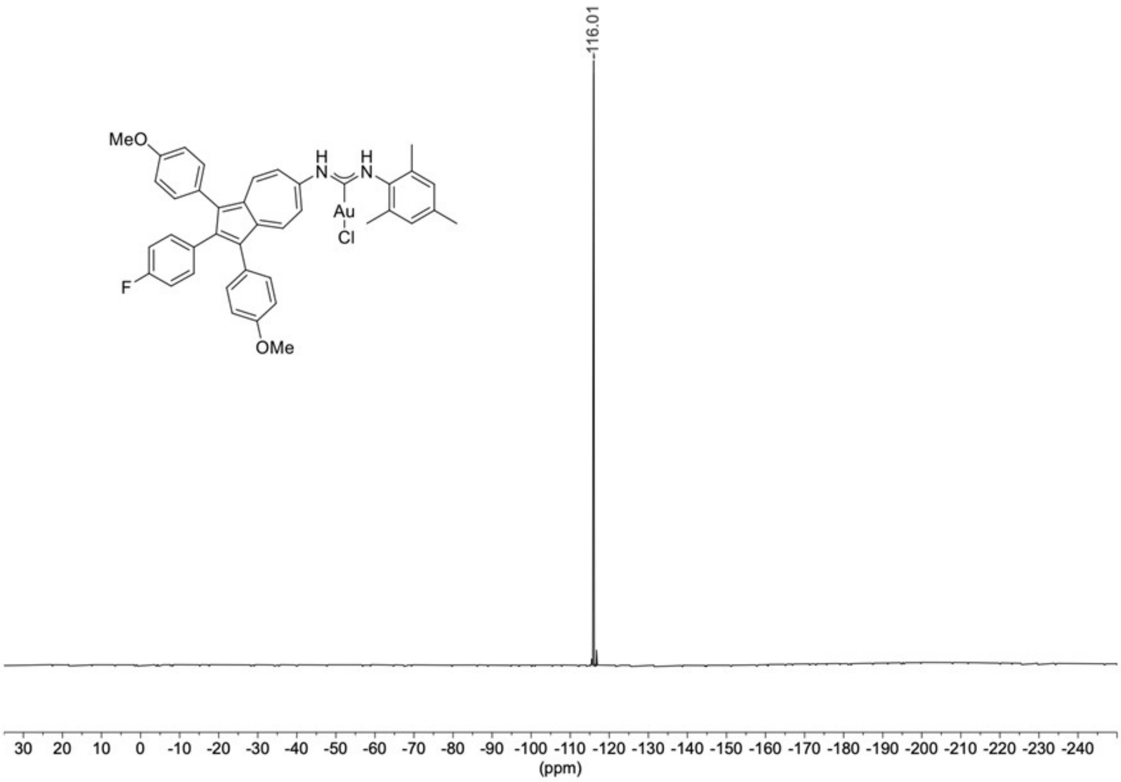

Figure S70. $^{19}F\{^{1}H\}$ **NMR** Spectrum (283 MHz, CD_2Cl_2, 298 K) of **14be**.

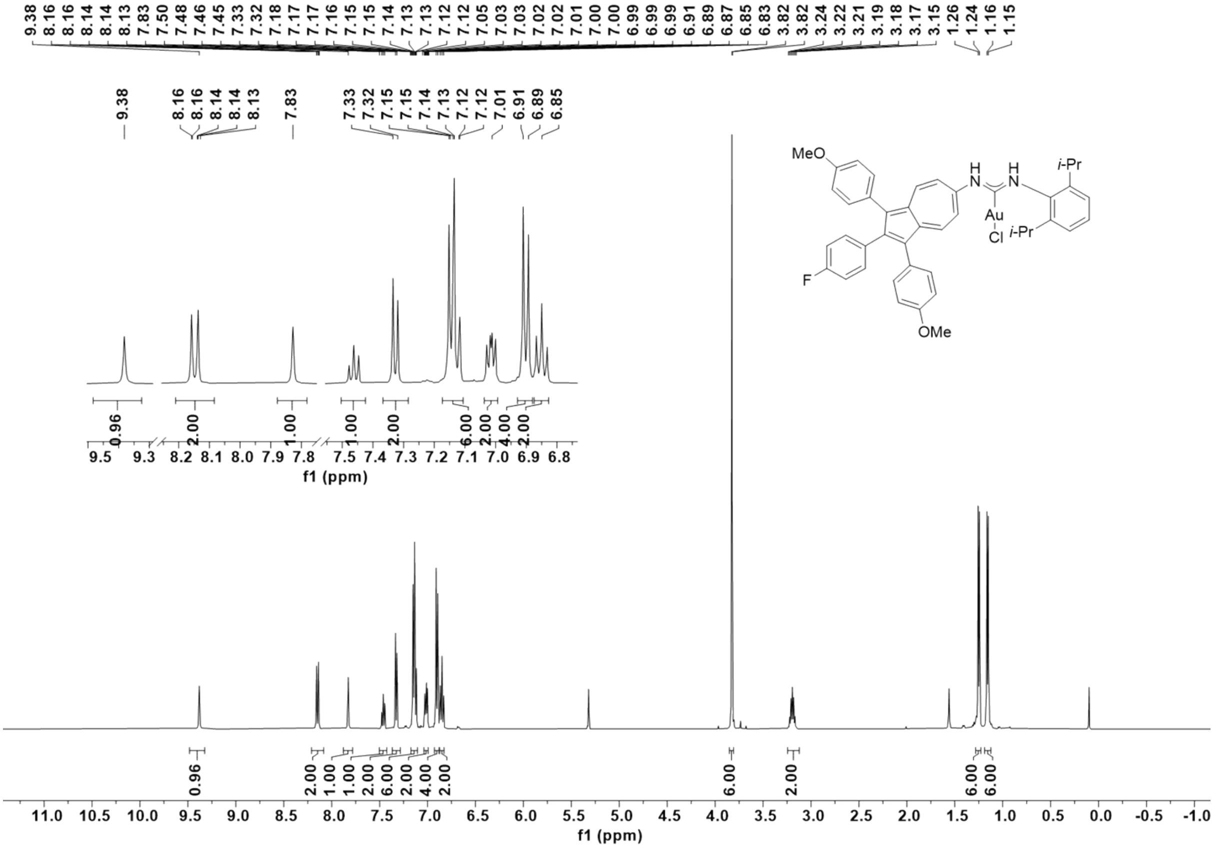

Figure S71. ^{1}**H{**^{19}**F} NMR** Spectrum (500 MHz, CD_2Cl_2, 295 K) of **14bf**.

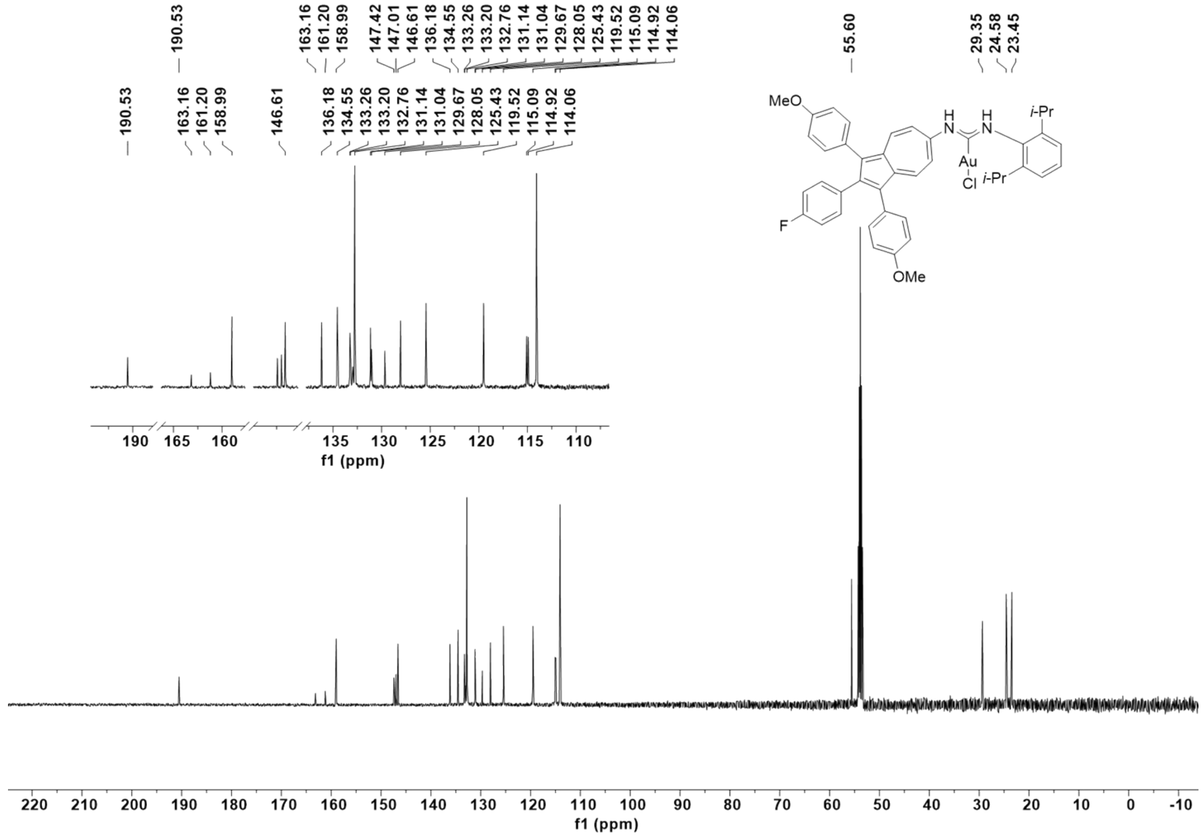

Figure S72. ^{13}**C{**^{19}**F,**^{1}**H} NMR** Spectrum (126 MHz, CD_2Cl_2, 295 K) of **14bf**.

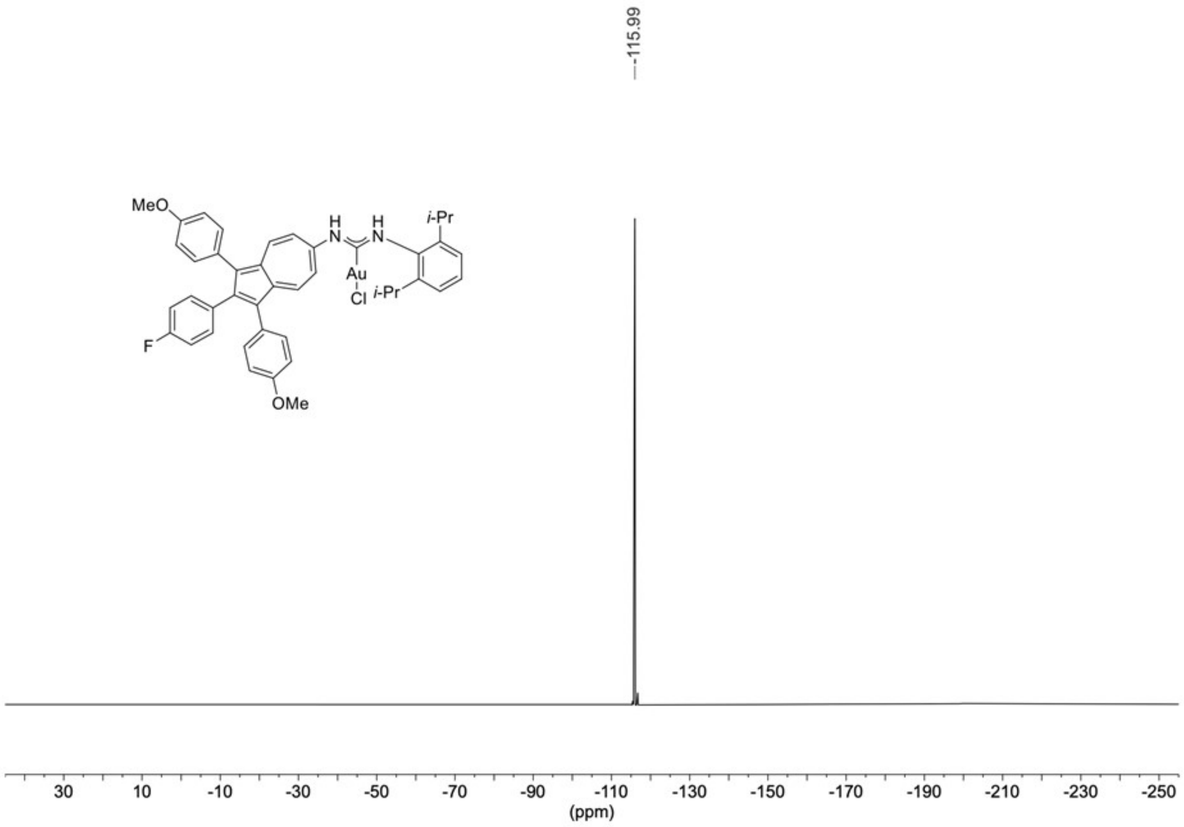

Figure S73. ^{19}**F{**^{1}**H} NMR** Spectrum (283 MHz, CD_2Cl_2, 298 K) of **14bf**.

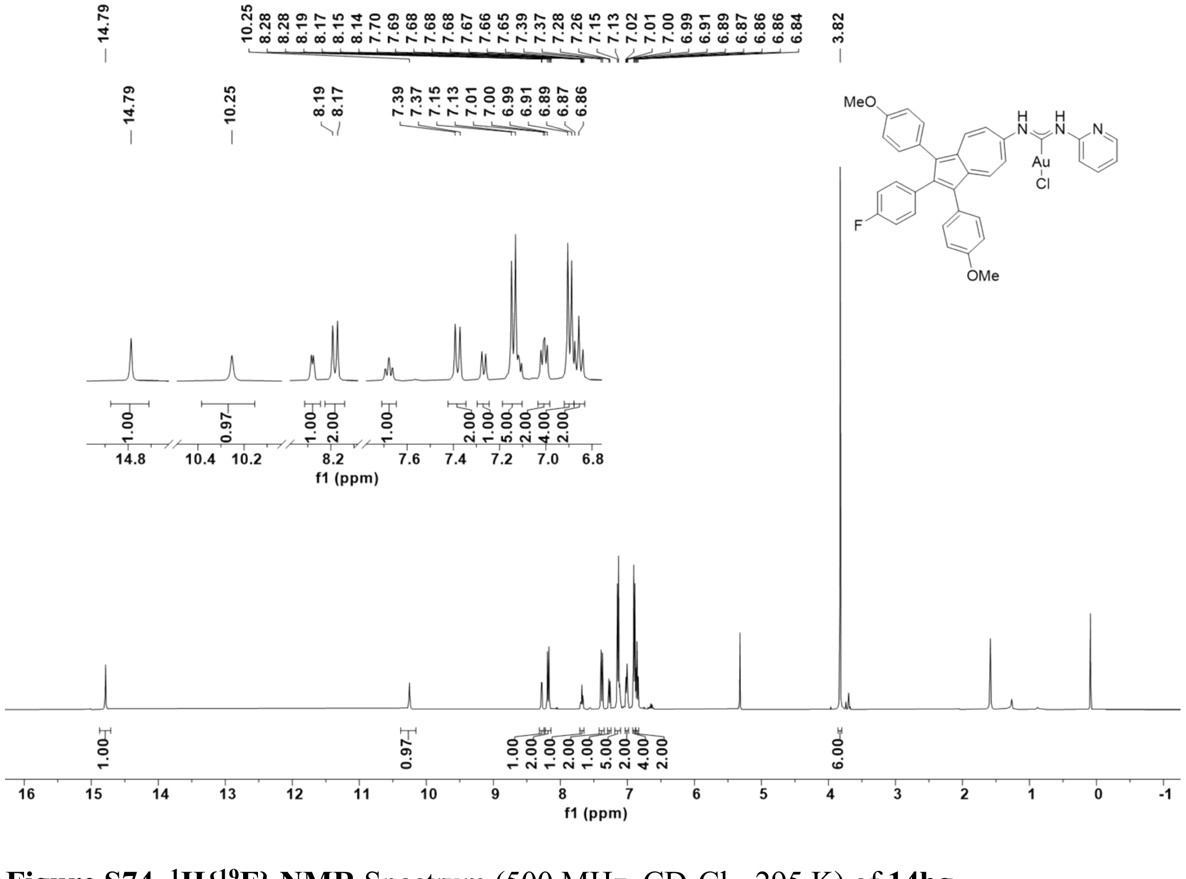

Figure S74. ^{1}**H{**^{19}**F} NMR** Spectrum (500 MHz, CD_2Cl_2, 295 K) of **14bg**.

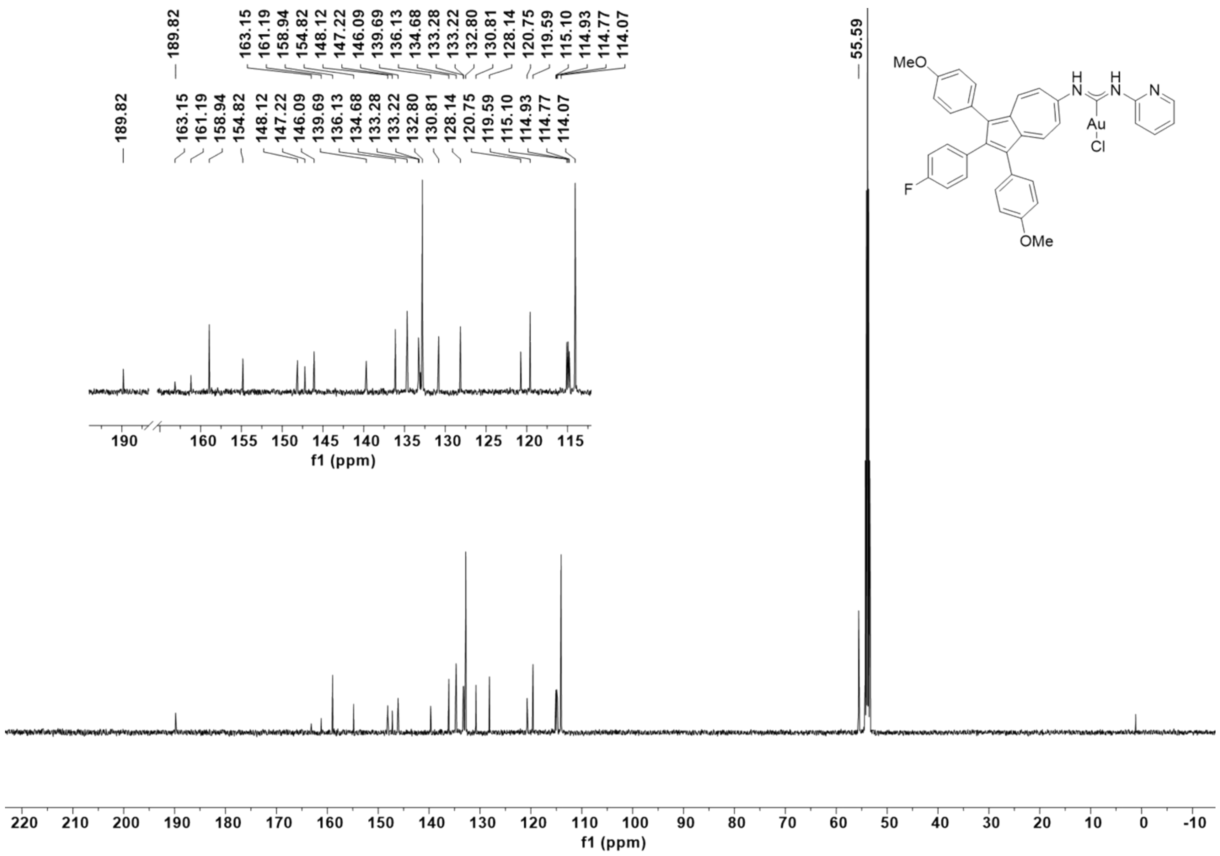

Figure S75. $^{13}C\{^{19}F,^{1}H\}$ NMR Spectrum (126 MHz, CD_2Cl_2, 295 K) of **14bg**.

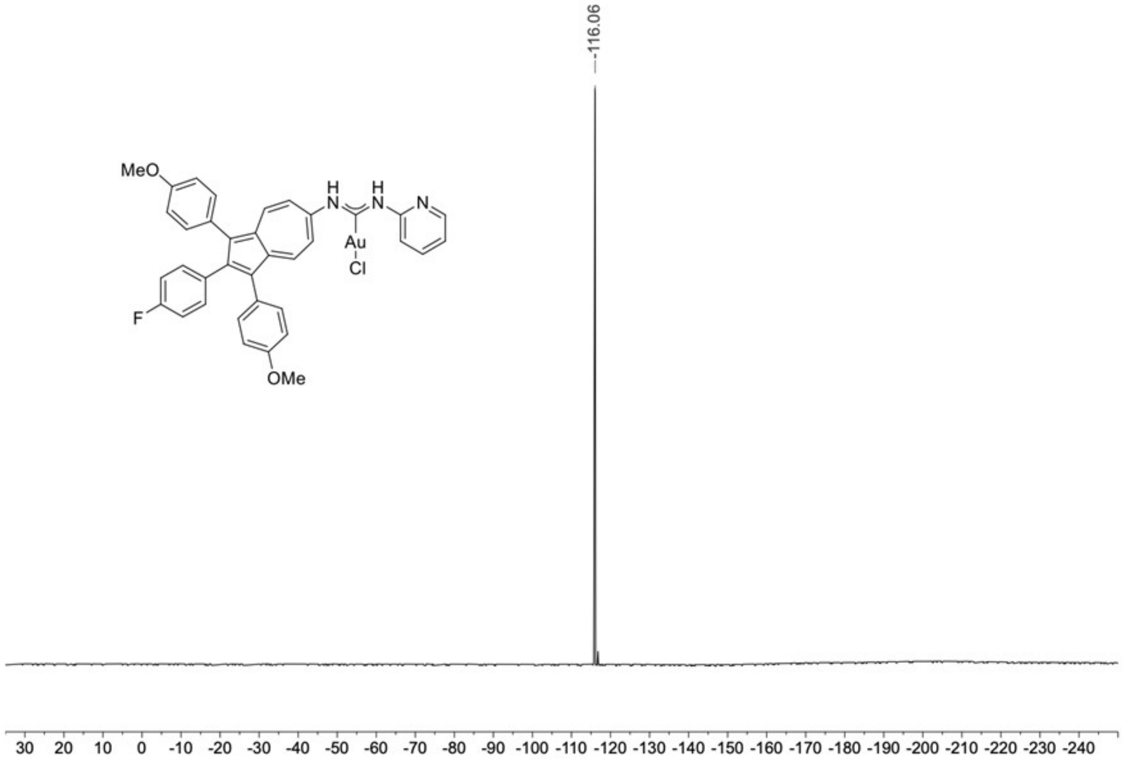

Figure S76. $^{19}F\{^{1}H\}$ NMR Spectrum (283 MHz, CD_2Cl_2, 295 K) of **14bg**.

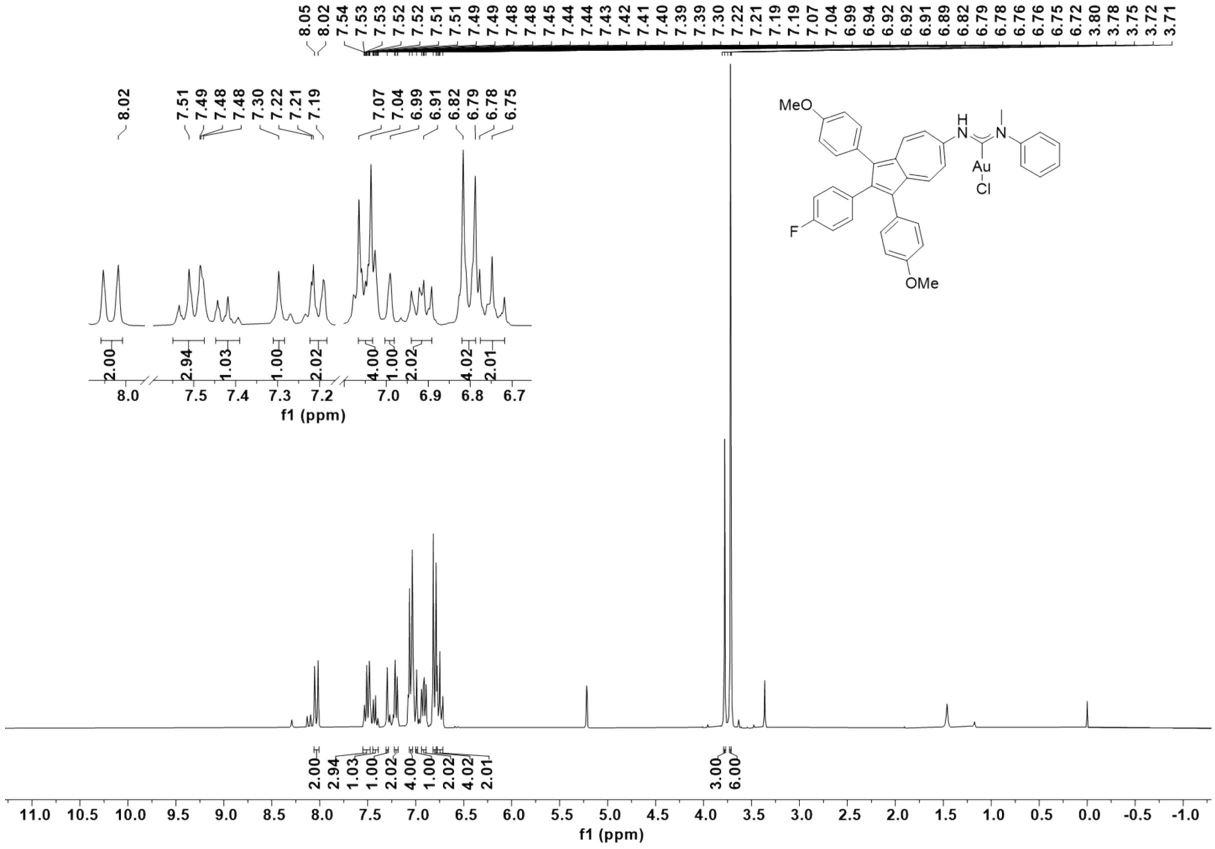

Figure S77. **$^{1}H\{^{19}F\}$ NMR** Spectrum (300 MHz, CD_2Cl_2, 295 K) of **14bh**.

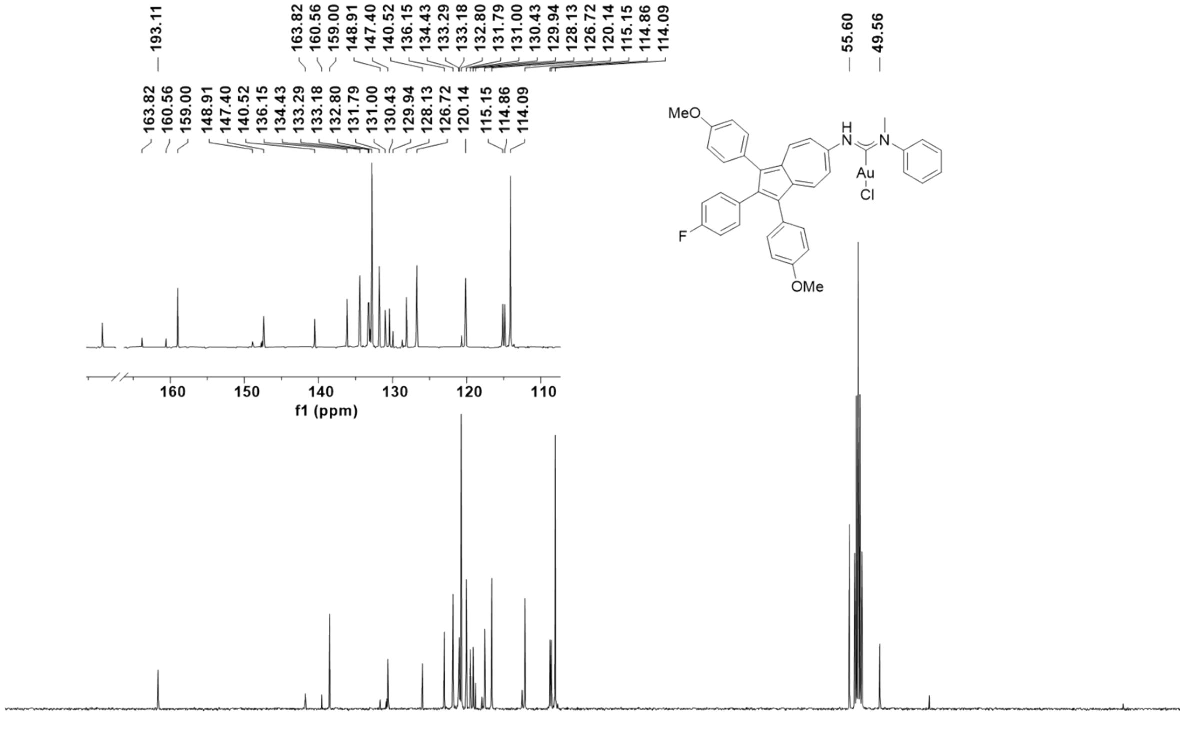

Figure S78. **$^{13}C\{^{19}F,^{1}H\}$ NMR** Spectrum (75 MHz, CD_2Cl_2, 295 K) of **14bh**.

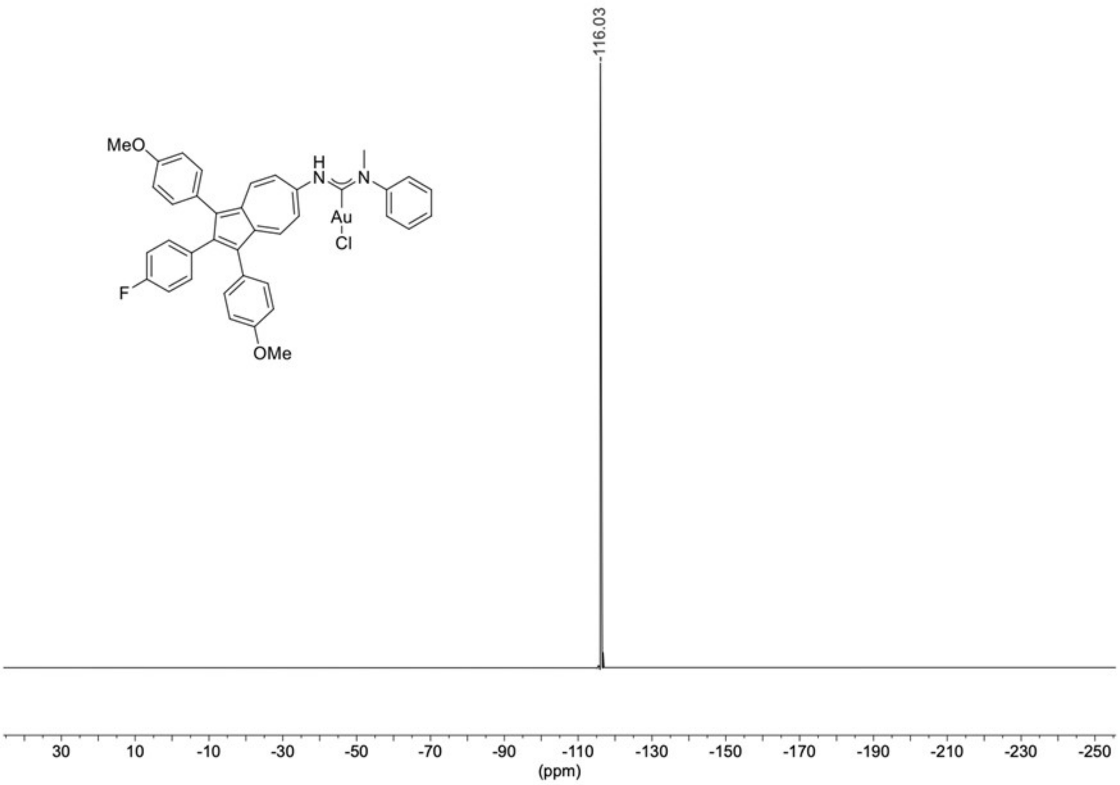

Figure S79. $^{19}F\{^{1}H\}$ **NMR** Spectrum (283 MHz, CD_2Cl_2, 298 K) of **14bh**.

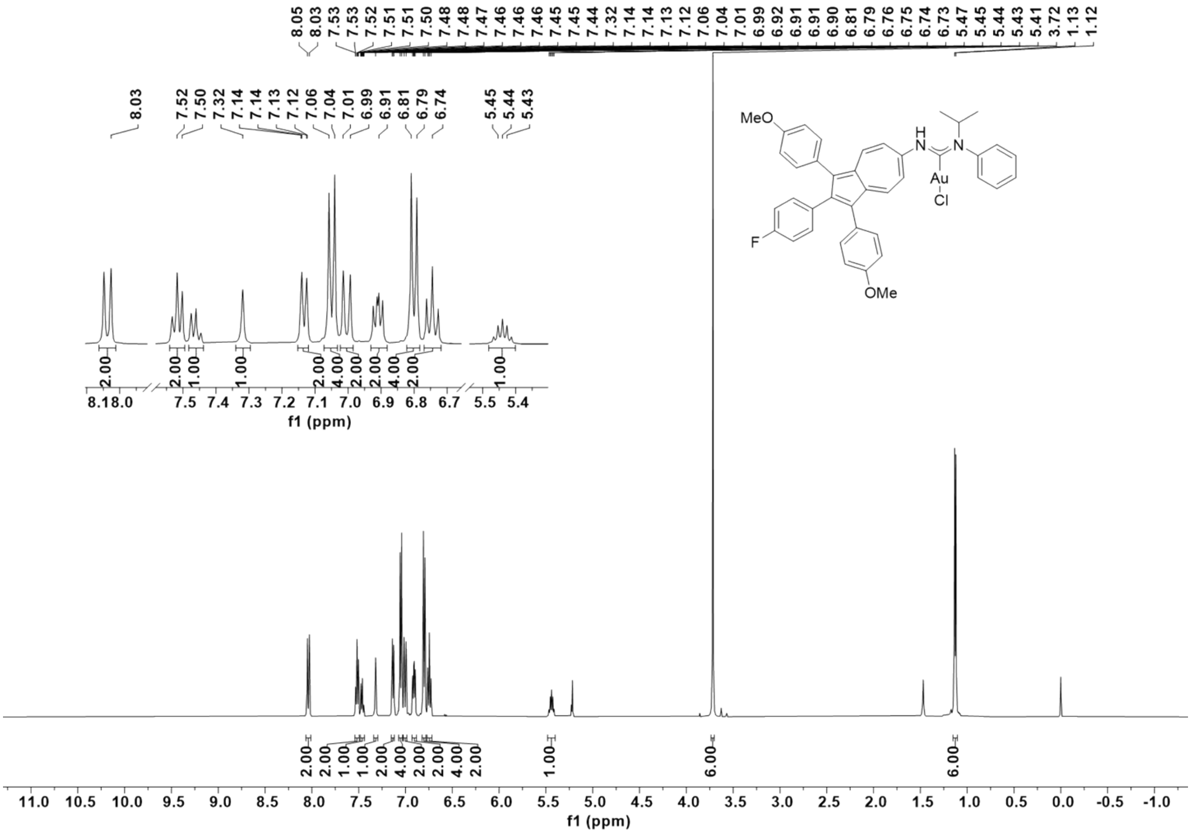

Figure S80. $^{1}H\{^{19}F\}$ **NMR** Spectrum (500 MHz, CD_2Cl_2, 295 K) of **14bi**.

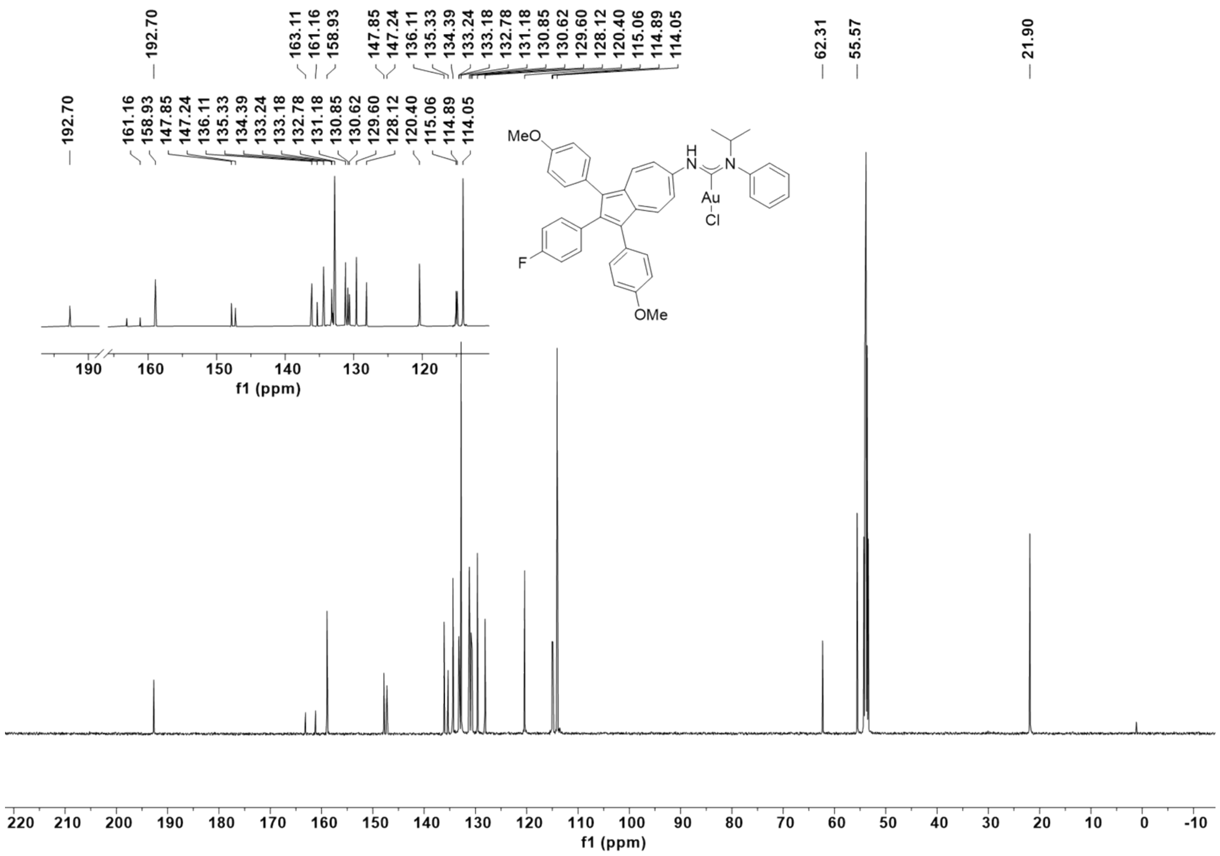

Figure S81. $^{13}C\{^{19}F,^{1}H\}$ **NMR** Spectrum (126 MHz, CD_2Cl_2, 295 K) of **14bi**.

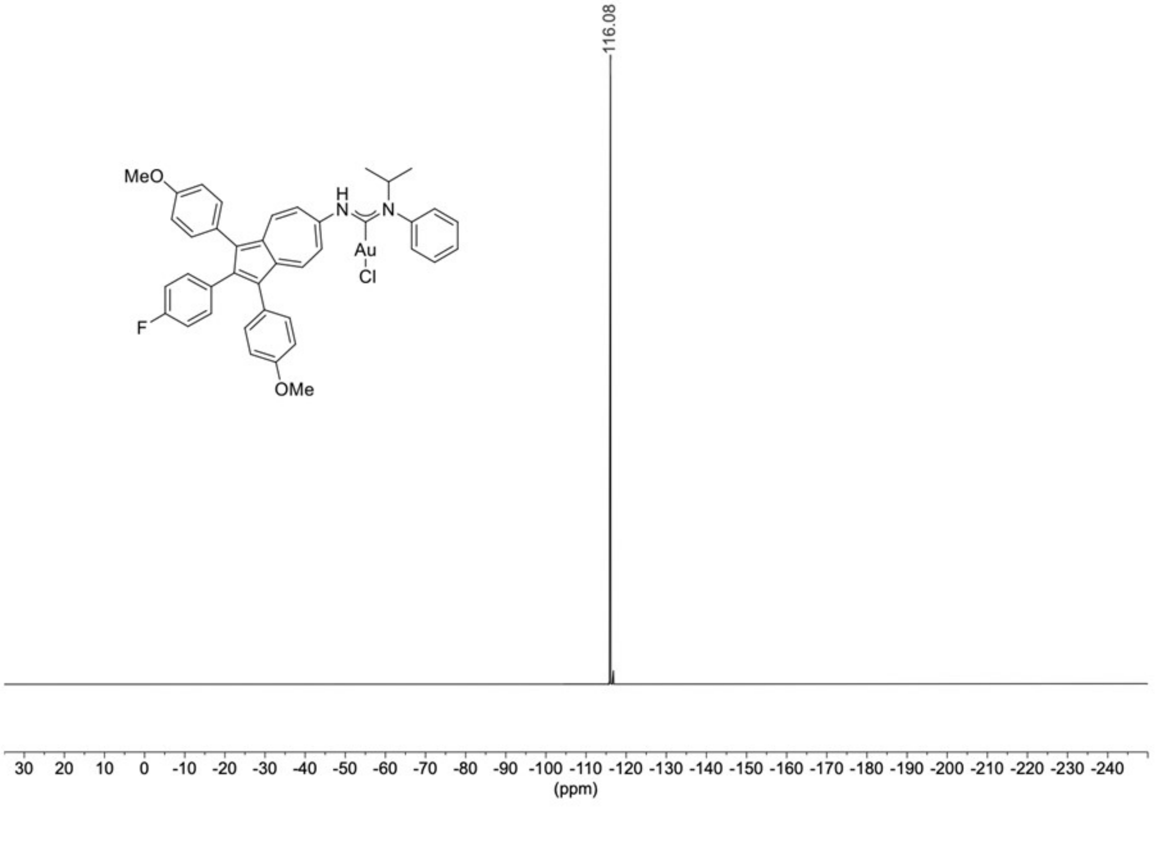

Figure S82. $^{19}F\{^{1}H\}$ **NMR** Spectrum (283 MHz, CD_2Cl_2, 298 K) of **14bi**.

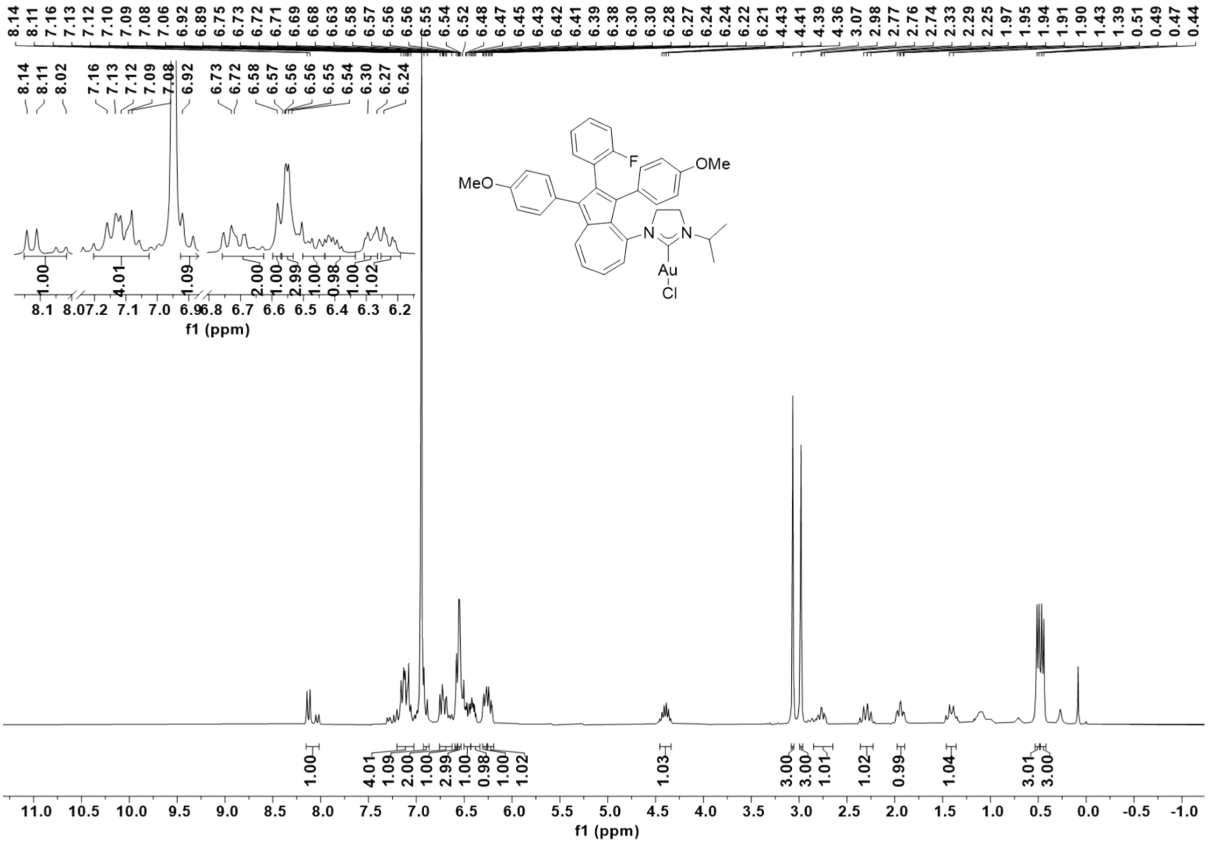

Figure S83. $^{1}H\{^{19}F\}$ **NMR** Spectrum (301 MHz, C_6D_6, 295 K) of **16a**.

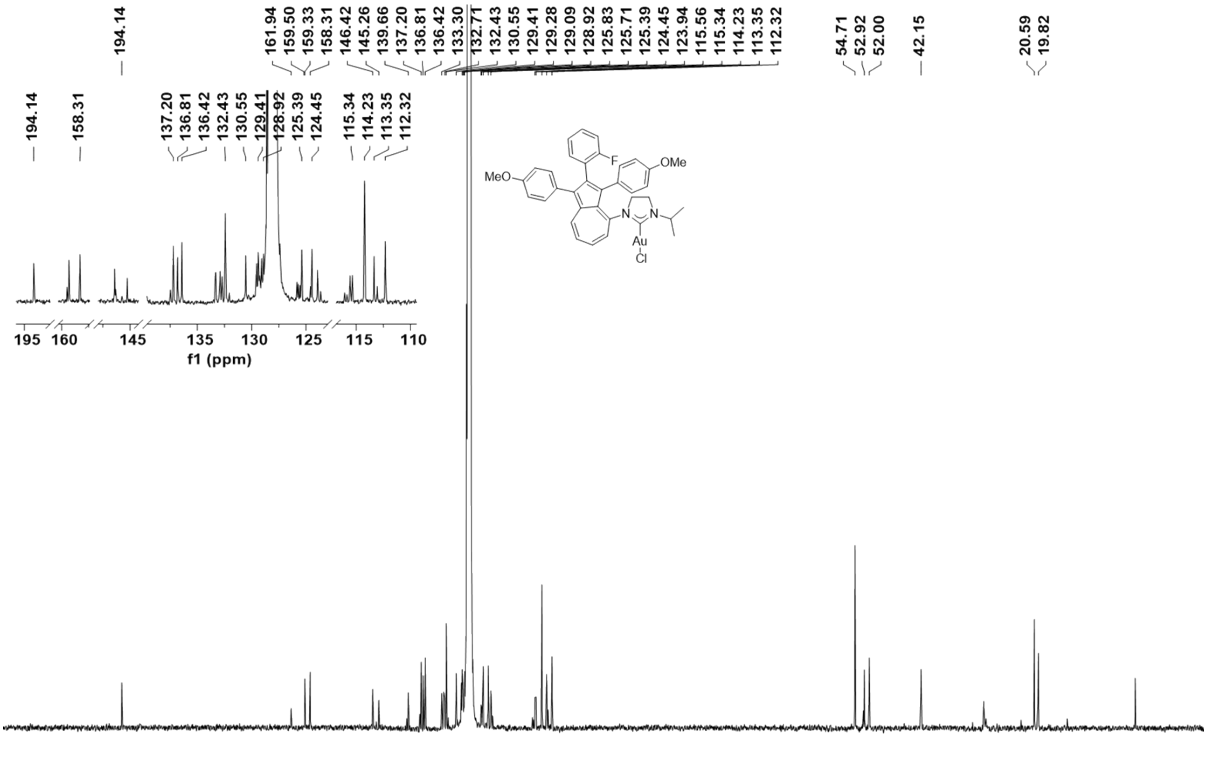

Figure S84. $^{13}C\{^{19}F,^{1}H\}$ **NMR** Spectrum (101 MHz, C_6D_6, 295 K) of **16a**.

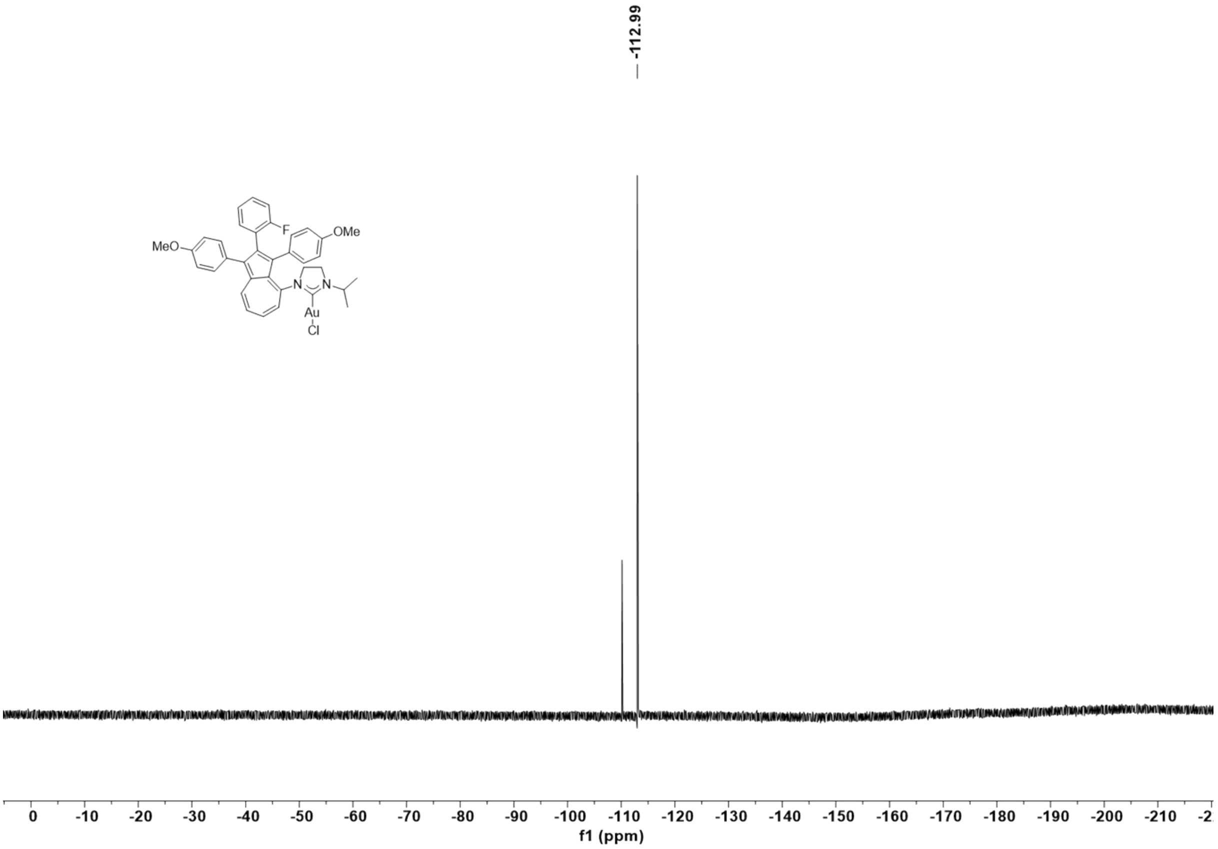

Figure S85. $^{19}F\{^1H\}$ **NMR** Spectrum (283 MHz, C_6D_6, 295 K) of **16a**.

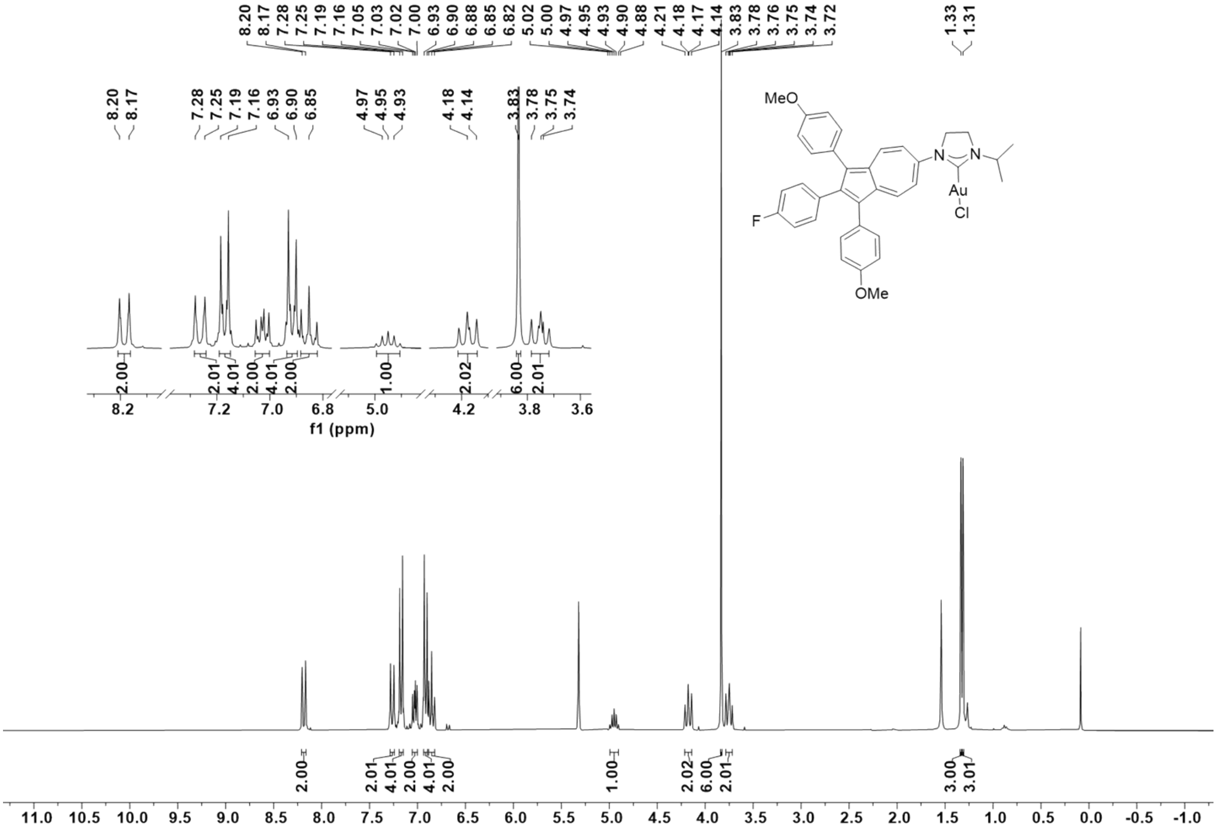

Figure S86. $^1H\{^{19}F\}$ **NMR** Spectrum (300 MHz, CD_2Cl_2, 295 K) of **16b**.

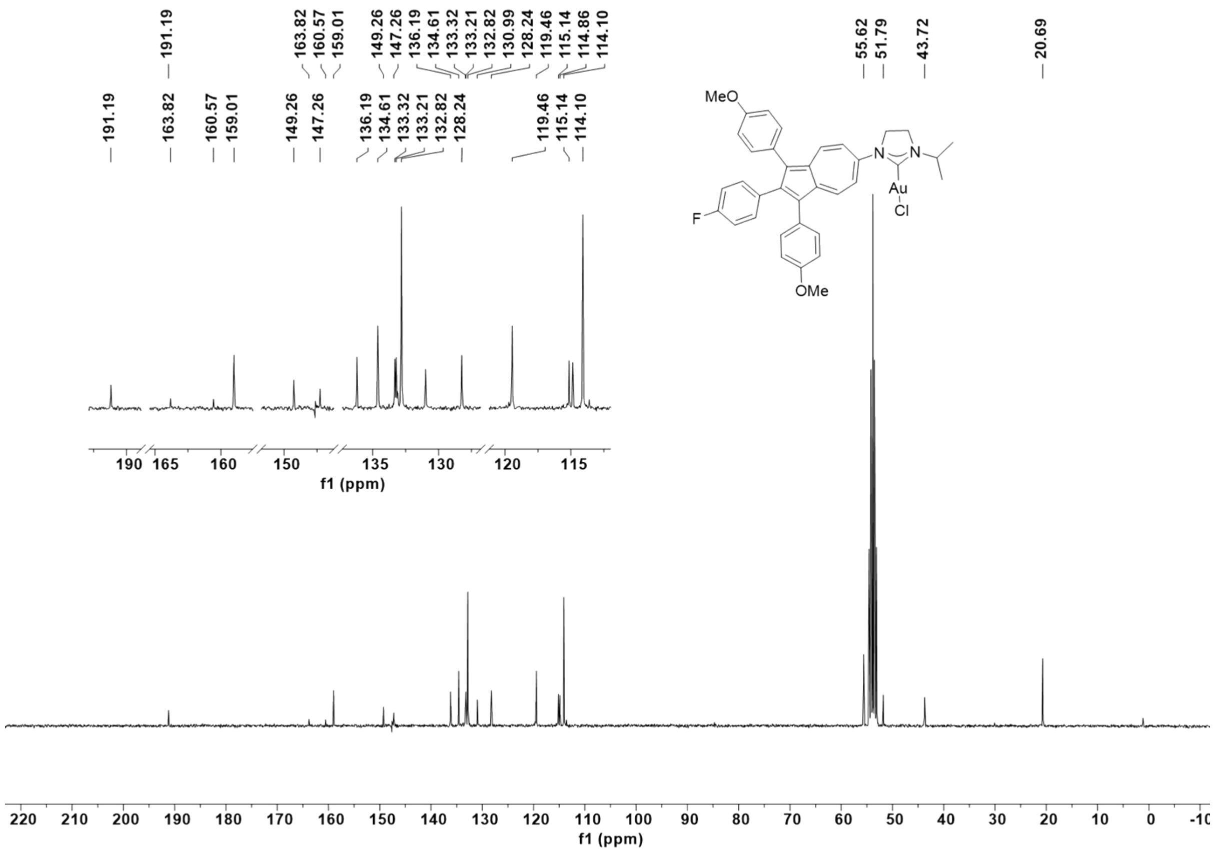

Figure S87. ^{13}**C{**^{19}**F,**^{1}**H} NMR** Spectrum (75 MHz, CD_2Cl_2, 295 K) of **16b**.

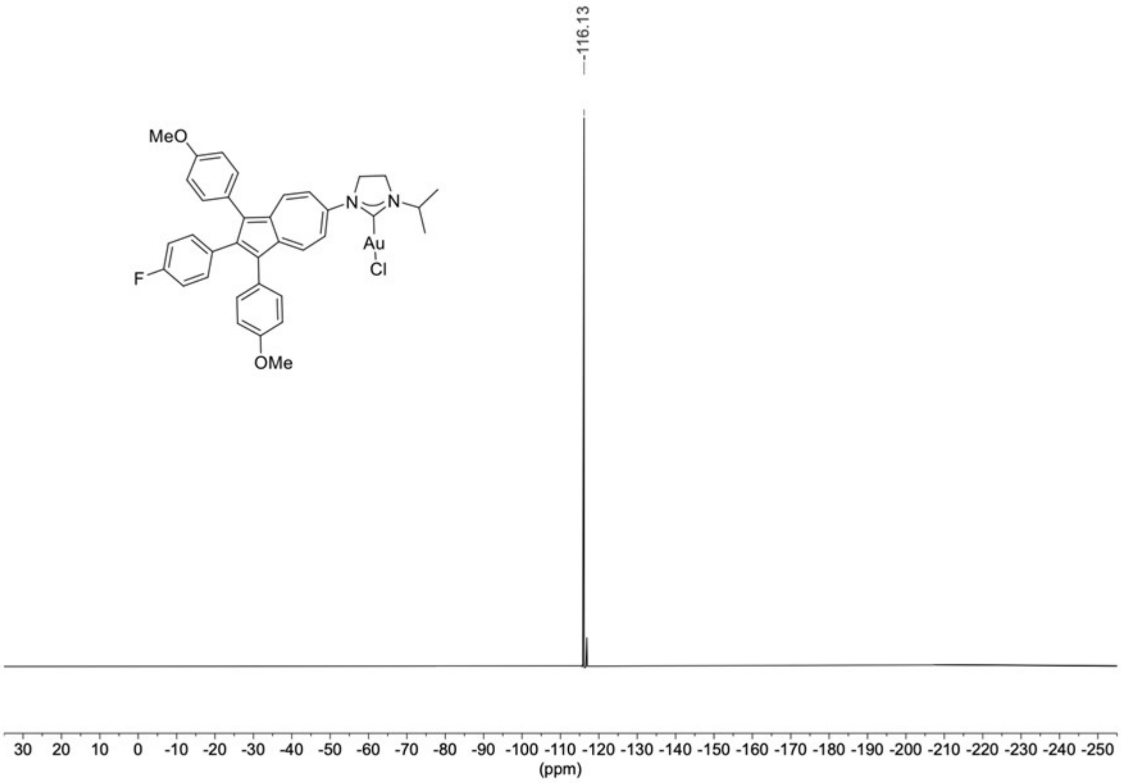

Figure S88. ^{19}**F{**^{1}**H} NMR** Spectrum (283 MHz, CD_2Cl_2, 298 K) of **16b**.

5 IR Spectra

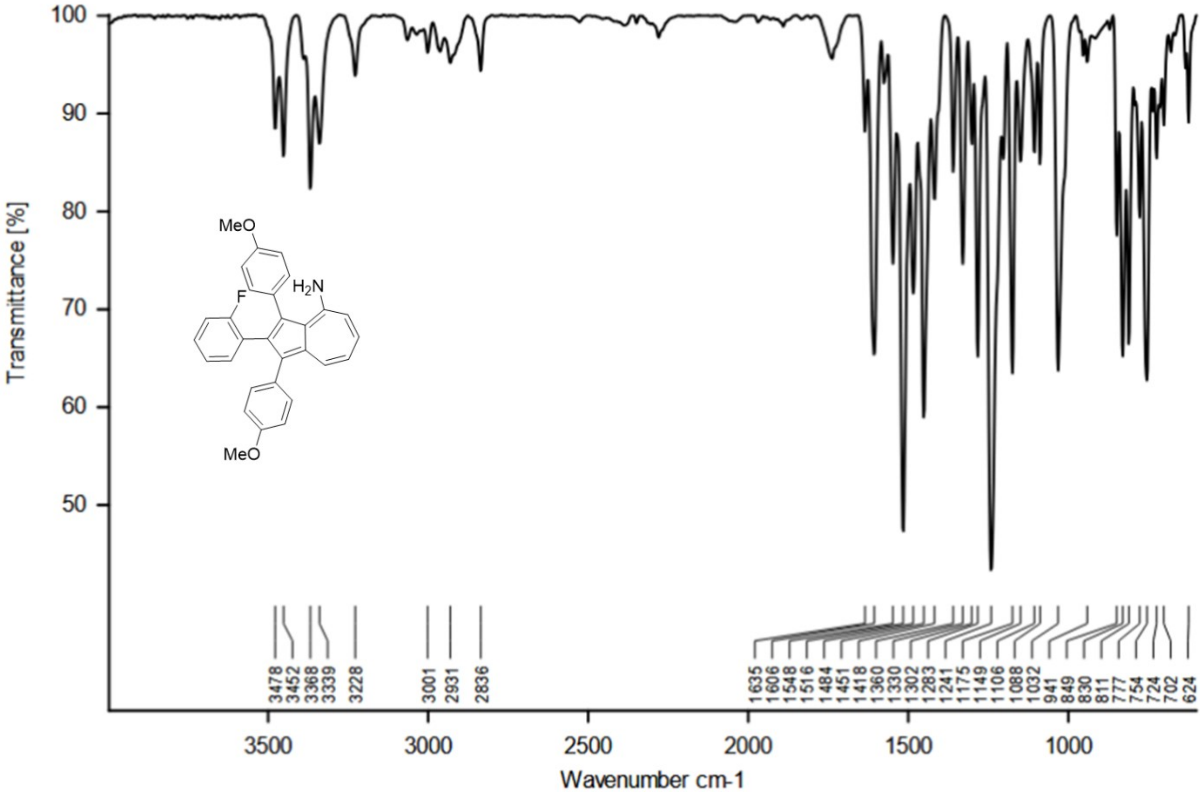

Figure S89. IR (ATR) Spectrum of **4a**.

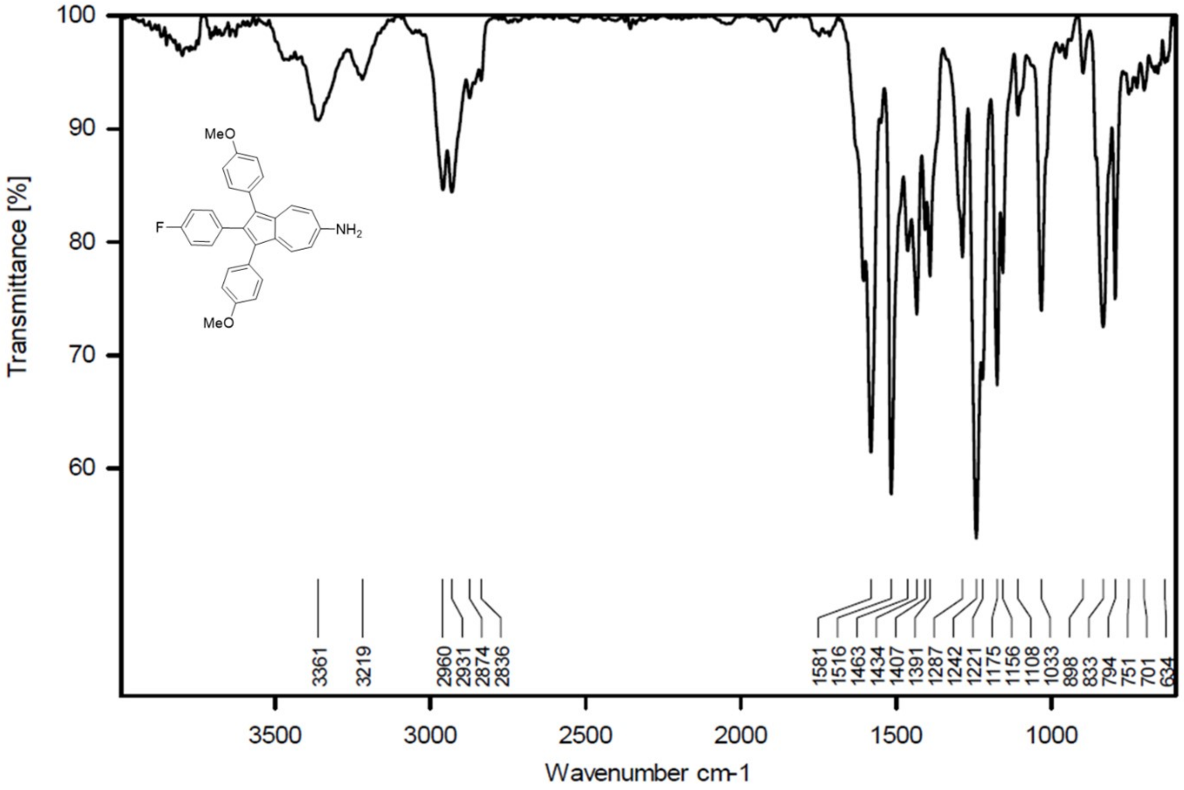

Figure S90. IR (ATR) Spectrum of **4b**.

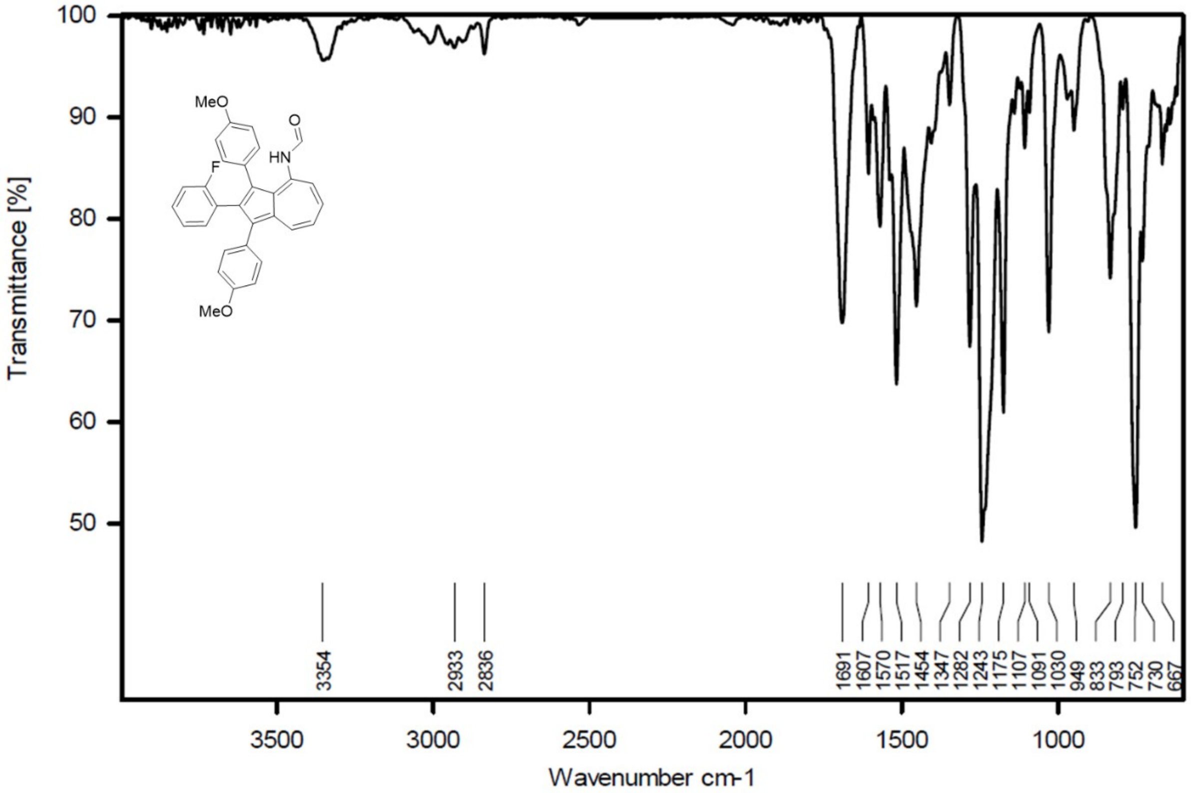

Figure S91. IR (ATR) Spectrum of **6a**.

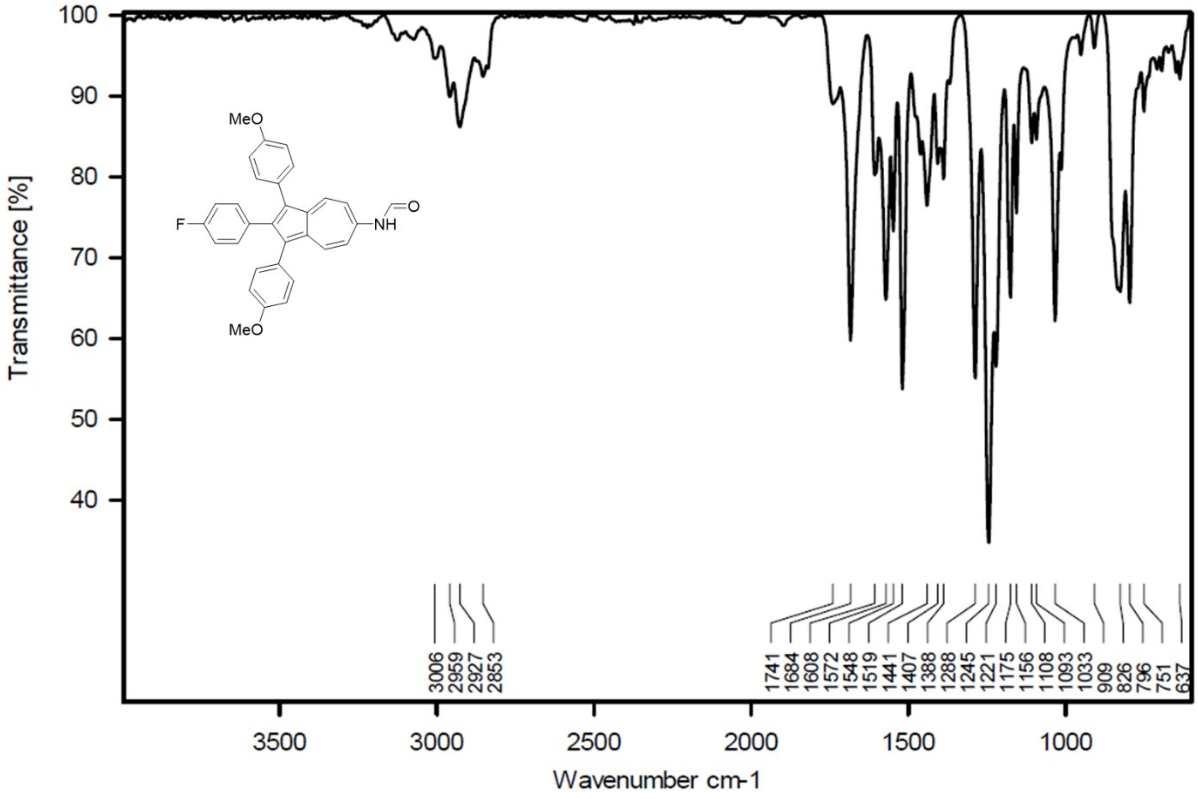

Figure S92. IR (ATR) Spectrum of **6b**.

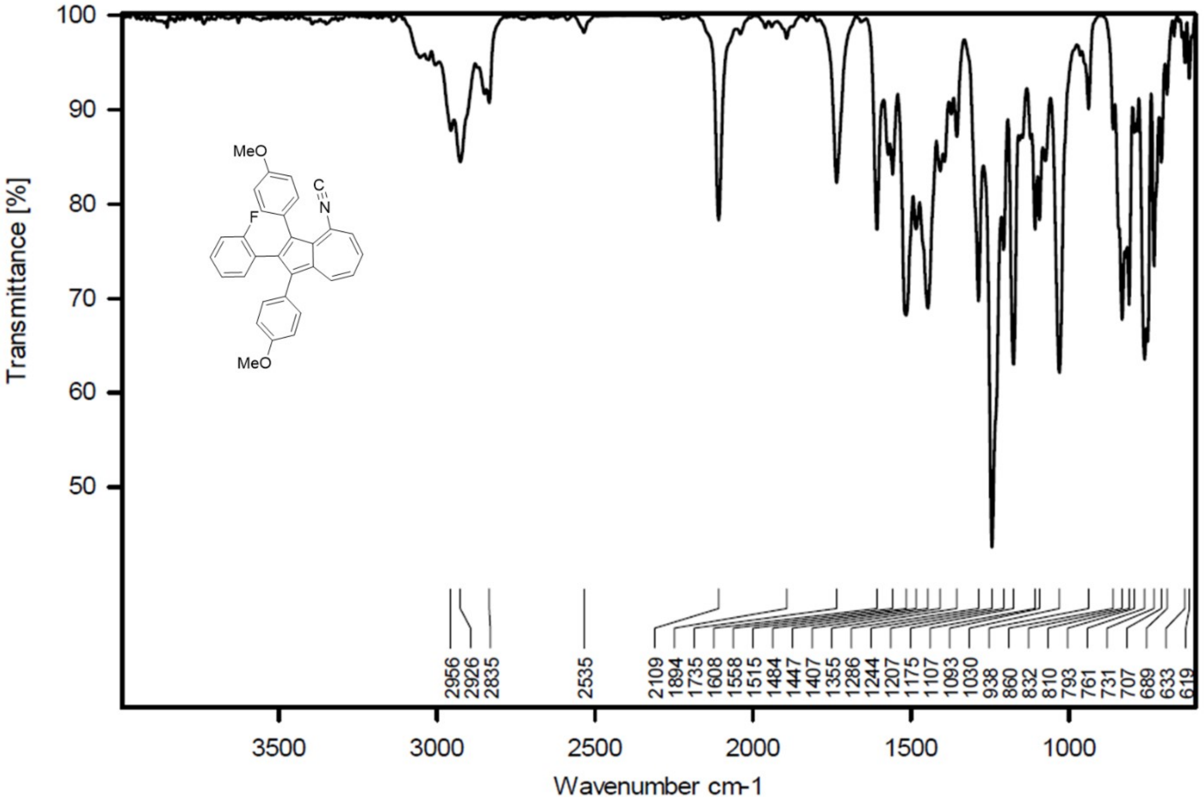

Figure S93. IR (ATR) Spectrum of **10a**.

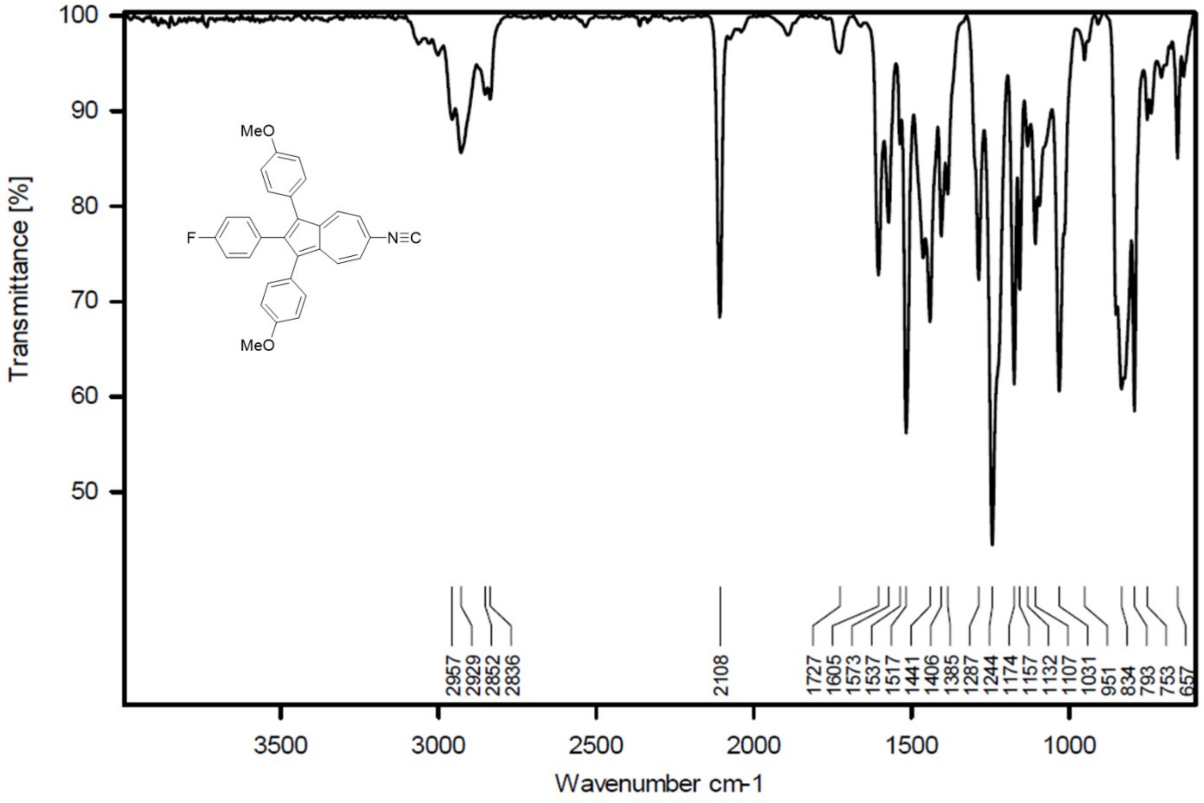

Figure S94. IR (ATR) Spectrum of **10b**.

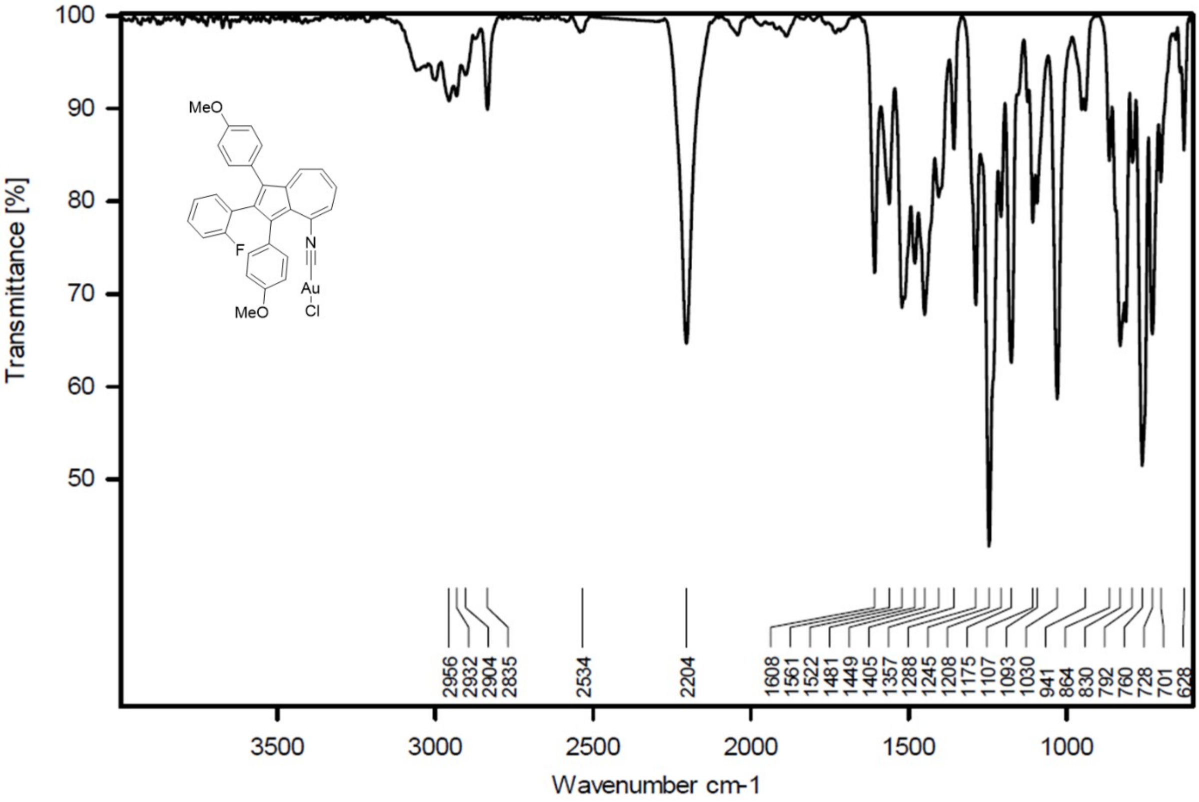

Figure S95. IR (ATR) Spectrum of **12a**.

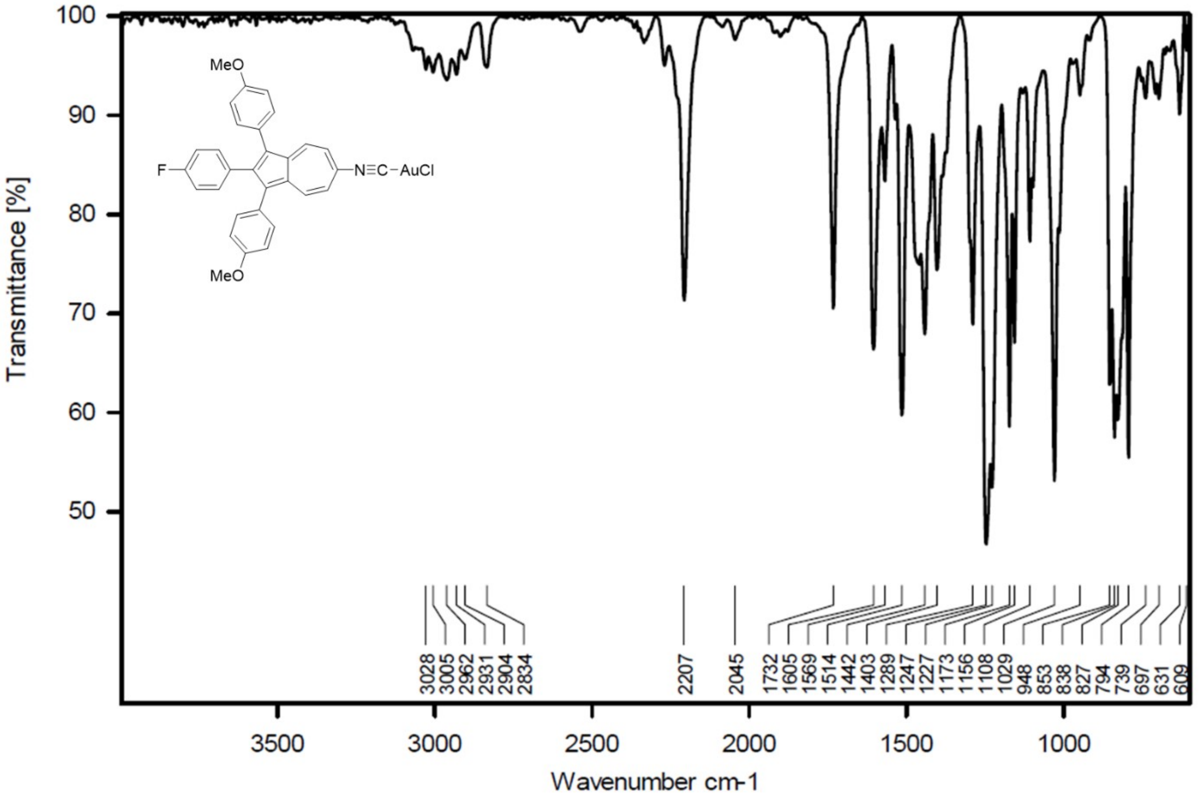

Figure S96. IR (ATR) Spectrum of **12b**.

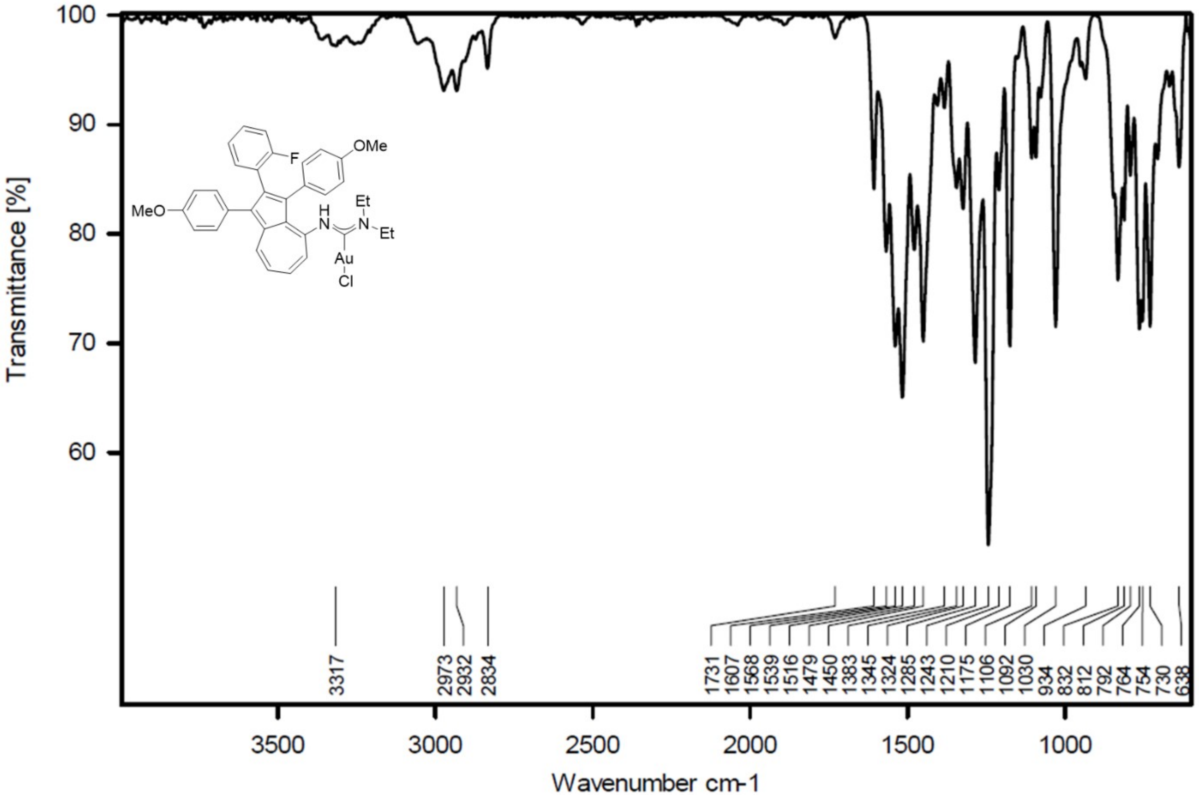

Figure S97. IR (ATR) Spectrum of **14aa**.

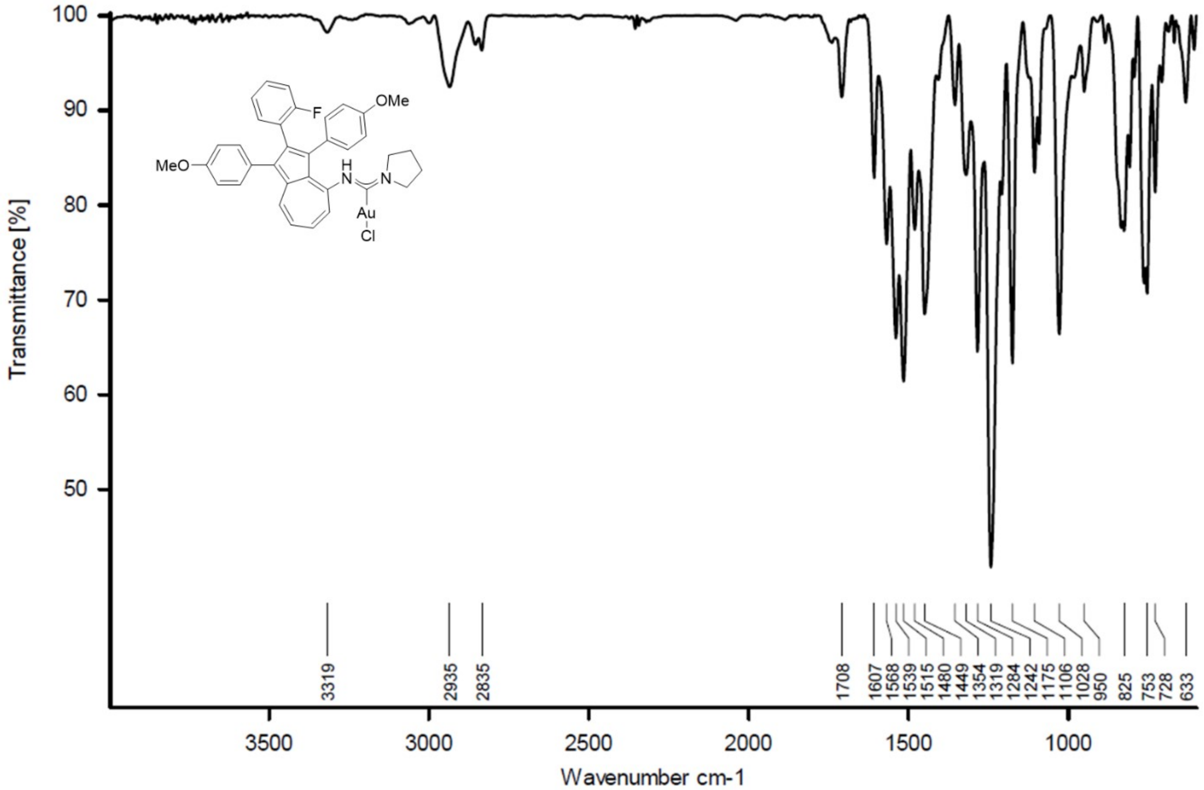

Figure S98. IR (ATR) Spectrum of **14ab**.

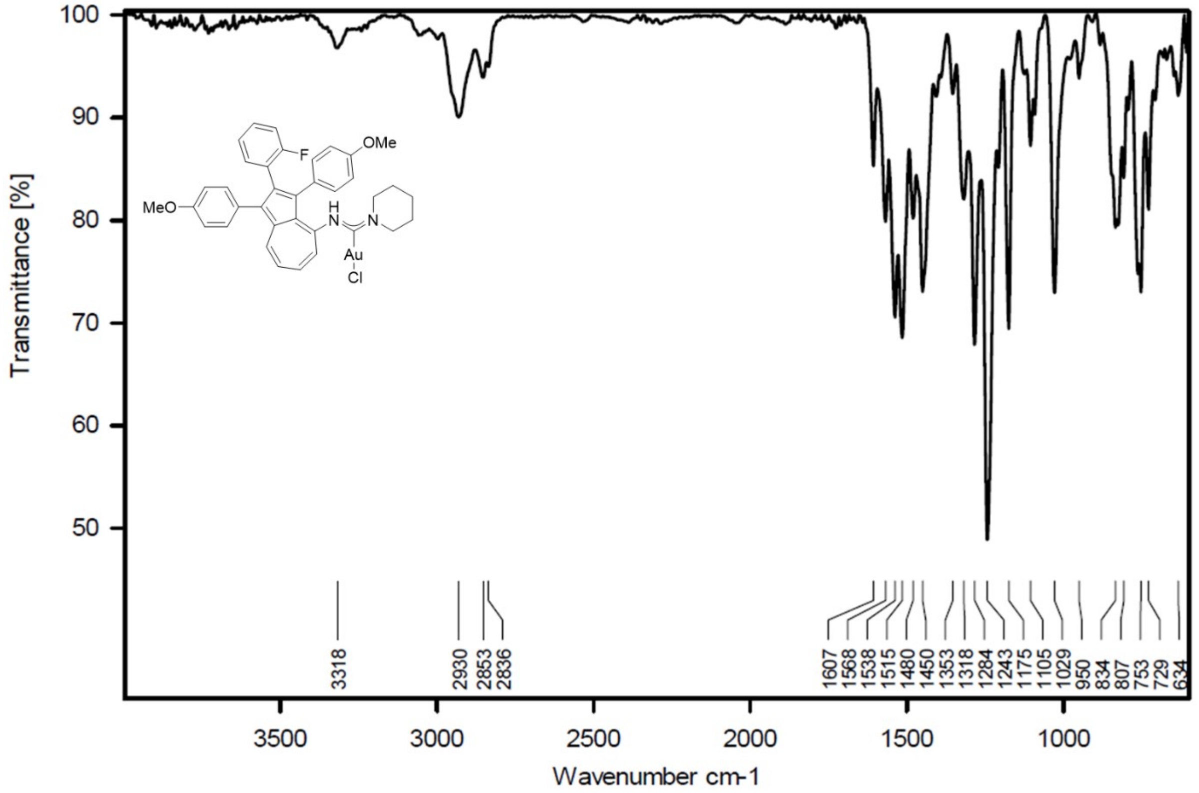

Figure S99. IR (ATR) Spectrum of **14ac**.

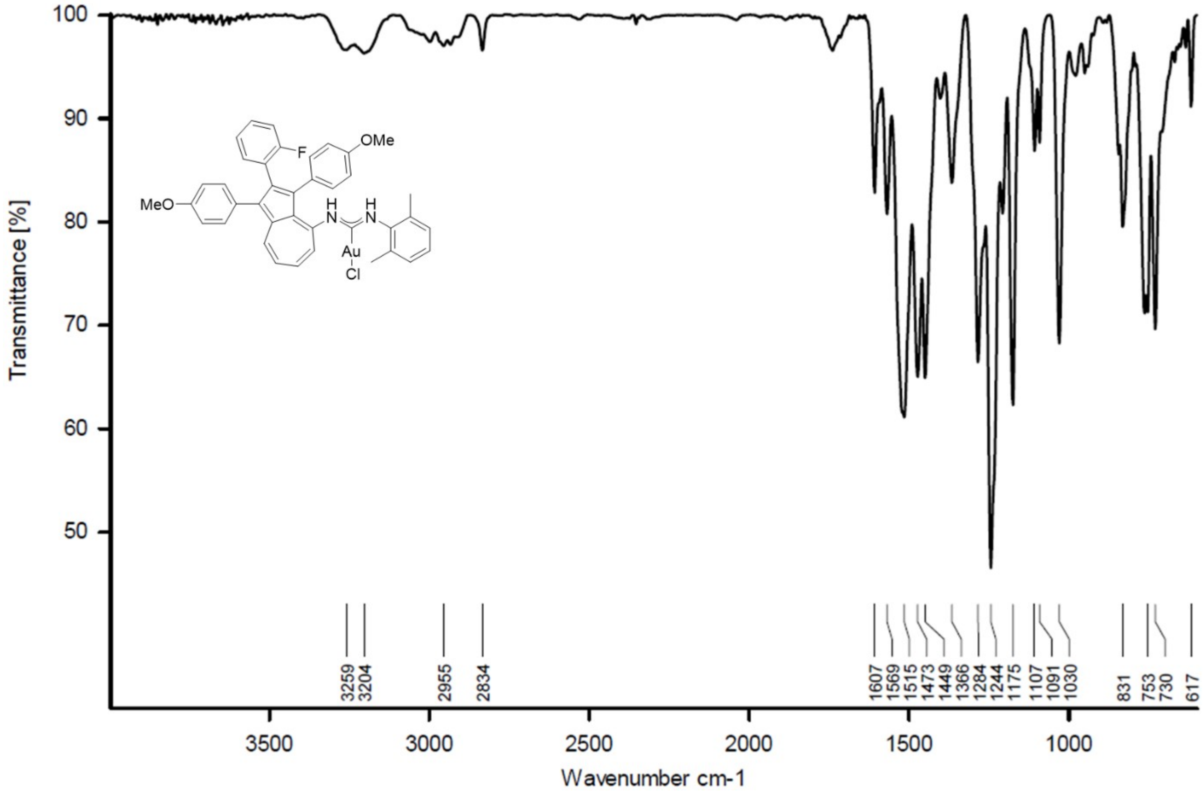

Figure S100. IR (ATR) Spectrum of **14ad**.

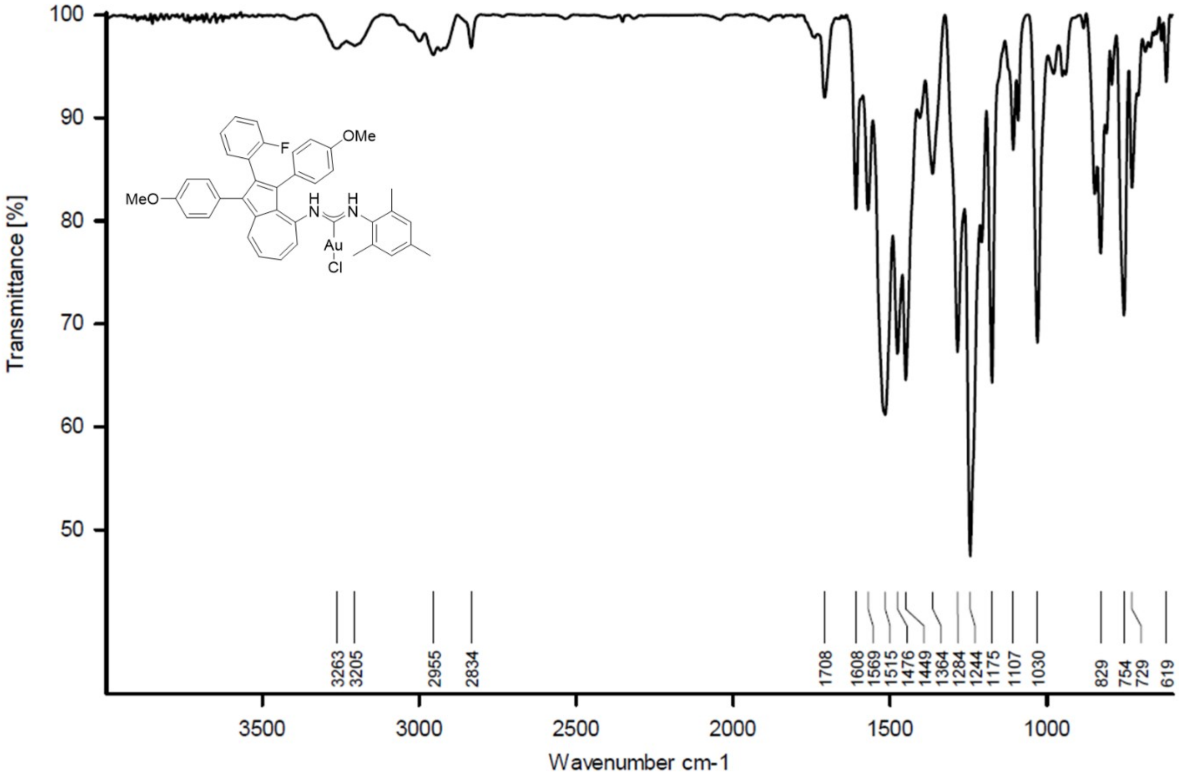

Figure S101. IR (ATR) Spectrum of **14ae**.

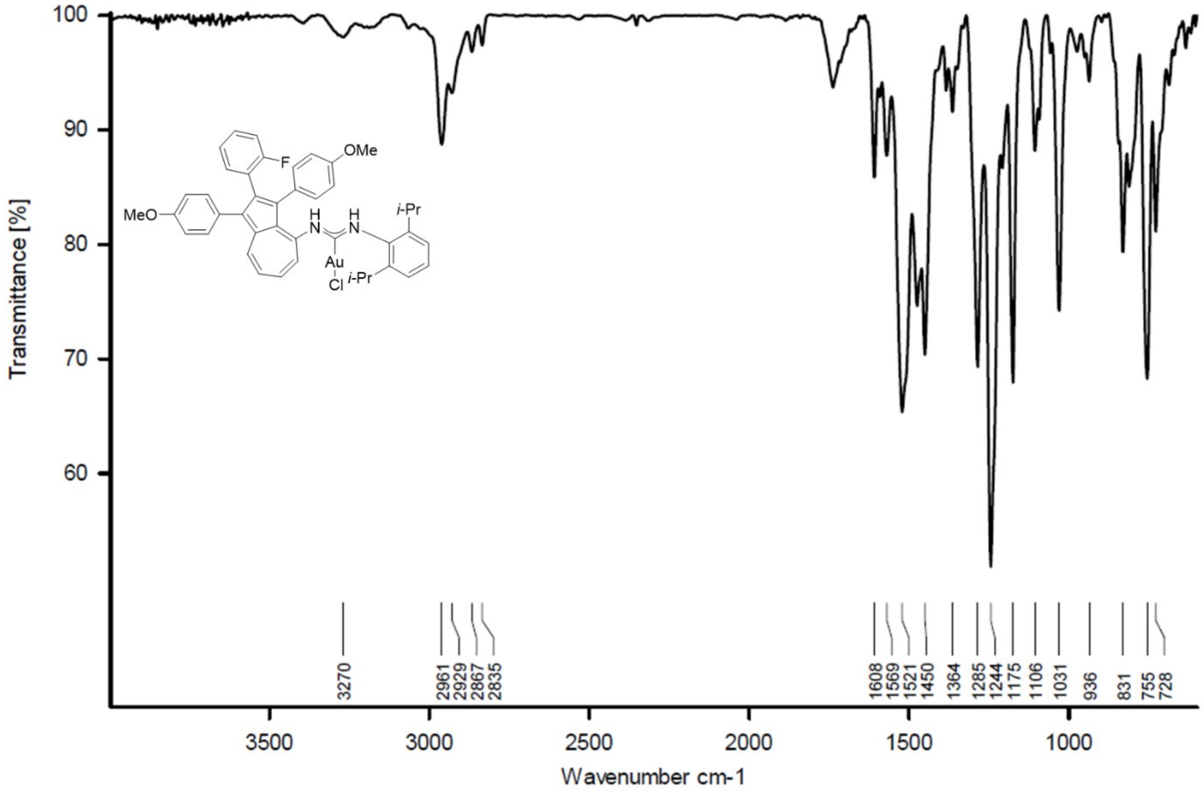

Figure S102. IR (ATR) Spectrum of **14af**.

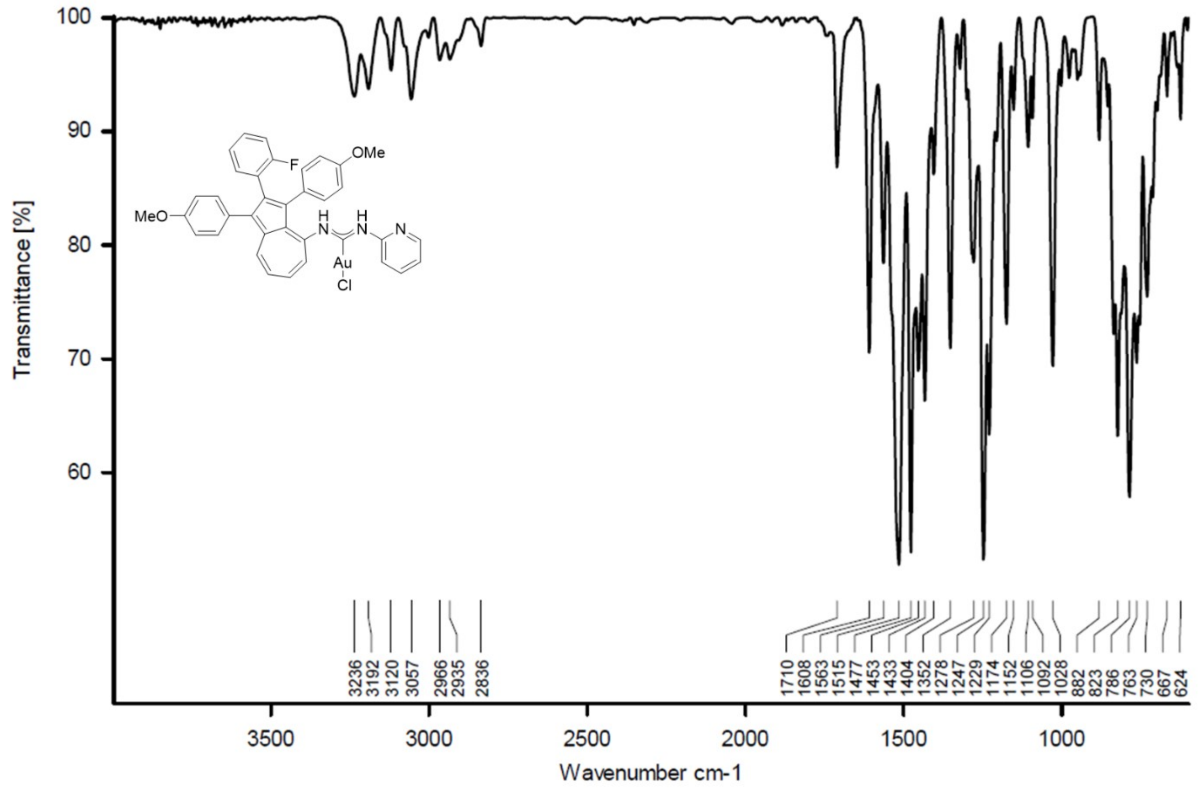

Figure S103. IR (ATR) Spectrum of **14ag**.

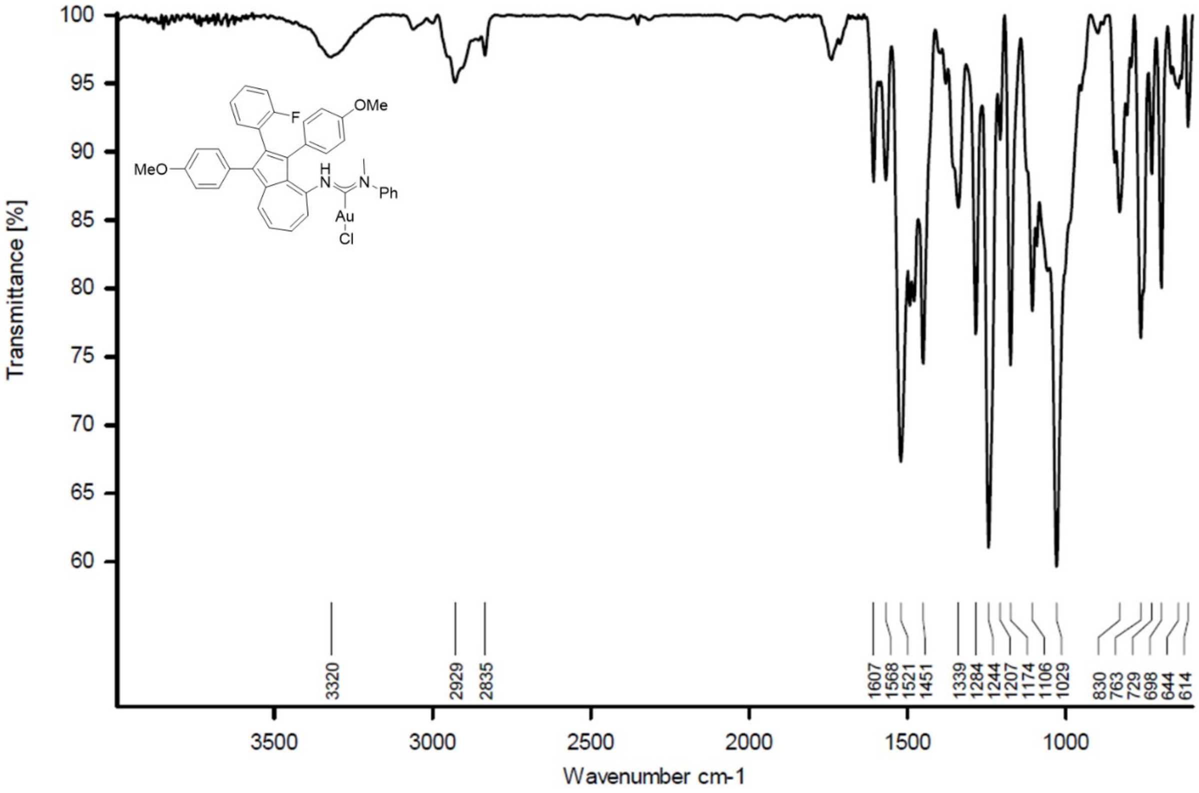

Figure S104. IR (ATR) Spectrum of **14ah**.

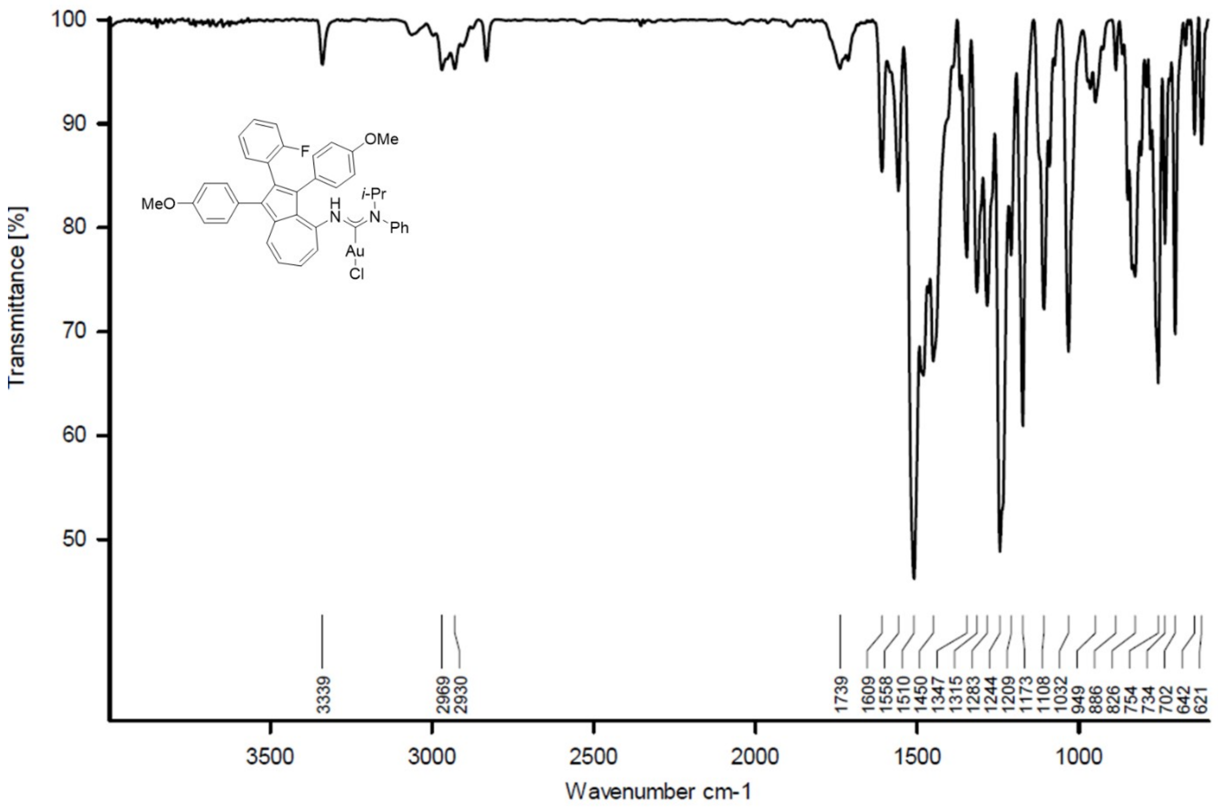

Figure S105. IR (ATR) Spectrum of **14ai**.

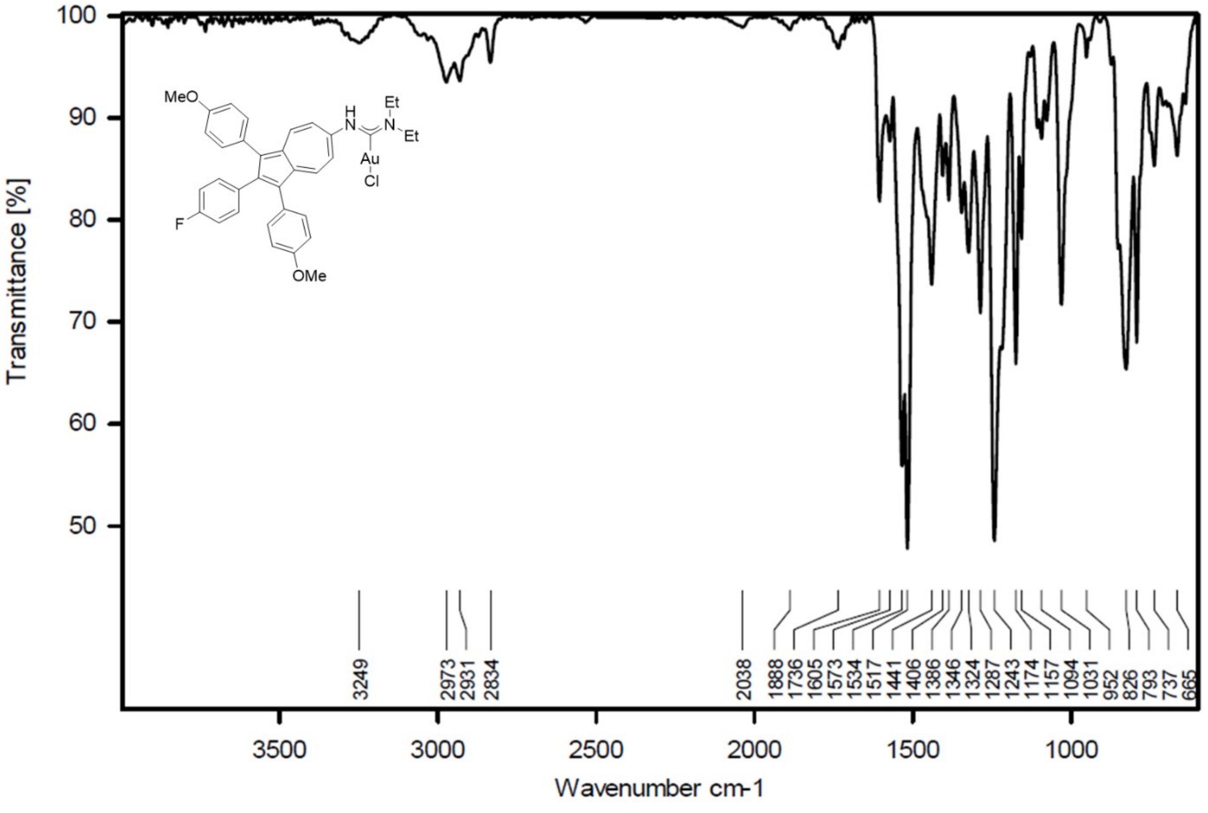

Figure S106. IR (ATR) Spectrum of **14ba**.

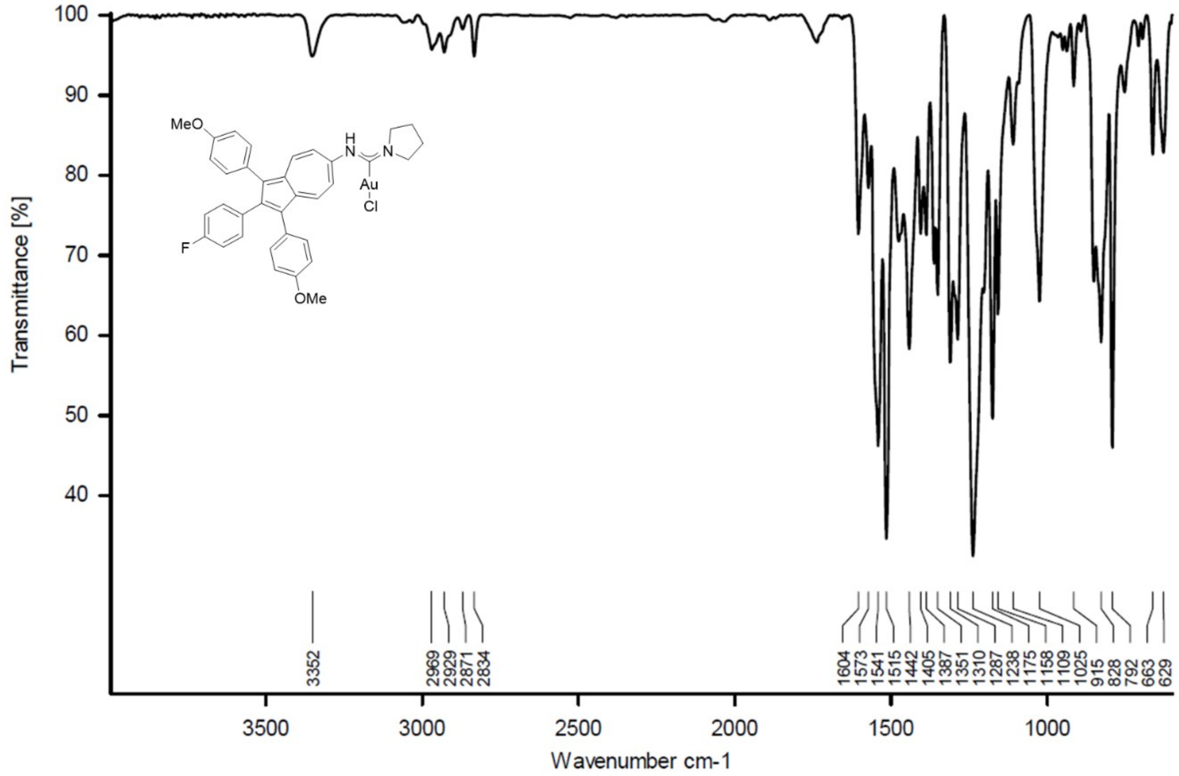

Figure S107. IR (ATR) Spectrum of **14bb**.

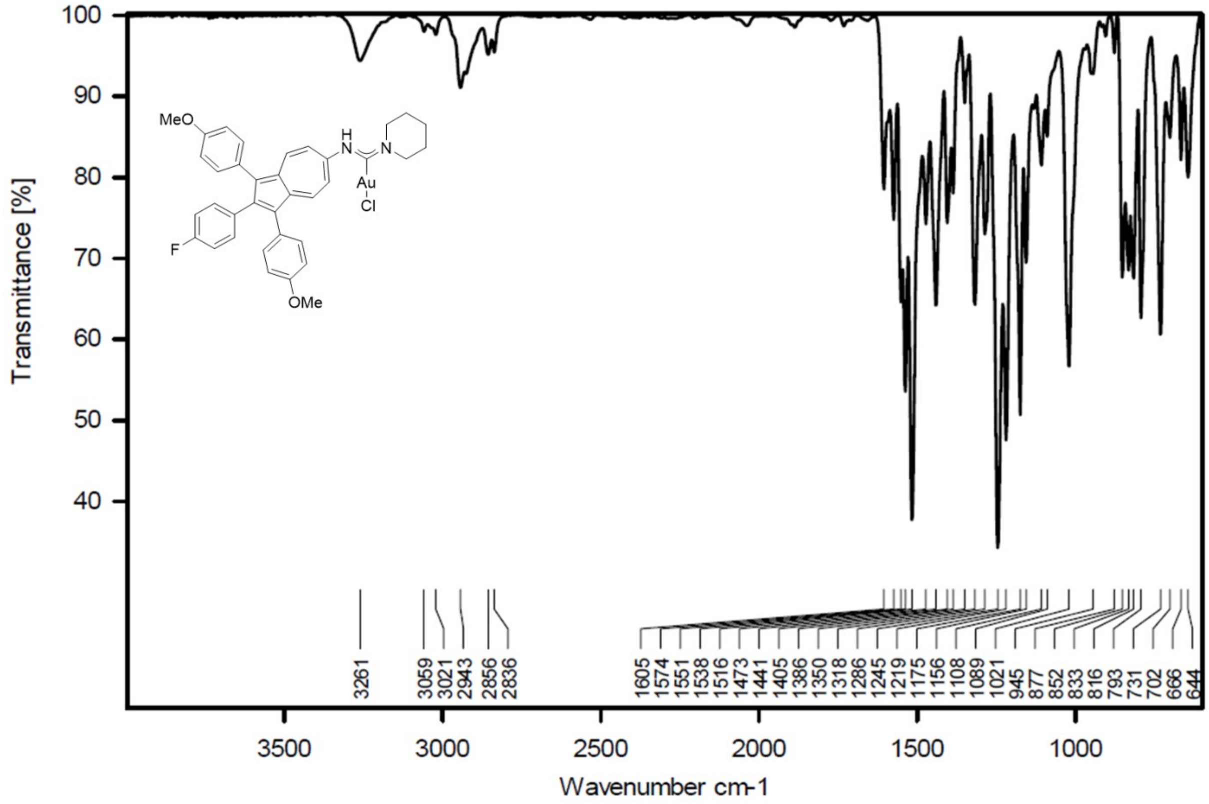

Figure S108. IR (ATR) Spectrum of **14bc**.

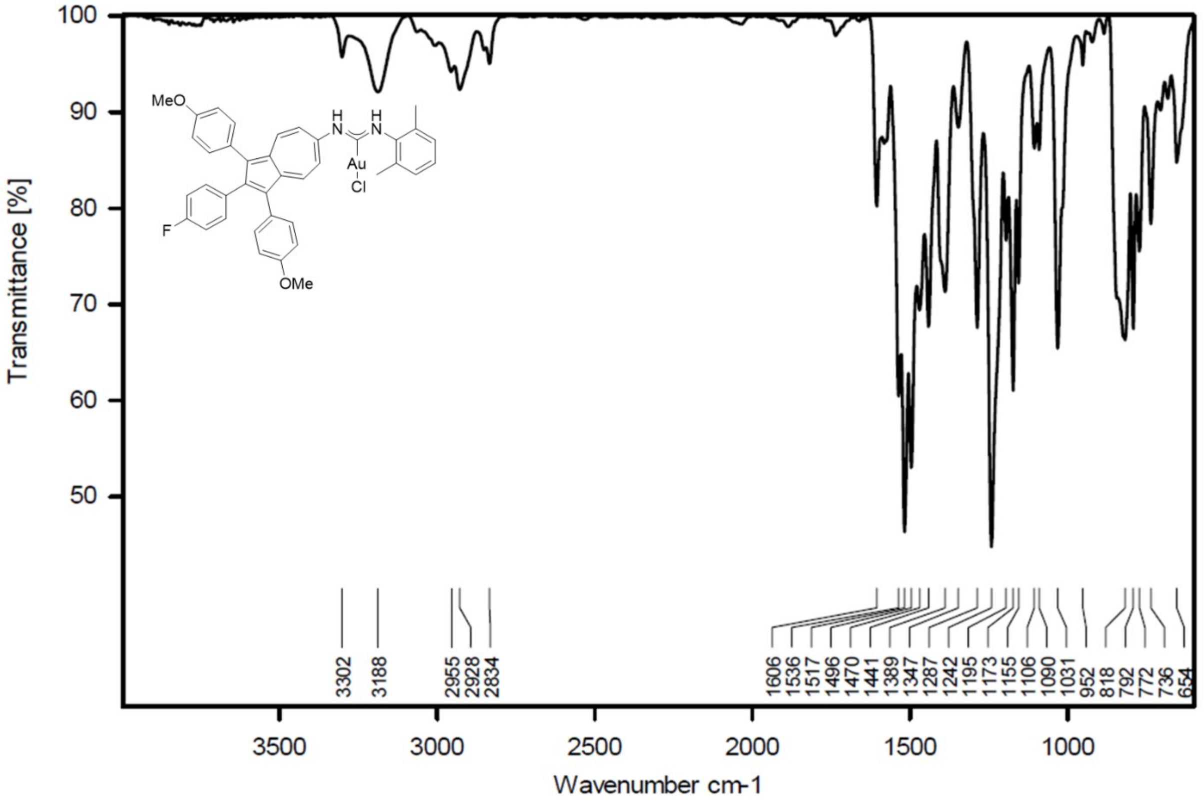

Figure S109. IR (ATR) Spectrum of **14bd**.

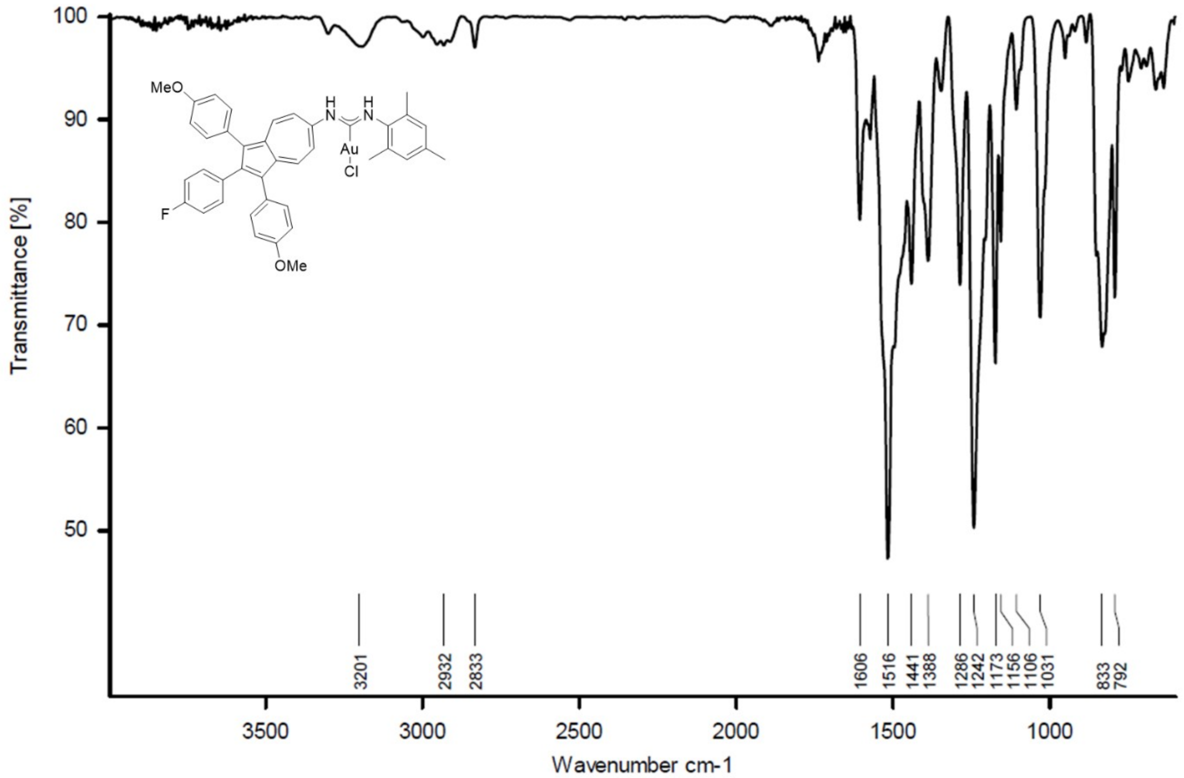

Figure S110. IR (ATR) Spectrum of **14be**.

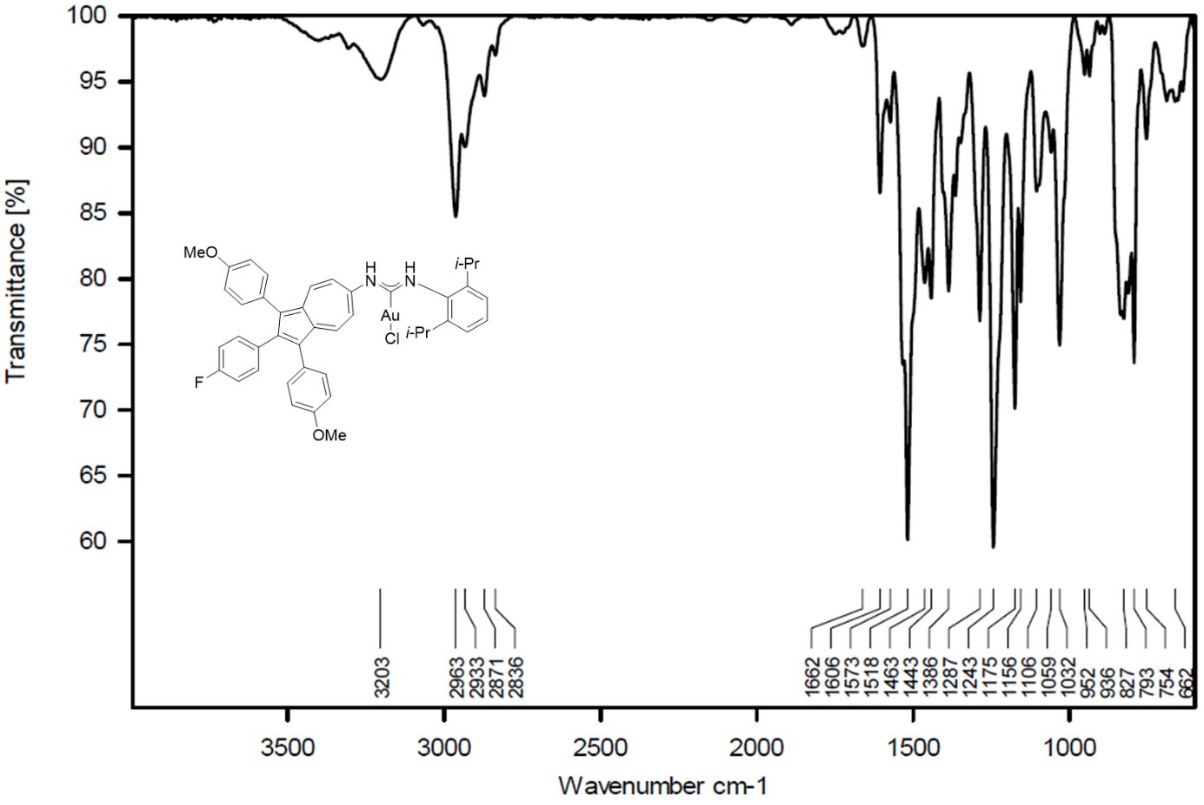

Figure S111. IR (ATR) Spectrum of **14bf**.

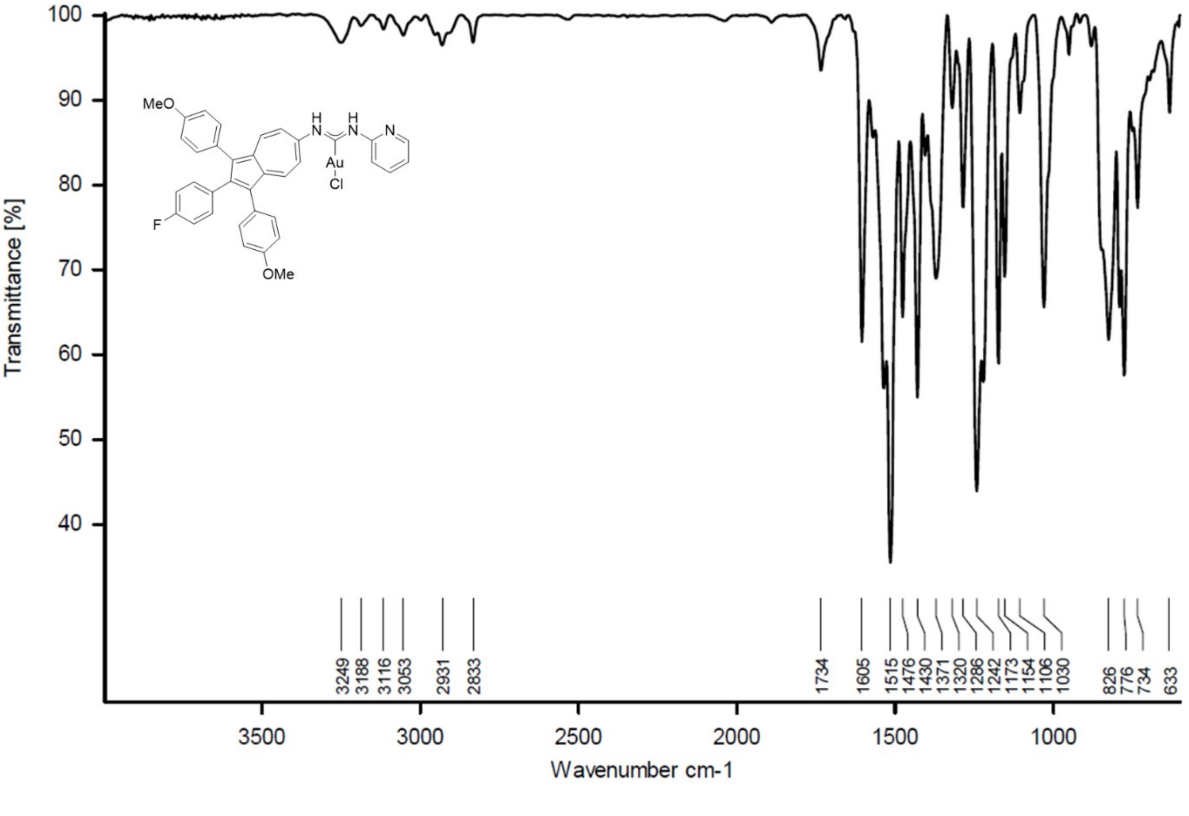

Figure S112. IR (ATR) Spectrum of **14bg**.

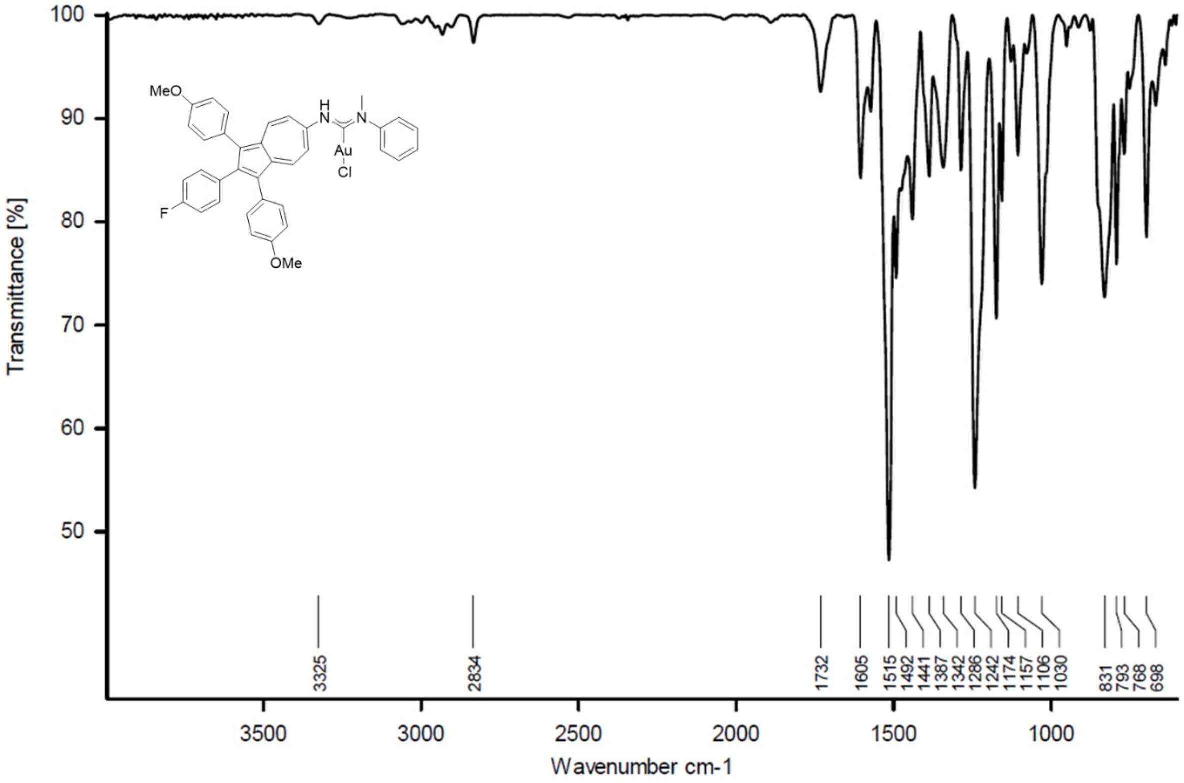

Figure S113. IR (ATR) Spectrum of **14bh**.

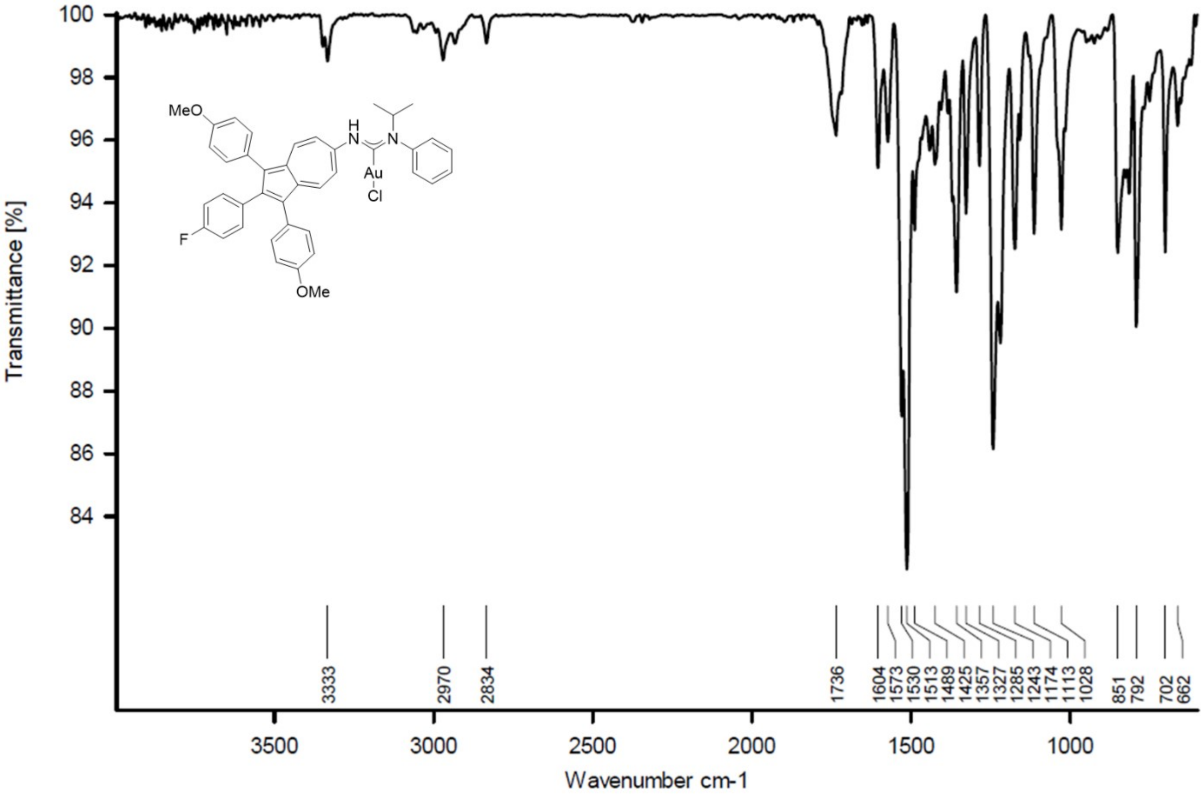

Figure S114. IR (ATR) Spectrum of **14bi**.

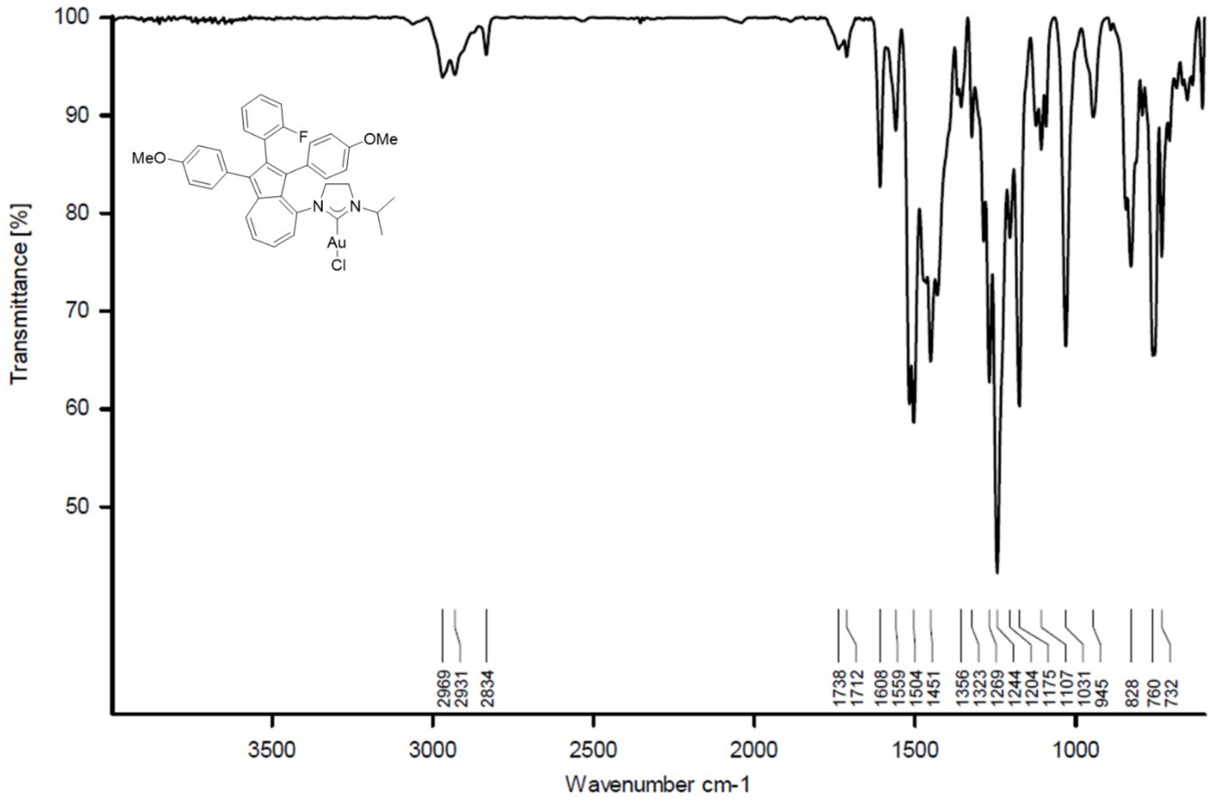

Figure S115. IR (ATR) Spectrum of **16a**.

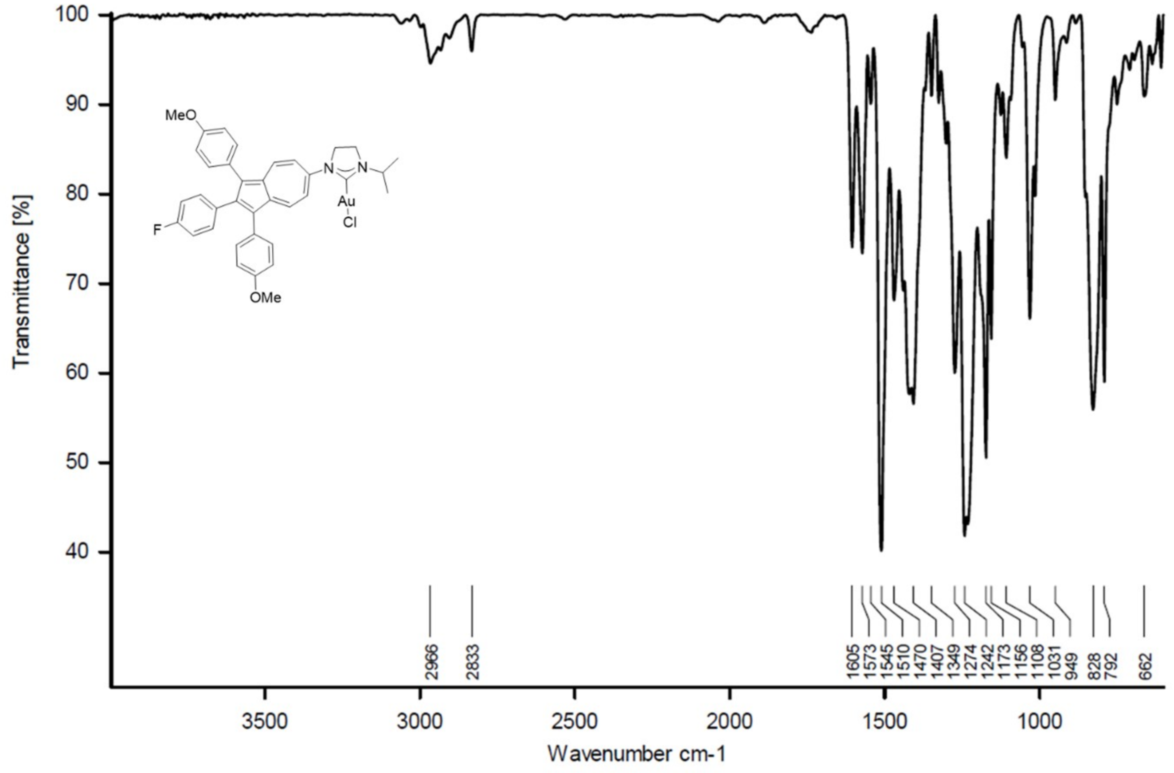

Figure S116. IR (ATR) Spectrum of **16b**.

6 Single Crystal Structures

6.1 Single Crystal Structure of 6b

Identification code	mad3	
Empirical formula	$C_{33}H_{26}Cl_6FNO_3$	
Formula weight	716.25	
Temperature	200(2) K	
Wavelength	0.71073 Å	
Crystal system	monoclinic	
Space group	C2/c	
Z	8	
Unit cell dimensions	a = 23.3655(7) Å	α = 90 deg.
	b = 9.5263(3) Å	β =108.2397(19) deg.
	c = 30.8642(10) Å	γ = 90 deg.
Volume	6524.8(4) $Å^3$	
Density (calculated)	1.46 g/cm^3	
Absorption coefficient	0.57 mm^{-1}	
Crystal shape	plank	
Crystal size	0.101 x 0.069 x 0.020 mm^3	
Crystal colour	green	
Theta range for data collection	1.4 to 22.7 deg.	
Index ranges	$-25 \le h \le 25$, $-10 \le k \le 10$, $-33 \le l \le 33$	
Reflections collected	32400	
Independent reflections	4405 (R(int) = 0.0902)	
Observed reflections	2853 ($I > 2\sigma(I)$)	
Absorption correction	Semi-empirical from equivalents	
Max. and min. transmission	0.96 and 0.90	
Refinement method	Full-matrix least-squares on F^2	
Data/restraints/parameters	4405 / 0 / 399	
Goodness-of-fit on F^2	1.03	
Final R indices (I>2sigma(I))	R1 = 0.080, wR2 = 0.207	
Largest diff. peak and hole	0.93 and -0.76 $eÅ^{-3}$	

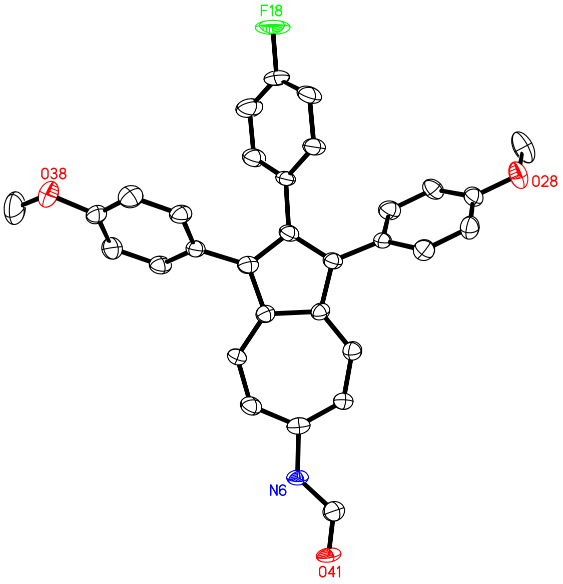

6.2 Single Crystal Structure of 10a

Identification code	mad2	
Empirical formula	$C_{31}H_{22}AuClFNO_2$	
Formula weight	691.91	
Temperature	200(2) K	
Wavelength	0.71073 Å	
Crystal system	monoclinic	
Space group	$P2_1/c$	
Z	4	
Unit cell dimensions	a = 12.9318(6) Å	α = 90 deg.
	b = 13.3667(6) Å	β =110.4578(12) deg.
	c = 15.7531(7) Å	γ = 90 deg.
Volume	2551.3(2) $Å^3$	
Density (calculated)	1.80 g/cm^3	
Absorption coefficient	5.91 mm^{-1}	
Crystal shape	stick	
Crystal size	0.078 x 0.035 x 0.026 mm^3	
Crystal colour	green	
Theta range for data collection	1.7 to 25.1 deg.	
Index ranges	$-15 \le h \le 15$, $-15 \le k \le 15$, $-18 \le l \le 18$	
Reflections collected	39986	
Independent reflections	4513 (R(int) = 0.1145)	
Observed reflections	3213 ($I > 2\sigma(I)$)	
Absorption correction	Semi-empirical from equivalents	
Max. and min. transmission	0.89 and 0.75	
Refinement method	Full-matrix least-squares on F^2	
Data/restraints/parameters	4513 / 0 / 336	
Goodness-of-fit on F^2	1.03	
Final R indices (I>2sigma(I))	R1 = 0.037, wR2 = 0.061	
Largest diff. peak and hole	1.01 and -0.75 $eÅ^{-3}$	

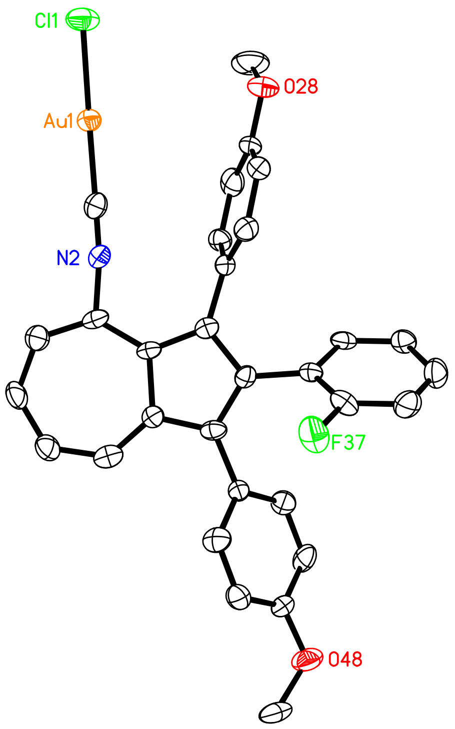

6.3 Single Crystal Structure of 10b

Identification code	mad1	
Empirical formula	$C_{32}H_{24}AuCl_3FNO_2$	
Formula weight	776.84	
Temperature	200(2) K	
Wavelength	0.71073 Å	
Crystal system	monoclinic	
Space group	$P2_1/n$	
Z	12	
Unit cell dimensions	a = 7.0291(7) Å	α = 90 deg.
	b = 31.375(3) Å	β = 91.392(3) deg.
	c = 40.124(4) Å	γ = 90 deg.
Volume	8846.3(15) $Å^3$	
Density (calculated)	1.75 g/cm^3	
Absorption coefficient	5.30 mm^{-1}	
Crystal shape	needle	
Crystal size	0.367 x 0.154 x 0.025 mm^3	
Crystal colour	green	
Theta range for data collection	1.0 to 23.3 deg.	
Index ranges	$-7\leq h\leq 7$, $-34\leq k\leq 34$, $-44\leq l\leq 44$	
Reflections collected	68236	
Independent reflections	12691 (R(int) = 0.1642)	
Observed reflections	8164 ($I > 2\sigma(I)$)	
Absorption correction	Semi-empirical from equivalents	
Max. and min. transmission	0.68 and 0.46	
Refinement method	Full-matrix least-squares on F^2	
Data/restraints/parameters	12691 / 2613 / 1087	
Goodness-of-fit on F^2	1.10	
Final R indices (I>2sigma(I))	R1 = 0.077, wR2 = 0.138	
Largest diff. peak and hole	1.58 and -1.90 $eÅ^{-3}$	

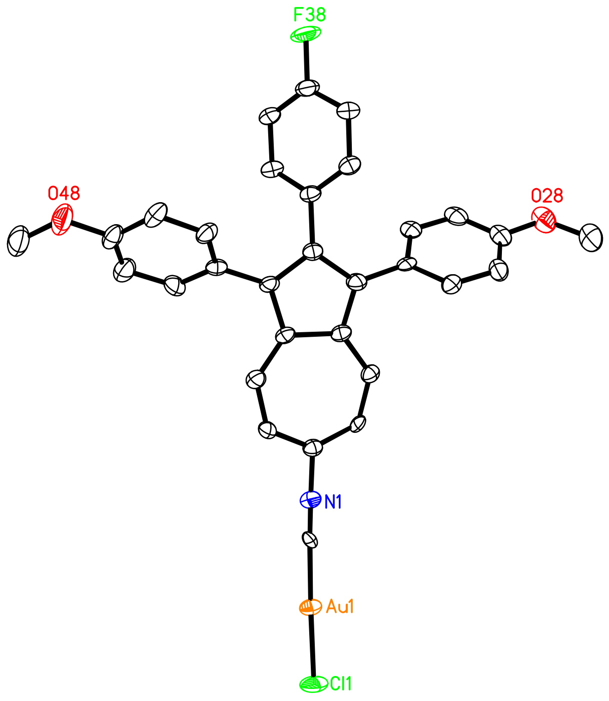

6.4 Single Crystal Structure of 14aa

Identification code	mad6	
Empirical formula	$C_{35.50}H_{34}AuCl_2FN_2O_2$	
Formula weight	807.51	
Temperature	200(2) K	
Wavelength	0.71073 Å	
Crystal system	tetragonal	
Space group	$P4_32_12$	
Z	8	
Unit cell dimensions	a = 9.5430(2) Å	α = 90 deg.
	b = 9.5430(2) Å	β = 90 deg.
	c = 70.4724(16) Å	γ = 90 deg.
Volume	6417.8(3) $Å^3$	
Density (calculated)	1.67 g/cm^3	
Absorption coefficient	4.79 mm^{-1}	
Crystal shape	plate	
Crystal size	0.117 x 0.049 x 0.022 mm^3	
Crystal colour	blue	
Theta range for data collection	1.2 to 27.4 deg.	
Index ranges	$-12 \leq h \leq 12$, $-12 \leq k \leq 12$, $-91 \leq l \leq 91$	
Reflections collected	67172	
Independent reflections	7332 (R(int) = 0.0829)	
Observed reflections	6551 ($I > 2\sigma(I)$)	
Absorption correction	Semi-empirical from equivalents	
Max. and min. transmission	0.91 and 0.78	
Refinement method	Full-matrix least-squares on F^2	
Data/restraints/parameters	7332 / 1 / 410	
Goodness-of-fit on F^2	1.23	
Final R indices (I>2sigma(I))	R1 = 0.039, wR2 = 0.085	
Absolute structure parameter	0.019(4)	

Largest diff. peak and hole	0.79 and -1.84 eÅ^{-3}

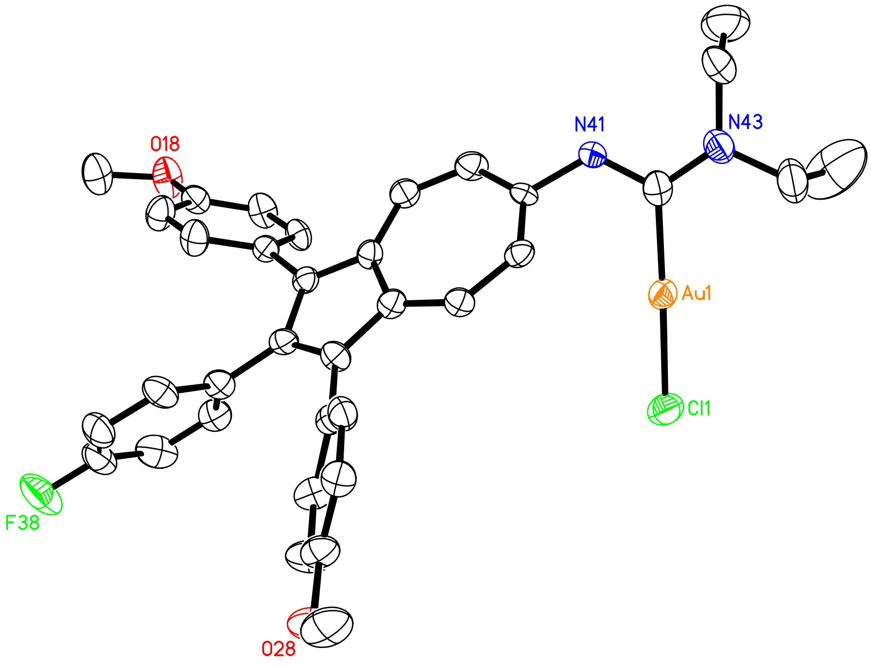

6.5 Single Crystal Structure of 14ba

Identification code	mad4	
Empirical formula	$C_{35}H_{33}AuClFN_2O_2$	
Formula weight	765.05	
Temperature	102(2) K	
Wavelength	0.71073 Å	
Crystal system	monoclinic	
Space group	$P2_1/c$	
Z	4	
Unit cell dimensions	a = 14.262(2) Å	α = 90 deg.
	b = 17.540(2) Å	β =107.296(6) deg.
	c = 12.714(2) Å	γ = 90 deg.
Volume	3036.7(8) Å^3	
Density (calculated)	1.67 g/cm^3	
Absorption coefficient	4.97 mm^{-1}	
Crystal shape	plank	
Crystal size	0.103 x 0.094 x 0.064 mm^3	
Crystal colour	blue	
Theta range for data collection	1.5 to 22.8 deg.	
Index ranges	$-15\le h\le 15$, $-18\le k\le 19$, $-13\le l\le 13$	
Reflections collected	14410	
Independent reflections	4097 (R(int) = 0.1380)	
Observed reflections	2470 ($I > 2\sigma(I)$)	
Absorption correction	Semi-empirical from equivalents	
Max. and min. transmission	0.91 and 0.64	
Refinement method	Full-matrix least-squares on F^2	
Data/restraints/parameters	4097 / 582 / 383	
Goodness-of-fit on F^2	1.01	
Final R indices (I>2sigma(I))	R1 = 0.057, wR2 = 0.113	

Largest diff. peak and hole	1.63 and -1.15 $eÅ^{-3}$

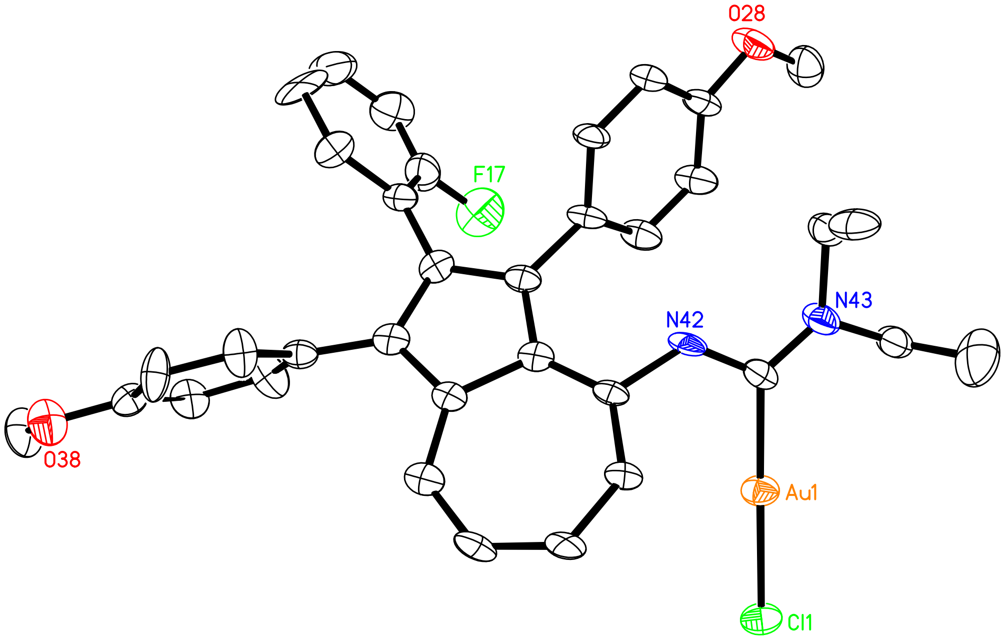

6.6 Single Crystal Structure of 14bd

Identification code	mad22	
Empirical formula	$C_{40.50}H_{34.50}AuClFN_2O_2$	
Formula weight	832.62	
Temperature	200(2) K	
Wavelength	0.71073 Å	
Crystal system	triclinic	
Space group	$P\bar{1}$	
Z	4	
Unit cell dimensions	a = 14.660(2) Å	α = 89.298(3) deg.
	b = 15.507(2) Å	β = 89.610(3) deg.
	c = 15.652(2) Å	γ = 88.852(4) deg.
Volume	3557.2(8) $Å^3$	
Density (calculated)	1.55 g/cm^3	
Absorption coefficient	4.25 mm^{-1}	
Crystal shape	plank	
Crystal size	0.075 x 0.025 x 0.015 mm^3	
Crystal colour	blue	
Theta range for data collection	1.3 to 20.5 deg.	
Index ranges	$-14\leq h\leq 14$, $-15\leq k\leq 15$, $-15\leq l\leq 15$	
Reflections collected	29217	
Independent reflections	7134 (R(int) = 0.0981)	
Observed reflections	4723 ($I > 2\sigma(I)$)	
Absorption correction	Semi-empirical from equivalents	
Max. and min. transmission	0.94 and 0.75	
Refinement method	Full-matrix least-squares on F^2	
Data/restraints/parameters	7134 / 1173 / 849	
Goodness-of-fit on F^2	1.04	
Final R indices (I>2sigma(I))	R1 = 0.055, wR2 = 0.127	

Largest diff. peak and hole	1.83 and -1.42 eÅ^{-3}

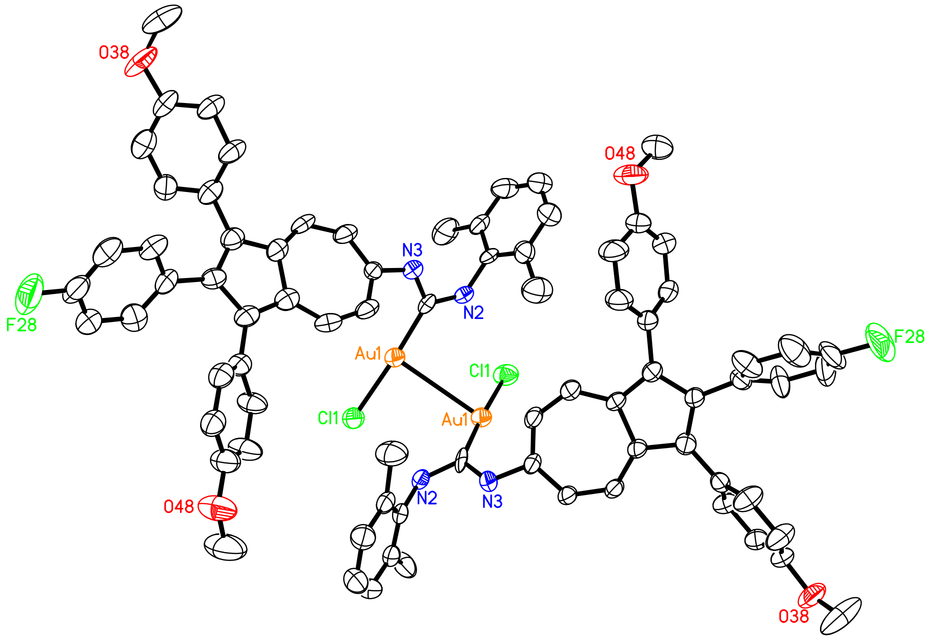

7 IC_{50} plots for complex 14aa

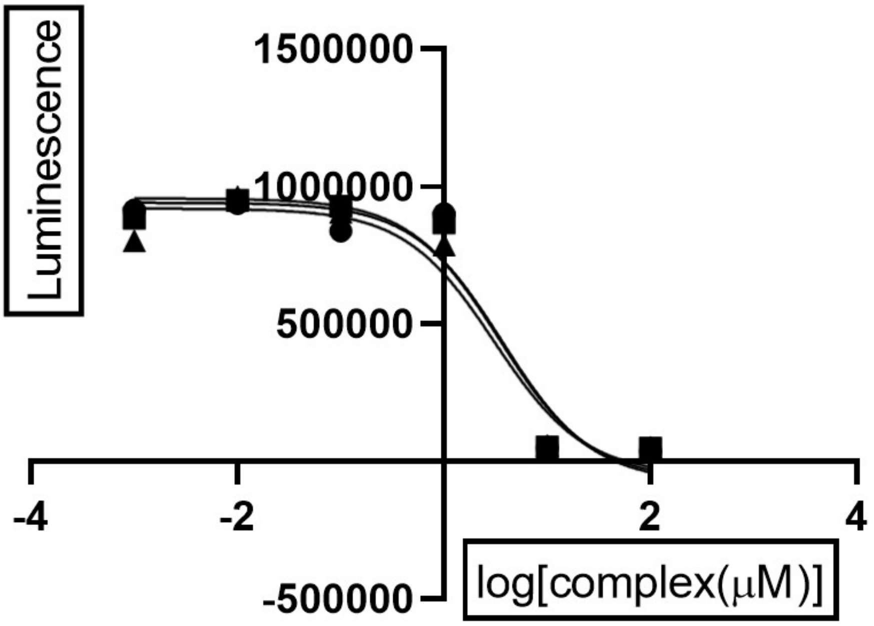

Figure S117. Plot for A2780.

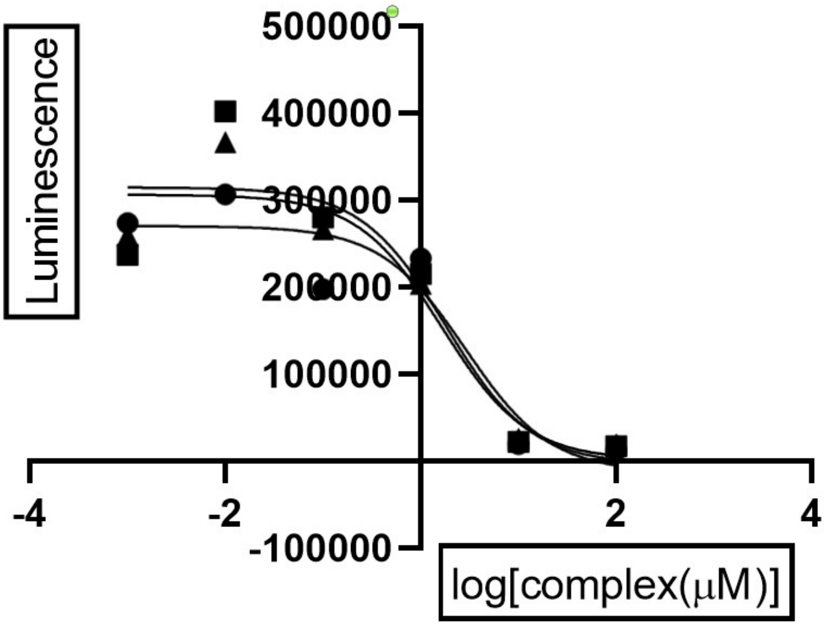

Figure S118. Plot for A2780cis.

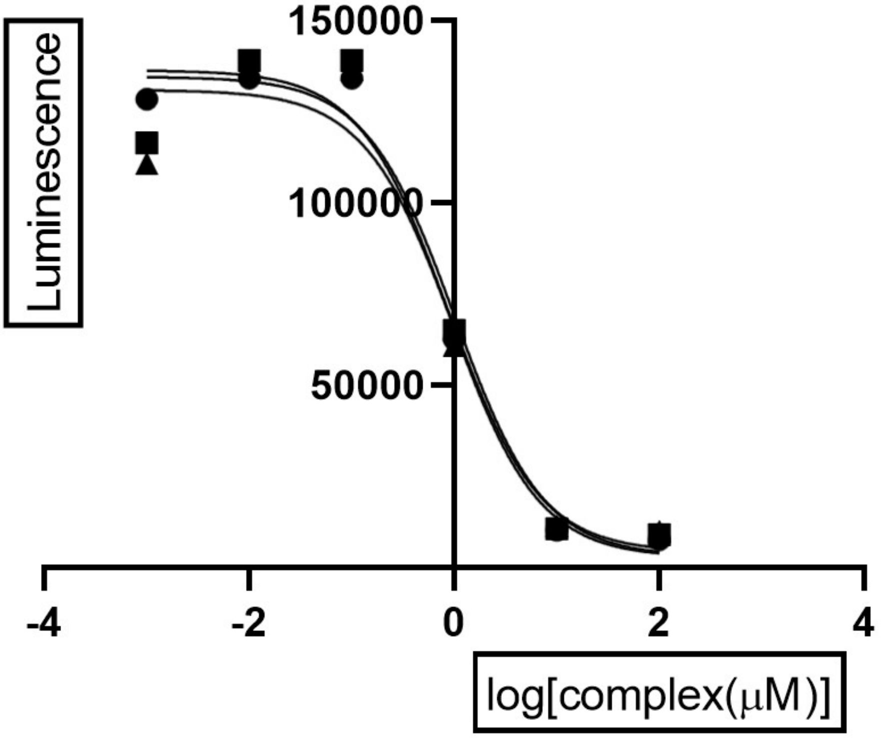

Figure S119. Plot for MDA-MB-231.

U87

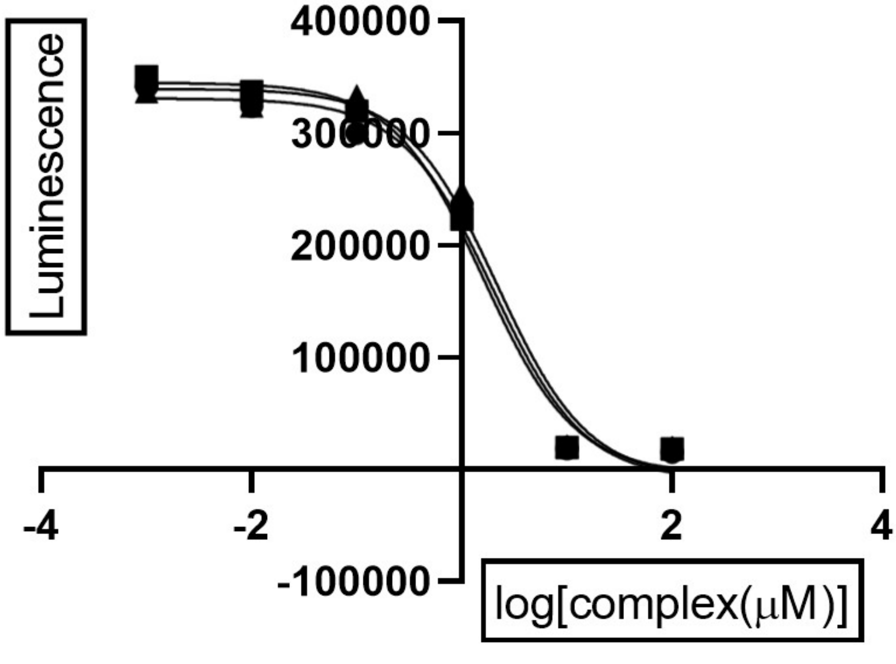

Figure S120. Plot for U87.

8 Stability of complexes 14ab and 14ba

To assess the stability of the used complexes in solution for the antiproliferative studies, exemplarily complexes 14ab and 14ba were dissolved in a 100 mM saline solution in D_2O and DMSO-d_6 in the ratio 1:5 and were left to stand at room temperature. ^{1}H NMR spectra of the solution were measured after t = 0 h, 24 h, 48 h, 72 h and 96 h. No decomposition was observed.

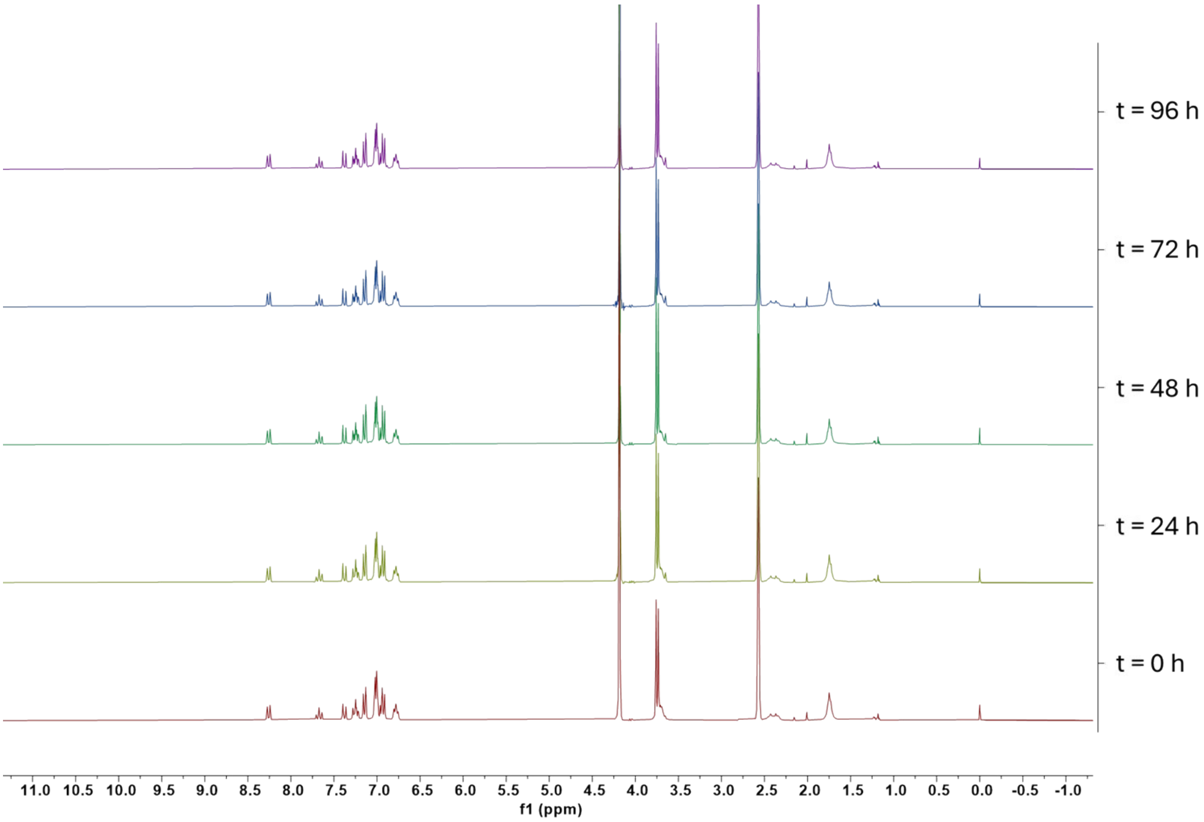

Figure S121. ^{1}H NMR Spectra (300 MHz, DMSO-d_6, 300 K) of **14ab** over the course of 96 h.

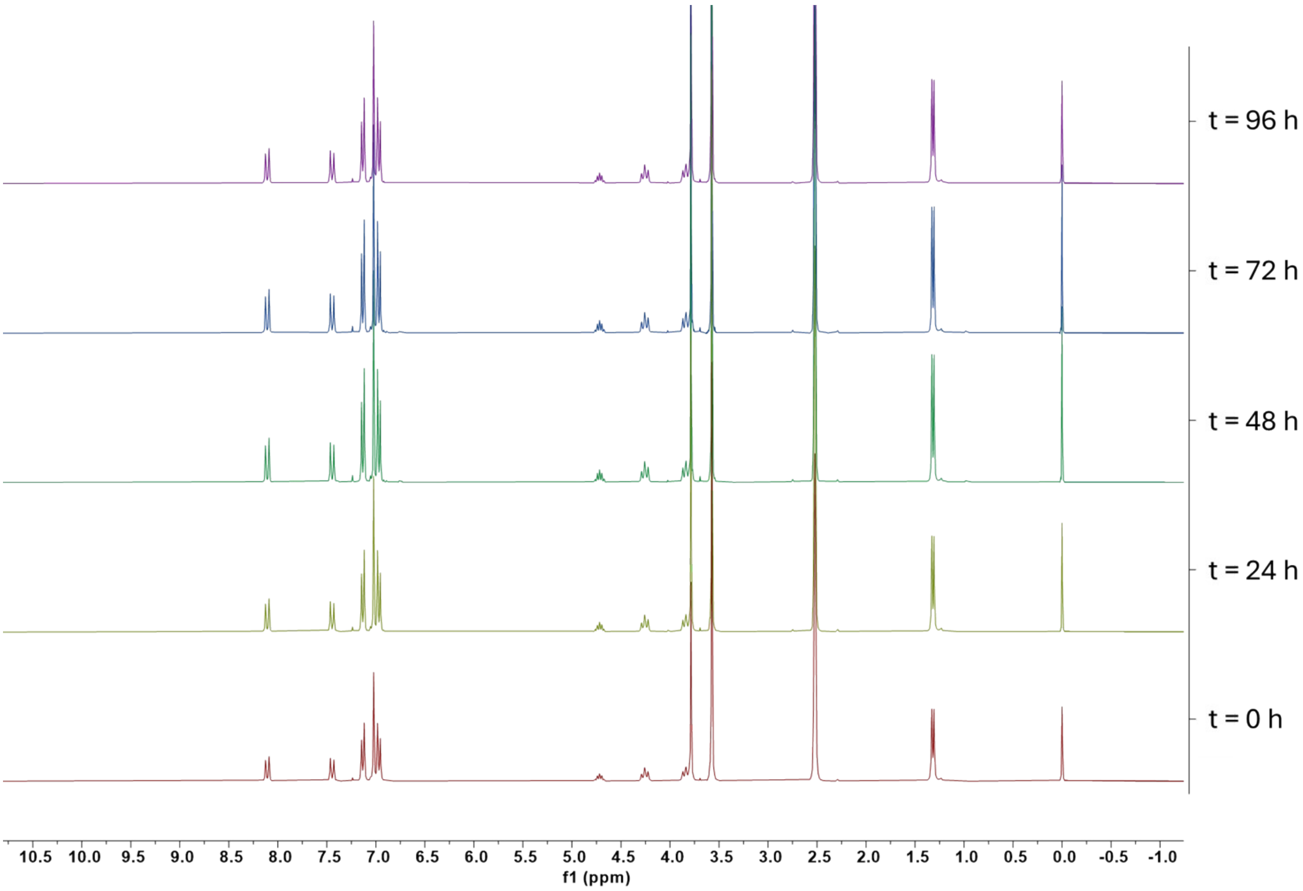

Figure S122. ^{1}H NMR Spectra (300 MHz, DMSO-d_6, 300 K) of **14ba** over the course of 96 h.

9 References

[1] G. Berthon-Gelloz, M. A. Siegler, A. L. Spek, B. Tinant, J. N. H. Reek, I. E. Markó, *Dalton Trans.* 2010, **39**, 1444–1446.

[2] A. Gómez-Suárez, R. S. Ramón, O. Songis, A. M. Z. Slawin, C. S. J. Cazin, S. P. Nolan, *Organometallics* 2011, **30**, 5463–5470.

[3] A. Ahrens, J. Schwarz, D. M. Lustosa, R. Pourkaveh, M. Hoffmann, F. Rominger, M. Rudolph, A. Dreuw, A. S. K. Hashmi, *Chem. Eur. J.* 2020, **26**, 5280–5287.

[4] V. Claus, M. Schukin, S. Harrer, M. Rudolph, F. Rominger, A. M. Asiri, J. Xie and A. S. K. Hashmi, *Angew. Chem. Int. Ed.* 2018, **57**, 12966–12970.

[5] H. Cai and F. P. Guengerich, *Chem. Res. Toxicol.* 2000, **13**, 327–335.
